# Aryl Sulfamoyl [^18^F]Fluorides: Preparation via Base-Free SuFEx ^18^F‑Fluorination and Assessment of Their Applicability as PET Tracers

**DOI:** 10.1021/acs.jmedchem.6c00147

**Published:** 2026-05-07

**Authors:** Jan Bertram, Otari Gokhadze, Felix Neumaier, Qinyu Wang, Andreas Dreiling, Lukas Vieth, Heike Endepols, Bernd Neumaier, Boris D. Zlatopolskiy

**Affiliations:** † 28334 Forschungszentrum Jülich GmbH, Institute of Neuroscience and Medicine, Nuclear Chemistry (INM-5), Wilhelm-Johnen-Straße, 52425 Jülich, Germany; ‡ University of Cologne, Faculty of Medicine and Cologne University Hospital, Institute of Radiochemistry and Experimental Molecular Imaging, Kerpener Straße 62, 50937 Cologne, Germany; § Department of Nuclear Medicine, University of Cologne, Faculty of Medicine and University Hospital Cologne, Kerpener Straße 62, 50937 Cologne, Germany

## Abstract

*N*-Aryl sulfamoyl fluorides (ArSAFs) are hydrolytically stable motifs widely used in drug design, yet their suitability for radiotracer development has not been explored. Here, we report the efficient radiosynthesis of [^18^F]­ArSAFs by base-free sulfur­(VI) fluoride exchange (SuFEx), affording high molar activities from nanomolar precursor amounts. The resulting compounds exhibited excellent *in vitro* stability across a wide pH range and in human plasma. *In vivo* studies in mice confirmed high metabolic stability for selected *N*-alkyl-*N*-aryl derivatives. Additionally, several *N*_in_-[^18^F]­SAF-substituted tryptophan analogues showed up to 2–3-fold higher cellular uptake than the current gold standard [^18^F]­FET in U87 MG glioblastoma cells, and their accumulation was sensitive to inhibition of amino acid transporters. However, *N*_in_-[^18^F]­SAF-substituted indole derivatives exhibited rapid *in vivo* defluorination, independent of substitution pattern. In contrast, [^18^F]­SAF-substituted dihydrotryptophan and 4-(MeNH)­Phe derivatives showed high *in vivo* stability. These findings establish *N*-alkyl-*N*-aryl sulfamoyl [^18^F]­fluorides as robust and accessible scaffolds for radiotracer development.

## Graphical abstract

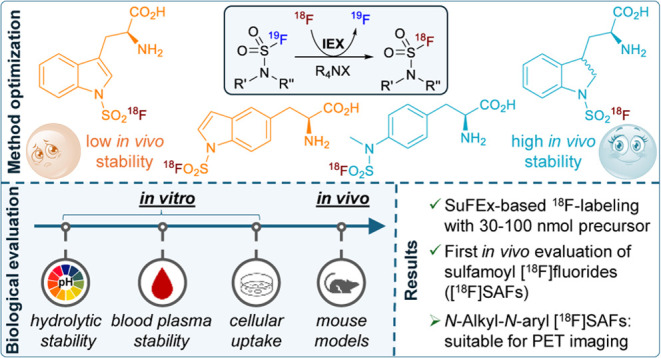

## 1 Introduction

Positron emission tomography (PET) is a noninvasive imaging modality that enables quantitative visualization of physiological and pathological processes by tracing the biodistribution of molecular probes labeled with β^+^-emitting radionuclides. Fluorine-18 (^18^F) represents the most widely used PET radionuclide, which can be attributed to its ready accessibility and favorable nuclear properties, such as a half-life (110 min) compatible with complex radiosyntheses, centralized production, and longitudinal measurements, as well as a low maximum positron energy (*E*_max_ = 635 keV) that translates into high spatial resolution.^1^ The continued demand for efficient and reliable ^18^F-labeling methods has resulted in significant methodological advances, such as the introduction of copper-mediated radiofluorination^2^ or minimalist labeling strategies based on onium salt precursors.^3^ Nevertheless, direct C–^18^F bond formation remains synthetically challenging and is often not suitable for radiolabeling of sensitive substrates. These limitations have fueled interest in alternative ^18^F-labeling strategies based on heteroatom–fluorine bond formation.^4^ In this context, sulfur­(VI)-fluoride exchange (SuFEx) chemistry^5^ has emerged as a particularly promising approach due to the high intrinsic stability of the S^VI^–F bond. Until 2021, the application of SuFEx in radiochemistry was largely limited to the preparation of metabolically labile aryl sulfonyl [^18^F]­fluorides.^6^ This changed with the report by Zheng et al. of a SuFEx-based isotopic exchange (IEX) method for the preparation of aryl [^18^F]­fluorosulfates (Scheme 1).^7^ Their protocol enabled rapid radiofluorination of 25 structurally diverse aryl fluorosulfates at ambient temperature, achieving radiochemical conversions (RCCs) of 83–100% and molar activities of up to 280 GBq μmol^–1^. In addition, *in vivo* imaging with an ^18^F-fluorosulfonylated olaparib analogue in tumor-bearing mice confirmed the potential of this functional group for PET tracer development.^7^

**Scheme 1:**
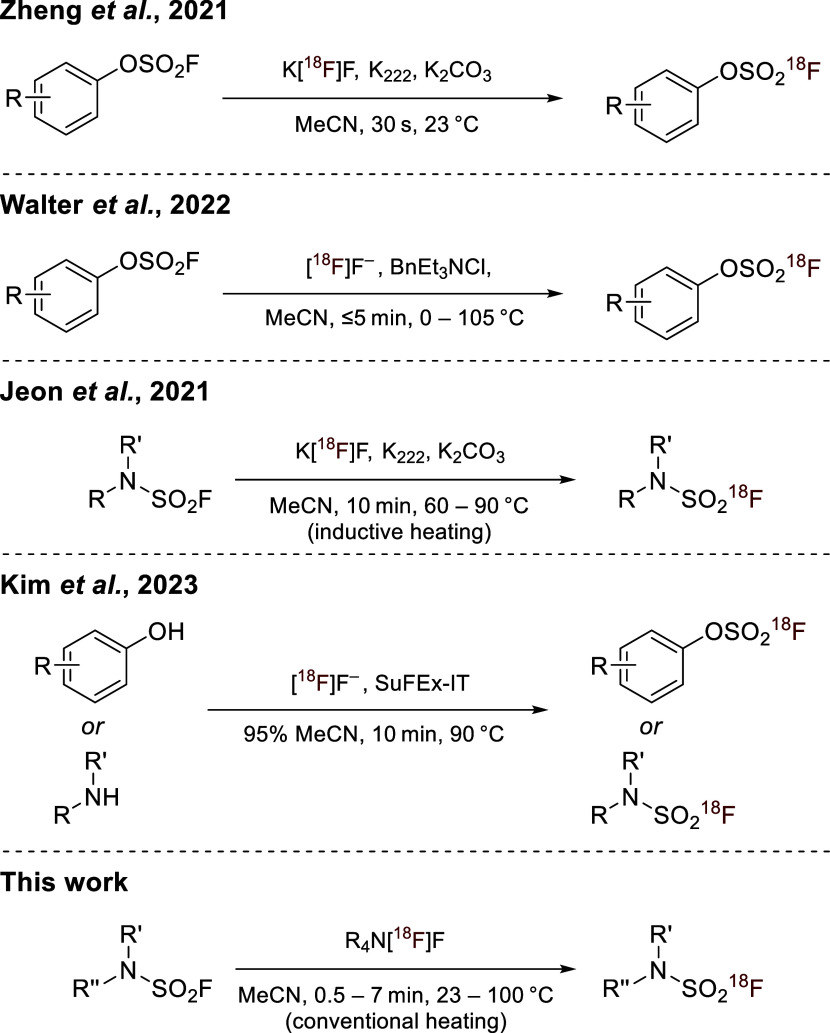
Protocols for the Preparation of ^18^F-Labeled Sulfamoyl Fluorides and Aryl Fluorosulfates.^7,8,11,12^

However, subsequent investigations revealed that the originally reported excellent RCCs were, in part, attributable to analytical conditions that led to nearly complete (>95%) adsorption of unreacted [^18^F]­fluoride during HPLC analysis, resulting in an overestimation of the radiolabeling efficiency.^8^ Under more suitable analytical conditions, the method based on basic K­[^18^F]­F/K_2_CO_3_/K_222_ showed substantially lower and less reproducible RCCs, necessitating the development of more effective approaches. To address this issue, we developed a base-free strategy for the elution of [^18^F]­fluoride ([^18^F]­F^–^) with neutral quaternary ammonium salts in MeOH (Scheme 1), which enabled efficient ^18^F-fluorination of diverse aryl fluorosulfates, including derivatives of complex biomolecules, while avoiding time-consuming azeotropic drying steps.^8^ Despite promising initial imaging results, the *in vivo* stability of the [^18^F]­fluorosulfate moiety was often insufficient, corroborating recent reports that aryl [^18^F]­fluorosulfates undergo substantial defluorination in human plasma and healthy mice.^9^

Due to their documented hydrolytic and thermal resistance, sulfamoyl [^18^F]­fluorides (SAFs) could represent a potentially more stable alternative to aryl [^18^F]­fluorosulfates.^5,10^ Jeon et al. recently introduced a SuFEx-based ^18^F/^19^F isotopic exchange method for the radiofluorination of SAFs using K­[^18^F]­F/K_2_CO_3_/K_222_ under inductive heating (Scheme 1), achieving RCCs of 50–97% across more than 30 SAF derivatives.^11^ Subsequently, the same group introduced a complementary strategy using the radiolabeled FSO_2_-transfer agent [^18^F]­SuFEx-IT for *in situ* generation of ^18^F-labeled fluorosulfates or SAFs from the corresponding phenols or amines (Scheme 1).^12^

Despite these advances, no *in vitro* or *in vivo* investigations of ^18^F-labeled SAFs have been reported to date. The present study therefore aimed to establish an efficient protocol for SAF labeling, evaluate the chemical and metabolic stability of the resulting compounds, and assess their suitability for PET tracer development. Notably, we assumed that application of our base-free SuFEx radiofluorination protocol to the production of [^18^F]­SAFs could significantly reduce the required precursor amounts compared to existing methods, which rely on disproportionally high precursor loadings (up to 20 μmol, see Scheme 1 in ref 11).

## 2 Results and Discussion

### 2.1 Development of a Base-Free SuFEx Labeling Strategy for Sulfamoyl Fluorides

To assess the applicability of our base-free SuFEx labeling strategy to sulfamoyl fluorides (SAFs), we initially employed Boc-Trp-OMe- and Boc-Trp-O*t*Bu-derived SAFs (**1** and **2**) as model substrates. Efficient ^18^F-labeling was achieved within 2 min at moderate temperatures (40–60 °C) under conventional heating, affording RCCs exceeding 80% (Figure 1A–C). As shown in Figure 1B (left panel), increasing the reaction temperature from 25 to 40 °C improved the RCCs for labeling of the *tert*-butyl ester **1** by approximately 10% after 5 min. Further elevation to 60 °C did not result in additional improvement, whereas temperatures above 60 °C led to decreased RCCs, presumably due to partial decomposition of **1** and [^18^F]**1**. Similar results were obtained for the methyl ester **2** (Figure 1B, right panel), although the decrease of RCCs at higher temperatures was more pronounced due to ester cleavage (see Figure S5 in the Supporting Information (SI)). Time-course experiments performed with **2** at 40 °C (Figure 1C) indicated that equilibrium was reached within 2 min, with no further improvement of RCCs observed for longer reaction times. Notably, the amount of the elution salt BnEt_3_NCl could be reduced from 10 to 2 μmol, although some increase in variability was observed (Figure 1D). We also evaluated three alternative elution salts previously identified as effective for fluorosulfate labeling.^8^ While Et_4_NOTf afforded RCCs comparable to BnEt_3_NCl, application of Et_4_NOH or Bu_4_NClO_4_ resulted in moderately reduced (about 10%) or highly inconsistent conversions, respectively (Figure 1D).

**Figure 1:**
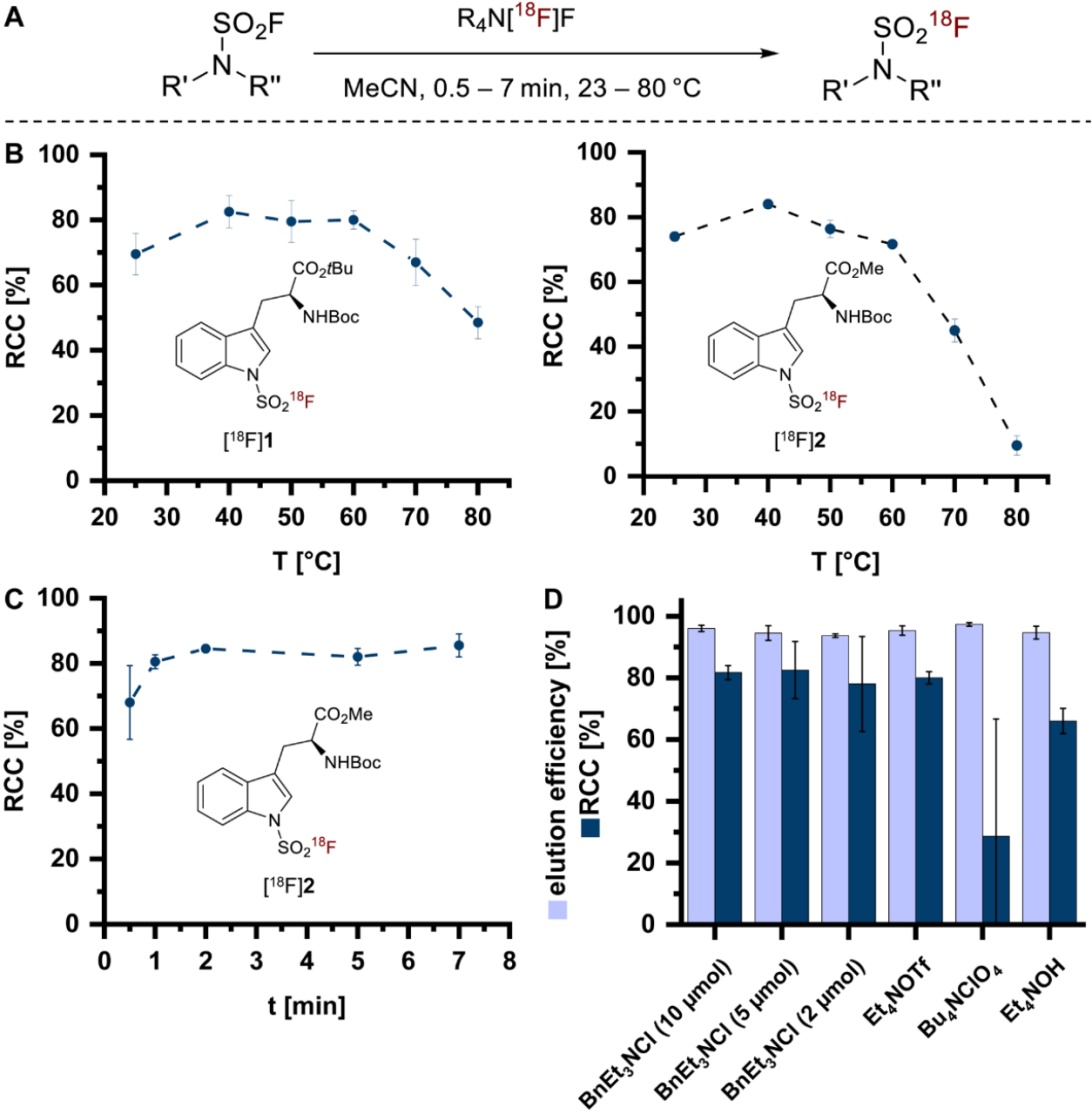
Application of the base-free SuFEx labeling strategy for the radiofluorination of sulfamoyl fluorides. (A) General conditions for the preparation of sulfamoyl [^18^F]­fluorides. (B) Dependency of radiochemical conversions (RCCs) for labeling of **1** (left panel) or **2** (right panel) on reaction temperature (conditions: 30 nmol, 5 min). Note that the methyl ester **2** was insufficiently stable at higher temperatures due to ester cleavage (for details, see Figure S5 in the Supporting Information). (C) Dependency of RCCs for labeling of **2** (conditions: 30 nmol, 40 °C) on reaction time. (D) Dependency of RCCs for labeling of **2** on different elution salts (conditions: 40 °C, 5 min). General conditions for **A**–**D**: 1 mL MeCN, 10 μmol elution salt (if not stated otherwise) in 1 mL MeOH. Data in B–D are given as mean ± standard deviation (B, C: *n* ≥ 2; **D**: *n* ≥ 3).

Next, we sought to determine the minimal precursor amount required for efficient ^18^F-labeling, as this parameter directly impacts the achievable molar activity (*A*_m_) in isotopic exchange protocols. Assessment of the labeling efficiency as a function of precursor loading demonstrated that Boc-Trp­(SO_2_[^18^F]­F)-OMe ([^18^F]**2**) could be prepared in high RCCs (>80%) using as little as 31 nmol precursor (Figure 2A). For the indoline derivative [^18^F]**3**, precursor amounts of 100 nmol were sufficient, although higher temperatures (80 °C) and longer reaction times (10 min) were required to achieve high RCCs (Figure 2A). In contrast, labeling of phenylpiperidyl sulfamoyl fluoride (**4**) required ≥ 2 μmol precursor to obtain RCCs above 50% (Figure 2A), resulting in low molar activities and highlighting a limitation of SuFEx-based isotopic exchange protocols for the preparation of aliphatic [^18^F]­SAFs.

**Figure 2:**
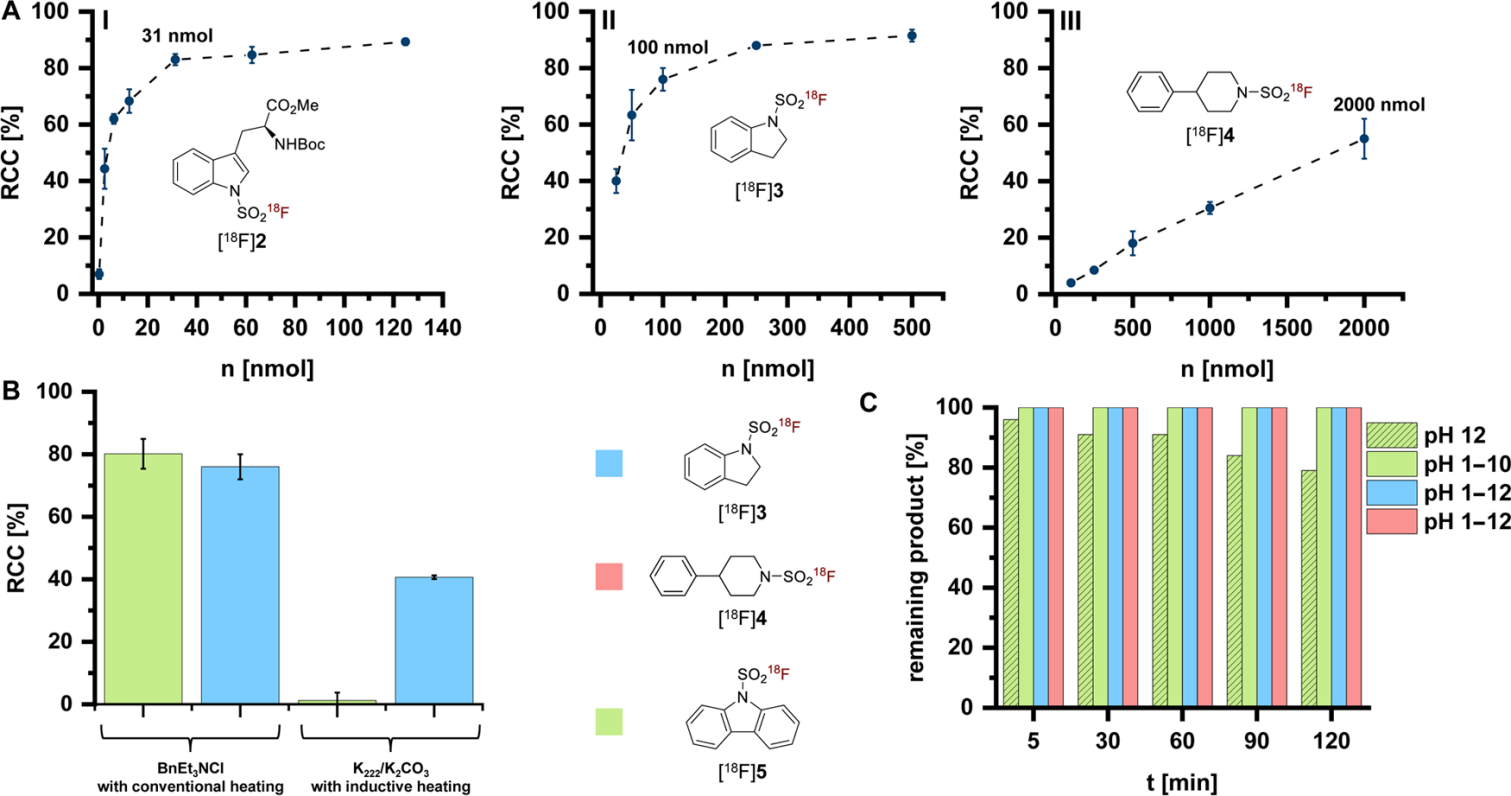
Performance of the base-free SuFEx labeling strategy and hydrolytic stability of sulfamoyl [^18^F]­fluorides. (A) Dependency of radiochemical conversions (RCCs) on precursor amount for three different substrates (**2**, **3**, and **4**). Conditions: **I**: 40 °C, 5 min, 1 mL MeCN; **II**: 80 °C, 10 min, 1 mL MeCN; **III**: 80 °C, 10 min, 1 mL MeCN. (B) Comparison of the K_222_/K_2_CO_3_-based labeling approach under inductive heating (conditions: 100 nmol precursor, 2 mL MeCN; [^18^F]**3** and [^18^F]**5**: 60 °C, 10 min) with the base-free approach using conventional heating (conditions: 100 nmol precursor, 2 mL MeCN; [^18^F]**5**: 40 °C, 3 min; [^18^F]**3**: 80 °C, 10 min). (C) Hydrolytic stability of [^18^F]**3**–**5** at different pH values (1–12) for 2 h at ambient temperature. For [^18^F]**3** and [^18^F]**4**, no hydrolysis was observed at any tested pH value, and therefore identical values (100% intact) are shown as single bars per time point for clarity. For [^18^F]**5**, no hydrolysis was observed at pH 1–10, while partial hydrolysis occurred at pH 12, which is displayed separately. Data in A and B are given as mean ± standard deviation (*n* ≥ 2).

To further benchmark the base-free protocol, we compared it with the previously reported procedure of Jeon et al.,^11^ which applies K_222_/K_2_CO_3_ and inductive heating (Monowave 50, Anton Paar). For the indoline derivative [^18^F]**3**, RCCs obtained under the optimized base-free conditions were nearly 2-fold higher (Figure 2B). Even more strikingly, the carbazole derivative [^18^F]**5**, which could not be prepared under basic conditions when using nanomolar precursor amounts, was produced in high RCCs of about 80% using BnEt_3_NCl (Figure 2B). It should be noted, however, that successful base-mediated labeling of **5** has been reported at micromolar precursor loading (e.g., 2 μmol), albeit with lower RCCs and at correspondingly lower attainable molar activities.^11^ These findings highlight the improved performance and broader substrate scope of the optimized base-free protocol when using nanomolar precursor amounts.

Next, we evaluated the hydrolytic stability of [^18^F]**3**–**5**. As summarized in Figure 2C, [^18^F]**3** and [^18^F]**4** remained stable across pH 1–12, while [^18^F]**5** showed mild degradation at pH 12 (21% defluorination after 120 min). Importantly, however, all three compounds exhibited excellent stability in phosphate-buffered saline (PBS, pH 7.4), with no detectable defluorination for up to 2 h.

### 2.2 Substrate Scope and Structure–Reactivity Relationship

To further assess the scope of the radiofluorination protocol, we applied it to a broader set of SAF substrates (Figure 3). Labeling of indole-derived SAFs (**2**, **5**, and **8**) consistently afforded high RCCs (71–83%) using minimal precursor amounts (30 nmol) and mild reaction conditions (40 °C). This trend persisted in the presence of electron-donating (**6**) or withdrawing (**7**) groups on the indole moiety. *N*-Alkyl-*N*-aryl SAFs (**3**, **9**, **13**, and **14**) required slightly higher precursor loadings (100 nmol) along with moderately elevated temperatures (80–90 °C) and reaction times (10 min) to achieve moderate-to-high RCCs (38–76%). Labeling of substrates with electron-donating groups in *para*-position (**10**, **11**) required even higher temperatures (100 °C), while the electron-deficient SAF **12** had to be labeled at lower temperatures to avoid decomposition and side-product formation. In contrast, the *N*-imidazolyl SAF Boc-His­(SO_2_F)-OMe (**15**) was effectively labeled at ambient temperature within just 1 min (RCC = 69 ± 5%). Notably, increasing the reaction temperature to 40 °C resulted in a significant decrease of RCCs to 52 ± 1% after 1 min of incubation and only 6 ± 4% after 5 min of incubation, indicating low stability of [^18^F]**15** under radiolabeling conditions.

**Figure 3:**
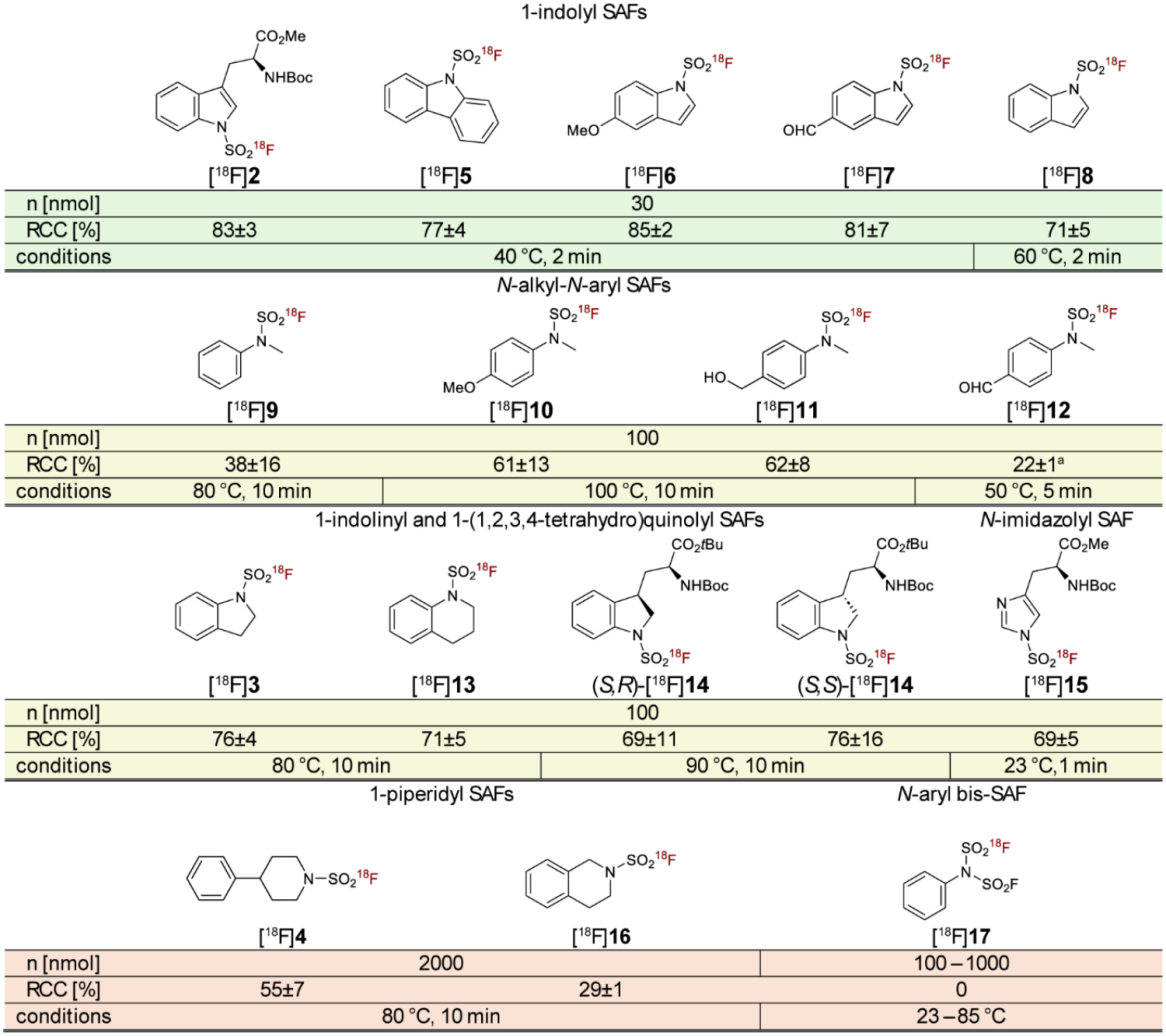
Substrate scope of the base-free SuFEx radiolabeling protocol under the indicated reaction conditions using BnEt_3_NCl (10 μmol) in MeOH (1 mL) for [^18^F]­fluoride elution. All radiolabeling experiments were conducted at least in triplicate. Data are given as mean ± standard deviation (*n* ≥ 3). ^a^ (*n* = 2). Abbreviations: RCC: radiochemical conversion; SAF: sulfamoyl fluoride.

In line with prior observations (Figure 2A), aliphatic SAFs like **4** and **16** proved to be less reactive, requiring precursor amounts in the micromolar range to achieve meaningful RCCs. Attempts to label (fluorosulfonyl)­(phenyl)­sulfamoyl fluoride (**17**), which contains a bis-SO_2_F-substituted amino group, were unsuccessful under a variety of reaction conditions, with chromatographic analysis indicating complete decomposition of the precursor. Similarly, its monosubstituted analogue already showed signs of degradation prior to use. These findings are consistent with the reported susceptibility of *N*-monosubstituted SAFs to hydrolysis,^5^ suggesting that such compounds are unsuitable for PET tracer development.

Due to the low attainable molar activities or pronounced instability, we excluded 1-piperidyl, *N*-imidazolyl, and monosubstituted SAFs from further evaluation and focused our subsequent studies on *N*-aryl substituted SAFs (ArSAFs). Notably, these findings suggest an inverse relationship between isotopic exchange efficiency and hydrolytic stability within this compound class. While *N*,*N*-dialkyl-substituted [^18^F]­SAFs may exhibit enhanced intrinsic stability, the low attainable molar activities using the SuFEx-IEX protocol currently limit its suitability for the preparation of such probes with high molar activity. Development of alternative radiolabeling strategies enabling efficient access to *N*,*N*-dialkyl-substituted [^18^F]­SAFs therefore represents a promising direction for future work.

### 2.3 Preclinical Evaluation of Representative [^18^F]­ArSAFs

To assess the *in vivo* stability of radiolabeled ArSAFs, we selected Boc-Trp­(SO_2_[^18^F]­F)-OMe ([^18^F]**2**) as a representative model compound. The objective of this experiment was to evaluate metabolic stability and potential defluorination of the [^18^F]­SAF motif *in vivo* rather than to investigate transporter-mediated uptake. Although the corresponding *tert*-butyl ester showed slightly higher stability at elevated temperatures (Figure 1), the methyl ester ([^18^F]**2**) was selected for *in vivo* evaluation as reduced stability was only observed at temperatures exceeding physiological limits. Following isolation by solid-phase extraction (SPE) and formulation, [^18^F]**2** was obtained in activity yields (AYs; non-decay-corrected) of 24 ± 2% and radiochemical purities (RCPs) of 99%, within a total production time of 50 min (Figure 4A). An *A*_m_ of 6.7 GBq/μmol was determined for 200 MBq of tracer (see SI). Preclinical PET imaging in healthy mice was performed to evaluate potential *in vivo* defluorination. As the objective at this stage was stability assessment rather than tumor targeting, evaluation in healthy animals was considered appropriate. The results demonstrated pronounced accumulation of [^18^F]**2** in the liver (maximum SUV_bw_ 381 ± 79, 0–30 min p.i.), gall bladder (maximum SUV_bw_ 1571 ± 301, 90–120 min p.i.), and intestine (maximum SUV_bw_ 975 ± 429, 90–120 min p.i.), consistent with hepatobiliary clearance of the highly lipophilic compound (Figure 4B). Importantly, bone uptake of radioactivity remained low throughout the whole observation period (maximum SUV_bw_ 133 ± 11, 90–120 min p.i.), indicating a high *in vivo* stability of [^18^F]**2** against defluorination.

**Figure 4:**
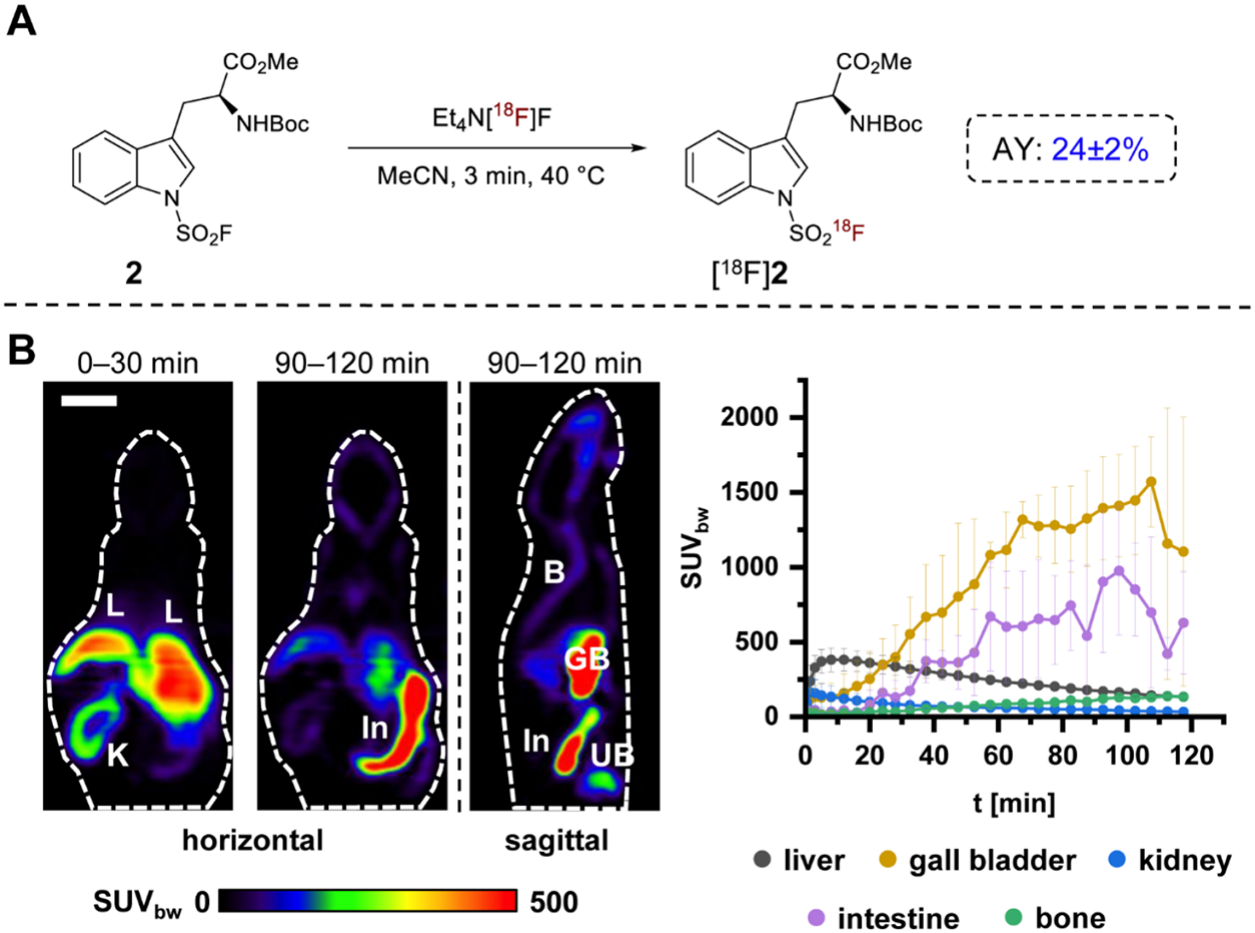
Preparation and preclinical evaluation of Boc-Trp­(SO_2_[^18^F]­F)-OMe ([^18^F]**2**). (A) Radiosynthesis of [^18^F]**2**. (B) *In vivo* evaluation of [^18^F]**2** by μPET imaging in healthy mice. Shown are representative horizontal and sagittal PET images (summed over the indicated 30 min time frames) of the same mouse (left) and time-activity curves (mean ± standard deviation, *n* = 3) for radioactivity accumulation in the indicated tissues (right). For blood time-activity curves, see Figure S6 in the Supporting Information. Scale bar: 1 cm. Abbreviations: K: kidney, L: liver; In: intestine; GB: gall bladder; UB: urinary bladder; B: bone.

Encouraged by the favorable *in vivo* stability, we next explored the applicability of the radiolabeling protocol for the preparation of clinically relevant PET tracers. Radiolabeled amino acids serve as valuable tools for visualizing the upregulation of amino acid transporters in various tumors, particularly within the central nervous system. Among these, *O*-(2-[^18^F]­fluoroethyl)­tyrosine ([^18^F]­FET) is routinely used for brain tumor imaging due to its selective tumor uptake via amino acid transporters like l-type amino acid transporter 1 (LAT1), a member of system L. To develop alternative tracers targeting these transporters, we prepared three radiolabeled ArSAF-substituted amino acids. To this end, Boc-Trp-OH was converted to *tert*-butyl ester **18**,^13^ which was *N*_in_-fluorosulfonylated with desmethyl SuFEx-IT,^14^ affording precursor **1** in 13% yield over two steps (99% purity based on HPLC). For preparation of the 2,3-dihydrotryptophan-derived precursors, (*S*,*S*)- and (*S*,*R*)-**14**, Boc-Trp-O*t*Bu was reduced with NaBH_3_CN and the resulting diastereoisomers were separated by column chromatography. Subsequent *N*-fluorosulfonylation with desmethyl SuFEx-IT furnished the desired precursors in a total yield of 17% for (*S*,*S*)-**14** and 10% for (*S*,*R*)-**14**, respectively (Scheme 2A). The intended preparation of a SAF-His derivative was impeded by unsuccessful synthesis of the corresponding reference compound H-His­(SO_2_F)-OH from Boc-His­(SO_2_F)-O*t*Bu (**S2**). Specifically, acidic deprotection resulted in partial cleavage of the FO_2_S-group and formation of histidine (for details see Supporting Information), while other SAF-substituted amino acids studied remained intact under such conditions.

**Scheme 2:**
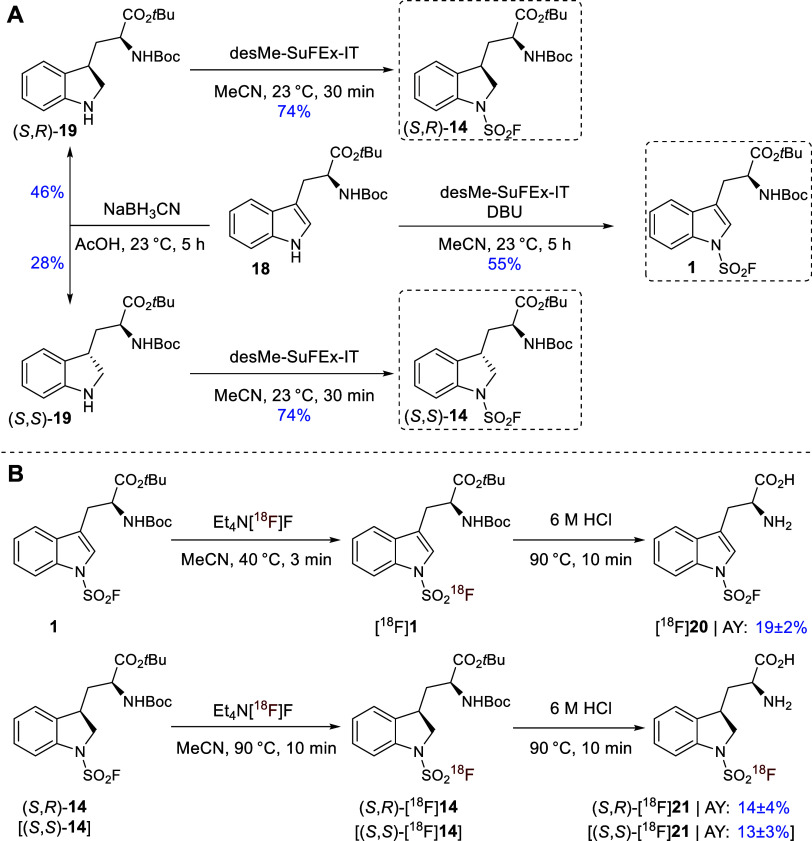
Application of the Base-Free SuFEx Labeling Strategy for the Preparation of Amino Acid-Based PET Tracers^a^

a (A) Preparation of radiolabeling precursors **1**, (*S,R*)-**14**, and (*S,S*)-**14**. (B) Radiosynthesis of amino acid tracers [^18^F]**20**, (*S,S*)-[^18^F]**21**, and (*S,R*)-[^18^F]**21**. Activity yields (AYs) are given as mean ± standard deviation (*n* ≥ 3).

*N*_in_-[^18^F]­Fluorosulfonyl-tryptophan {H-Trp­(SO_2_[^18^F]­F)-OH; [^18^F]**20**} was prepared by isotopic exchange on precursor **1**, followed by acidic deprotection of the radiolabeled intermediate [^18^F]**1**. Subsequent isolation by SPE and formulation as an injectable solution afforded the desired tracer in AYs of 19 ± 2% within 55 min (Scheme 2B). The diastereomers of *N*_1_-[^18^F]­fluorosulfonyl-2,3-dihydrotryptophan {(*S*,*S*)-H-Dht­(SO_2_[^18^F]­F)-OH; (*S*,*S*)-[^18^F]**21**; and (*S,R*)-H-Dht­(SO_2_[^18^F]­F)-OH; (*S*,*R*)-[^18^F]**21**} were prepared from (*S*,*S*)- and (*S*,*R*)-**14** using analogous conditions and obtained in AYs of 13 ± 3% {(*S*,*S*)-[^18^F]**21**} and 14 ± 4% {(*S*,*R*)-[^18^F]**21**} within 70 min (Scheme 2B). All three tracer candidates were obtained in RCPs of >98%.

The hydrolytic stability and cellular uptake of the candidate probes were examined *in vitro*. [^18^F]**20** exhibited a high stability at pH 5–10 (<4% defluorination, 2 h) but suffered from substantial (>50%, 2 h) defluorination at pH 12 (Figure 5A, left panel). (*S*,*S*)-[^18^F]**21** remained completely intact at pH 5–10 and showed negligible defluorination (3%) at pH 12 (Figure 5A, right panel). The hydrolytic stability of the protected histidine derivative [^18^F]**15** was also investigated, as the corresponding deprotected analogue could not be obtained (see above). In contrast to [^18^F]**20** and (*S*,*S*)-[^18^F]**21**, [^18^F]**15** already exhibited pronounced defluorination (>30%, 2 h) at pH 8.5, indicating increased susceptibility of the SAF-imidazolyl moiety toward hydrolysis. Together with its instability under acidic deprotection conditions, these results demonstrate that SAF-imidazolyl derivatives are insufficiently stable for practical applications and were therefore excluded from further biological evaluation. Conversely, the compounds [^18^F]**20** and (*S*,*S*)-[^18^F]**21** also demonstrated excellent stability in human plasma, with no defluorination detected over a period of 2 h. Next, cellular uptake of the candidate tracers by U87 MG glioblastoma cells over a period of 1 h was compared to that of the established tracer [^18^F]­FET (Figure 5B). Both diastereomers of H-Dht­(SO_2_[^18^F]­F)-OH exhibited approximately 2-fold higher cellular accumulation than [^18^F]­FET, with the (*S,S*)-diastereomer showing slightly superior uptake (Figure 5B). Strikingly, H-Trp­(SO_2_[^18^F]­F)-OH ([^18^F]**20**) demonstrated even a 3-fold higher uptake relative to [^18^F]­FET (Figure 5B), highlighting its potential for brain tumor imaging. To elucidate the amino acid transport systems mediating tracer uptake, we performed competitive inhibition assays with U87 MG cells and inhibitors targeting system L, system ASC (alanine-serine-cysteine transport system) and system A (a Na^+^-dependent neutral amino acid transport system), which are among the principal transport pathways implicated in cellular uptake of α-amino acids (Figure 5C). 2-Aminobicyclo[2.2.1]­heptane-2-carboxylic acid (BCH), a competitive inhibitor of system L transporters like LAT1 that can also affect the Na^+^-dependent transport systems B^0^ and B^0,+^,^15,16^ strongly reduced cellular accumulation of all three candidate probes in a concentration-dependent manner. l-Serine, which is frequently employed as a substrate of system ASC, also reduced tracer accumulation, although the effect was less pronounced. However, l-serine is not exclusively selective for system ASC and has been reported to interact with system L transporters, particularly LAT2.^17^ A very similar inhibition profile was observed for [^18^F]­FET, whose uptake is typically predominantly attributed to system L (LAT1 and, to a lesser extent, LAT2), with additional contributions from systems B^0^ and B^0,+^.^16,18^ Therefore, while these findings do not permit definitive assignment of individual transporter subtypes, they indicate that the novel tracer candidates have a transport profile similar to that of [^18^F]­FET. Interestingly, inhibition of system A with α-(methylamino)­isobutyric acid (MeAIB) selectively reduced uptake of (*S,R*)-[^18^F]**21**, suggesting an influence of the configuration of the distant stereocenter on the interaction with amino acid transporters. Additionally, the increase in cellular uptake of [^18^F]**20** observed at higher MeAIB concentrations is consistent with amino acid counter-transport (trans-stimulation), a characteristic feature of exchanger-type transport systems such as system L.^19^ This finding further supports the involvement of amino acid transport mechanisms rather than passive diffusion. However, given the complexity and overlap of amino acid transport systems in glioma cells, additional contributions from other transport pathways, including lysosomal amino acid transporters (e.g., system H) or system T, cannot be excluded.^20^ Definitive assignment of individual transporter subtypes would require further inhibition studies or transporter-specific knockdown experiments.

**Figure 5:**
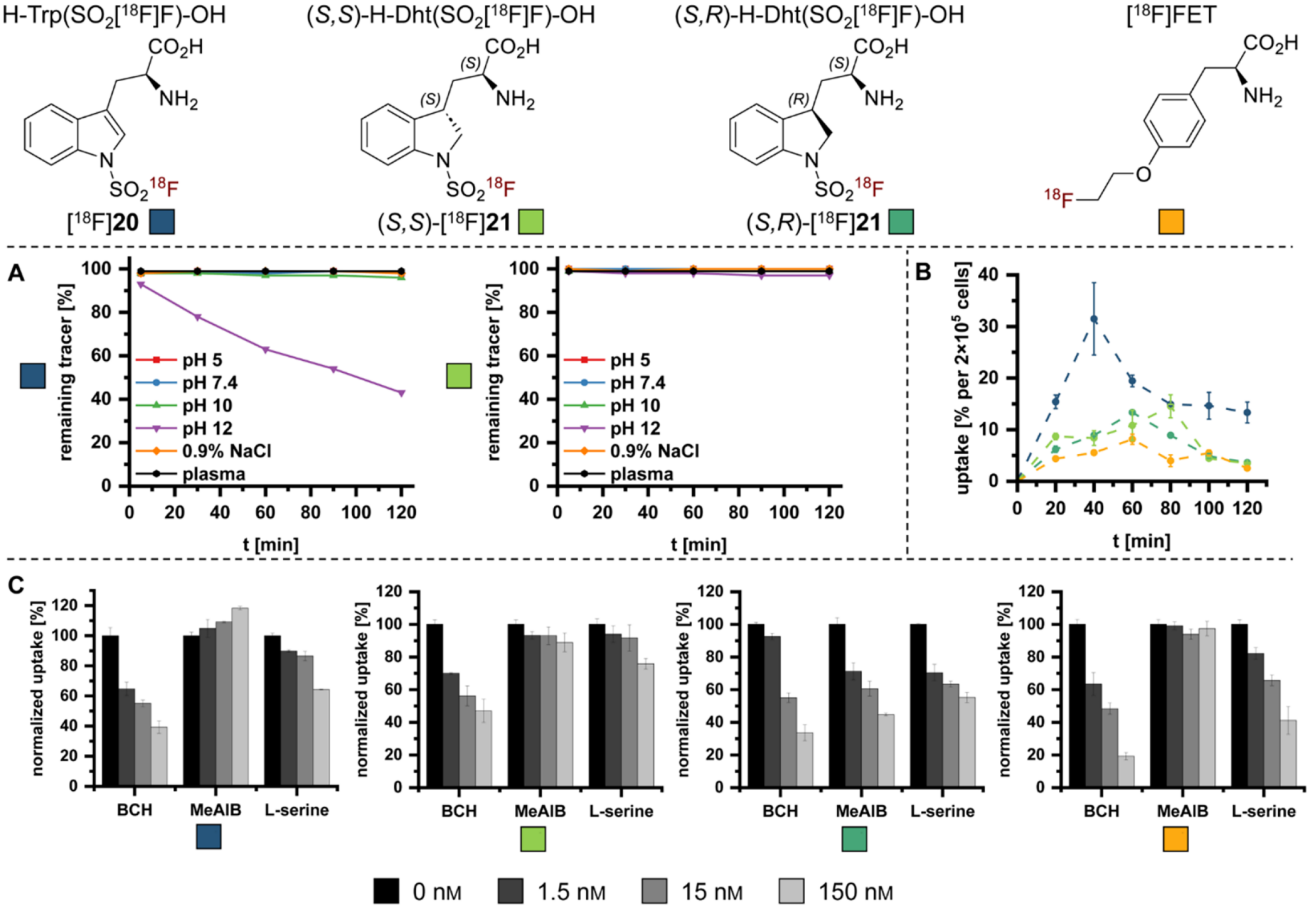
Hydrolytic stability and cellular uptake of ArSAF-based PET tracer candidates. (A) Hydrolytic stability of [^18^F]**20** (left) and (*S,S*)-[^18^F]**21** (right) at different pH values (5–12), in isotonic saline, and in human plasma over 2 h. (B) Uptake of [^18^F]**20**, (*S,S*)- and (*S,R*)-[^18^F]**21** in comparison to [^18^F]­FET by human U87 MG glioblastoma cells. Uptake was quantified after incubation of the cells with 150 kBq of the different radiolabeled probes for up to 2 h. (C) Effect of different amino acid transport inhibitors on the uptake of (from left to right) [^18^F]**20**, (*S,S*)- and (*S,R*)-[^18^F]**21**, and [^18^F]­FET by U87 MG glioblastoma cells. Uptake was quantified after incubation of the cells with 150 kBq of the tracers for 1 h in the presence of the indicated inhibitor concentrations and normalized to the mean value observed without inhibitor (0 nm). The inhibitors used were 2-aminobicyclo[2.2.1]­heptane-2-carboxylic acid (BCH) for system L, l-serine for system ASC, and α-(methylamino)­isobutyric acid (MeAIB) for system A.

Finally, (*S,S*)-H-Dht­(SO_2_[^18^F]­F)-OH {(*S*,*S*)-[^18^F]**21**} and H-Trp­(SO_2_[^18^F]­F)-OH ([^18^F]**20**) were evaluated by *in vivo* PET imaging in a subcutaneous tumor xenograft mouse model based on the same U87 MG cell line as used for the uptake studies. (*S*,*R*)-[^18^F]**21** was not further investigated, as it exhibited slightly lower cellular uptake *in vitro* and possesses physicochemical properties comparable to those of its diastereomer, suggesting similar *in vivo* stability. As illustrated in Figure 6, both investigated tracers enabled visualization of the tumors within 30 min, although tumor uptake was lower than that of the reference tracer [^18^F]­FET. Notably, high skeletal accumulation of radioactivity was observed for [^18^F]**20**, indicative of significant *in vivo* defluorination. Specifically, PET images acquired with [^18^F]**20** revealed prominent vertebral radioactivity accumulation that was already evident shortly after tracer injection and increased over time (see representative sagittal PET images at 90–120 min p.i. in Figure 6A). In addition, bone time-activity curves for [^18^F]**20** showed a progressive increase throughout the whole measurements, reaching SUV_bw_ values of 201 ± 37 at 90–120 min p.i. (Figure 6B). In contrast, (*S,S*)-[^18^F]**21** showed substantially lower bone uptake, suggesting superior *in vivo* stability of this tracer (maximum SUV_bw_ 42 ± 16). One possible explanation for the lower stability of H-Trp­(SO_2_[^18^F]­F)-OH is that it undergoes facilitated SuFEx reaction with nucleophilic side chains of amino acid residues during interaction with proteins, leading to [^18^F]­F^–^ release and accounting for the pronounced *in vivo* defluorination.

**Figure 6:**
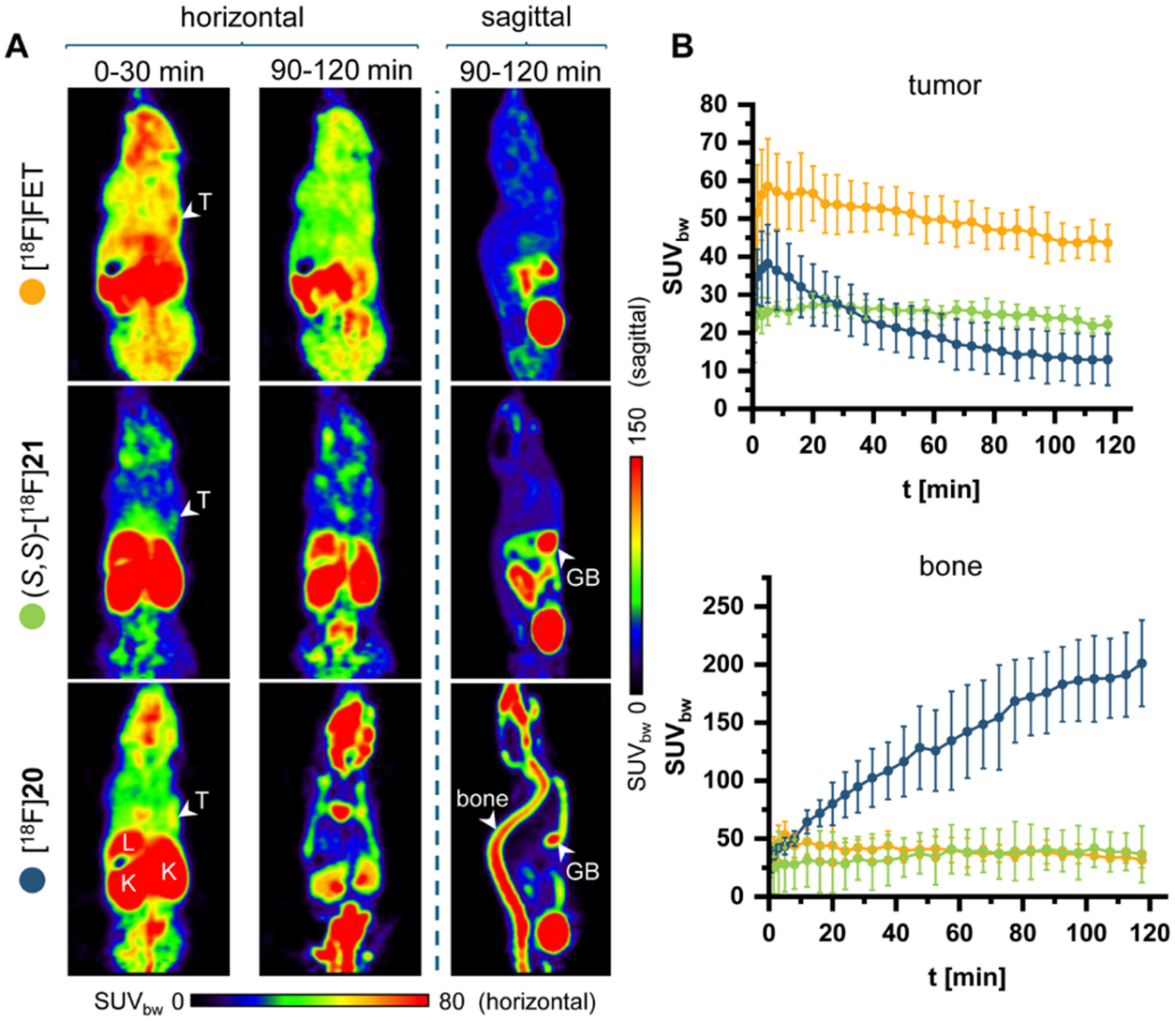
Comparison of *in vivo* tumor uptake of [^18^F]**20**, (*S,S*)-[^18^F]**21**, and [^18^F]­FET in a subcutaneous U87 MG glioblastoma mouse model. (A) Representative horizontal and sagittal PET images (summed over the indicated 30 min time frames) of the same tumor-bearing mouse measured (on different days) with [^18^F]­FET (top), (*S,S*)-[^18^F]**21** (middle), and [^18^F]**20** (bottom). (B) Comparison of time-activity curves (mean ± standard deviation, *n* = 3 for each tracer) for tumoral and bone accumulation of radioactivity in measurements with [^18^F]**20** (blue), (*S,S*)-[^18^F]**21** (green), and [^18^F]­FET (orange). Abbreviations: GB: gall bladder, K: kidney, L: liver, T: tumor.

To mitigate the observed defluorination of H-Trp­(SO_2_[^18^F]­F)-OH ([^18^F]**20**), we explored structural modification of the indole scaffold. Given the complete stability of the tracers under physiologically relevant pH conditions and in freshly prepared blood plasma, simple hydrolytic cleavage and/or cleavage via interaction with low-molecular-weight nucleophiles or proteins present in circulation appears unlikely to explain the observed *in vivo* defluorination. Considering the well-established electrophilic character of sulfur­(VI) fluorides, interactions with nucleophilic amino acid residues (e.g., Ser, Cys, Tyr, or His) within proteins or enzymatic microenvironments may represent a plausible alternative pathway for S–^18^F bond cleavage.^21^ We hypothesized that shifting the Ala-residue from the third to the fifth position of the indole ring could alter the orientation of the tracer during interaction with proteins. Such a modification should reduce the likelihood of a productive alignment between the *N*_in_-fluorosulfonyl substituent and nucleophilic residues (like Ser, Tyr, Cys, and His). Based on this rationale, we selected *N*_in_-[^18^F]­SAF-3-(1*H*-5-indolyl)­alanine ([^18^F]**26**, H-5-*iso*-Trp­(SO_2_[^18^F]­F)-OH), a regioisomer of [^18^F]**20** expected to attenuate tracer degradation via protein-mediated [^18^F]­F^–^ release. The radiolabeling precursor **25** was prepared as follows (Scheme 3). Negishi cross-coupling between Boc-3-I-Ala-OMe and 5-bromoindole according to a protocol of Dachwitz et al.^22^ afforded Boc-5-*iso*-Trp-OMe (**22**), which was hydrolyzed to Boc-5-*iso*-Trp-OH (**23**). The latter was esterified with dicyclopropylmethanol in the presence of EDC/DMAP to obtain the corresponding Dcpm ester (**24**),^23^ which was *N*_in_-fluorosulfonylated with AISF^24^ to furnish the radiolabeling precursor **25** in 10% yield over four steps.

**Scheme 3:**
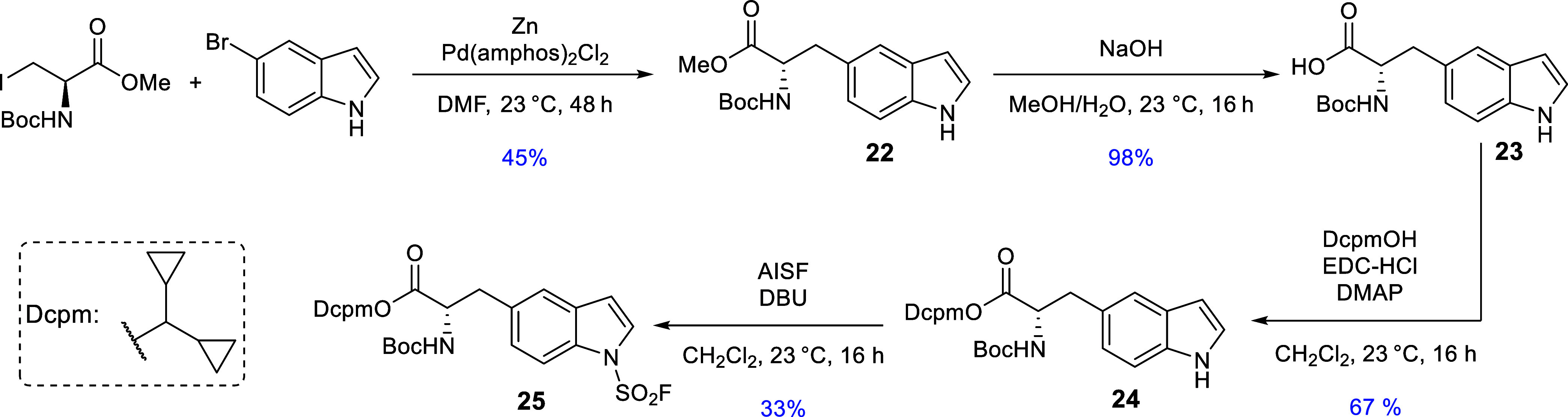
Preparation of Boc-5-*iso*-Trp­(SO_2_F)-ODcpm (**25**)

HCl·H-5-*iso*-Trp­(SO_2_F)-OH (**26**·HCl) was prepared by acidic deprotection of **23**, which afforded the reference compound in 92% yield. The radiolabeled amino acid [^18^F]**26** was produced from **25** using the same SuFEx-based protocol as described for [^18^F]**20** (AY: 13 ± 8, *n* = 2, Figure 7A) and evaluated in healthy mice (Figure 7B). However, radioactivity uptake in bones was comparable to that observed for [^18^F]**20** (maximum SUV_bw_ 243 ± 36, 90–120 min p.i., Figure 7B), suggesting that indole-substituted SAFs are intrinsically more susceptible to *in vivo* defluorination and exhibit reduced stability compared to the corresponding indoline analogues. These findings further support the observed inverse relationship between isotopic exchange efficiency and *in vivo* stability. Structural optimization may therefore be required to improve *in vivo* stability and unlock the translational potential of this subclass.

**Figure 7:**
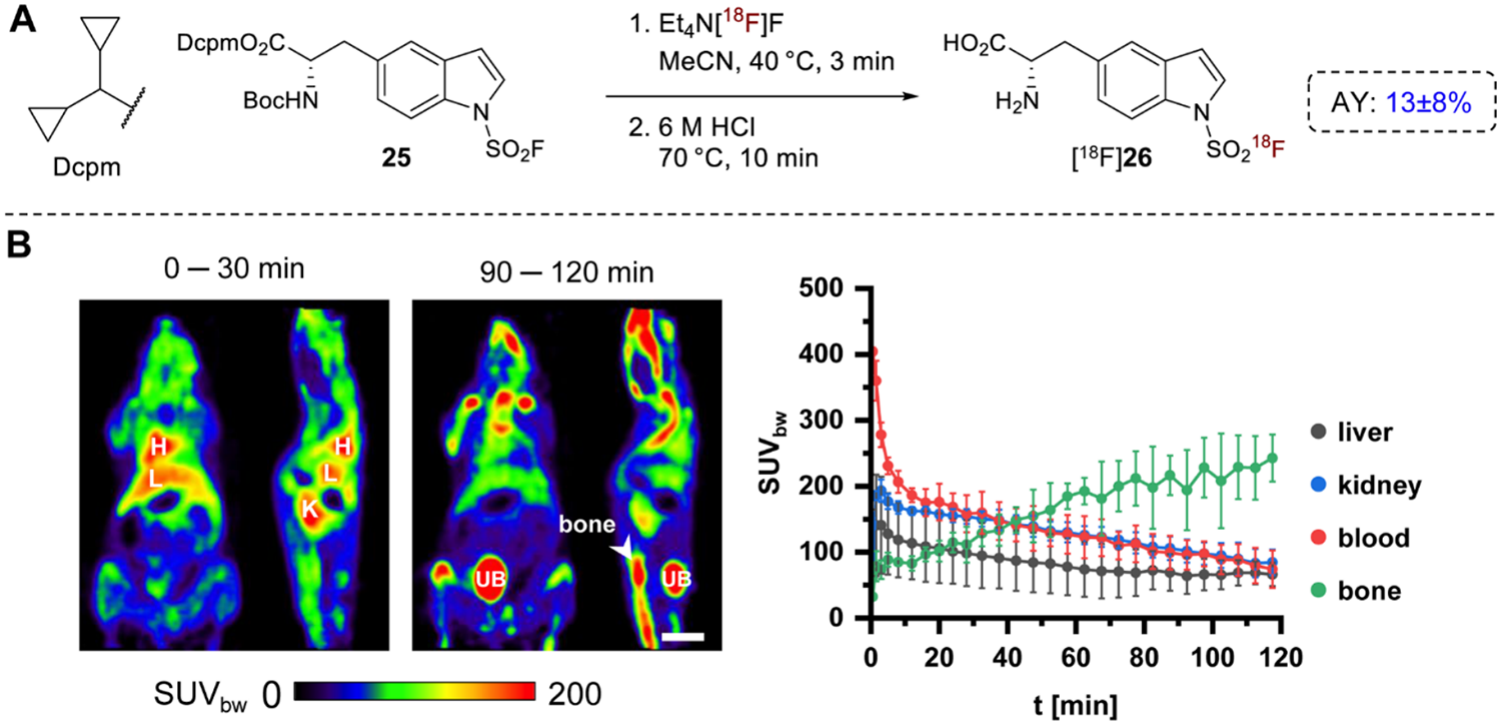
Preparation and preclinical evaluation of H-5-iso-Trp­(SO_2_[^18^F]­F)-OH ([^18^F]**26**). (A) Radiosynthesis of [^18^F]**26**. (B) *In vivo* evaluation of [^18^F]**26** by μPET imaging in healthy mice. Shown are representative horizontal and sagittal PET images (summed over the indicated 30 min time frames) of one mouse (left) and time-activity curves (mean ± standard deviation, *n* = 4) for radioactivity accumulation in the indicated tissues (right). Scale bar: 1 cm. Abbreviations: H: heart; K: kidney; L: liver; UB: urinary bladder.

Finally, to confirm the superior stability of *N*-alkyl-*N*-aryl SAFs seen with (*S,S*)-[^18^F]**21**, we prepared and evaluated the [^18^F]­FET mimic H-4-(NMeSO_2_[^18^F]­F)­Phe-OH ([^18^F]**33**). For synthesis of the corresponding radiolabeling precursor **32** (Scheme 4), 4-(methylamino)­benzaldehyde^25^ was reduced with NaBH_4_ and the resulting 4-(methylamino)­benzyl alcohol (**28**) was fluorosulfonylated with desmethyl SuFEx-IT^14^ to obtain the alcohol **11**. Subsequent bromination of **11** with CBr_4_/PPh_3_ using the Appel reaction^26^ afforded benzyl bromide **29**, which was applied for alkylation of a commercially available (*S*)-Ni-BPB-Gly complex according to a known protocol.^27^ Although the fluorosulfonylated Ni-complex **30** thus obtained could be directly used for the preparation of [^18^F]**33** (for the application of Ni-BPB-AA complexes as precursors for the production of radiofluorinated amino acids, see, e.g., Craig et al.^27^ and Krasikova et al.^28^), this approach afforded only moderate RCCs of 33 ± 1% (*n* = 2, 500 nmol, 90 °C, 10 min) and resulted in the formation of several radiofluorinated side products. Accordingly, we instead pursued the protected precursor **32** by decomposing **30** with DTPA­(Et_4_N)_2_, followed by *N*-Boc protection of the intermediate H-4-(NMeSO_2_F)­Phe-OH.^29^ Subsequent esterification of the resulting Boc-4-(NMeSO_2_F)­Phe-OH (**31**) with *tert*-butyl trichloroacetimidate afforded the desired radiolabeling precursor **32** in a total yield of 48% over seven steps.

**Scheme 4:**
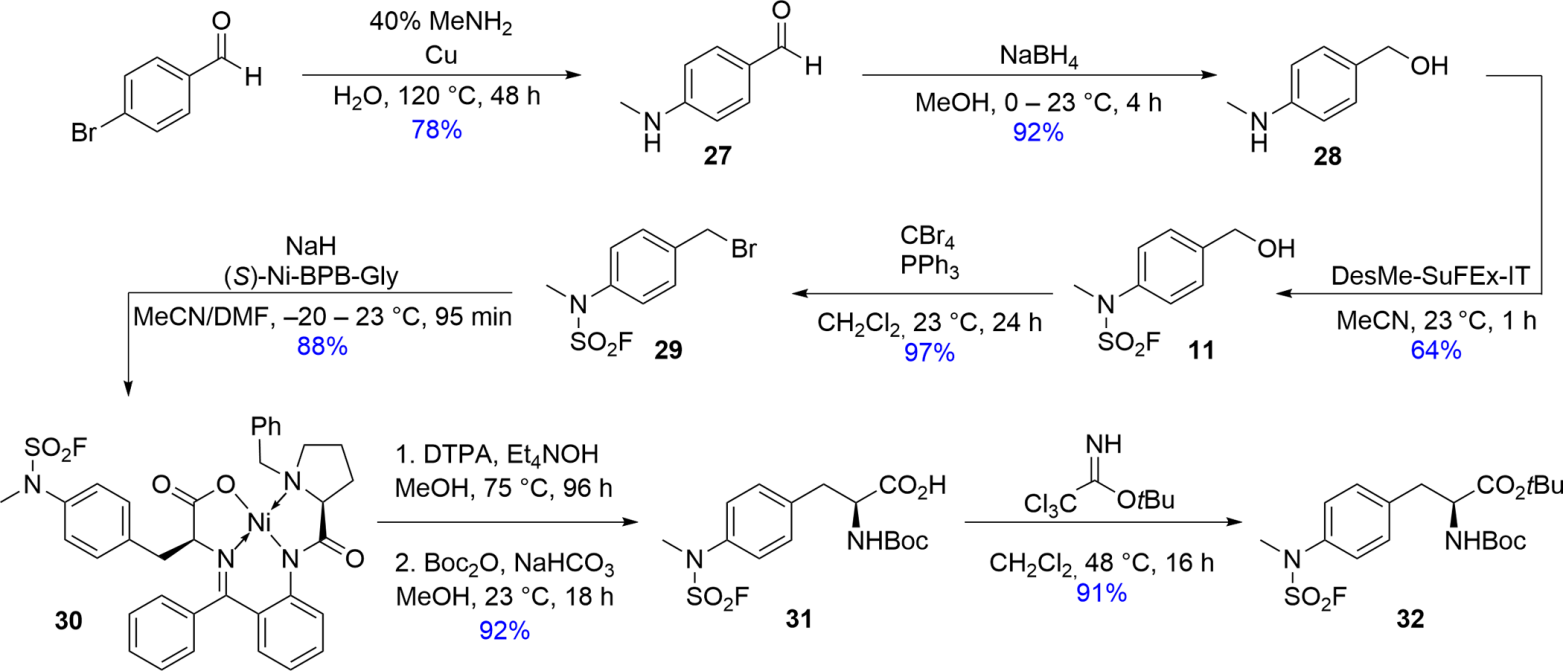
Preparation of Boc-4-(NMeSO_2_F)­Phe-O*t*Bu (**32**)

The analytically pure reference compound **33**·HCl was obtained in 93% yield by deprotecting **32** with gaseous HCl using a method described by Verschueren et al.^30^ [^18^F]**33** was produced by isotopic exchange on precursor **32** followed by acidic deprotection of the radiolabeled intermediate [^18^F]**32**, which afforded the tracer candidate in AYs of 22 ± 4% (Figure 8A, *n* = 2). Based on the optimization studies with model substrates, precursor loading was adjusted to 200 nmol, as experiments with 100 nmol **32** at 80 °C resulted in low RCC (30%), whereas higher temperatures led to byproduct formation. μPET imaging in healthy mice demonstrated predominantly renal clearance of [^18^F]**33**, with strong accumulation in the kidneys (maximum SUV_bw_ 168 ± 22, 30–60 min p.i., Figure 8B). More importantly, there was negligible bone uptake of radioactivity (maximum SUV_bw_ 52 ± 3, 60–90 min p.i., Figure 8B), confirming a high *in vivo* stability of the tracer. These results reaffirm the potential of *N*-alkyl-*N*-aryl-substituted SAFs as metabolically stable scaffolds, warranting further exploration of this structural motif for radiotracer development.

**Figure 8:**
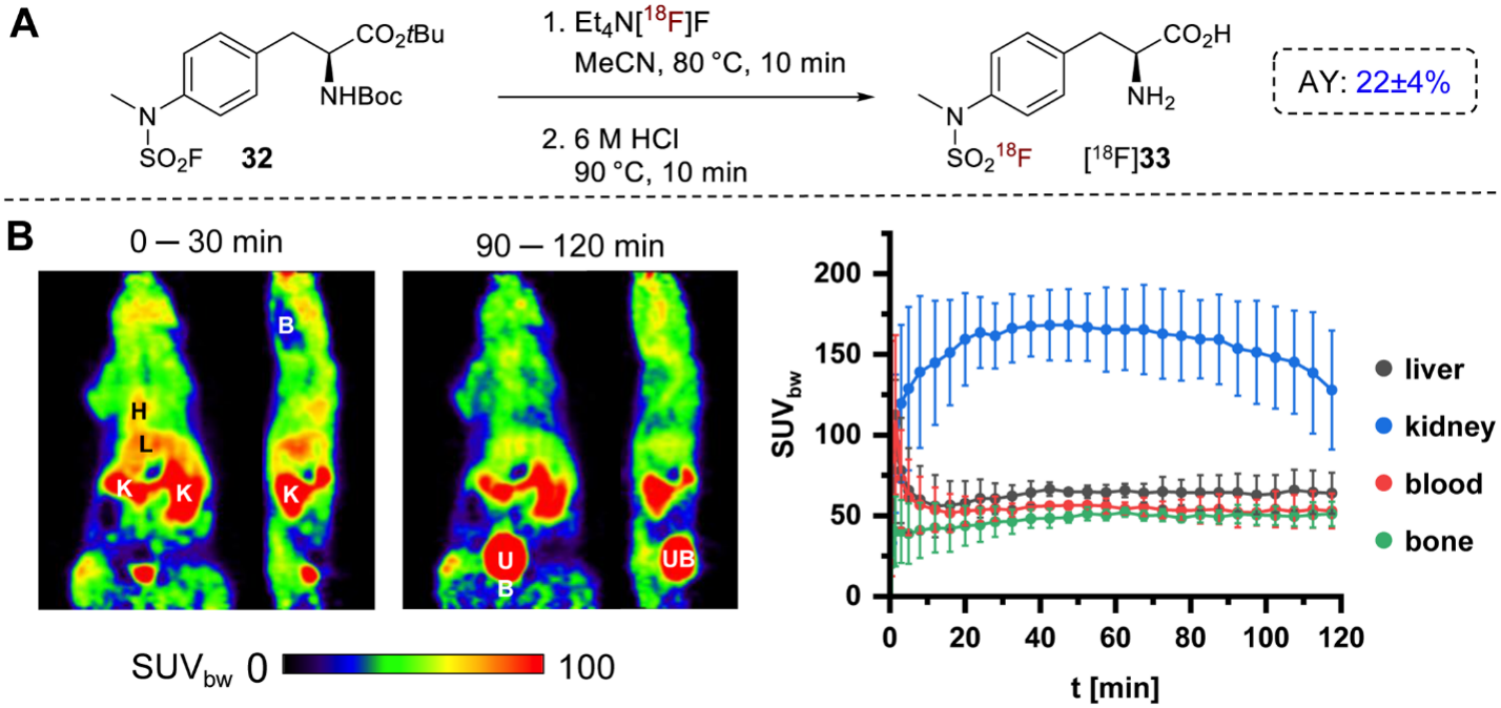
Preparation and preclinical evaluation of H-(NMeSO_2_[^18^F]­F)­Phe-OH ([^18^F]**33**). (A) Radiosynthesis of [^18^F]**33**. (B) *In vivo* evaluation of [^18^F]**33** by μPET imaging in healthy mice. Shown are representative horizontal and sagittal PET images (summed over the indicated 30 min time frames) of one mouse (left) and time-activity curves (mean ± standard deviation, *n* = 4) for radioactivity accumulation in the indicated tissues (right). Abbreviations: B: brain; H: heart; K: kidney; L: liver; UB: urinary bladder.

## 3 Conclusion

In this study, we successfully adapted a base-free SuFEx radiofluorination strategy to the labeling of *N*-aryl substituted sulfamoyl fluorides (ArSAFs) and demonstrated its utility for PET tracer development. Importantly, the presence of an *N*-aryl substituent enhanced labeling efficiency. The optimized protocol enabled efficient ^18^F-fluorination under mild conditions across a structurally diverse set of ArSAF substrates. High radiochemical conversions were achieved using minimal precursor amounts.

Stability assessments revealed that the evaluated ^18^F-labeled ArSAFs display excellent resistance to hydrolytic degradation over a broad pH range. *In vivo* evaluation of the protected amino acid derivative [^18^F]**2** confirmed high metabolic stability, with negligible defluorination in healthy mice. However, PET imaging with the tryptophan derivative [^18^F]**20** and its regioisomer [^18^F]**26** revealed substantial radioactivity accumulation in bones, suggesting that *N*-indolyl substituted ArSAFs may be prone to metabolic cleavage. In contrast, the *N*-alkyl-*N*-aryl substituted dihydro-analogue (*S,S*)-[^18^F]**21** and the [^18^F]­FET-mimetic [^18^F]**33** demonstrated markedly improved *in vivo* stability and remain promising candidates for further tracer development. In addition, the Trp-derived tracers [^18^F]**20** and (*S,S*)-[^18^F]**21** enabled clear tumor visualization *in vivo*, validating the general suitability of amino acid derivatives with a [^18^F]­SAF motif for oncological imaging. Finally, *in vitro* uptake studies indicated that cellular accumulation of the novel candidate tracers is mediated by amino acid transport systems, with an inhibition profile similar to that of [^18^F]­FET, although definitive assignment to individual transporter subtypes will require further investigation.

Taken together, these results establish secondary *N*-aryl sulfamoyl fluorides as a robust and tunable alternative to fluorosulfates for PET radiochemistry. The observed inverse relationship between isotopic exchange efficiency and hydrolytic stability also highlights an important design consideration for future development of sulfamoyl [^18^F]­fluoride-based PET tracers, particularly for *N*,*N*-dialkyl-substituted members of this class, for which alternative labeling strategies are required. Future studies will focus on expanding the structural repertoire of accessible [^18^F]­SAF-based probes to enhance their translational potential for molecular imaging applications.

## 4 Experimental Section

### 4.1 Organic Chemistry

#### 4.1.1 General

Unless noted otherwise, all chemicals and solvents were purchased from VWR International GmbH (Darmstadt, Germany), Sigma-Aldrich Chemie GmbH (Steinheim, Germany), ABCR GmbH (Karlsruhe, Germany), Apollo Scientific Ltd. (Bredbury, United Kingdom), Alfa Aesar GmbH (Karlsruhe, Germany), or BLD Pharmatech GmbH (Kaiserslautern, Germany) and used without further purification.

All reactions were carried out with magnetic stirring (unless noted otherwise) and organic extracts were dried over anhydrous MgSO_4_. Air- or moisture-sensitive reagents were handled under argon (>99.999%, Air Liquide GmbH, Düsseldorf, Germany). Solutions were concentrated under reduced pressure (1–900 mbar) at 40–50 °C using a rotary evaporator (Heidolph GmbH & Co. KG, Schwabach, Germany). All compounds were >95% pure by HPLC analysis.

COware gas two-chamber reactors from Sigma-Aldrich Chemie GmbH (Steinheim, Germany; catalog numbers: STW5 and STW6) were used for the generation of SO_2_F_2_ and HCl (except during the preparation of **2** and **8**).

#### 4.1.2 Nuclear Magnetic Resonance (NMR) Spectroscopy

NMR spectra were measured at ambient temperature in deuterochloroform (CDCl_3_), tetradeuteromethanol (CD_3_OD), or trideuteroacetonitrile (CD_3_CN) as indicated, using a Bruker Ascend 400 (^1^H: 400 MHz; ^13^C: 101 MHz; ^19^F: 376 MHz; Bruker Biospin GmbH, Rheinstetten, Germany). The measured chemical shifts are reported in δ [ppm] relative to residual peaks of nondeuterated solvents. Higher-order NMR spectra were approximately interpreted as first-order spectra if possible. The observed signal multiplicities are characterized as follows: s = singlet, d = doublet, t = triplet, q = quartet, m = multiplet, dd = doublet of doublets, dt = doublet of triplets, td = triplet of doublets, tt = triplet of triplets, and qd = quartet of doublets. Coupling constants *J* are reported in hertz (Hz). Doublets in ^13^C-spectra of sulfamoyl fluorides have very small coupling constants and can be difficult to detect in some cases, and therefore they are only provided where reliable determination was possible. If mixtures of two rotamers were observed, only the signals of the main rotamer are shown.

#### 4.1.3 Mass Spectrometry (MS)

Low resolution electrospray ionization mass spectrometry (LR-ESI-MS) was performed with an MSQ PlusTM mass spectrometer (Thermo Electron Corporation, San Jose, USA). High resolution electrospray ionization mass spectrometry (HR-ESI-MS) was performed with an LTQ XL Orbitrap (Thermo Fisher Scientific Inc., Bremen, Germany). High resolution electron ionization mass spectrometry (HR-EI-MS) and high resolution positive chemical ionization (HR-PCI-MS) were performed with an Exactive GC Orbitrap (Thermo Fisher Scientific Inc., Bremen, Germany).

#### 4.1.4 Column Chromatography

Manual column chromatography was performed with silica gel, 60 Å, 230–400 mesh particle size from VWR International GmbH (Darmstadt, Germany) or silica gel (w/0.1% Ca), 60 Å, 230–400 mesh particle size from Sigma-Aldrich GmbH (Steinheim, Germany). Automated column chromatography was performed on a Büchi Pure C-815 flash system (Büchi Labortechnik GmbH, Essen, Germany) using Reveleris C_18_ reversed phase cartridges (Büchi Labortechnik GmbH, Essen, Germany).

#### 4.1.5 Thin Layer Chromatography (TLC)

TLC was performed using aluminum sheets coated with silica gel 0.25 mm SIL G/UV 254 (Merck KGaA, Darmstadt, Germany). Chromatograms were inspected under UV light (λ = 254 nm) and/or stained with phosphomolybdic acid (4% in EtOH), ninhydrin (0.5% in 1-butanol) or potassium permanganate solution (0.75% KMnO_4_, 5% K_2_CO_3_, and 0.07% NaOH in H_2_O).

#### 4.1.6 Procedures

##### 4.1.6.1 *tert*-Butyl *N*_α_-(*tert*-butoxycarbonyl)-1-(fluorosulfonyl)-l-tryptophan (**1**)

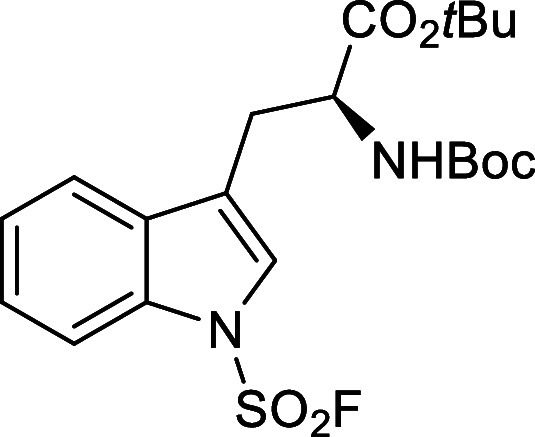



Desmethyl SuFEx-IT (144 mg, 458 μmol, 1.1 equiv) was added to a solution of Boc-Trp-O*t*Bu (**18**, 150 mg, 416 μmol, 1.0 equiv) and DBU (62 μL, 416 μmol, 1.0 equiv) in MeCN (1 mL) and the resulting mixture was stirred for 1 h. Three additional portions of desmethyl SuFEx-IT (1.1 equiv) and DBU (1.0 equiv) were added at hourly intervals until TLC indicated full conversion. The solvent was removed under reduced pressure, the residue was taken up in CH_2_Cl_2_ (10 mL) and the solution was filtered through a silica plug. The plug was washed with *n*-hexane:EtOAc (2:1) and the filtrate was concentrated under reduced pressure. The residue was purified by column chromatography (*n*-hexane:EtOAc/4:1, TLC: *R*_f_ = 0.61) to afford the title compound as a colorless solid (102 mg, 231 μmol, 55%). C_20_H_27_FN_2_O_6_S (442.50 g/mol). ^1^H NMR (400 MHz, CDCl_3_): δ 7.88 (d, *J* = 8.1 Hz, 1H), 7.66 (d, *J* = 7.6 Hz, 1H), 7.53–7.34 (m, 2H), 7.26 (s, 1H, overlap with residual solvent), 5.17 (d, *J* = 7.0 Hz, 1H), 4.56 (q, *J* = 6.1 Hz, 1H), 3.40–2.94 (m, 2H), 1.44 (s, 9H), 1.39 (s, 9H). ^13^C­{^1^H} NMR (101 MHz, CDCl_3_): δ 170.60, 155.23, 135.06, 131.08, 126.17, 124.82, 123.79, 120.52, 120.22, 113.75, 82.91, 80.19, 53.87, 28.43, 28.18, 28.04. ^19^F NMR (376 MHz, CDCl_3_): δ 54.00. HR-ESI-MS *m*/*z*: [M + Na]^+^ Calcd for C_20_H_27_FN_2_O_6_SNa 465.14661; Found: 465.14699.

##### 4.1.6.2 Methyl *N*_α_-(*tert*-butoxycarbonyl)-1-(fluorosulfonyl)-l-tryptophan (**2**)

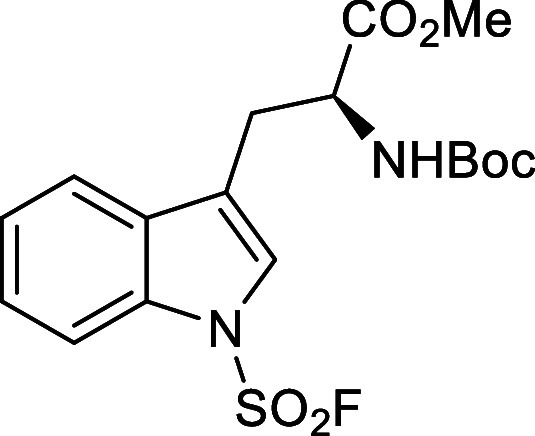



A two-chamber system was used for the reaction. Chamber A was loaded with SDI (3.98 g, 20.1 mmol, 16.0 equiv) and KF (2.92 g, 50.2 mmol, 40.0 equiv), while chamber B was loaded with a solution of Boc-Trp-OMe (400 mg, 1.26 mmol, 1.0 equiv) and DIPEA (641 μL, 3.77 mmol, 3.0 equiv) in MeCN (5 mL) and cooled to –40 °C (dry ice in MeCN). TFA (20 mL) was then cautiously added to chamber A and the resulting SO_2_F_2_ gas was bubbled (via a cannula) through the solution in chamber B. After a few minutes, the cannula was removed from the solution to avoid suck-back into chamber A. The reaction mixture in chamber B was stirred for 12 h, diluted with CH_2_Cl_2_ (40 mL), and washed with H_2_O (2 × 20 mL) and brine (20 mL). The organic phase was dried and concentrated under reduced pressure. The residue was purified by column chromatography (*n*-hexane:EtOAc/4:1, TLC: *R*_f_ = 0.39) to afford the title compound as a colorless solid (256 mg, 640 μmol, 51%). C_17_H_21_FN_2_O_6_S (400.42 g/mol). ^1^H NMR (400 MHz, CDCl_3_): δ 7.88 (d, *J* = 8.1 Hz, 1H), 7.62–7.56 (m, 1H), 7.47–7.41 (m, 1H), 7.41–7.36 (m, 1H), 7.24 (s, 1H), 5.16 (d, *J* = 7.2 Hz, 1H), 4.78–4.59 (m, 1H), 3.71 (s, 3H), 3.34–3.13 (m, 2H), 1.43 (s, 9H). ^13^C­{^1^H} NMR (101 MHz, CDCl_3_): δ 172.00, 155.16, 135.14, 130.82, 126.25, 124.88, 123.87, 120.15, 119.76, 113.85, 80.45, 53.38, 52. 68, 28.39, 28.08. ^19^F NMR (376 MHz, CDCl_3_): δ 54.06. HR-ESI-MS *m*/*z*: [M + Na]^+^ Calcd for C_17_H_21_FN_2_O_6_SNa 423.09966; Found: 423.09974.

##### 4.1.6.3 Indoline-1-sulfonyl Fluoride (**3**)

The title compound was prepared as described in a previous publication.^14^

##### 4.1.6.4 4-Phenylpiperidine-1-sulfonyl Fluoride (**4**)

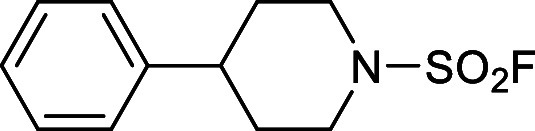



DBU (510 μL, 3.41 mmol, 2.2 equiv) was slowly added to a solution of 4-phenylpipridine (250 mg, 1.55 mmol, 1.0 equiv) and AISF (585 mg, 1.86 mmol, 1.2 equiv) in THF (10 mL) and the mixture was stirred for 30 min. The reaction mixture was diluted with EtOAc (40 mL) and washed with 0.5 m NaHSO_4_ (2 × 10 mL) and brine (10 mL). The organic phase was dried and concentrated under reduced pressure. The residue was purified by column chromatography (*n*-hexane:EtOAc/14:1, TLC: *R*_*f*_ = 0.28) to afford the title compound as a colorless solid (298 mg, 1.22 mmol, 79%). C_11_H_14_FNO_2_S (243.30 g/mol). ^1^H NMR (400 MHz, CDCl_3_): δ 7.42–7.30 (m, 2H), 7.30–7.18 (m, 3H), 4.16–3.97 (m, 2H), 3.13 (tt, *J* = 12.7, 3.1 Hz, 2H), 2.69 (tt, *J* = 12.0, 3.8 Hz, 1H), 2.08–1.80 (m, 4H). ^13^C­{^1^H} NMR (101 MHz, CDCl_3_): δ 144.26, 128.90, 127.04, 126.79, 48.03, 41.59, 32.09 (d, *J* = 0.9 Hz). ^19^F NMR (376 MHz, CDCl_3_): δ 40.43. HR-EI-MS *m*/*z*: [M]^•+^ Calcd for C_11_H_14_FNO_2_S 243.07238; Found: 243.07227.

##### 4.1.6.5 9*H*-Carbazole-9-sulfonyl Fluoride (**5**)

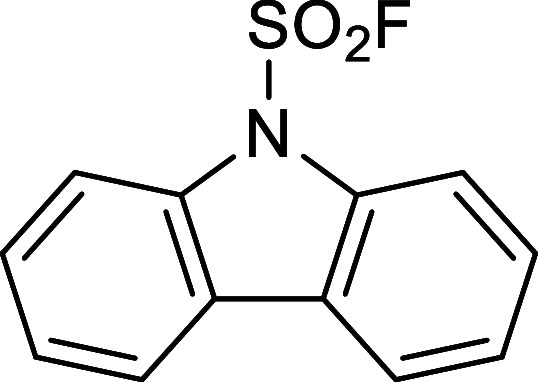



DBU (522 μL, 533 mg, 3.50 mmol, 2.2 equiv) was added to a solution of carbazole (266 mg, 1.59 mmol, 1.0 equiv) and AISF (600 mg, 1.91 mmol, 1.2 equiv)^24^ in THF (10 mL) and the reaction mixture was stirred for 2 h. Additional portions of AISF (0.4 g, 1.27 mmol, 0.8 equiv) and DBU (348 μL, 355 mg, 2.33 mmol, 1.47 equiv) were then added and stirring was continued for another 2 h. Thereafter, the reaction mixture was diluted with Et_2_O (40 mL) and washed with 0.5 m NaHSO_4_ (3 × 10 mL) and brine (2 × 10 mL). The organic phase was dried and concentrated under reduced pressure. The residue was purified by column chromatography (*n*-hexane:EtOAc/14:1, TLC: *R*_*f*_ = 0.28) to afford the title compound as a colorless solid (348 mg, 1.40 mmol, 88%). C_12_H_8_FNO_2_S (249.26 g/mol). ^1^H NMR (400 MHz, CDCl_3_): δ 8.07 (d, *J* = 8.3 Hz, 2H), 8.02 (dd, 2H), 7.55 (td, *J* = 8.4, 7.9, 1.4 Hz, 2H), 7.49 (td, *J* = 7.5, 0.9 Hz, 2H). ^13^C­{^1^H} NMR (101 MHz, CDCl_3_): δ 137.56 (d, *J* = 1.7 Hz), 128.19, 126.42, 125.41, 120.55, 114.95. ^19^F NMR (376 MHz, CDCl_3_): δ 52.32. HR-EI-MS *m*/*z*: [M]^•+^ Calcd for C_12_H_8_FNO_2_S 249.0254; Found: 249.0252.

##### 4.1.6.6 5-Methoxy-1*H*-indole-1-sulfonyl Fluoride (**6**)

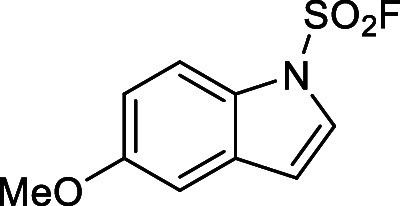



Desmethyl SuFEx-IT^14^ (587 mg, 1.87 mmol, 1.1 equiv) was added to a solution of 5-methoxy-1*H*-indole (250 mg, 1.70 mmol, 1.0 equiv) and DBU (254 μL, 1.70 mmol, 1.0 equiv) in MeCN (10 mL) and the resulting mixture was stirred for 2 h. Additional portions of desmethyl SuFEx-IT (2.67 g, 8.50 mmol, 5.0 equiv) and DBU (1.27 mL, 8.50 mmol, 1.0 equiv) were then added and the mixture was stirred for an additional 1 h. The solvent was removed under reduced pressure, the residue was taken up in CH_2_Cl_2_ (10 mL) and the solution was filtered through a silica plug. The plug was washed with CH_2_Cl_2_ and the filtrate was concentrated under reduced pressure. The crude product was purified by column chromatography (*n*-hexane:EtOAc/20:1, TLC: *R*_*f*_ = 0.25) to afford the title compound as a colorless solid (211 mg, 918 μmol, 54%). C_9_H_8_FNO_3_S (229.23 g/mol). ^1^H NMR (400 MHz, CDCl_3_): δ 7.78 (d, *J* = 9.0 Hz, 1H), 7.39 (d, *J* = 3.8 Hz, 1H), 7.08 (d, *J* = 2.5 Hz, 1H), 7.03 (dd, *J* = 9.0, 2.5 Hz, 1H), 6.73 (d, *J* = 3.8 Hz, 1H), 3.87 (s, 3H). ^13^C­{^1^H} NMR (101 MHz, CDCl_3_): δ 157.54, 131.57, 129.46 (d, *J* = 1.3 Hz), 126.90 (d, *J* = 2.0 Hz), 114.71, 114.46, 111.24 (d, *J* = 1.3 Hz), 104.56, 55.88. ^19^F NMR (376 MHz, CDCl_3_): δ 53.75. HR-EI-MS *m*/*z*: [M]^•+^ Calcd for C_9_H_8_FNO_3_S 229.0203; Found: 229.0201.

##### 4.1.6.7 5-Formyl-1*H*-indole-1-sulfonyl Fluoride (**7**)

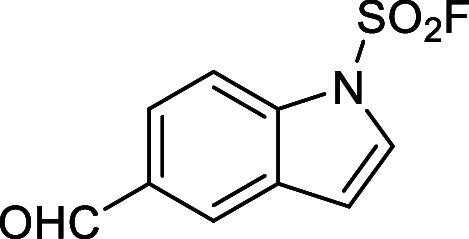



Desmethyl SuFEx-IT (2.71 g, 8.61 mmol, 5.0 equiv) was added to a solution of 5-formyl-1*H*-indole (250 mg, 1.72 mmol, 1.0 equiv) and DBU (1.29 mL, 8.61 mmol, 5.0 equiv) in MeCN (10 mL) and the resulting mixture was stirred for 2 h. The solvent was removed under reduced pressure, the residue was taken up in CH_2_Cl_2_ (10 mL) and the solution was filtered through a silica plug. The plug was washed with CH_2_Cl_2_ and the filtrate was concentrated under reduced pressure. The residue was purified by column chromatography (*n*-hexane:EtOAc/5:1, TLC: *R*_*f*_ = 0.29) to afford the title compound as a colorless solid (100 mg, 440 μmol, 26%). C_9_H_6_FNO_3_S (227.21 g/mol). ^1^H NMR (400 MHz, CDCl_3_): δ 10.11 (s, 1H), 8.19 (dd, *J* = 1.5, 0.7 Hz, 1H), 8.04 (d, *J* = 8.6 Hz, 1H), 7.98 (dd, *J* = 8.6, 1.5 Hz, 1H), 7.55 (d, *J* = 3.8 Hz, 1H), 6.94 (dd, *J* = 3.8, 0.7 Hz, 1H). ^13^C­{^1^H} NMR (101 MHz, CDCl_3_): δ 191.47, 138.15, 133.63, 130.79, 127.92 (d, *J* = 1.7 Hz), 126.89, 124.74, 114.26, 111.31 (d, *J* = 1.4 Hz). ^19^F NMR (376 MHz, CDCl_3_): δ 55.87. HR-EI-MS *m*/*z*: [M]^•+^ Calcd for C_9_H_7_FNO_3_S 227.0052; Found: 227.0043.

##### 4.1.6.8 1*H*-Indole-1-sulfonyl Fluoride (**8**)

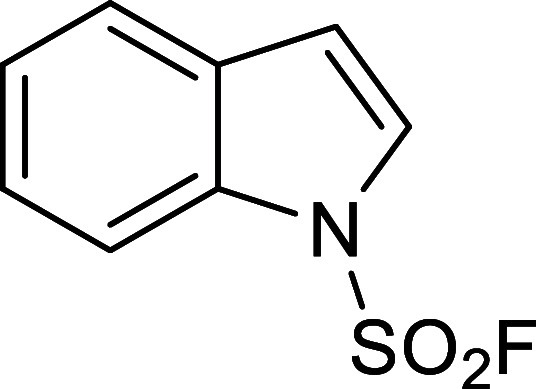



A two-chamber system with argon atmosphere was used for the reaction. Chamber A was loaded with SDI (3.38 g, 17.0 mmol, 8.0 equiv) and KF (2.47 g, 42.6 mmol, 20.0 equiv), while chamber B was loaded with a solution of indole (250 mg, 2.13 mmol, 1.0 equiv) and DIPEA (1.09 mL, 6.39 mmol, 3.0 equiv) in MeCN (5 mL) and cooled to −40 °C (dry ice in MeCN). TFA (15 mL) was then cautiously added to chamber A and the resulting gas was bubbled (via a cannula) through the solution in chamber B. After a few minutes, the cannula was removed from the solution to avoid suck-back into chamber A. The reaction mixture in chamber B was stirred for 12 h, diluted with CH_2_Cl_2_ (40 mL), and washed with H_2_O (2 × 20 mL) and brine (20 mL). The organic phase was dried and concentrated under reduced pressure. The residue was purified by column chromatography (*n*-hexane:EtOAc/8:1, TLC: *R*_*f*_ = 0.63) to afford the title compound as a colorless oil (307 mg, 1.54 mmol, 72%). C_8_H_6_FNO_2_S (199.20 g/mol). ^1^H NMR (400 MHz, CDCl_3_): δ 7.91 (d, *J* = 9.0 Hz, 1H), 7.64 (d, *J* = 7.9 Hz, 1H), 7.47–7.41 (m, 2H), 7.41–7.35 (m, 1H), 6.81 (d, *J* = 4.4 Hz, 1H). ^13^C­{^1^H} NMR (101 MHz, CDCl_3_): δ 134.97, 130.47, 126.21 (d, *J* = 2.0 Hz), 125.98, 124.94, 122.07, 113.68, 111.14 (d, *J* = 1.3 Hz). ^19^F NMR (376 MHz, CDCl_3_): δ 54.31. HR-EI-MS *m*/*z*: [M]^•+^ Calcd for C_8_H_6_FNO_2_S 199.0098; Found: 199.0095.

##### 4.1.6.9 Methyl­(phenyl)­sulfamoyl Fluoride (**9**)

The title compound was prepared as described in a previous publication.^14^

##### 4.1.6.10 (4-Methoxyphenyl)­(methyl)­sulfamoyl Fluoride (**10**)

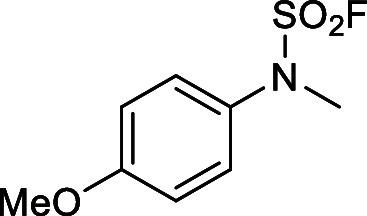



Desmethyl SuFEx-IT (630 mg, 2.00 mmol, 1.1 equiv) was added to a solution of 4-methoxy-*N*-methylaniline (250 mg, 1.82 mmol, 1.0 equiv) in MeCN (3 mL) and the resulting mixture was stirred for 1 h and concentrated under reduced pressure. The crude product was purified by dry-loaded column chromatography (*n*-hexane:EtOAc/10:1, TLC: *R*_*f*_ = 0.21) to afford the title compound as a yellow liquid (353 mg, 1.62 mmol, 88%). C_8_H_10_FNO_3_S (219.23 g/mol). ^1^H NMR (400 MHz, CDCl_3_): δ 7.39–7.28 (m, 2H), 7.00–6.86 (m, 2H), 3.82 (s, 3H), 3.40 (s, 3H). ^13^C­{^1^H} NMR (101 MHz, CDCl_3_): δ 159.99, 132.52 (d, *J* = 2.8 Hz), 128.23 (d, *J* = 2.1 Hz), 115.10, 55.68, 41.10 (d, *J* = 1.3 Hz). ^19^F NMR (376 MHz, CDCl_3_): δ 41.28. HR-EI-MS *m*/*z*: [M]^•+^ Calcd for C_8_H_10_FNO_3_S 219.0359; Found: 219.0358.

##### 4.1.6.11 [4-(Hydroxymethyl)­phenyl]­(methyl)­sulfamoyl Fluoride (**11**)

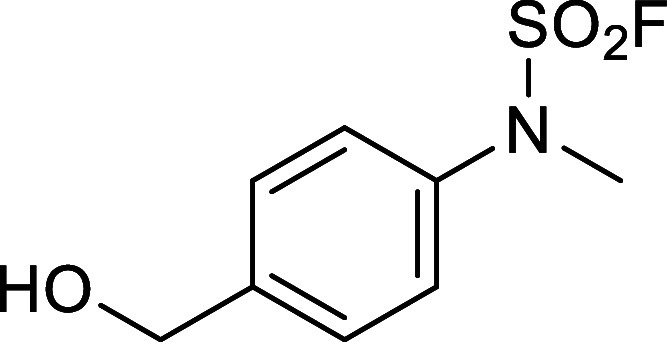



Desmethyl SuFEx-IT (3.15 g, 10.0 mmol, 1.1 equiv) was added to a solution of [4-(methylamino)­phenyl]­methanol (**28**, 1.25 g, 9.11 mmol, 1.0 equiv) in MeCN (10 mL) and the resulting mixture was stirred for 1 h and concentrated under reduced pressure. The crude product was purified by dry-loaded column chromatography (*n*-hexane:EtOAc/2:1, TLC: *R*_*f*_ = 0.15) to afford the title compound as a greenish oil (1.28 g, 5.84 mmol, 64%). C_8_H_10_FNO_3_S (219.23 g/mol). ^1^H NMR (400 MHz, CDCl_3_): δ 7.48–7.40 (m, 2H), 7.40–7.35 (m, 2H), 4.71 (s, 2H), 3.42 (s, 3H), 1.95 (s, 1H). ^13^C­{^1^H} NMR (101 MHz, CDCl_3_): δ 142.04, 139.15 (d, *J* = 2.9 Hz), 128.25, 126.86 (d, *J* = 1.9 Hz), 64.51, 40.81 (d, *J* = 1.2 Hz). ^19^F NMR (376 MHz, CDCl_3_): δ 42.31. HR-ESI-MS *m*/*z*: [M + Na]^+^ Calcd for C_8_H_10_FNO_3_SNa 242.02576; Found: 242.02586.

##### 4.1.6.12 (4-Formylphenyl)­(methyl)­sulfamoyl Fluoride (**12**)

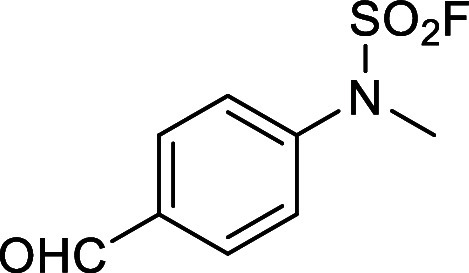



Desmethyl SuFEx-IT (640 mg, 2.04 mmol, 1.1 equiv) was added to a solution of 4-(methylamino)­benzaldehyde (250 mg, 1.85 mmol, 1.0 equiv) and DBU (276 μL, 1.85 mmol, 1.0 equiv) in MeCN (2 mL) and the resulting mixture was stirred for 2 h. Additional portions of desmethyl SuFEx-IT (1.74 g, 5.55 mmol, 3.0 equiv) and DBU (555 μL, 3.70 mmol, 2.0 equiv) were then added and the mixture was stirred for another 1 h. The solvent was removed under reduced pressure, the residue was taken up in CH_2_Cl_2_ (5 mL) and the solution was filtered through a silica plug. The plug was washed with CH_2_Cl_2_ and the filtrate was concentrated under reduced pressure. The resulting crude product was purified by column chromatography (*n-*hexane:EtOAc/2:1, TLC: *R*_*f*_ = 0.38) to afford the title compound as an orange solid (38 mg, 175 μmol, 9%). C_8_H_8_FNO_3_S (217.21 g/mol). ^1^H NMR (400 MHz, CDCl_3_): δ 10.04 (s, 1H), 8.14–7.83 (m, 2H), 7.67–7.44 (m, 2H), 3.50 (s, 3H). ^13^C­{^1^H} NMR (101 MHz, CDCl_3_): δ 190.79, 144.95 (d, *J* = 2.4 Hz), 136.05, 131.17, 126.74 (d, *J* = 2.0 Hz), 40.39 (d, *J* = 1.4 Hz). ^19^F NMR (376 MHz, CDCl_3_): δ 44.16. HR-EI-MS *m*/*z*: [M]^•+^ Calcd for C_8_H_8_FNO_3_S 217.0197; Found: 217.0203.

##### 4.1.6.13 3,4-Dihydroquinoline-1­(2*H*)-sulfonyl Fluoride (**13**)

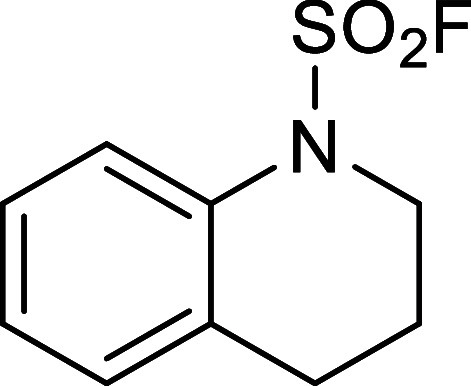



Desmethyl SuFEx-IT (389 mg, 1.24 mmol, 1.1 equiv) was added to a solution of 1,2,3,4-tetrahydroquinoline (150 mg, 1.13 mmol, 1.0 equiv) in MeCN (3 mL), and the resulting mixture was stirred for 1 h before it was concentrated under reduced pressure. The crude product was purified by dry-loaded column chromatography (*n*-hexane:EtOAc/20:1, TLC: *R*_*f*_ = 0.29) to afford the title compound as a colorless solid (207 mg, 962 μmol, 85%). C_9_H_10_FNO_3_S (215.24 g/mol). ^1^H NMR (400 MHz, CDCl_3_): δ 7.52 (d, *J* = 8.0 Hz, 1H), 7.26–7.09 (m, 3H), 3.97–3.81 (m, 2H), 2.91 (t, *J* = 6.8 Hz, 2H), 2.15–2.09 (m, 2H). ^13^C­{^1^H} NMR (101 MHz, CDCl_3_): δ ^13^C NMR (101 MHz, CDCl_3_) δ 135.30 (d, *J* = 2.6 Hz), 130.72, 129.67, 126.95, 126.52, 124.21 (d, *J* = 2.0 Hz), 48.60 (d, *J* = 2.2 Hz), 26.23, 22.24. ^19^F NMR (376 MHz, CDCl_3_): δ 49.47. HR-EI-MS *m*/*z*: [M]^•+^ Calcd for C_9_H_10_FNO_2_S 215.0411; Found: 215.0406.

##### 4.1.6.14 *tert*-Butyl (*S,S*)- and (*S,R*)-2-[(*tert*-butoxycarbonyl)­amino]-3-[1-(fluorosulfonyl)­indolin-3-yl]­propanoate [(*S,R*)-**14** and (*S,S*)-**14**]

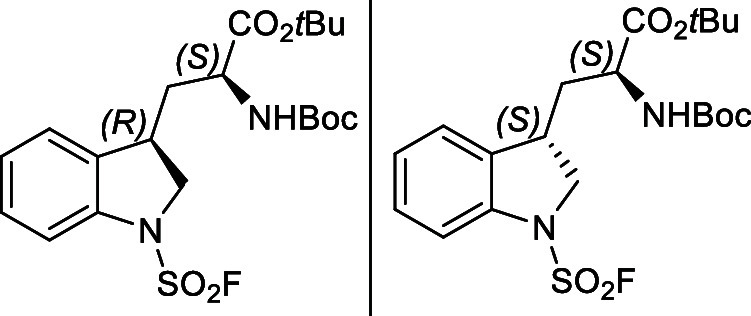



Desmethyl SuFEx-IT (292 mg, 929 μmol, 1.1 equiv) was added to a solution of (*S*,*S*)-**19** (324 mg, 893 μmol, 1.0 equiv) in MeCN (5 mL). The reaction mixture was stirred for 30 min and then concentrated under reduced pressure. The residue was taken up in EtOAc (20 mL) and the resulting solution was washed with H_2_O (20 mL). The organic phase was dried and concentrated under reduced pressure. The residue was purified by column chromatography (*n*-hexane:EtOAc/8:1, TLC: (*S,S*)-**14**: *R*_f_ = 0.17) to afford (*S*,*S*)-**14** as a colorless solid (275 mg, 618 μmol, 74%). Using the same procedure, (*S*,*R*)-**14** was prepared from (*S*,*R*)-**19** as a colorless solid in 74% yield (*n*-hexane:EtOAc/8:1, TLC: (*S,R*)-**14**: *R*_f_ = 0.21). C_20_H_29_FN_2_O_6_S (444.52 g/mol). (*S*,*R*)-**14**: ^1^H NMR (400 MHz, CDCl_3_): δ 7.46 (d, *J* = 8.1 Hz, 1H), 7.27 (t, *J* = 7.6 Hz, 1H), 7.22 (d, *J* = 7.4 Hz, 1H), 7.14 (t, *J* = 7.4 Hz, 1H), 5.22 (d, *J* = 7.2 Hz, 1H), 4.49–4.05 (m, 2H), 4.05–3.84 (m, 1H), 3.62–3.41 (m, 1H), 2.19–2.03 (m, 1H), 2.02–1.93 (m, 1H), 1.48 (s, 9H), 1.46 (s, 9H). ^13^C­{^1^H} NMR (101 MHz, CDCl_3_): δ 171.28, 155.75, 139.77 (d, *J* = 2.7 Hz), 134.33, 128.83, 125.39, 124.73, 114.93, 83.01, 80.38, 57.28, 52.46, 38.99, 37.33, 28.42, 28.13. ^19^F NMR (376 MHz, CDCl_3_): δ 39.61. HR-ESI-MS *m*/*z*: [M + Na]^+^ Calcd for C_20_H_29_FN_2_O_6_SNa 467.16226; Found: 467.16223. (*S*,*S*)-**14**: ^1^H NMR (400 MHz, CDCl_3_): δ 7.45 (d, *J* = 8.1 Hz, 1H), 7.38–7.23 (m, 2H), 7.16 (td, *J* = 7.5, 0.9 Hz, 1H), 5.17 (d, *J* = 7.5 Hz, 1H), 4.44–4.04 (m, 2H), 3.95–3.84 (m, 1H), 3.69–3.45 (m, 1H), 2.47–2.19 (m, 1H), 1.97–1.79 (m, 1H), 1.48 (s, 9H), 1.47 (s, 9H). ^13^C­{^1^H} NMR (101 MHz, CDCl_3_): δ 171.31, 155.49, 139.52 (d, *J* = 2.6 Hz), 134.26, 128.84, 125.51, 125.09, 114.83, 83.08, 80.45, 57.71, 52.93, 38.46, 37.56, 28.45, 28.14. ^19^F NMR (376 MHz, CDCl_3_): δ 39.38. HR-ESI-MS *m*/*z*: [M + Na]^+^ Calcd for C_20_H_29_FN_2_O_6_SNa 467.16226; Found: 467.16232.

##### 4.1.6.15 Methyl *N*_α_-(*tert*-butoxycarbonyl)-*N*_*im*_-(fluorosulfonyl)-l-histidinate (**15)**

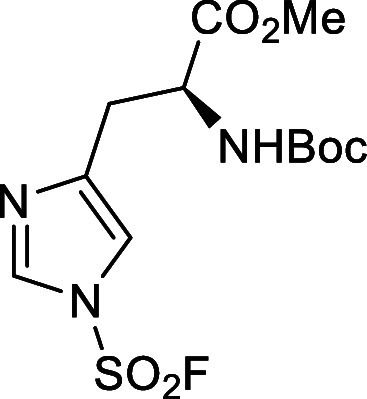



DBU (522 μL, 532 mg, 3.50 mmol, 2.2 equiv) was added to a solution of Boc-His-OMe (428 mg, 1.59 mmol, 1.0 equiv) and AISF (600 mg, 1.91 mmol, 1.2 equiv) in THF (10 mL) and the reaction mixture was stirred for 2 h. The reaction mixture was then diluted with Et_2_O (40 mL) and washed with 0.5 m NaHSO_4_ (3 × 10 mL) and brine (2 × 10 mL). The organic phase was dried and concentrated under reduced pressure. The residue was purified by column chromatography (*n*-hexane:EtOAc/1:1, TLC: *R*_*f*_ = 0.75) and low temperature crystallization (−25 °C) from Et_2_O/pentane to afford the title compound as a colorless solid (316 mg, 899 μmol, 57%). C_12_H_18_FN_3_O_6_S (351.35 g/mol). ^1^H NMR (400 MHz, CDCl_3_): δ 7.96 (s, 1H), 7.20 (s, 1H), 5.45 (d, *J* = 7.5 Hz, 1H), 4.74–4.39 (m, 1H), 3.75 (s, 3H), 3.25–2.93 (m, 2H), 1.43 (s, 9H). ^13^C­{^1^H} NMR (101 MHz, CDCl_3_): δ 171.97, 155.41, 141.58, 137.00, 115.50, 80.32, 52.70, 52.67, 30.68, 28.40. ^19^F NMR (376 MHz, CDCl_3_): δ 59.36. HR-EI-MS *m*/*z*: [M-Boc]^•+^ Calcd for C_7_H_9_FN_3_O_4_S 250.0298; Found: 250.0290.

Note that phase inversion of the C-2 signal in the APT spectrum of imidazole derivatives has been described in the literature as a “regular feature” of these compounds (for details see Ebner et al.^31^).

##### 4.1.6.16 1,2,3,4-Tetrahydroisoquinoline-2-sulfonyl Fluoride (**16**)

The title compound was prepared as described in a previous publication.^14^

##### 4.1.6.17 Phenyliminodisulfonyl Difluoride (**17**)

The title compound was prepared as described in a previous publication.^14^

##### 4.1.6.18 *tert*-Butyl (*tert*-butoxycarbonyl)-l-tryptophan (**18**)^32^

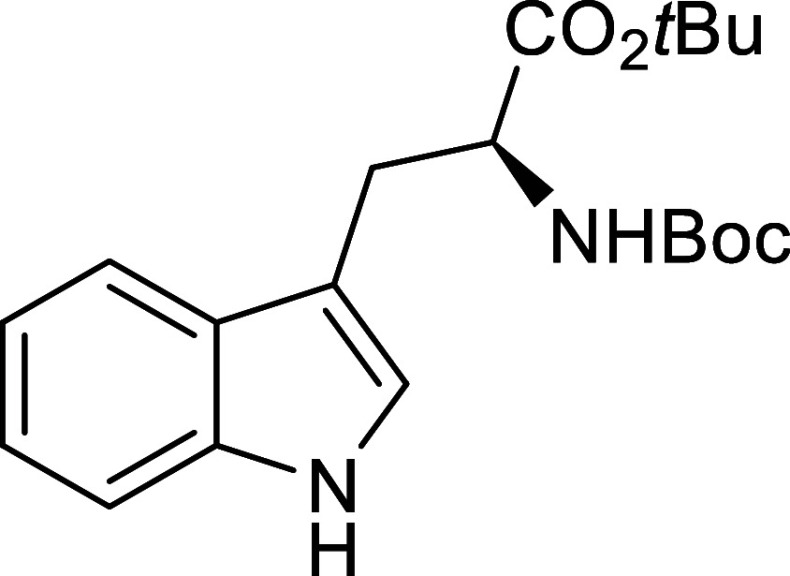



*t*BuBr (16.7 mL, 147.9 mmol, 10.0 equiv) was added dropwise to a suspension of K_2_CO_3_ (10.2 g, 73.9 mmol, 5.0 equiv) in a solution of Boc-Trp-OH (4.51 g, 14.8 mmol, 1.0 equiv) and BnEt_3_NCl (3.37 g, 14.8 mmol, 1.0 equiv) in DMA (20 mL), and the mixture was heated for 2.5 h at 55 °C. The reaction mixture was diluted with H_2_O (40 mL) and extracted with EtOAc (3 × 25 mL). The organic phase was washed with H_2_O (10 × 25 mL) and brine (2 × 25 mL), dried, and concentrated under reduced pressure. The residue was recrystallized from Et_2_O/*n*-hexane to afford the title compound as a colorless solid (2.66 g, 7.39 mmol, 50%). C_20_H_28_N_2_O_4_ (360.45 g/mol). ^1^H NMR (400 MHz, CDCl_3_): δ 8.32 (s, 1H), 7.75 (d, *J* = 7.9 Hz, 1H), 7.47 (d, *J* = 8.0 Hz, 1H), 7.35– 7.29 (m, 1H), 7.28–7.21 (m, 1H), 7.17–7.09 (m, 1H), 5.21 (d, *J* = 7.9 Hz, 1H), 4.68 (q, *J* = 5.8 Hz, 1H), 3.39 (tt, *J* = 15.0, 7.4 Hz, 2H), 1.56 (s, 9H), 1.51 (s, 9H). ^13^C­{^1^H} NMR (101 MHz, CDCl_3_): δ 171.59, 155.42, 136.18, 128.06, 122.78, 122.17, 119.56, 119.21, 111.17, 110.76, 81.91, 79.70, 54.84, 28.47, 28.13, 28.07. HR-ESI-MS *m*/*z*: [M + H]^+^ Calcd for C_20_H_29_N_2_O_4_ 361.21218; Found: 361.21252.

##### 4.1.6.19 *tert*-Butyl (*S,S*)- and (*S,R*)-2-[(*tert*-butoxycarbonyl)­amino]-3-(indolin-3-yl)­propanoate [(*S,R*)-**19** and (*S,S*)-**19**]

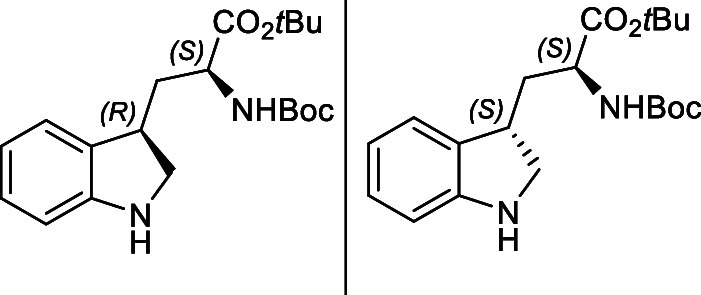



NaBH_3_CN (1.57 g, 25.3 mmol, 3.0 equiv) was added to an ice-cooled solution of Boc-Trp-O*t*Bu (**18**, 3.04 g, 8.43 mmol, 1.0 equiv) in AcOH (40 mL). Additional portions of NaBH_3_CN (8 × 1.05 g, 16.6 mmol, 2.0 equiv) were added at hourly intervals until HPLC indicated complete consumption of the starting material. The reaction mixture was then diluted with H_2_O (100 mL) and the pH was adjusted to 10 using 1 m NaOH. The resulting solution was extracted with CH_2_Cl_2_ (2 × 200 mL), the organic phase was washed with brine (150 mL), dried and concentrated under reduced pressure. The residue was purified and the diastereomers separated by three consecutive column chromatography runs (*n*-hexane:EtOAc/3:1, TLC: (*S,R*)-**19**: *R*_f_ = 0.22/(*S,S*)-**19**: *R*_f_ = 0.17). The diastereomers were obtained as colorless oils [(*S,R*)-**19**: 729 mg, 2.01 mmol, 46%; (*S,S*)-**19** *:* 431 mg, 1.19 mmol, 28%].* C_20_H_30_N_2_O_4_ (362.47 g/mol). (*S,R*)-**19** *:* ^1^H NMR (400 MHz, CDCl_3_): δ 7.07 (d, *J* = 7.3 Hz, 1H), 7.04 (t, *J* = 7.7 Hz, 1H), 6.72 (t, *J* = 7.4 Hz, 1H), 6.64 (d, *J* = 7.7 Hz, 1H), 5.10 (d, *J* = 8.1 Hz, 1H), 4.29 (td, *J* = 9.0, 4.2 Hz, 1H), 3.95–3.46 (m, 2H), 3.44–3.19 (m, 2H), 2.18–1.91 (m, 2H), 1.46 (s, 9H), 1.45 (s, 9H). ^13^C­{^1^H} NMR (101 MHz, CDCl_3_): δ 172.11, 155.66, 151.46, 132.16, 127.92, 123.83, 118.78, 109.72, 82.23, 79.92, 53.29, 52.75, 38.82, 38.08, 28.45, 28.14. HR-ESI-MS *m*/*z*: [M + H]^+^ Calcd for C_20_H_31_N_2_O_4_ 363.22783; Found: 363.22767. (*S,S*)-**19** *:* ^1^H NMR (400 MHz, CDCl_3_): δ 7.14 (d, *J* = 7.1 Hz, 1H), 7.04 (t, *J* = 7.6 Hz, 1H), 6.73 (td, *J* = 7.4, 0.9 Hz, 1H), 6.65 (d, *J* = 7.7 Hz, 1H), 5.09 (d, *J* = 7.9 Hz, 1H), 4.44–4.02 (m, 1H), 3.75 (t, *J* = 8.6 Hz, 1H), 3.45–3.33 (m, 1H), 3.28 (t, *J* = 8.0 Hz, 1H), 2.38–2.13 (m, 1H), 1.93–1.78 (m, 1H), 1.47 (s, 9H), 1.45 (s, 9H). ^13^C­{^1^H} NMR (101 MHz, CDCl_3_): δ 172.11, 155.35, 151.26, 132.11, 127.90, 124.09, 118.90, 109.74, 82.28, 79.94, 53.76, 53.11, 39.12, 37.97, 28.47, 28.16. HR-ESI-MS *m*/*z*: [M + H]^+^ Calcd for C_20_H_31_N_2_O_4_ 363.22783; Found: 363.22814.

* The stereoconfiguration of the diastereomers was determined by comparing the corresponding ^1^H NMR spectra with literature data for enantiomers of Boc-Dht-OMe.^33^

##### 4.1.6.20 1-(Fluorosulfonyl)-l-tryptophan Hydrochloride (**20**·HCl)

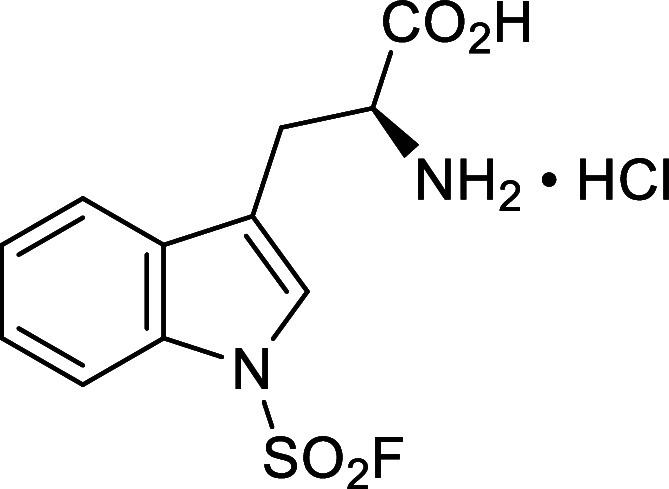



Boc-Trp­(SO_2_F)-O*t*Bu (65 mg, 147 μmol, 1.0 equiv) was added to 4 m HCl in dioxane (500 μL) and the resulting mixture was stirred at 60 °C until TLC indicated full conversion (2 h). The reaction mixture was concentrated under reduced pressure, the residue was washed with Et_2_O/*n*-pentane (1:1, 2 × 5 mL), and the resulting solid was dried under reduced pressure to afford the title compound as a colorless solid (46 mg, 143 μmol, 97%). C_11_H_12_ClFN_2_O_4_S (322.74 g/mol). ^1^H NMR (400 MHz, CD_3_OD): δ 7.89 (d, *J* = 7.8 Hz, 1H), 7.81 (d, *J* = 7.4 Hz, 1H), 7.62 (s, 1H), 7.57–7.42 (m, 2H), 4.39 (t, *J* = 6.4 Hz, 1H), 3.57–3.34 (m, 2H). ^13^C­{^1^H} NMR (101 MHz, CD_3_OD): δ 169.66, 135.17, 129.97, 126.57, 126.16, 124.82, 119.84, 117.72, 113.17, 51.98, 25.37. ^19^F NMR (376 MHz, CD_3_OD): δ 51.68. HR-ESI-MS *m*/*z*: [M + H]^+^ Calcd for C_11_H_12_FN_2_O_4_S 287.04963; Found: 287.04977.

##### 4.1.6.21 (*S,R*)-2-Amino-3-[1-(fluorosulfonyl)­indolin-3-yl]­propanoic Acid Hydrochloride [(*S,R*)**-21**·HCl]

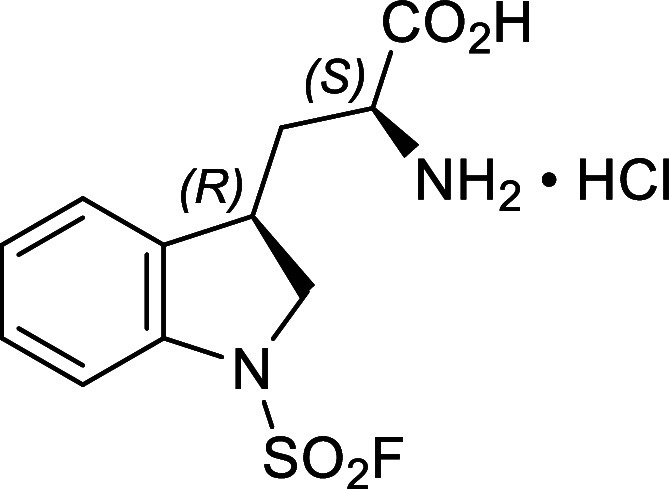



(*S,R*)**-14** (50 mg, 112 μmol, 1.0 equiv) was dissolved in 4 m HCl in dioxane (500 μL) and the resulting mixture was stirred at 60 °C in a closed reaction vessel until TLC indicated full conversion (3 h). The reaction mixture was concentrated under reduced pressure, the residue was washed with Et_2_O (2 × 2 mL) and the resulting solid was dried under reduced pressure to afford the title compound as a colorless solid (33 mg, 102 μmol, 91%). C_11_H_14_ClFN_2_O_4_S (324.75 g/mol). ^1^H NMR (400 MHz, CD_3_OD): δ 7.45 (dd, *J* = 7.5, 5.2 Hz, 2H), 7.35 (t, *J* = 8.1 Hz, 1H), 7.29–7.18 (m, 1H), 4.47–4.30 (m, 1H), 4.12 (t, *J* = 7.2 Hz, 1H), 4.00 (dd, *J* = 12.4, 4.7 Hz, 1H), 3.82–3.68 (m, 1H), 2.35–2.15 (m, 2H). ^13^C­{^1^H} NMR (101 MHz, CD_3_OD): δ 171.37, 140.75 (d, *J* = 2.6 Hz), 134.96, 130.13, 126.74, 126.53, 115.74, 58.36, 52.17, 38.26, 37.09. ^19^F NMR (376 MHz, CD_3_OD): δ 36.65. HR-ESI-MS *m*/*z*: [M + H]^+^ Calcd for C_11_H_14_FN_2_O_4_S 289.06528; Found: 289.06535.

##### 4.1.6.22 (*S,S*)-2-Amino-3-[(1-(fluorosulfonyl)­indolin-3-yl)]­propanoic Acid Hydrochloride [(*S,S*)-**21**·HCl]

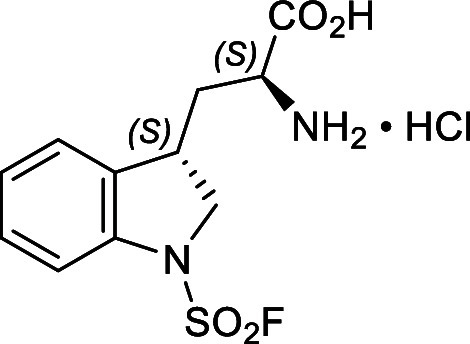



Acetyl chloride (2.8 mL, 40 mmol) was added slowly to an ice-cooled solution of (*S,S*)-**14** (47 mg, 106 μmol, 1.0 equiv) in 1,2-dichloroethane (5.5 mL) and MeOH (1.6 mL, 40 mmol). The flask was closed with a screw cap and the reaction mixture was stirred until TLC indicated full conversion (2.5 h). The reaction mixture was concentrated under reduced pressure to afford the pure title compound as a colorless solid (31 mg, 96 μmol, 91%). C_11_H_14_ClFN_2_O_4_S (324.75 g/mol). ^1^H NMR (400 MHz, CD_3_OD): δ 7.48–7.38 (m, 2H), 7.39–7.30 (m, 1H), 7.23 (td, *J* = 7.5, 1.0 Hz, 1H), 4.49–4.29 (m, 1H), 4.26–4.10 (m, 1H), 4.10–3.96 (m, 1H), 3.93–3.72 (m, 1H), 2.57–2.38 (m, 1H), 2.19–2.04 (m, 1H). ^13^C­{^1^H} NMR (101 MHz, CD_3_OD): δ 171.31, 140.63 (d, *J* = 2.2 Hz)., 135.09, 130.02, 126.79, 126.04, 115.62, 57.78, 51.99, 37.97, 36.46. ^19^F NMR (376 MHz, CD_3_OD): δ 36.54. HR-ESI-MS *m*/*z*: [M + H]^+^ Calcd for C_11_H_14_FN_2_O_4_S 289.06528; Found: 289.06533.

##### 4.1.6.23 Methyl (*S*)-2-[(*tert*-butoxycarbonyl)­amino]-3-(1*H*-indol-5-yl)­propanoate (**22**)^22^

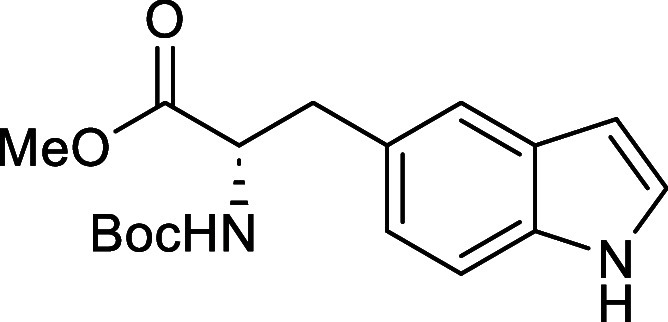



Anhydrous DMF (10 mL) was added to a flame-dried flask containing 5-bromoindole (1.63 g, 8.29 mmol, 1.0 equiv), *N*-(*tert*-butoxycarbonyl)-3-iodo-l-alanine methyl ester (3.00 g, 9.11 mmol, 1.1 equiv), Pd­(amphos)_2_Cl_2_ (293 mg, 415 μmol, 0.05 eq,) and zinc powder (813 mg, 12.43 mmol, 1.5 equiv), and the resulting suspension was vigorously stirred for 48 h. The mixture was filtered over a plug of silica and the plug was washed with EtOAc. The resulting clear filtrate was concentrated under reduced pressure. The residue was first purified by column chromatography (*n*-hexane:EtOAc/3.5:1, TLC: *R*_*f*_ = 0.20) and then by recrystallization from Et_2_O at −18 °C to afford the title compound as a colorless solid (1.19 g, 3.74 mmol, 45%). C_17_H_22_N_2_O_4_ (318.37 g/mol). ^1^H NMR* (400 MHz, CDCl_3_): δ 8.25 (s, 1H), 7.41 (s, 1H), 7.33 (d, *J* = 8.3 Hz, 1H), 7.24–7.19 (m, 1H), 6.97 (dd, *J* = 8.3, 1.6 Hz, 1H), 6.58–6.47 (m, 1H), 5.00 (d, *J* = 7.7 Hz, 1H), 4.62 (q, *J* = 6.0 Hz, 1H), 3.74 (s, 3H), 3.20 (t, *J* = 7.7 Hz, 2H), 1.44 (s, 9H). HR-ESI-MS *m*/*z*: [M + Na]^+^ Calcd for C_17_H_22_N_2_O_4_Na 341.14718; Found: 341.14778.

^*1^H NMR is in accordance with the literature.^22^

##### 4.1.6.24 (*S*)-2-[(*tert*-Butoxycarbonyl)­amino]-3-(1*H*-indol-5-yl)­propanoic Acid (**23**)

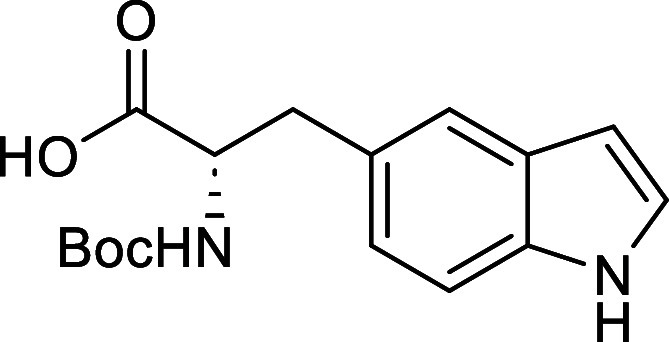



NaOH (139 mg, 3.49 mmol, 1.5 equiv) was added to a solution of **22** (740 mg, 2.32 mmol, 1.0 equiv) in MeOH/H_2_O (9 mL, 7:1) and the reaction mixture was stirred for 16 h. The MeOH was removed under reduced pressure, the residual aqueous solution acidified to pH 3 with 0.1 m HCl and extracted with EtOAc (6 × 30 mL). The organic phase was dried and concentrated to afford the title compound as a yellow foam (698 mg, 2.29 mmol, 98%). C_16_H_20_N_2_O_4_S (304.35 g/mol). ^1^H NMR (400 MHz, CDCl_3_): δ 8.29 (s, 1H), 7.43 (s, 1H), 7.37–7.24 (m, 1H), 7.17 (s, 1H), 7.00 (d, *J* = 8.1 Hz, 1H), 6.49 (s, 1H), 5.00 (d, *J* = 6.8 Hz, 1H), 4.74–4.26 (m, 1H), 3.47–2.78 (m, 2H), 1.41 (s, 9H). ^13^C­{^1^H} NMR (101 MHz, CDCl_3_): δ 176.86, 155.82, 135.20, 128.27, 126.97, 124.79, 123.45, 121.44, 111.45, 102.51, 80.38, 54.97, 37.89, 28.43. HR-ESI-MS *m*/*z*: [M + Na]^+^ Calcd for C_16_H_20_N_2_O_4_SNa 327.13153; Found: 327.13183.

##### 4.1.6.25 Dicyclopropylmethyl (*S*)-2-[(*ter*t-butoxycarbonyl)­amino]-3-(1*H*-indol-5-yl)­propanoate (**24**)

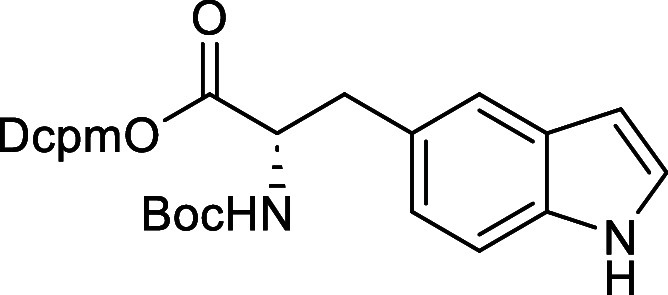



Dicyclopropylmethanol (DcpmOH; 174 μL, 1.48 mmol, 1.0 equiv) was added dropwise to a solution of **23** (450 mg, 1.48 mmol, 1.0 equiv), 1-ethyl-3-(3-(dimethylamino)­propyl)­carbodiimide hydrochloride (EDC·HCl; 368 mg, 1.92 mmol, 1.3 equiv), and 4-dimethylaminopyridine (DMAP; 36 mg, 296 μmol, 0.2 equiv) in CH_2_Cl_2_ (5 mL). The reaction mixture was stirred for 16 h at 23 °C and filtered over a plug of deactivated silica* that was washed with *n*-hexane/EtOAc (1:1) thereafter. The filtrate was concentrated under reduced pressure and the crude product was purified by column chromatography on deactivated silica* (*n*-hexane:EtOAc/3:1, TLC: *R*_*f*_ = 0.28) to afford the title compound as a colorless foam (397 mg, 995 μmol, 67%). C_23_H_30_N_2_O_4_ (398.50 g/mol). ^1^H NMR (400 MHz, CDCl_3_): δ 8.21 (s, 1H), 7.45 (s, 1H), 7.29 (d, *J* = 8.3 Hz, 1H), 7.21–7.16 (m, 1H), 7.03 (d, *J* = 8.3 Hz, 1H), 6.51–6.41 (m, 1H), 4.98 (d, *J* = 8.0 Hz, 1H), 4.60 (q, *J* = 5.6 Hz, 1H), 3.92 (t, *J* = 8.3 Hz, 1H), 3.23 (d, *J* = 5.6 Hz, 2H), 1.42 (s, 9H), 1.13 – 1.02 (m, 2H), 0.60–0.29 (m, 8H). ^13^C­{^1^H} NMR (101 MHz, CDCl_3_): δ 172.04, 155.38, 135.13, 128.21, 127.30, 124.60, 123.81, 121.63, 111.12, 102.50, 83.49, 79.72, 55.14, 38.22, 28.47, 14.82, 14.81, 3.27, 3.07, 3.03, 2.81.** HR-ESI-MS *m*/*z*: [M + Na]^+^ Calcd for C_23_H_30_N_2_O_4_Na 421.20977; Found: 421.21005.

*Silica was deactivated by flushing with one column volume of the solvent system containing 5% Et_3_N and three column volumes of the solvent system only.

**CH-and CH_2_-groups of the dicyclopropylmethyl group are diastereotopic, resulting in individual signals in ^13^C NMR.^34^

##### 4.1.6.26 Dicyclopropylmethyl (*S*)-2-[(*tert*-butoxycarbonyl)­amino]-3-[1-(fluorosulfonyl)-1*H*-indol-5-yl]­propanoate (**25**)

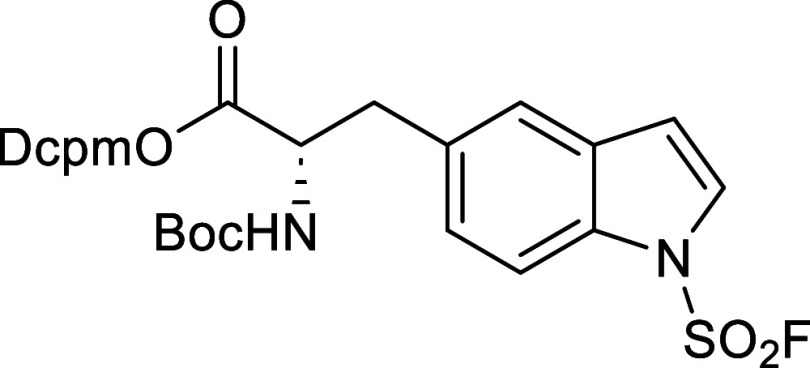



DBU (303 μL, 311 mg, 2.04 mmol, 2.2 equiv) was added to a solution of **24** (367 mg, 921 μmol, 1.0 equiv) and AISF (347 mg, 1.11 mmol, 1.2 equiv) in CH_2_Cl_2_ (10 mL) and the mixture was stirred for 30 min at 23 °C. AISF (5 × 145 mg, 461 μmol, 0.5 equiv) and another portion of DBU (137 μL, 140 mg, 921 μmol, 1.0 equiv) were then added every 30 min until no further conversion was observed via HPLC. The reaction mixture was washed with 0.1 m phosphate buffer (pH 7.6, 5 × 10 mL), the organic phase was dried and filtered over aluminum oxide (90 active, neutral). The filtrate was concentrated under reduced pressure and the crude product was purified by column chromatography on deactivated silica* (*n*-hexane:EtOAc/5:1, TLC: *R*_*f*_ = 0.31) to afford the title compound as a colorless solid (146 mg, 304 μmol, 33%). C_23_H_29_FN_2_O_6_S (480.55 g/mol). ^1^H NMR (400 MHz, CDCl_3_): δ 7.80 (d, *J* = 8.5 Hz, 1H), 7.50–7.44 (m, 1H), 7.40 (d, *J* = 3.8 Hz, 1H), 7.28 (dd, *J* = 8.5, 1.5 Hz, 1H), 6.73 (d, *J* = 3.8 Hz, 1H), 5.02 (d, *J* = 7.9 Hz, 1H), 4.62 (q, *J* = 6.0 Hz, 1H), 3.88 (t, *J* = 8.5 Hz, 1H), 3.25 (qd, *J* = 13.8, 5.7 Hz, 2H), 1.42 (s, 9H), 1.15–1.02 (m, 2H), 0.64–0.52 (m, 2H), 0.52–0.40 (m, 2H), 0.39–0.26 (m, 4H). ^13^C­{^1^H} NMR (101 MHz, CDCl_3_): δ 171.52, 155.18, 134.06, 133.21, 130.68, 127.66, 126.51, 122.90, 113.49, 110.99, 84.15, 80.03, 54.85, 38.17, 28.45, 14.83, 14.79, 3.33, 3.15, 3.13, 2.87.^** 19^F NMR (376 MHz, CDCl_3_): δ 54.33. HR-ESI-MS *m*/*z*: [M + Na]^+^ Calcd for C_23_H_29_FN_2_O_6_SNa 503.16226; Found: 503.16235.

*Silica was deactivated by flushing with one column volume of the solvent system containing 5% Et_3_N and three column volumes of the solvent system only.

**CH-and CH_2_-groups of the dicyclopropylmethyl group are diastereotopic, resulting in individual signals in ^13^C NMR.^34^

##### 4.1.6.27 (*S*)-2-Amino-3-[1-(fluorosulfonyl)-1*H*-indol-5-yl]­propanoic Acid Hydrochloride (**26**·HCl)

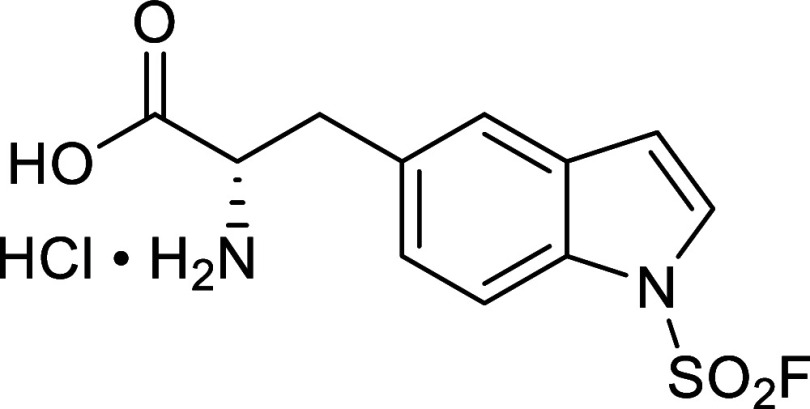



For the deprotection of **25**, a solvent-free method described by Verschueren et al. was used.^30^ To this end, NaCl (61 mg, 1.04 mmol, 10.0 equiv) was placed in chamber one of a two-chamber reactor and **25** (50 mg, 104 μmol, 1.0 equiv) was placed in chamber two. Concentrated H_2_SO_4_ (0.5 mL) was added to chamber one via a syringe to generate HCl and the setup was left for reaction for 16 h. The title compound was collected from chamber two as a colorless solid (31 mg, 96 μmol, 92%). C_11_H_12_ClFN_2_O_4_S (322.74 g/mol). ^1^H NMR (400 MHz, CD_3_OD): δ 7.89 (d, *J* = 8.6 Hz, 1H), 7.67 (d, *J* = 1.3 Hz, 1H), 7.64 (d, *J* = 3.8 Hz, 1H), 7.42 (dd, *J* = 8.6, 1.7 Hz, 1H), 6.96 (dd, *J* = 3.8, 0.7 Hz, 1H), 4.33 (dd, *J* = 7.7, 5.6 Hz, 1H), 3.46 (dd, *J* = 14.6, 5.6 Hz, 1H), 3.35–3.27 (m, 2H). ^13^C­{^1^H} NMR (101 MHz, CD_3_OD): δ 171.12, 135.76, 132.70, 132.69, 128.34, 128.24, 124.20, 114.89, 112.42, 55.26, 37.10. ^19^F NMR (376 MHz, CD_3_OD): δ 51.40. HR-ESI-MS *m*/*z*: [M + H]^+^ Calcd for C_11_H_12_FN_2_O_4_S 287.04963; Found: 287.04984.

##### 4.1.6.28 4-(Methylamino)­benzaldehyde (**27**)^25^

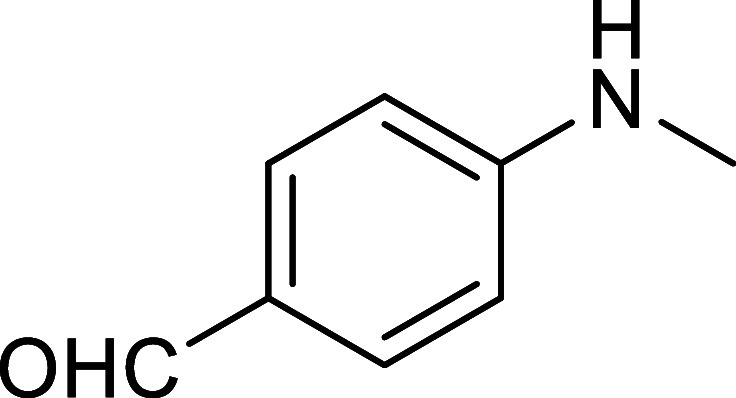



4-Bromobenzaldehyde (9.25 g, 50.0 mmol, 1.0 equiv) and copper powder (159 mg, 2.5 mmol, 0.05 equiv) were suspended in a solution of 40% MeNH_2_ in H_2_O (43.2 mL, 500 mmol, 10.0 equiv) in a PTFE reaction flask. The flask was placed in a high-pressure steel container and the reaction mixture was stirred for 48 h at 120 °C. After cooling to 23 °C, EtOAc (40 mL) was added and the aqueous phase was extracted with EtOAc (3 × 50 mL). The organic phase was dried over MgSO_4_ and the solvent was removed under reduced pressure. The crude product was purified by column chromatography (*n*-hexane:EtOAc/1:1.5, TLC: *R*_*f*_ = 0.48) to afford the title compound as a yellow solid (5.26 g, 38.9 mmol, 78%). C_8_H_9_NO (135.17 g/mol). ^1^H NMR (400 MHz, CDCl_3_): δ 9.71 (s, 1H), 7.78–7.55 (m, 2H), 6.74–6.50 (m, 2H), 4.61 (bs, 1H), 2.90 (s, 3H). ^13^C­{^1^H} NMR (101 MHz, CDCl_3_): δ 190.45, 154.42, 132.39, 126.36, 111.55, 30.08. ESI-MS *m*/*z*: [M + H]^+^ Calcd for C_8_H_10_NO 136.08; Found: 136.19.

##### 4.1.6.29 [4-(Methylamino)­phenyl]­methanol (**28**)^35^

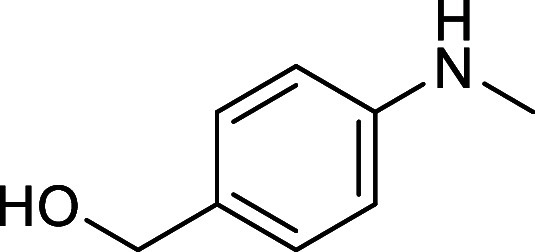



NaBH_4_ (1.40 g, 37.0 mmol, 2.5 equiv) was added to an ice-cooled solution of 4-(methylamino)­benzaldehyde (**27**) (2.00 g, 14.8 mmol, 1.0 equiv) in MeOH (50 mL) and the mixture was stirred for 3 h at 0 °C and 1 h at 23 °C. The solvent was removed under reduced pressure, the residue was taken up in H_2_O (50 mL) and the aqueous phase was extracted with EtOAc (3 × 50 mL). The organic phase was dried and concentrated under reduced pressure. The crude product was purified by column chromatography (*n*-hexane:EtOAc/1:1, TLC: *R*_*f*_ = 0.32) to afford the title compound as a colorless solid (1.86 g, 13.6 mmol, 92%). C_8_H_11_NO (137.18 g/mol). ^1^H NMR (400 MHz, CDCl_3_): δ 7.24–7.15 (m, 2H), 6.65–6.55 (m, 2H), 4.54 (s, 2H), 2.83 (s, 3H). (-O*H* was not detected) ^13^C­{^1^H} NMR (101 MHz, CDCl_3_): δ 149.16, 129.79, 128.90, 112.51, 65.47, 30.87. ESI-MS *m*/*z*: [M + H]^+^ Calcd for C_8_H_12_NO 138.09; Found: 138.19.

##### 4.1.6.30 [4-(Bromomethyl)­phenyl]­(methyl)­sulfamoyl Fluoride (**29**)

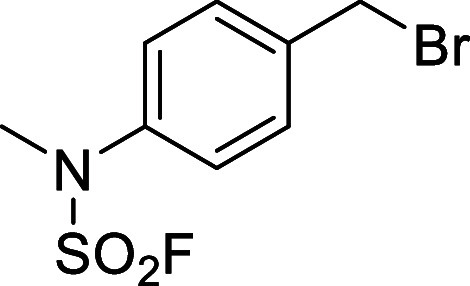



CBr_4_ (4.78 g, 13.6 mmol, 2.71 equiv) was added to an ice-cold solution of **11** (1.1 g, 5.02 mmol, 1.0 equiv) and PPh_3_ (3.65 g, 13.6 mmol, 2.71 equiv) in anhydrous CH_2_Cl_2_ (10 mL). After 10 min, the cooling bath was removed and the reaction mixture was stirred for 24 h. The mixture was loaded onto a pad of silica (30 g) and the crude product was eluted with CH_2_Cl_2_ (TLC-control). The resulting solution was concentrated under reduced pressure and the residue was purified by column chromatography (*n*-hexane:EtOAc/4:1, TLC: *R*_*f*_ = 0.56). The product fraction was concentrated under reduced pressure and the residue was dried under dynamic vacuum at 2 mbar and 50 °C to afford the title compound (1.37 g, 4.86 mmol, 97%) as a colorless solid. C_8_H_9_BrFNO_2_S (282.13 g/mol). ^1^H NMR (400 MHz, CDCl_3_): δ 7.52–7.43 (m, 1H), 7.41–7.32 (m, 1H), 4.48 (s, 2H), 3.43 (d, *J* = 2.2 Hz, 3H). ^13^C­{^1^H} NMR (101 MHz, CDCl_3_): δ 139.83 (d, *J* = 2.8 Hz), 138.85, 130.61, 127.04 (d, *J* = 2.0 Hz), 40.70 (d, *J* = 1.2 Hz), 32.04. ^19^F NMR (376 MHz, CDCl_3_) δ 42.62. HR-EI-MS *m*/*z*: [M]^•+^ Calcd for C_8_H_9_BrFNO_2_S 280.9516; Found: 280.9516.

##### 4.1.6.31 (*S,S*)-Ni-BPB-4-(NMeSO_2_F)­Phe (**30**)

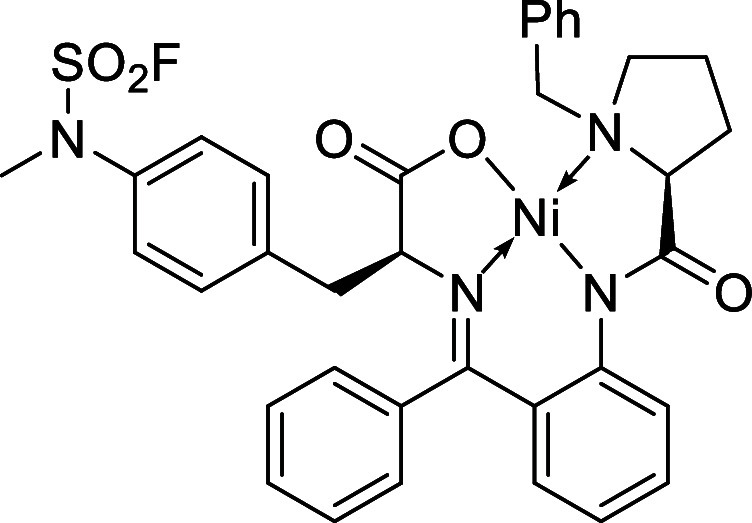



A suspension of (*S*)-Ni-BPB-Gly (1.92 g, 3.85 mmol, 1 equiv) in a solution of **29** (1.35 g, 4.79 mmol, 1.2 equiv) in anhydrous DMF/MeCN (15 mL, 1:2) was vigorously stirred until most of the complex was dissolved. The mixture was cooled to −20 °C (40% EtOH/liquid nitrogen) and NaH (60% suspension in mineral oil, 0.24 g, 6 mmol, 1.6 equiv) was added to the resulting suspension. After 5 min, the cooling bath was removed and the reaction mixture was vigorously stirred for 90 min. A weak exothermic reaction was observed when the reaction temperature reached approximately 10 °C. The reaction mixture (an almost clear solution) was poured into an ice-cold solution of AcOH (1 mL, 1.05 g, 16.65 mmol, 4.3 equiv) in H_2_O (1 L). The resulting suspension was stirred for 10 min, filtered, the filter cake was washed with H_2_O (4 × 50 mL), dried by suction, and dissolved in CH_2_Cl_2_ (10 mL). The resulting solution was dried and concentrated under reduced pressure. The residue was purified by column chromatography (CHCl_3_/acetone: 9:1, TLC: R_*f*_ = 0.37, two-time development) followed by trituration with Et_2_O to afford the title compound (2.38 g, 3.40 mmol, 88%) as a red solid. Additionally, the corresponding dialkylated Ni-complex **E1** (0.29 g, 0.32 mmol, 8%; TLC: CHCl_3_/acetone = 9/1, R_*f*_ = 0.42, two-time development; red solid) was isolated. **30**: C_35_H_33_FN_4_NiO_5_S (699.42 g/mol). ^1^H NMR (400 MHz, CDCl_3_): δ 8.28–8.19 (m, 1H), 8.04–7.95 (m, 2H), 7.63–7.47 (m, 2H), 7.41 (td, *J* = 7.6, 1.5 Hz, 1H), 7.37–7.27 (m, 5H), 7.15 (ddd, *J* = 8.5, 5.5, 3.3 Hz, 4H), 6.75–6.62 (m, 3H), 4.33 (d, *J* = 12.7 Hz, 1H), 4.25 (t, *J* = 5.6 Hz, 1H), 3.53 (d, *J* = 12.7 Hz, 1H), 3.41 (d, *J* = 2.0 Hz, 3H), 3.34 (dd, *J* = 10.6, 6.5 Hz, 1H), 3.29–3.15 (m, 2H), 3.01 (dd, *J* = 13.8, 6.2 Hz, 1H), 2.75 (td, *J* = 17.5, 15.4, 7.3 Hz, 1H), 2.57–2.34 (m, 2H), 1.97–1.83 (m, 2H). ^13^C­{^1^H} NMR (101 MHz, CDCl_3_) δ 180.46, 178.28, 171.61, 142.93, 139.15 (d, *J* = 2.5 Hz), 137.03, 134.06, 133.74, 133.25, 132.73, 131.77, 131.59, 130.03, 129.27, 129.18, 128.99 (×2), 127.92, 127.36, 126.87 (d, *J* = 1.5 Hz), 126.19, 123.54, 120.90, 71.28, 70.38, 63.22, 56.93, 40.78, 40.17, 30.99, 23.42. *o*- and *m*-Carbons of the phenyl group of the [(2-amido)­phenyl]­phenylmethanimine fragment are inequivalent. ^19^F NMR (376 MHz, CDCl_3_) δ 42.47. HR-ESI-MS *m*/*z*: [M + H]^+^ Calcd for C_35_H_34_FN_4_NiO_5_S 699.15819; Found: 699.15801. Correct isotopic pattern.
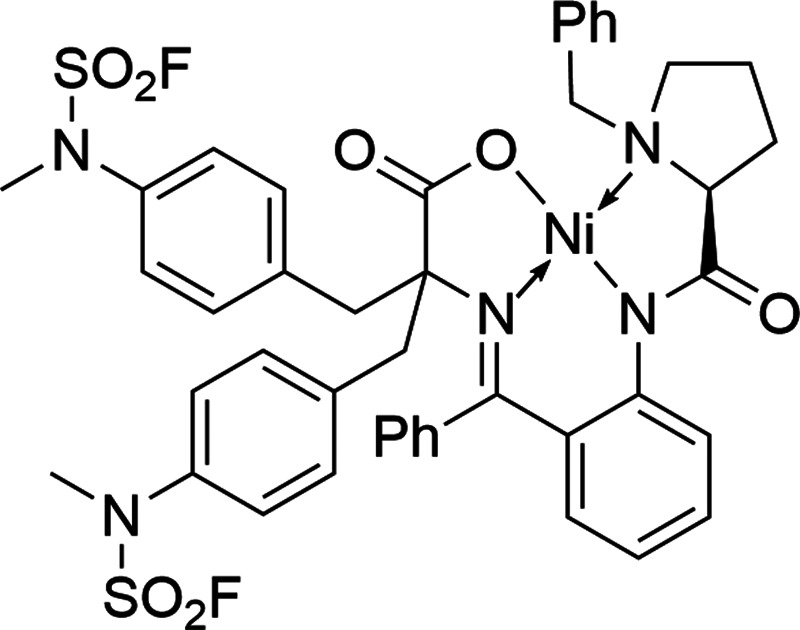


**E1**: C_43_H_41_F2N_5_NiO_7_S_2_ (900.64 g/mol). ^1^H NMR (400 MHz, CDCl_3_): δ 7.99–7.94 (m, 2H), 7.92 (dd, *J* = 8.6, 1.0 Hz, 1H), 7.60 (d, *J* = 8.6 Hz, 2H), 7.52 (dd, *J* = 15.2, 8.0 Hz, 3H), 7.48–7.44 (m, 2H), 7.43–7.37 (m, 4H), 7.34 (dd, *J* = 13.5, 6.1 Hz, 2H), 7.20 (t, *J* = 7.5 Hz, 1H), 7.14–7.03 (m, 3H), 6.61 (ddd, *J* = 8.2, 6.9, 1.2 Hz, 1H), 6.53 (dd, *J* = 8.5, 1.5 Hz, 1H), 4.32 (d, *J* = 12.7 Hz, 1H), 3.47 (d, *J* = 1.8 Hz, 3H), 3.43–3.51 (m, 1H), 3.40 (d, *J* = 2.0 Hz, 3H), 3.37 (d, *J* = 12.8 Hz, 1H), 3.30–3.24 (m, 1H), 3.24–3.17 (m, 3H), 3.04 (d, *J* = 14.5 Hz, 1H), 2.94 (d, *J* = 16.6 Hz, 1H), 2.38–2.27 (m, 1H), 2.27–2.16 (m, 1H), 2.16–2.01 (m, 1H), 1.83 (td, *J* = 10.7, 6.2 Hz, 1H), 1.74–1.63 (m, 1H). ^13^C­{^1^H} NMR (101 MHz, CDCl_3_): δ 180.64, 179.43, 173.15, 142.43, 139.37 (d, *J* = 2 Hz), 138.53 (d, *J* = 3 Hz), 137.94, 137.48, 136.68, 133.76, 133.54, 132.37, 132.27, 131.54, 130.82, 130.15, 129.18, 129.03, 128.89, 128.39, 128.05, 127.69, 127.63, 126.86, 126.78, 124.15, 121.00, 80.64, 69.89, 63.79, 57.64, 45.82, 44.59, 40.78, 30.86, 22.83. *o*- and *m*-Carbons of the phenyl group of the [(2-amido)­phenyl]­phenylmethanimine fragment are inequivalent. 4-[(Fluorosulfonyl)­(methyl)]­benzyl groups are inequivalent. ^19^F NMR (376 MHz, CDCl_3_) δ 42.64, 42.34. ESI-MS *m*/*z*: [M + H]^+^ Calcd for C_43_H_42_F_2_N_5_NiO_7_S_2_ 900.19; Found: 900.24. Correct isotopic pattern.

##### 4.1.6.32 (*S*)-2-[(*tert*-Butoxycarbonyl)­amino]-3-{4-[(fluorosulfonyl)­(methyl)­amino]­phenyl}­propanoic Acid (**31**)

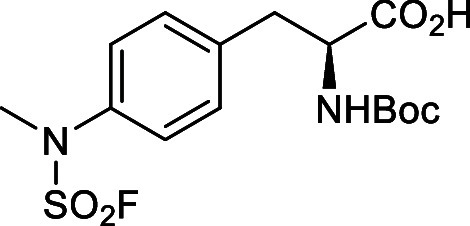



A solution of DTPA­(Et_3_N)_2_ was prepared from DTPA (2.81 g, 7.15 mmol, 10.0 equiv) and 25% Et_4_NOH (8.26 mL, 14.3 mmol, 20.0 equiv), and added to a solution of **30** (500 mg, 715 μmol, 1.0 equiv) in MeOH (25 mL). The red-colored reaction mixture was stirred for 96 h at 75 °C until a complete color change from red to blue was observed. The MeOH was removed under reduced pressure and the residual solution was cooled in an ice bath. The precipitate was isolated by filtration, washed with cold H_2_O, and dried to obtain (*S*)-2-[*N*-(*N*′-benzyl-prolyl)­amino]­benzophenone (BPB). The filtrate was basified with NaHCO_3_ to pH 8–9 and extracted with Et_2_O (3 × 40 mL). Boc_2_O (469 mg, 2.15 mmol, 3.0 equiv) was added to the aqueous phase and the mixture was homogenized by the addition of MeOH. The mixture was stirred for 16 h at ambient temperature. Two further portions of Boc_2_O (469 mg, 2.15 mmol, 3.0 equiv) were added in 2 h intervals. Thereafter, the MeOH was removed under reduced pressure and the remaining solution was extracted with Et_2_O (2 × 40 mL). The aqueous phase was acidified to pH 2–3 with solid NaHSO_4_, followed by extraction with Et_2_O (2 × 40 mL). The organic phase was washed with 1 m NaHSO_4_ (2 × 40 mL), H_2_O (2 × 40 mL), and brine (40 mL), dried and concentrated under reduced pressure to afford the title compound as a colorless solid (248 mg, 659 μmol, 92%). C_18_H_21_FN_2_O_6_S (376.40 g/mol). ^1^H NMR (400 MHz, CD_3_OD): δ 7.36 (s, 4H), 4.38 (dd, *J* = 9.2, 4.7 Hz, 1H), 3.41 (d, *J* = 1.9 Hz, 3H), 3.22 (dd, *J* = 13.8, 4.8 Hz, 1H), 2.94 (dd, *J* = 13.7, 9.5 Hz, 1H), 1.37 (s, 9H).^13^C­{^1^H} NMR (101 MHz, CD_3_OD): δ 175.02, 157.79, 139.94 (d, *J* = 2.3 Hz), 131.78, 127.75, 80.56, 56.02, 41.14 (d, *J* = 1.1 Hz), 38.25, 28.65. ^19^F NMR (376 MHz, CDCl_3_) δ 39.55. HR-ESI-MS *m*/*z*: [M + Na]^+^ Calcd for C_18_H_21_FN_2_O_6_SNa 399.09966; Found: 399.09954.

##### 4.1.6.33 *tert*-Butyl (*S*)-2-[(*tert*-butoxycarbonyl)­amino]-3-{4-[(fluorosulfonyl)­(methyl)­amino]­phenyl} Propanoate (**32**)

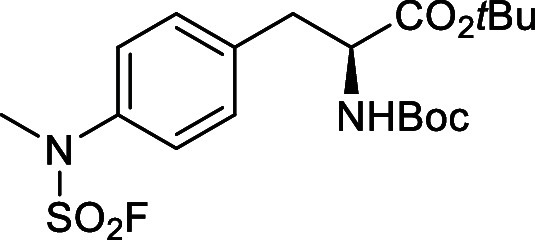



*tert*-Butyl 2,2,2-trichloroacetimidate (262 mg, 1.20 mmol, 3.0 equiv) was added to a solution of **31** (150 mg, 399 μmol, 1.0 equiv) in dry CH_2_Cl_2_ (0.2 m) and the mixture was stirred for 16 h at 48 °C. The flask was then cooled to −18 °C, a cold mixture of CH_2_Cl_2_/*n*-pentane (1:1, 20 mL, −18 °C) was added, and the resulting precipitate was removed by filtration. The filtrate was concentrated under reduced pressure and the residue was taken up in Et_2_O. The organic phase was successively washed with H_2_O (×1) and brine (×2), before it was dried and concentrated under reduced pressure. The residue was taken up into *n*-pentane, the precipitate was removed by filtration, and the filtrate was concentrated under reduced pressure. The crude product was purified by column chromatography (*n*-hexane:EtOAc = 5:1, TLC *R*_*f*_ = 0.21) to afford the title compound (158 mg, 365 μmol, 91%) as a colorless oil. C_19_H_29_FN_2_O_6_S (432.51 g/mol). ^1^H NMR (400 MHz, CDCl_3_): δ 7.35–7.21 (m, 4H), 5.04 (d, *J* = 7.7 Hz, 1H), 4.45 (q, *J* = 6.5 Hz, 1H), 3.41 (s, 3H), 3.21–2.87 (m, 2H), 1.42 (s, 9H), 1.39 (s, 9H). ^13^C­{^1^H} NMR (101 MHz, CDCl_3_): δ 170.77, 155.14, 138.64, 137.95, 131.08, 126.59, 82.59, 80.01, 54.81, 40.80 (d, *J* = 1.0 Hz), 38.44, 28.43, 28.05. ^19^F NMR (376 MHz, CD_3_OD): δ 42.09. HR-ESI-MS *m*/*z*: [M + Na]^+^ Calcd for C_19_H_29_FN_2_O_6_SNa 455.16225; Found: 455.16238.

##### 4.1.6.34 (*S*)-2-Amino-3-{4-[(fluorosulfonyl)­(methyl)­amino]­phenyl}­propanoic Acid Hydrochloride (**33**·HCl)

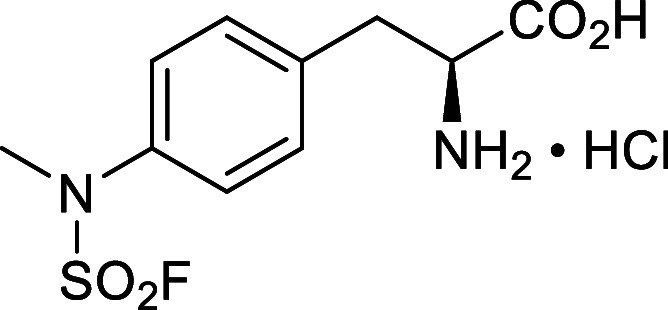



For the deprotection of **32**, a solvent-free method described by Verschueren et al. was used.^30^ To this end, NaCl (93 mg, 1.59 mmol, 10.0 equiv) was placed in chamber one of a two-chamber reactor and **32** (60 mg, 159 μmol, 1.0 equiv) was placed in chamber two. Concentrated H_2_SO_4_ (0.5 mL) was added to chamber one via a syringe to generate HCl and the setup was left for reaction for 16 h. The title compound was collected from chamber two as a colorless solid (46 mg, 147 μmol, 93%). C_10_H_14_FClN_2_O_4_S (312.74 g/mol). ^1^H NMR (400 MHz, CD_3_OD): δ 7.56–7.37 (m, 4H), 4.31 (dd, *J* = 7.6, 5.6 Hz, 1H), 3.44 (s, 3H), 3.37 (dd, *J* = 14.6, 5.6 Hz, 1H), 3.22 (dd, *J* = 14.6, 7.6 Hz, 1H). ^13^C­{^1^H} NMR (101 MHz, CD_3_OD): δ 170.99, 140.95 (d, *J* = 2.7 Hz), 136.89, 132.03, 128.47 (d, *J* = 1.8 Hz), 54.85, 41.09 (d, *J* = 1.0 Hz), 36.75. ^19^F NMR (376 MHz, CD_3_OD): δ 39.95. HR-ESI-MS *m*/*z*: [M + H]^+^ Calcd for C_10_H_14_FN_2_O_4_S 277.06528; Found: 277.06541.

### 4.2 Radiochemistry

#### 4.2.1 General

[^18^F]­Fluoride ([^18^F]­F^–^) was produced via the ^18^O­(p,n)^18^F reaction by bombardment of enriched [^18^O]­H_2_O with 17 MeV protons in a BC1710 cyclotron (The Japan Steel Works, Tokyo, Japan) or a GE PETtrace (GE Healthcare, Chicago, USA), both located at the INM-5 (Forschungszentrum Jülich GmbH). Radioactivity was measured using a CRC-120 Radioisotope Calibrator (Capintec Inc., Florham Park, Netherlands) or a Curiementor 2 (PTW, Freiburg, Germany). The following cartridges were used for radiosyntheses and solid-phase extractions (SPE): Sep-Pak Plus Light QMA Carbonate cartridges (130 mg sorbent per cartridge, preconditioned with 1 mL H_2_O) from Waters GmbH (Eschborn, Germany) and Strata-X 33 μm Polymeric RP cartridges [30 mg sorbent, preconditioned with EtOH (2 mL) and H_2_O (10 mL)] from Phenomenex (Aschaffenburg, Germany).

Radiochemical yields (RCYs) corrected for decay to the start of synthesis and/or activity yields (AYs, non-decay-corrected) are provided for analytically pure radiolabeled compounds. Radiochemical conversions (RCCs) were determined by HPLC analysis after dilution of the reaction mixtures with H_2_O as follows. The decay corrected product peak areas in the chromatograms were compared to the peak area obtained by a postcolumn injection (p.c.i.).^36^ The identity of radiolabeled products was confirmed by coinjection of the nonradioactive reference compounds if the amount of unlabeled precursor was insufficient for reliable detection.

HPLC analyses were carried out on a Dionex Ultimate 3000 System with Ultimate 3000 RS Variable Wavelength Detector or Ultimate 3000 Diode Array Detector, coupled in series with a Berthold LB500 NaI detector. Two Rheodyne 6-port injection valves equipped with equal sample loops were installed before and behind the column. The UV and radioactivity detectors were coupled in series, giving a time delay of 0.1–0.5 min (depending on flow rate) between the corresponding responses. Columns and methods are given for each chromatogram (see SI).

All radiosyntheses were carried out according to the radiation protection rules of our institute using appropriate personal protective equipment and shielding. Unless noted otherwise, 5 mL screw-top V-Vials (20–400 thread, 20 mm × 65 mm) were used for all radiosyntheses. Prior to their first use and after every 10–15 reactions, the vials were (re)­siliconized to minimize adsorption of [^18^F]­F^–^ to the vessel walls. To this end, the vials were incubated for 10 min with a solution of Me_2_SiCl_2_ (5%) in CH_2_Cl_2_. Following incubation, the vials were sequentially rinsed with EtOAc, CH_2_Cl_2_, acetone, H_2_O, acetone, and MeOH. During the H_2_O and acetone rinsing steps, mechanical assistance with a lint-free paper towel was applied to enhance cleaning efficiency. Finally, the vials were dried at 90 °C for 10 min prior to (re)­use.

#### 4.2.2 General SuFEx Radiolabeling Protocol (GP1)

1. Aqueous [^18^F]­F^–^ (10–30 MBq) was loaded (from the male side) onto a Sep-Pak Plus Light QMA Carbonate cartridge [130 mg sorbent, preconditioned with H_2_O (1 mL)].
2. The cartridge was washed (from the male side) with MeOH (2 mL) to remove residual water.
3. [^18^F]­F^–^ was eluted (from the female side) with a solution of BnEt_3_NCl or Et_4_NOTf (10 μmol) in MeOH (1 mL).
4. The MeOH was evaporated at 80–90 °C under dynamic vacuum (400 mbar) and in a stream of argon for 3–5 min. Complete removal of MeOH was verified by visual inspection (*thorough drying is essential and should not be guided by time alone!*).
5. After equilibration of the reaction vessel to the desired temperature (3 min), a solution of the precursor (30–1000 nmol, depending on the substrate) in MeCN (1 mL) was added.
6. The reaction was allowed to proceed for 1–10 min at 23–100 °C, depending on the specific substrate.
7. The reaction was quenched by the addition of H_2_O (2–4 mL) and the RCC was determined by radio-HPLC.

#### 4.2.3 Preparation of [^18^F]­3 and [^18^F]­5 Using K_222_/K_2_CO_3_ and Inductive Heating

[^18^F]**3** and [^18^F]**5** were additionally prepared using a modified version of the K_222_/K_2_CO_3_-based protocol with inductive heating, adapted from Jeon et al. to avoid labeling with aliquots.^11^ Aqueous [^18^F]­F^–^ was loaded (from the male side) onto a Sep-Pak Plus Light QMA Carbonate cartridge (130 mg sorbent, preconditioned with 10 mL H_2_O). The trapped [^18^F]­F^–^ was then eluted with a solution of K_222_ (3.76 mg, 9.99 μmol) and K_2_CO_3_ (0.69 mg, 5.0 μmol) in MeCN:H_2_O (96:4, 1.2 mL) into a borosilicate glass vessel equipped with a silicone/PTFE septum cap (Anton Paar GmbH, Austria). The solvent was removed at 110 °C under dynamic vacuum (400 mbar) in a stream of argon for 10–12 min. After cooling for 3 min, the dried [^18^F]­F^–^ complex was redissolved in MeCN (1 mL), and a solution of the precursor (100 nmol) in MeCN (1 mL) was added. The reaction was performed in an inductively heated synthesis reactor (Monowave 50, Anton Paar GmbH, Austria). After 10 min at 60 °C, the reaction was quenched by the addition of H_2_O:MeCN (1:1, 2 mL) and the RCC was determined by radio-HPLC.

For comparison, labeling with our base-free protocol according to GP1 was performed using identical precursor amounts (100 nmol) and solvent volumes (2 mL). **5** was labeled at 40 °C for 3 min, and **3** was labeled at 80 °C for 10 min.

#### 4.2.4 Preparation of Boc-Trp­(SO_2_[^18^F]­F)-OMe ([^18^F]­2)

The general radiolabeling protocol GP1 was followed up to step 4, after which the procedure continued as follows:

1. After equilibration of the reactor to 40 °C, a solution of precursor **2** (30 nmol) in MeCN (1 mL) was added.
2. The reaction mixture was incubated for 3 min at 40 °C.
3. The reaction was quenched by the addition of H_2_O (9 mL) and the RCC was determined by radio-HPLC.
4. The crude reaction mixture was loaded (from the female side) onto a Strata-X RP (30 mg) cartridge.
5. The cartridge was dried with air (50 mL syringe) and the product was eluted with EtOH (1 mL).
6. The EtOH was removed at 80 °C under dynamic vacuum (400 mbar) in a stream of argon.
7. The tracer was reconstituted in 1% Tween 80 solution (200 μL).

#### 4.2.5 Preparation of [^18^F]­20, (*S,S*)*-*[^18^F]­21, (*S,R*)*-*[^18^F]­21, [^18^F]­26, and [^18^F]­33

The general radiolabeling protocol GP1 was followed up to step 4, after which the procedure continued as follows:

1. After equilibration of the reactor to the target temperature (40 °C for **1** and **25**; 90 °C for **14** and 80 °C for **32**), a solution of the precursor (**1** and **25**: 30 nmol,* **14**: 100 nmol,* **32**: 200 nmol) in MeCN (1 mL) was added.
2. The reaction mixture was either incubated for 3 min at 40 °C (**1** and **25**) or for 10 min at 90 °C (**14**) or 80 °C (**32**).
3. The reaction was quenched by the addition of H_2_O (9 mL) and the RCC was determined by radio-HPLC.
4. The crude reaction mixture was loaded (from the female side) onto a Strata-X RP (30 mg) cartridge.
5. The cartridge was dried with air (50 mL syringe) and the product was eluted with acetone (500 μL).
6. The acetone was removed at 80 °C under dynamic vacuum (400 mbar) in a stream of argon within 2–3 min.
7. The residue was treated with 6 m HCl (100 μL) and heated at 90 °C (70 °C for [^18^F]**25**) for 10 min to cleave the protecting groups.
8. HCl was removed under dynamic vacuum (400 mbar) in a stream of argon at 90 °C for 5–10 min using a liquid N_2_-cooled trap.
9. The tracer was reconstituted in isotonic NaCl solution or 0.1 m NaOAc buffer (pH 5.3).**

*Probes for the *in vivo* experiments were prepared using 100 nmol of **1** and **25**, and 200 nmol of (*S,S*)-**14** and **32**.

** For *in vivo* studies, formulation of the tracers in 0.1 m NaOAc buffer was preferred over isotonic NaCl to maintain an acceptable pH for injection (4.5–8.5, per European Pharmacopeia for [^18^F]­FET). In small formulation volumes (e.g., 500 μL), residual HCl occasionally reduced the pH to <3 if isotonic NaCl was used.

#### 4.2.6 Preparation of [^18^F]­FET

[^18^F]­FET was produced at the radiopharmaceutical unit of INM-5 (Forschungszentrum Jülich) as described previously.^37^

### 4.3 Preclinical Evaluation

#### 4.3.1 pH Stability Tests

[^18^F]**3**, [^18^F]**4**, and [^18^F]**5** were prepared according to GP1 (using 50 nmol of precursor). The reaction mixtures were diluted with H_2_O (9 mL), and the crude products were loaded (from the female side) onto Strata-X RP cartridges (30 mg). The cartridges were washed (from the female side) with H_2_O (10 mL) and dried with air (50 mL syringe). The radiolabeled products were then eluted with MeCN (500 μL), followed by the addition of H_2_O (500 μL). [^18^F]**20** and (*S,S*)-[^18^F]**21** were prepared as described in section 4.2.5.

For stability testing, an aliquot (1–2 MBq, 100 μL) of each radiotracer solution was mixed with an equal volume (100 μL) of aqueous solution at a defined pH (**Table S2**) and the resulting mixtures were incubated at ambient temperature. At predetermined time points (5, 30, 60, 90, and 120 min), small aliquots (2.5 μL) of each solution were collected and spotted on a TLC plate. The nonradioactive reference compounds were spotted on a separate lane. The TLC plates were air-dried and developed using an appropriate mobile phase. After development, UV-active spots of the reference compounds, along with the origin and solvent front at both edges of the TLC plate, were spiked with a diluted radioactive solution to aid alignment. The TLC plates were then analyzed using a phosphor imager (BAS 5000, Fuji Pharma, Japan) and phosphor imaging plates (Fuji Pharma, Japan). Radiolabeled compounds were identified by comparing their migration distances with those of the nonradioactive reference compounds. pH values of the buffer solutions were measured using a model 766 pH meter (Knick, Berlin, Germany) equipped with an Inlab Micro electrode (Mettler Toledo, Columbus, USA).

#### 4.3.2 Stability of H*-*Trp­(SO_2_[^18^F]­F)*-*OH ([^18^F]­20) and (*S,S*)*-*H*-*Dht­(SO_2_[^18^F]­F)*-*OH {(*S,S*)*-*[^18^F]­21} in Human Blood Plasma

The stability of H-Trp­(SO_2_[^18^F]­F)-OH ([^18^F]**20**) and (*S,S*)*-*H-Dht­(SO_2_[^18^F]­F)-OH {(*S,S*)-[^18^F]**21**} was assessed in human plasma obtained by centrifugation (300 rcf) of fresh, heparinized, or citrated blood. For each tracer, 500 μL of plasma was prewarmed in a 2 mL Eppendorf vial at 37 °C for 5 min using a thermoshaker. A solution of the radiotracer in isotonic NaCl (10 μL, approximately 0.5–1 MBq) was then added, and the mixture was further shaken at 37 °C. Aliquots (80 μL) of the mixtures were removed at 5, 30, 60, 90, and 120 min. To determine the recovery rate, 1 μL of each aliquot was immediately spotted on a paper strip (backside coated with a polymer layer), while 60 μL were transferred into an Eppendorf vial containing 120 μL of MeCN for precipitation of plasma proteins. The vials were vigorously vortexed for 2 min and centrifuged at 20,000 rcf for 2 min, after which the supernatant was spotted on a TLC plate (three lanes, 2 μL per time point). The nonradioactive reference compounds were spotted on a separate lane. The TLC plates were air-dried and developed using *n*-butanol/H_2_O/AcOH (5:1:1) as the mobile phase. After development, the UV-active spots of the reference compounds, the origin, and the solvent front at both edges of the TLC plate were spiked with a diluted radioactive solution to aid alignment. The TLC plates and the paper strips with untreated plasma samples were simultaneously scanned using a phosphor imager (BAS 5000, Fuji Pharma, Japan) and phosphor imaging plates (Fuji Pharma, Japan). Radioactivity recovery for the precipitation step was calculated by comparing the activity of the untreated plasma samples (paper strips) and the supernatant after protein precipitation, taking into account the dilution factor. H-Trp­(SO_2_[^18^F]­F)-OH ([^18^F]**20**) and (*S,S*)*-*H-Dht­(SO_2_[^18^F]­F)-OH {(*S,S*)-[^18^F]**21**} were identified by comparing their migration distances with those of the nonradioactive reference compounds.

#### 4.3.3 Cell Culture

Human U87 MG glioblastoma cells were purchased from the American Type Culture Collection (ATCC) and cultured under normal growth conditions (37 °C and 5% CO_2_) in minimum essential medium GlutaMAX (MEM, Gibco 41090 028, Fisher Scientific GmbH, Schwerte, Germany) supplemented with 10% fetal bovine serum (FBS, Sigma-Aldrich F2442, Merck KGaA, Darmstadt, Germany) and 1% penicillin/streptomycin (Gibco 115140 122). The cells were grown in cell-culture dishes (Thermo Fisher 150350, F 100 mm) with 9 mL culture medium and routinely passaged every 4–5 days after they had reached 80–90% confluency. For the cellular uptake and inhibition studies, cells were seeded into 12-well plates (2 × 10^5^ cells in 1 mL medium/well) 48 h before the start of the experiments.

#### 4.3.4 Cellular Uptake Experiments

Two hours before the start of the experiments, the culture medium was carefully aspirated, the cells were washed with phosphate-buffered saline (PBS, 1 mL, Gibco 10 010 023), and a dye exclusion test with trypan blue (Sigma-Aldrich T 8154) was performed to determine cell viability and the exact cell count (cell viability was always >95%). The tracer solutions were prepared in FBS- and amino acid-free Dulbecco's phosphate-buffered saline (DPBS) at a concentration of 150 kBq/mL. PBS was removed from the wells, and the tracer solution was added (1 mL/well). The cells were then incubated with the tracer solution at 37 °C for 60 min, washed twice with ice-cold PBS (1 mL), trypsinized, and harvested. The accumulated radioactivity was measured on an automatic γ counter (PerkinElmer, 1480 Automatic γ Counter, Shelton, USA) and the counts were normalized to the cell number. Each experiment was conducted at least in triplicate.

#### 4.3.5 Cellular Inhibition Studies

For the inhibition experiments, U87 MG cells were used and cultured as described above. The following inhibitors, obtained from Sigma-Aldrich, were used: 2-(methylamino)-2-methylpropionic acid (MeAIB) for system A, l-serine for system ASC, and 2-aminobicyclo­[2,2,1]­heptane-2-carboxylic acid (BCH) for system L transporters. The inhibitors were diluted with DPBS to give the desired final concentrations (1.5 nm, 15 nm, or 150 nm) and added together with the tracer. After incubation for 60 min at 37 °C, the cells were processed, and the uptake of radioactivity was measured, as described in section 4.3.4. Cellular uptake in the presence of different inhibitor concentrations was then normalized by the mean value observed under control conditions (i.e., in the absence of inhibitor).

#### 4.3.6 *In Vivo* Experiments

All experiments were carried out in accordance with the EU directive 2010/63/EU for animal experiments and the German Animal Welfare Act (TierSchG, 2006) and were approved by the regional authorities (State Office for Nature, Environment and Consumer Protection of North-Rhine Westphalia [LAVE NRW], Dept. Animal Welfare, license number: 81–02.04.2023.A034).

#### 4.3.7 μPET Imaging in Healthy Mice

Four healthy male C57BL/6 mice (35–40 g) were anesthetized with isoflurane (5% for induction, 2% for maintenance) in O_2_/air (3:7), and a catheter for tracer injection was inserted into the lateral tail vein. Mice were placed on an animal holder (Medres, Cologne, Germany) and fixed with a tooth bar in a respiratory mask. Body temperature was maintained at 37 °C by continuous warm water flow through the wall of the animal bed. Eyes were protected from drying with Bepanthen eye and nose ointment (Bayer, Leverkusen, Germany). A dynamic PET scan in list mode was conducted using a Focus 220 micro PET scanner (CTI-Siemens, Erlangen, Germany) with a resolution at the center of the field of view of 1.4 mm. Data acquisition started with intravenous injection of a tracer (9.7 ± 0.2 MBq [^18^F]**2**; 10.9 ± 0.5 MBq [^18^F]**26**; 10.1 ± 1.5 MBq [^18^F]**33** in 125 μL) and ended after 120 min. This was followed by a 10 min transmission scan using a ^57^Co point source for attenuation correction. After the scan was finished, the catheter was removed and the mice were allowed to recover in their home cages. Following Fourier rebinning, data were reconstructed in two ways: 4 × 30 min frames for visual display and 28 frames (2 × 1 min, 2 × 2 min, 6 × 4 min, 18 × 5 min) for time-activity curves. An iterative OSEM3D/MAP algorithm with attenuation and decay correction was used. The resulting voxel sizes were 0.47 mm × 0.47 mm × 0.80 mm. All further analyses were performed with the software VINCI 5.24 (Max Planck Institute for Metabolism Research, Cologne, Germany). Standardized uptake values based on body weight (SUV_bw_) were determined according to the following equation: SUV_bw_ = radioactivity [Bq/g] × body weight [g] × 100/injected dose [Bq].

#### 4.3.8 Subcutaneous Glioma Model

Four male CB17-SCID mice (Janvier Laboratories, Le Genest-Saint-Isle, France) were used for subcutaneous tumor xenografts. To promote tumor cell survival and growth, natural killer cell activity was reduced by intraperitoneal injection of 20 μL Anti asialo GM1 rabbit (Fujifilm Wako Chemicals Europe GmbH, Neuss, Germany) diluted with 80 μL 0.9% NaCl 24 h before tumor inoculation. For tumor inoculation, 1 × 10^7^ U87 MG cells were resuspended in 75 μL culture medium, mixed with 75 μL Corning Matrigel (Merck KGaA, Darmstadt, Germany), and injected subcutaneously in the right shoulder region. After 2 weeks, the mice developed palpable tumors of about 0.5–1 cm^3^. PET scans were performed as described above with a scan time of 120 min after tracer injection (15.5 ± 0.8 MBq [^18^F]**20**; 12.6 ± 0.7 MBq (*S*,*S*)-[^18^F]**21**; 13.0 ± 1.8 MBq [^18^F]­FET in 125 μL i.v.). Images were reconstructed and SUV_bw_ was calculated as described above.

## Supplementary Material





## Data Availability

The data underlying this study are available in the published article and its Supporting Information.

## Notes

Details on the preparation and attempted deprotection of Boc-His­(SO_2_F)-O*t*Bu (**S2**), NMR spectra, HPLC chromatograms, details on the determination of molar activity, radio-TLC chromatograms for the pH stability tests, data on hydrolytic stability of [^18^F]**15** and ester cleavage during labeling of **2**, blood time-activity curve for experiments shown in Figure 4 (PDF)
Molecular formula strings (CSV)

## Abbreviations Used

[^18^F]­FET: *O*-(2-[^18^F]­fluoroethyl)­tyrosine
*A*_m_: molar activity
AISF: [4-(acetylamino)­phenyl]­imidodisulfuryl difluoride
ArSAFs: aromatic sulfamoyl fluorides
ASC: alanine/serine/cysteine transporter
AY: activity yield
BCH: 2-aminobicyclo­[2,2,1]­heptane-2-carboxylic acid
BPB: (*S*)-2-[*N*-(*N*′-benzyl-prolyl)­amino]­benzophenone
DBU: 1,8-diazabicyclo­[5.4.0]­undec-7-ene
Dcpm: dicyclopropylmethyl
desMe-SuFEx-IT: 1-(fluorosulfonyl)-3-methyl-1*H*-imidazole trifluoromethanesulfonate
Dht: 2,3-dihydrotryptophan
DMAP: 4-(dimethylamino)­pyridine
DTPA: diethylenetriaminepentaacetic acid
EDC: 1-ethyl-3-(3-(dimethylamino)­propyl)­carbodiimide
HPLC: high-performance liquid chromatography
IEX: isotopic exchange
iso NaCl: isotonic saline
LAT1: l-type amino acid transporter
MeAIB: α-(methylamino)­isobutyric acid
PBS: phosphate-buffered saline
PET: positron emission tomography
QMA: quaternary methylammonium
RCC: radiochemical conversion
RCP: radiochemical purity
SAF: sulfamoyl fluoride
SPE: solid-phase extraction
SuFEx: sulfur fluoride exchange
SUV_bw_: standardized uptake value normalized by body weight
TAC: time-activity curve
TBRs: target-to-background ratios
Tf: triflate
U87 MG: Uppsala 87 malignant glioma
VOI: volume of interest
