# Supplementary material for: Aryl Sulfamoyl [18F]Fluorides: Preparation via Base-Free SuFEx 18F‑Fluorination and Assessment of Their Applicability as PET Tracers
Source: J Med Chem. 2026 May 7;69(10):12324–45. doi: 10.1021/acs.jmedchem.6c00147 (PMC13224094; doi:10.1021/acs.jmedchem.6c00147)
Supplement: Supplementary file 1 [file jm6c00147_si_001.pdf]

Supporting Information for

Arylsulfamoyl [^{18}F]Fluorides: Preparation via Base-free SuFEx ^{18}F-Fluorination and Assessment of their Applicability as PET Tracers

Jan Bertram[a,b], Otari Gokhadze[a,b], Felix Neumaier[a,b], Qinyu Wang[b], Andreas Dreiling[a,b], Lukas Vieth[a,b], Heike Endepols[a,,b,c], Bernd Neumaier[a,b],* Boris D. Zlatopolskiy[a,b]

[a] Forschungszentrum Jülich GmbH, Institute of Neuroscience and Medicine, Nuclear Chemistry (INM-5), Wilhelm-Johnen-Straße, 52425 Jülich, Germany, j.bertram@fz-juelich.de (JB), adreilin@smail.uni-koeln.de (AD), f.neumaier@fz-juelich.de (FN), l.vieth@fz-juelich.de (LV), o.gokhadze@fz-juelich.de (OG), b.neumaier@fz-juelich.de (BN)

[b] University of Cologne, Faculty of Medicine and Cologne University Hospital, Institute of Radiochemistry and Experimental Molecular Imaging, Kerpener Straße 62, 50937 Cologne, Germany, qinyu.wang@uk-koeln.de (QW), heike.endepols@uk-koeln.de (HE), boris.zlatopolskiy@uk-koeln.de (BDZ)

[c] University of Cologne, Faculty of Medicine and University Hospital Cologne, Department of Nuclear Medicine, Kerpener Straße 62, 50937, Cologne, Germany

*correspondence: b.neumaier@fz-juelich.de

Table of contents

Preparation of Boc-His(SO_2F)-O*t*Bu (S2) 3
Deprotection of S2 7
NMR spectra 8
Chromatograms 64
Chromatograms of purified compounds 73
Determination of molar activity of [^{18}F]2 76
Radio-TLCs pH stability tests (after 120 min) 77
Hydrolytic stability of [^{18}F]15 81
Ester cleavage during labeling of methyl ester 2 81
Blood time-activity-curve (TAC) for experiments shown in Fig.4 82
References 82

Preparation of Boc-His(SO_2F)-O*t*Bu (S2)

tert-Butyl (*tert*-butoxycarbonyl)-L-histidinate (Boc-His-OtBu, **S1**)[1]

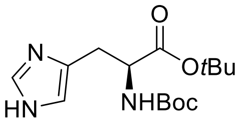

S1

2-*tert*-Butyl-1,3-diisopropyl isourea (8.0 mL, 6.72 g, 33.55 mmol, 3.4 eq) was added to a suspension of Boc-His-OH (2.55 g, 10 mmol, 1 eq) in anhydrous CH_2Cl_2 and the reaction mixture was stirred for 72 h. The resulting suspension was filtered, the filtrate was concentrated under reduced pressure, and the residue was purified by column chromatography ($CHCl_3$:MeOH = 8:1, TLC: R_f = 0.49) to afford the title compound (1.60 g, 5.14 mmol, 51%) as a colorless foam. $C_{15}H_{25}N_3O_4$ (311.38 g/mol). ^{1}H NMR (400 MHz, CD_3OD): δ = 7.59 (s, 1H), 6.85 (s, 1H), 4.25 (dd, *J* = 8.2, 6.4 Hz, 1H), 2.96 (ddd, *J* = 23.2, 14.7, 6.4 Hz, 2H), 1.42 (s, 18H). ^{13}C{^{1}H} NMR (101 MHz, CD_3OD): δ = 172.93, 157.80, 136.17, 118.66, 82.63, 80.48, 55.93, 30.31, 28.69, 28.20. The signal of C-4 of the imidazole ring was not observed. ESI-MS (negative mode) *m/z*: [M–H]$^-$: 310.20. ESI-MS (positive mode) *m/z*: [M+H]$^+$: 312.23.

^{1}H NMR

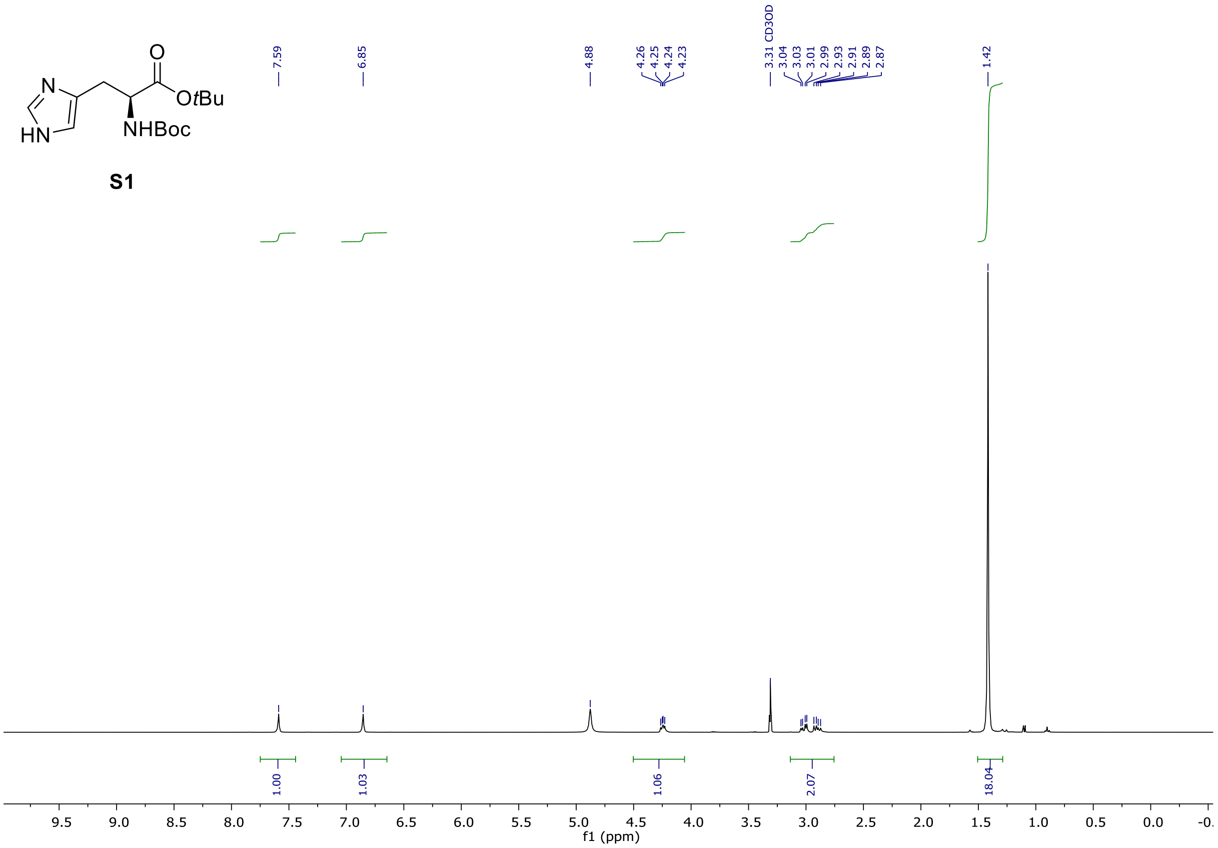

$^{13}C\{^{1}H\}$ NMR

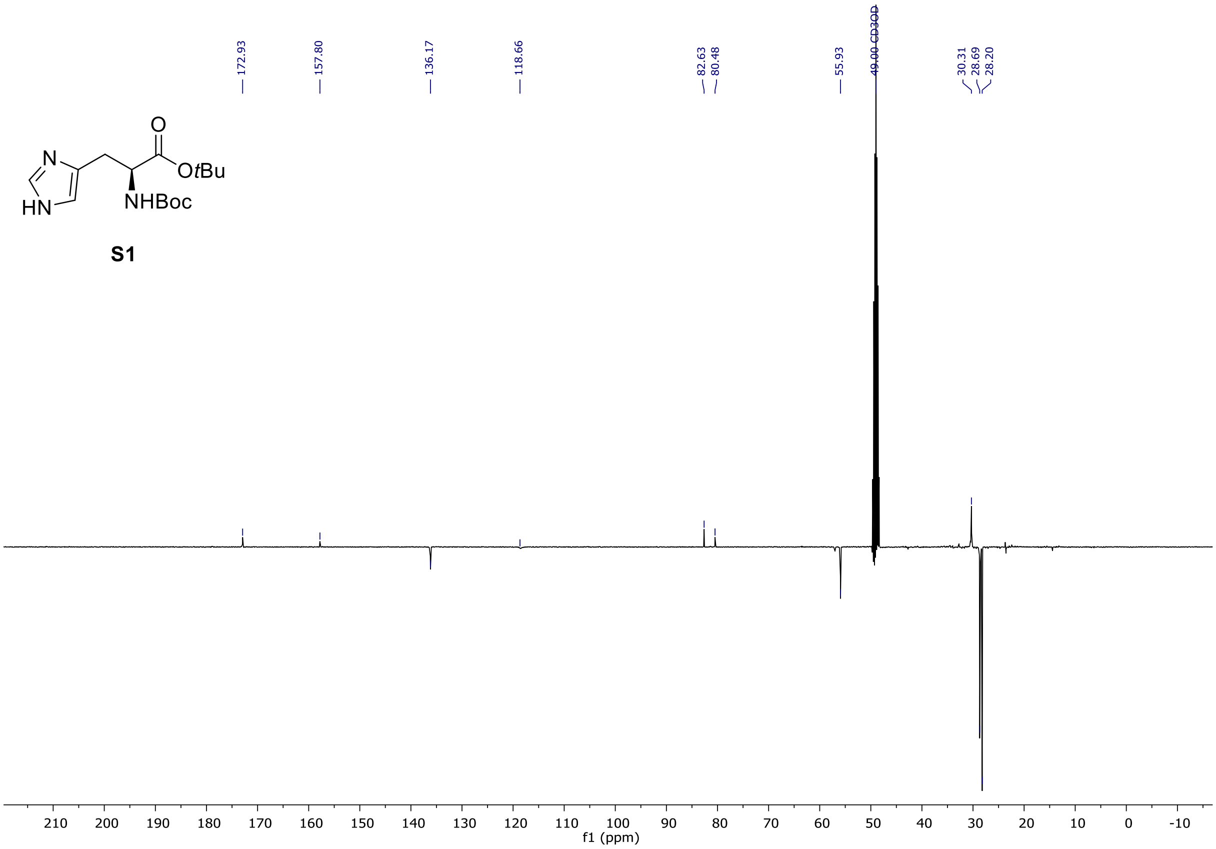

tert-Butyl N^{α}-(*tert*-butoxycarbonyl)-N^{τ}-(fluorosulfonyl)-L-histidinate (Boc-His(SO_2F)-O*t*Bu, **S2**)

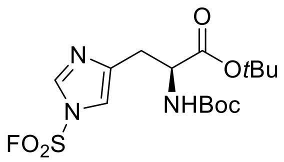

S2

DBU (1.6 mL, 1.63 g, 10.72 mmol, 2.2 eq.) was added to a solution of **S1** (1.54 g, 4.95 mmol, 1.0 eq.) and AISF (1.86 g, 5.92 mmol, 1.2 eq.) in anhydrous THF (30 mL) and the reaction mixture was stirred for 3 h. The reaction mixture was then diluted with Et_2O (100 mL) and washed with 0.5 M $NaHSO_4$ (3×30 mL) and brine (2×10 mL). The organic phase was dried and concentrated under reduced pressure. The residue was purified by column chromatography (twice; first using $CHCl_3$:MeOH/8:1, TLC: R_f = 0.9, and then using hexane:EtOAc/5:1, TLC: R_f = 0.58) to afford the title compound (1.08 g, 2.75 mmol, 56%) as a colorless oil, which gradually solidified into a colorless solid. $C_{15}H_{24}FN_3O_6S$ (393.43 g/mol). ^{1}H NMR (400 MHz, $CDCl_3$): δ 7.95 (d, J = 0.9 Hz, 1H), 7.20 (s, 1H), 5.41 (d, J = 7.7 Hz, 1H), 4.49 (dd, J = 12.6, 6.0 Hz, 1H), 3.21 – 2.92 (m, 2H), 1.43 (s, 18H). $^{13}C\{^{1}H\}$ NMR (101 MHz, $CDCl_3$): δ 170.54,

155.44, 141.97, 136.81, 115.37, 82.49, 80.02, 53.12, 30.98, 28.40, 28.07. ^{19}F NMR (376 MHz, $CDCl_3$): δ 59.16. ESI-MS (positive mode) *m/z*: $[M+H]^+$: 394.11.

^{1}H NMR

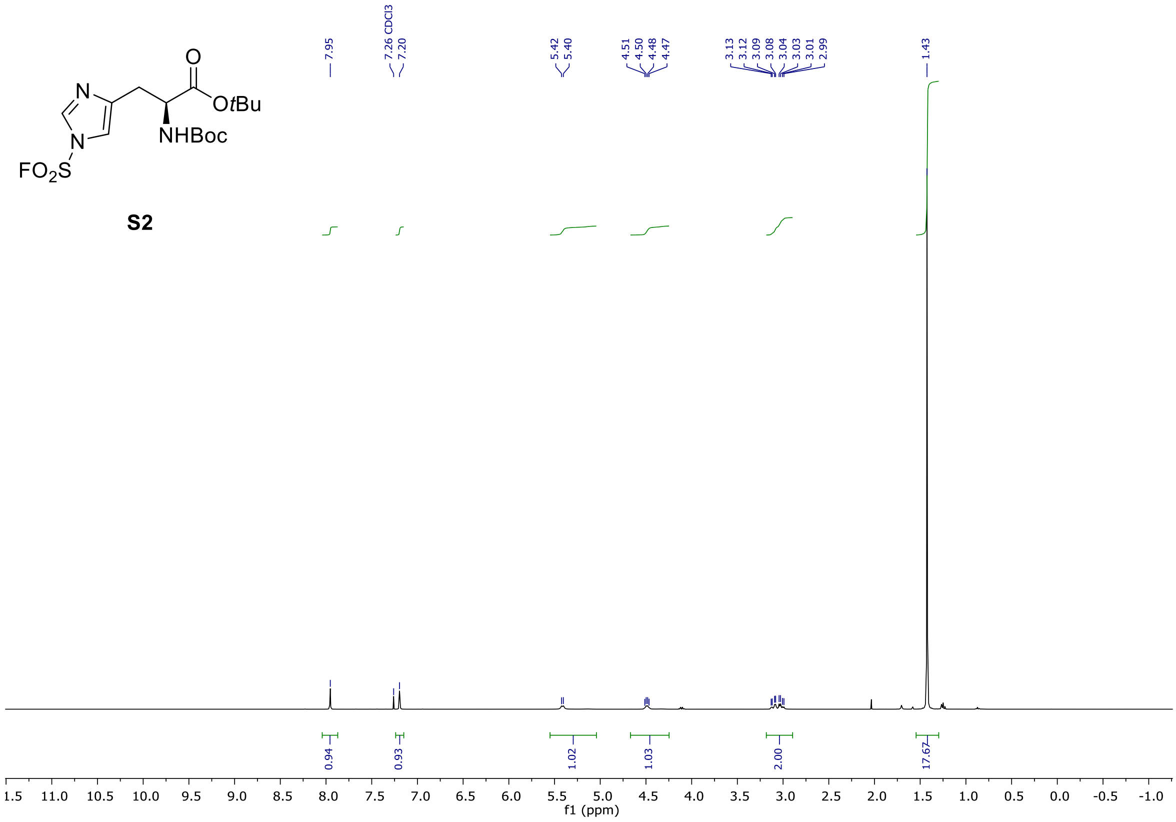

S2

$^{13}C\{^{1}H\}$ NMR

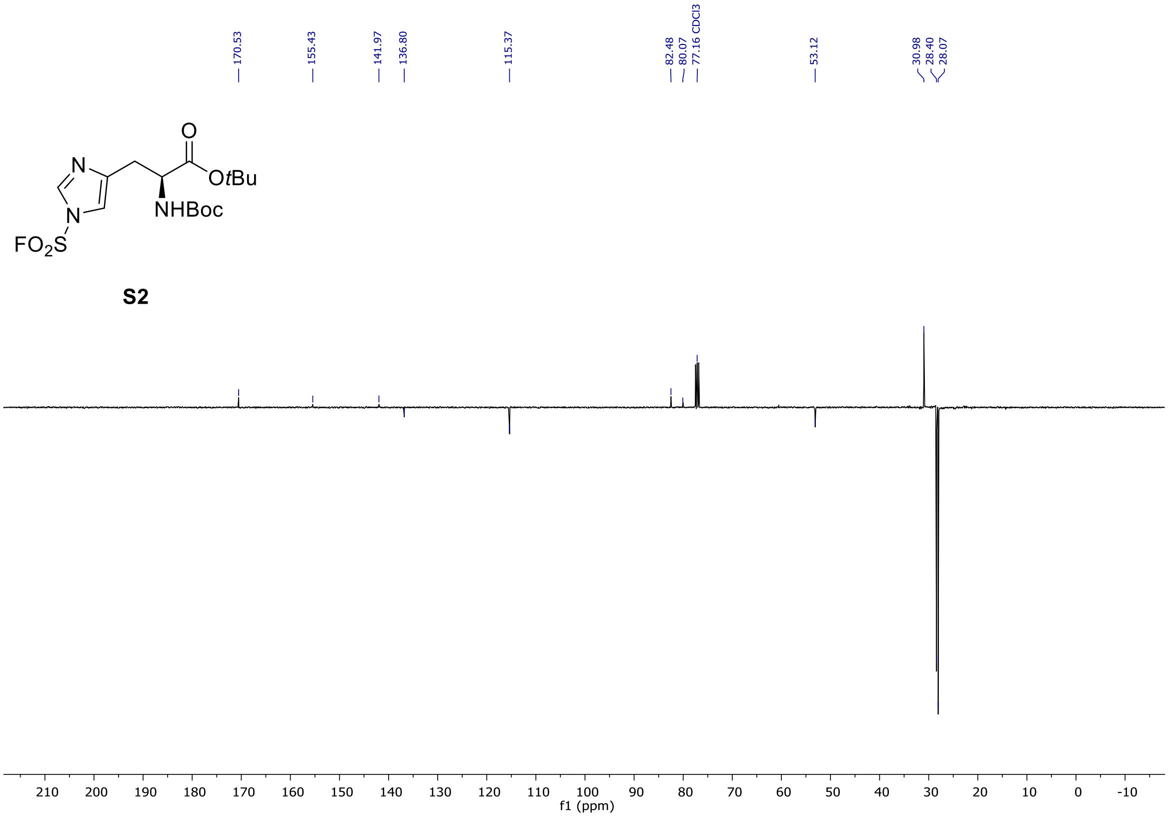

^{19}F NMR

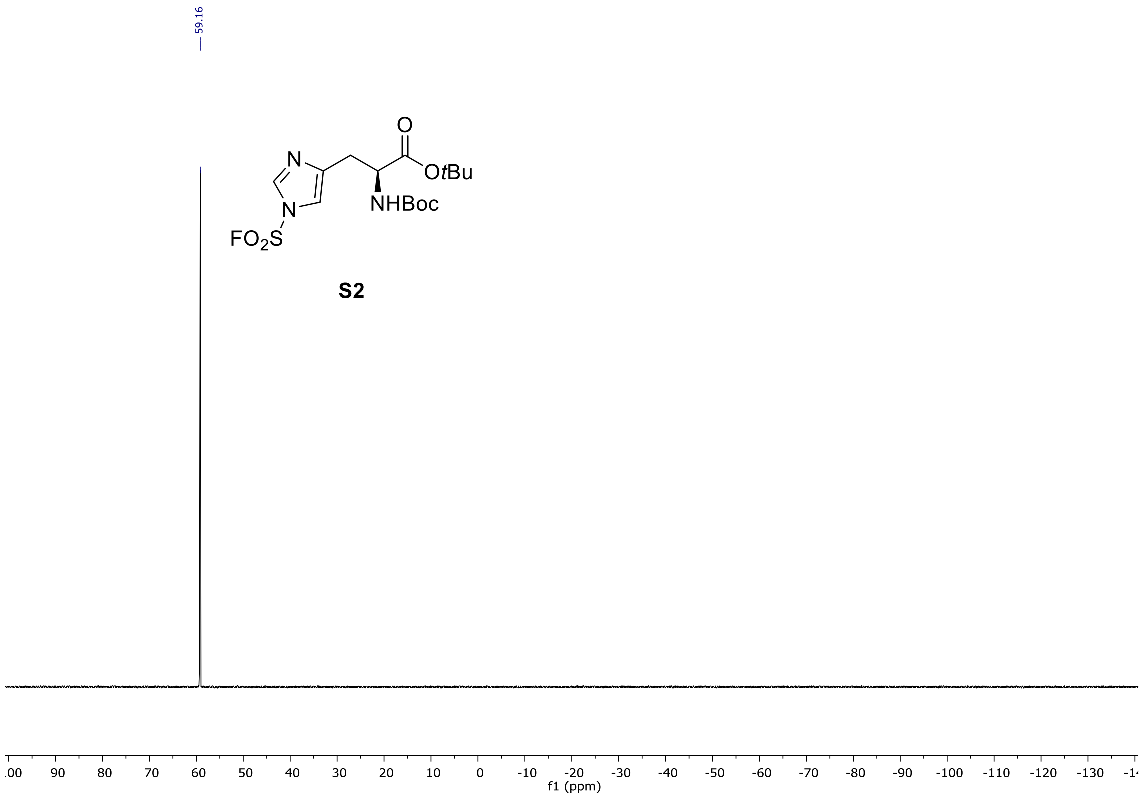

Deprotection of S2

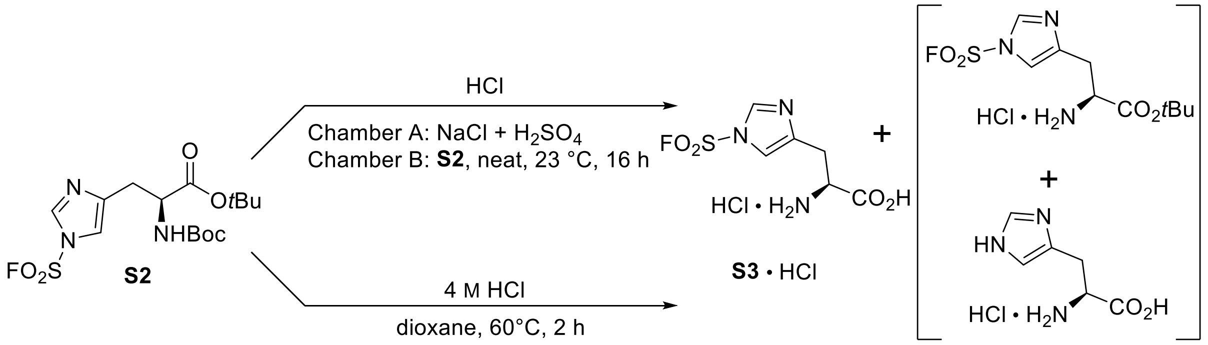

Figure S1: Attempts for preparation of N^{τ}-(fluorosulfonyl)-L-histidine hydrochloride (**S3**·HCl) via acidic deprotection of **S2**.

Deprotection of **S2** was attempted using two different approaches. Treatment with 4 M HCl in dioxane at 60 °C resulted in only partial deprotection after 2 h and was accompanied by noticeable degradation to His. Consequently, this reaction was not pursued further.

An alternative method employing *in situ* HCl generation in a two-chamber reactor, as described by Verschueren et al.[2], at room temperature led to complete removal of Boc and *tert*-butyl protecting groups. However, this approach also resulted in substantial formation of His.

The formation of His under strongly acidic conditions can be attributed to protonation of the imidazole moiety and generation of an imidazolium species, which activates the fluorosulfuryl substituent toward cleavage. As a result, the S–N linkage becomes labile, leading to loss of the fluorosulfuryl group. This side reaction competes with the intended deprotection and accounts for the substantial formation of His.

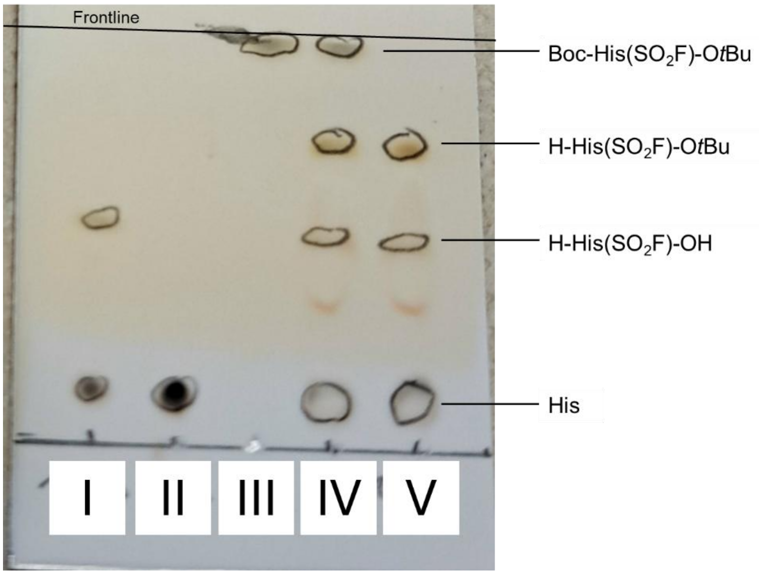

Figure S2: Combined TLC for attempted preparation of N^{τ}-(fluorosulfonyl)-L-histidine hydrochloride (**S3**·HCl) via acidic deprotection of **S2**. Eluent: *n*BuOH:AcOH:H_2O (3:1:1). **I**: Reaction mixture from attempted deprotection with *in situ* generated gaseous HCl, **II**: His, **III**: **S3**, **IV**: Mixed: **S2** + reaction mixture from attempted deprotection with 4 M HCl in dioxane. **V**: reaction mixture from attempted deprotection with 4 M HCl in dioxane. Ninhydrin spray (0.5% in *n*BuOH) was used for staining.

NMR spectra

N_{α}-(*tert*-Butoxycarbonyl)-1-(fluorosulfonyl)-L-tryptophan *tert*-butyl ester (**1**)

<u>^{1}H NMR</u>

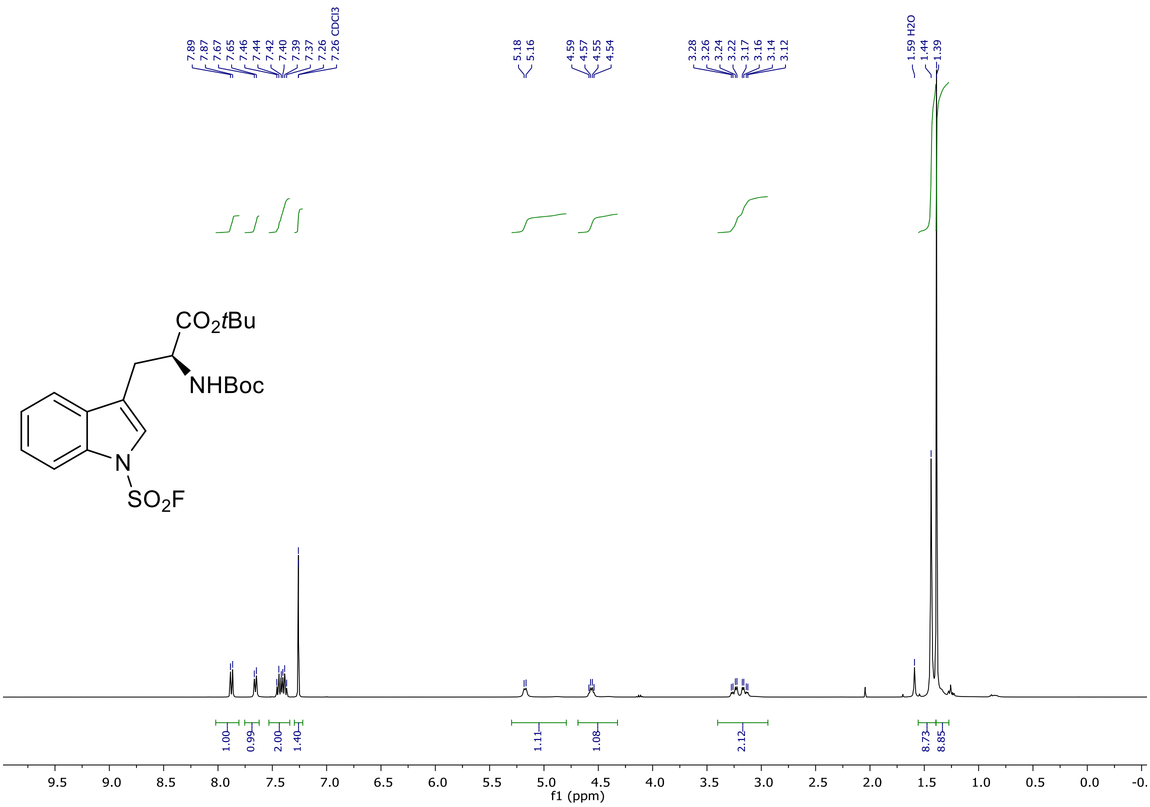

<u>^{13}C{^{1}H} NMR</u>

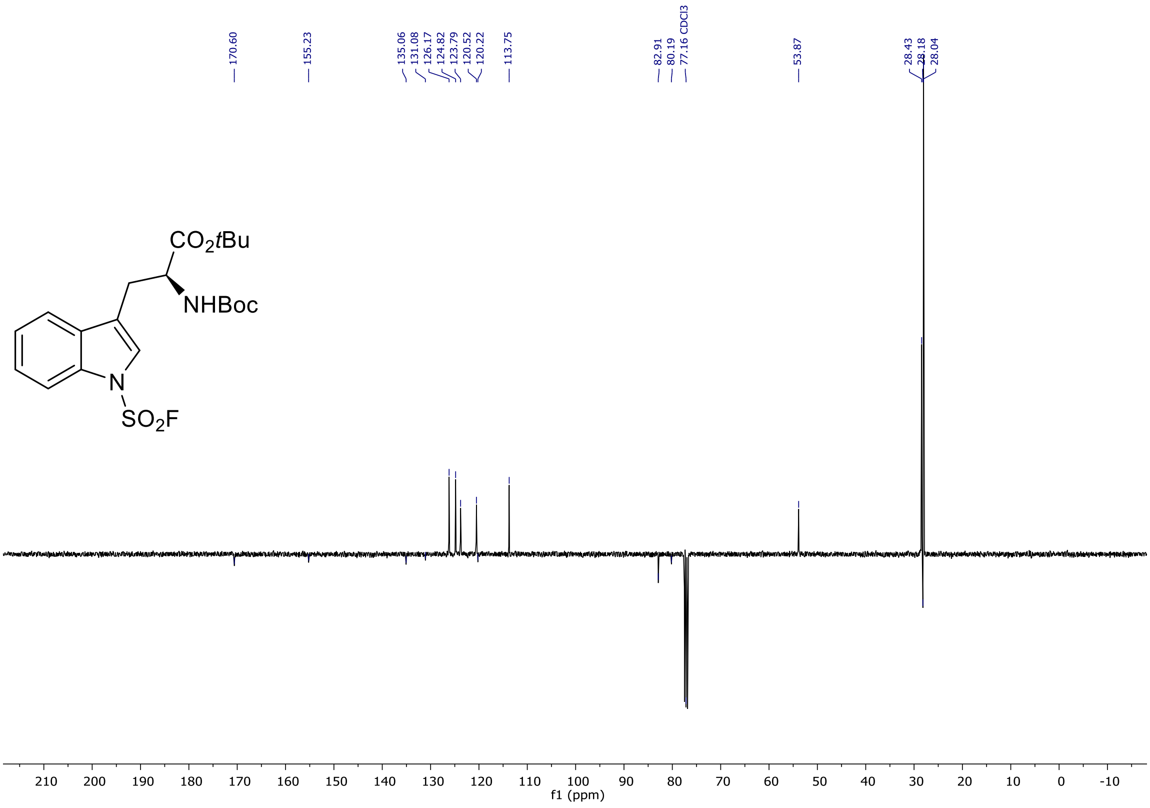

^{19}F NMR

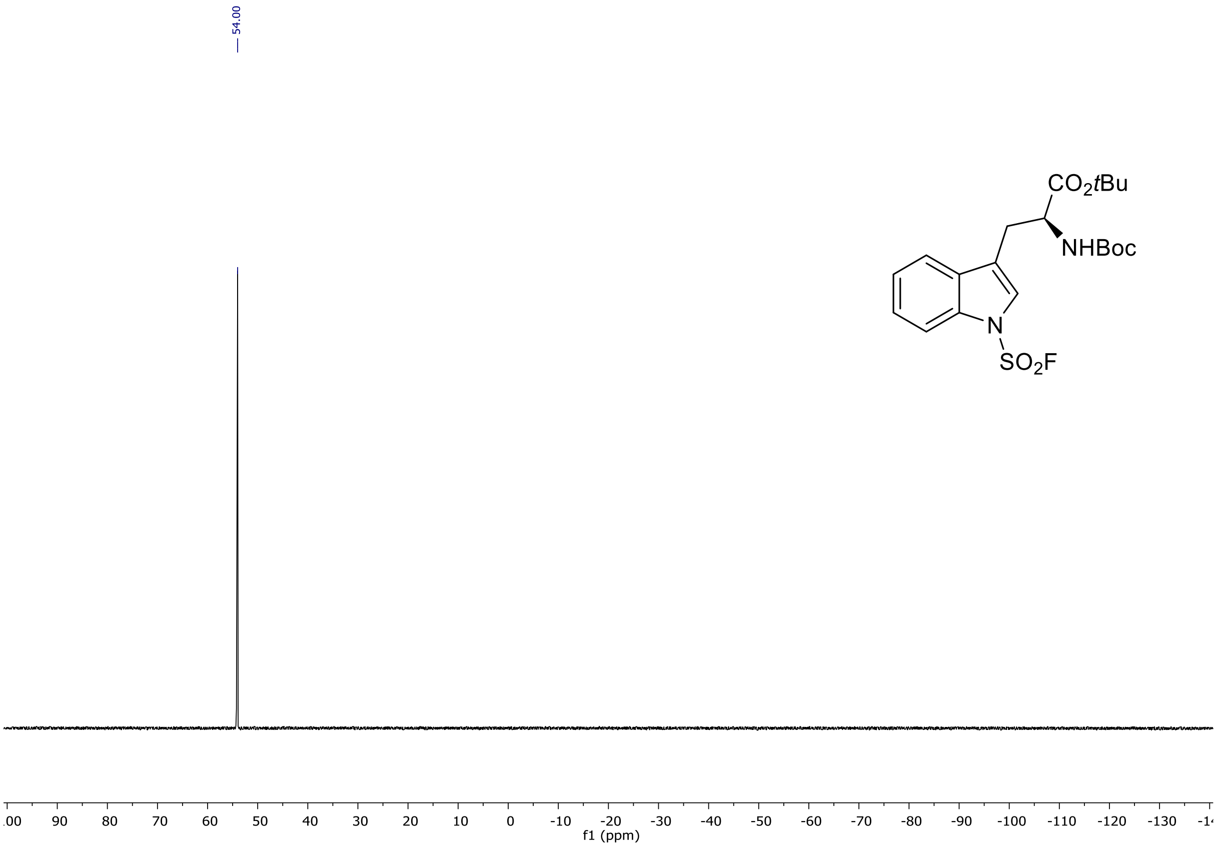

54.00
CO2tBu
NHBoc
N
SO2F
00
90
80
70
60
50
40
30
20
10
0
-10
-20
-30
-40
-50
-60
-70
-80
-90
-100
-110
-120
-130
f1 (ppm)

Methyl N_{α}-(*tert*-butoxycarbonyl)-1-(fluorosulfonyl)-L-tryptophan (**2**)

^{1}H NMR

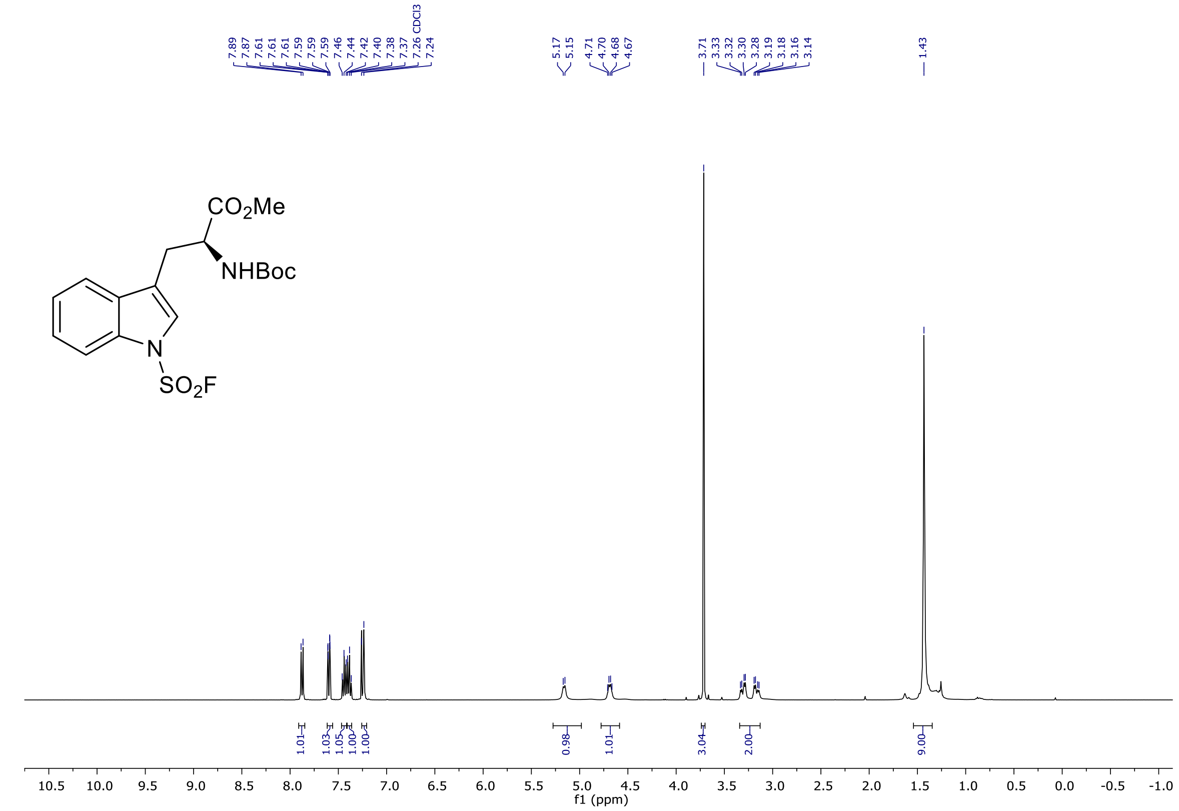

^{13}C{^{1}H} NMR

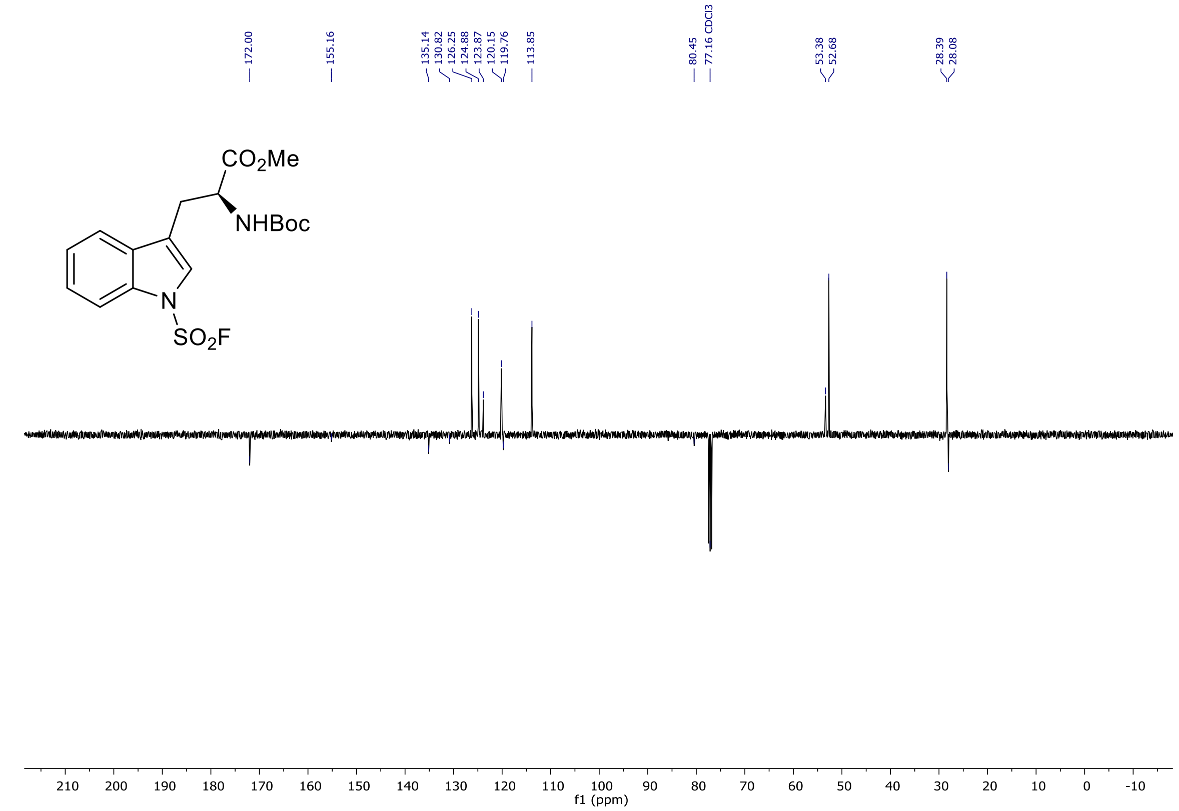

^{19}F NMR

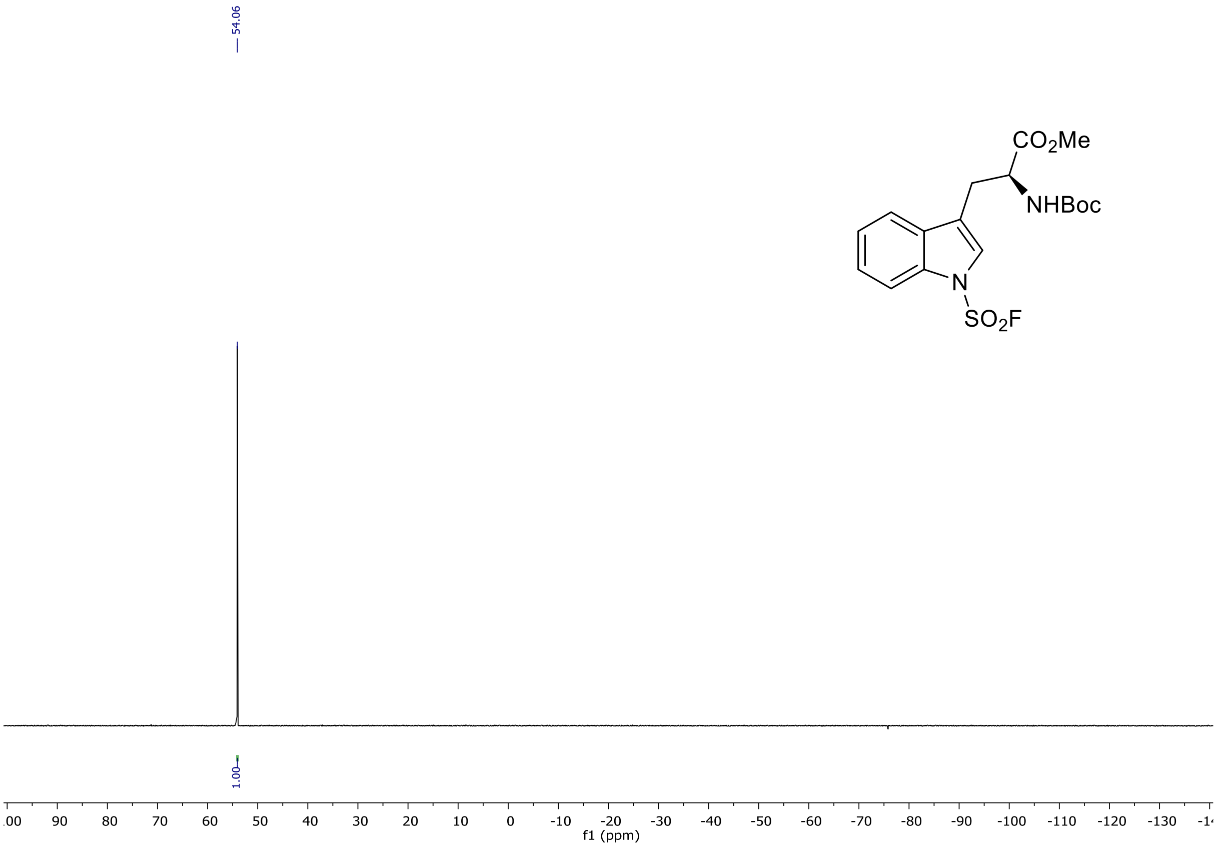

54.06
CO2Me
NHBoc
N
SO2F
1.00
00 90 80 70 60 50 40 30 20 10 0 -10 -20 -30 -40 -50 -60 -70 -80 -90 -100 -110 -120 -130
f1 (ppm)

4-Phenylpiperidine-1-sulfonyl fluoride (**4**)

<u>^{1}H NMR</u>

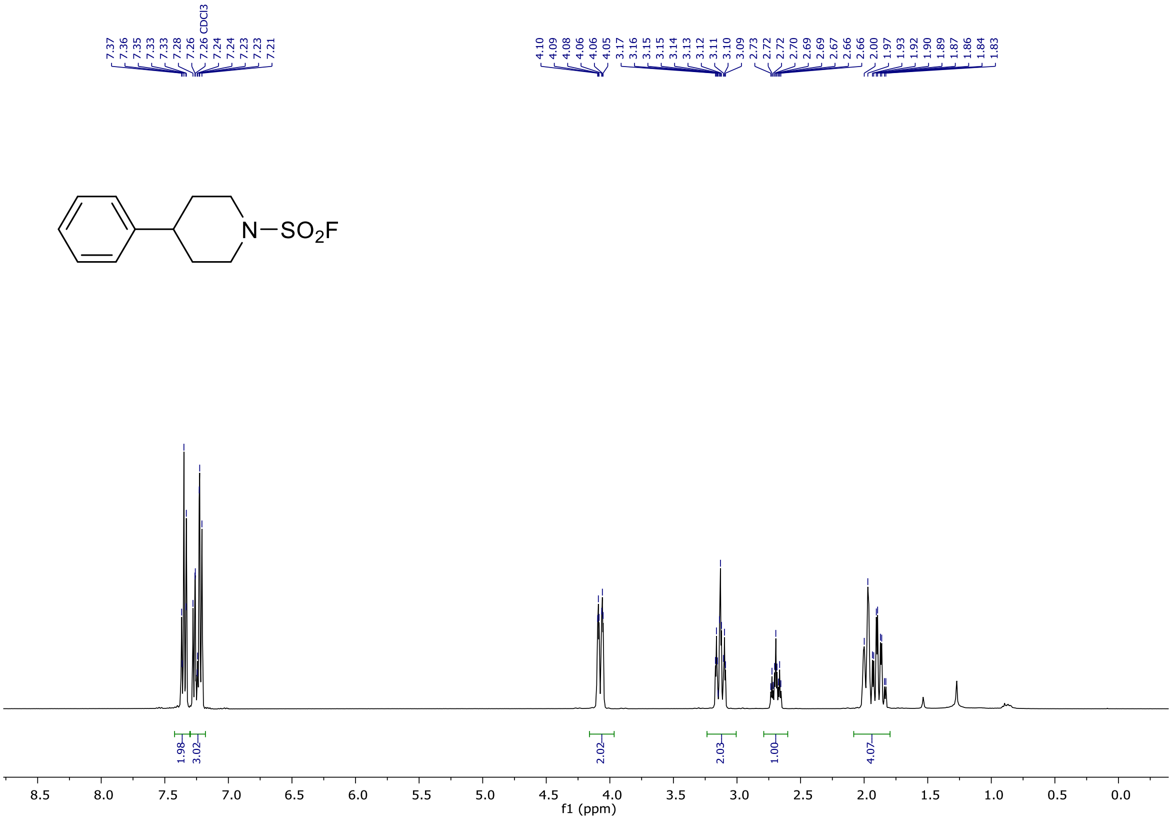

<u>^{13}C{^{1}H} NMR</u>

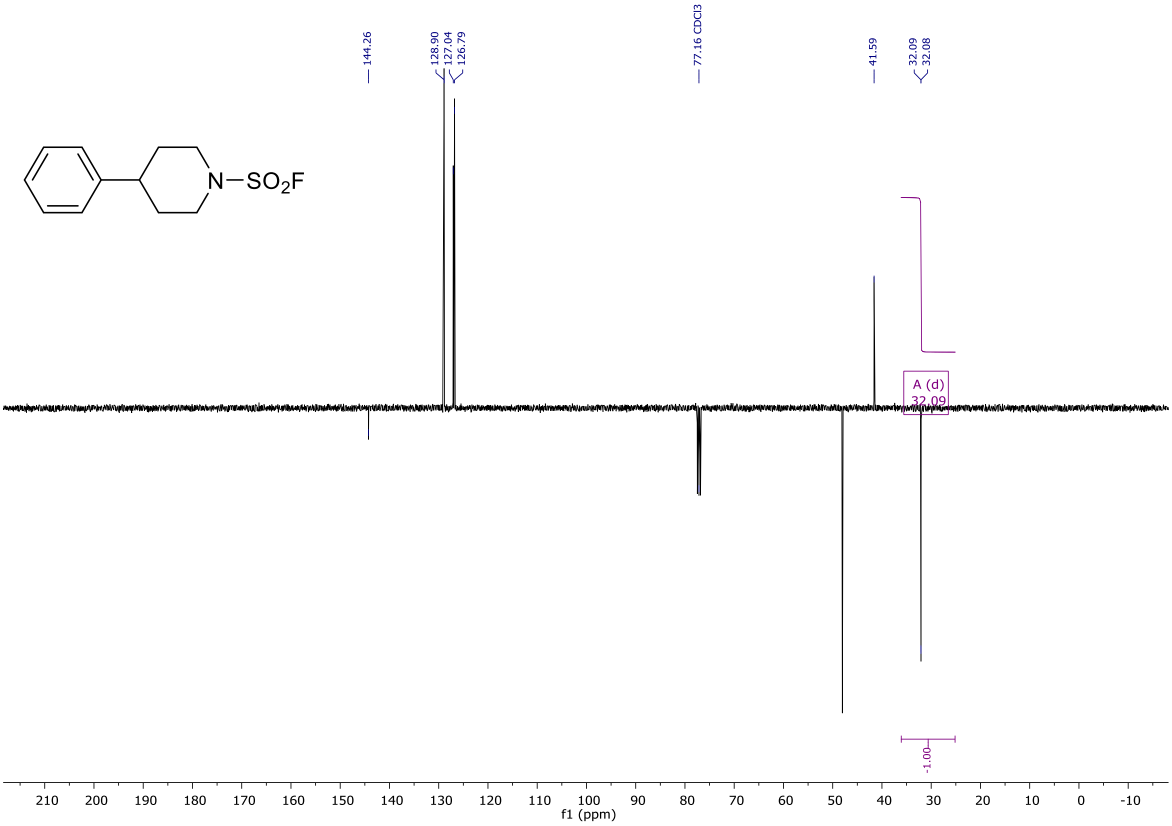

^{19}F NMR

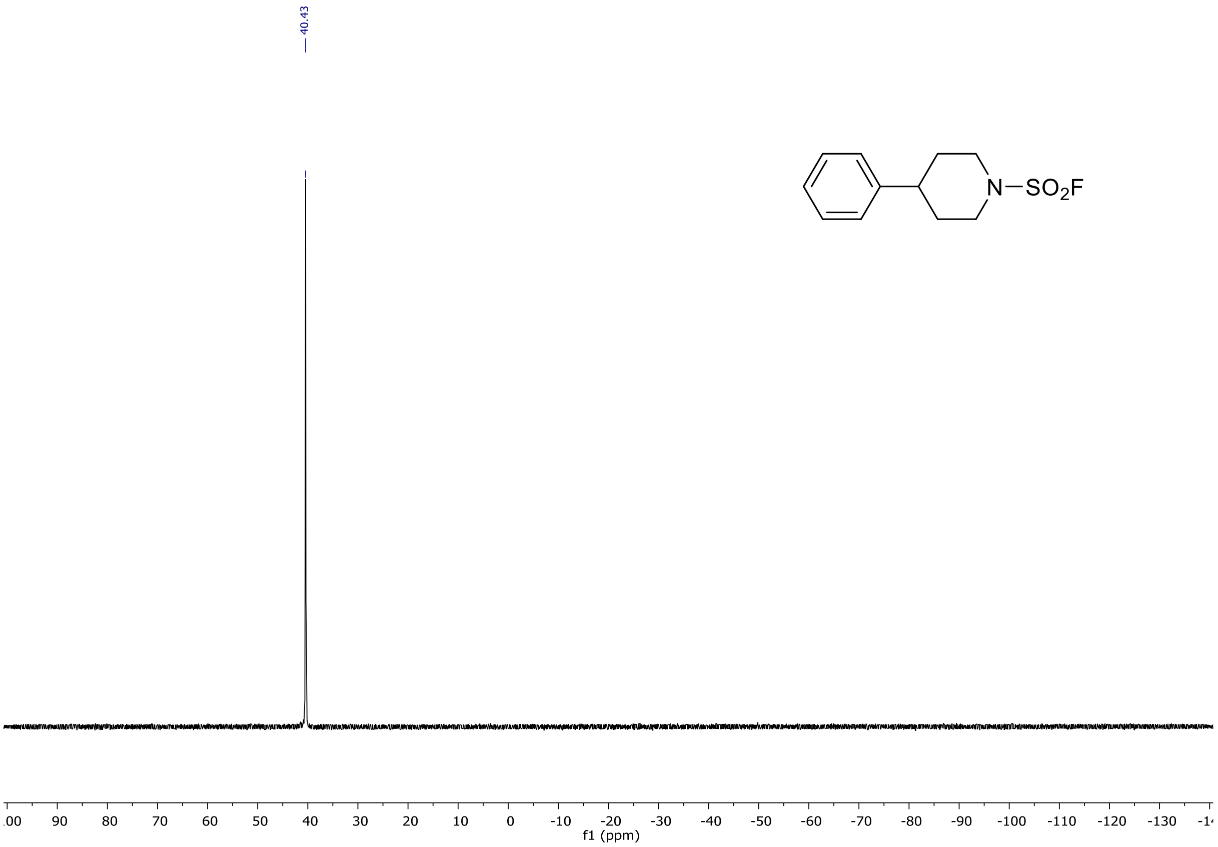

40.43
N−SO2F
.00 90 80 70 60 50 40 30 20 10 0 -10 -20 -30 -40 -50 -60 -70 -80 -90 -100 -110 -120 -130
f1 (ppm)

9*H*-Carbazole-9-sulfonyl fluoride (**5**)

^{1}H NMR

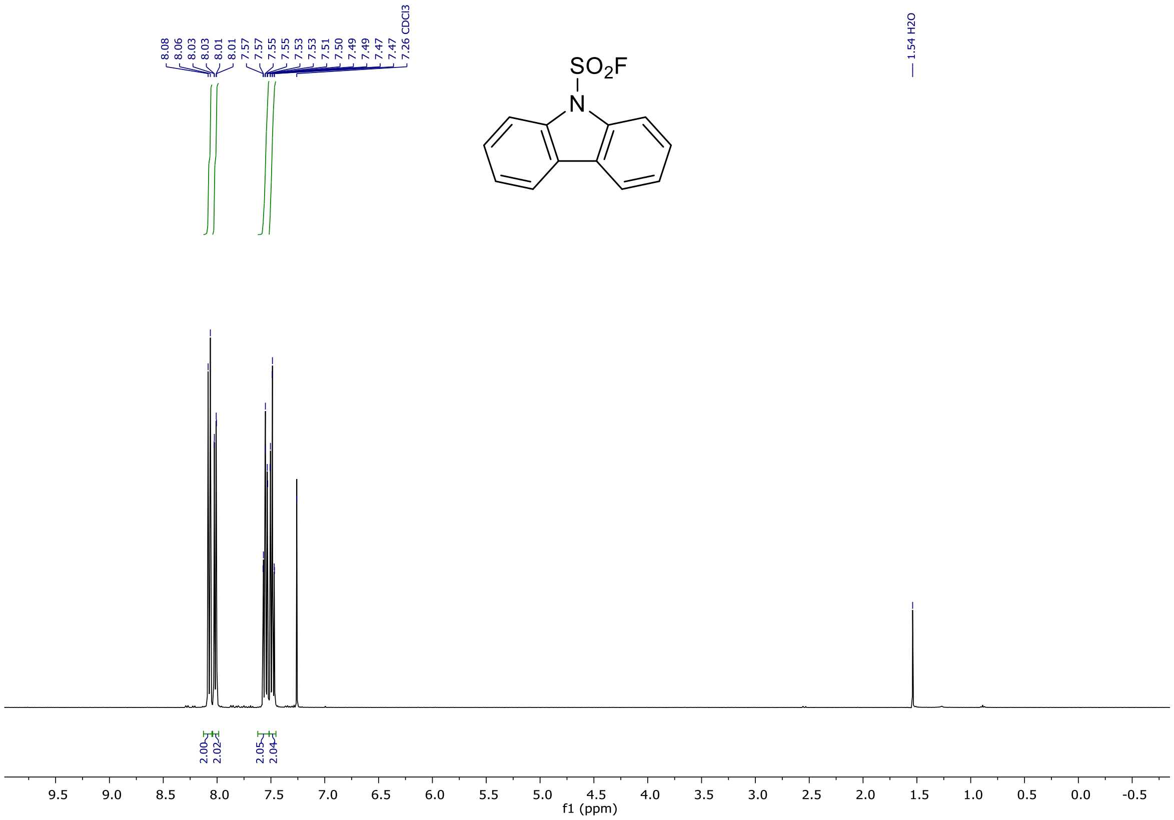

^{13}C{^{1}H} NMR

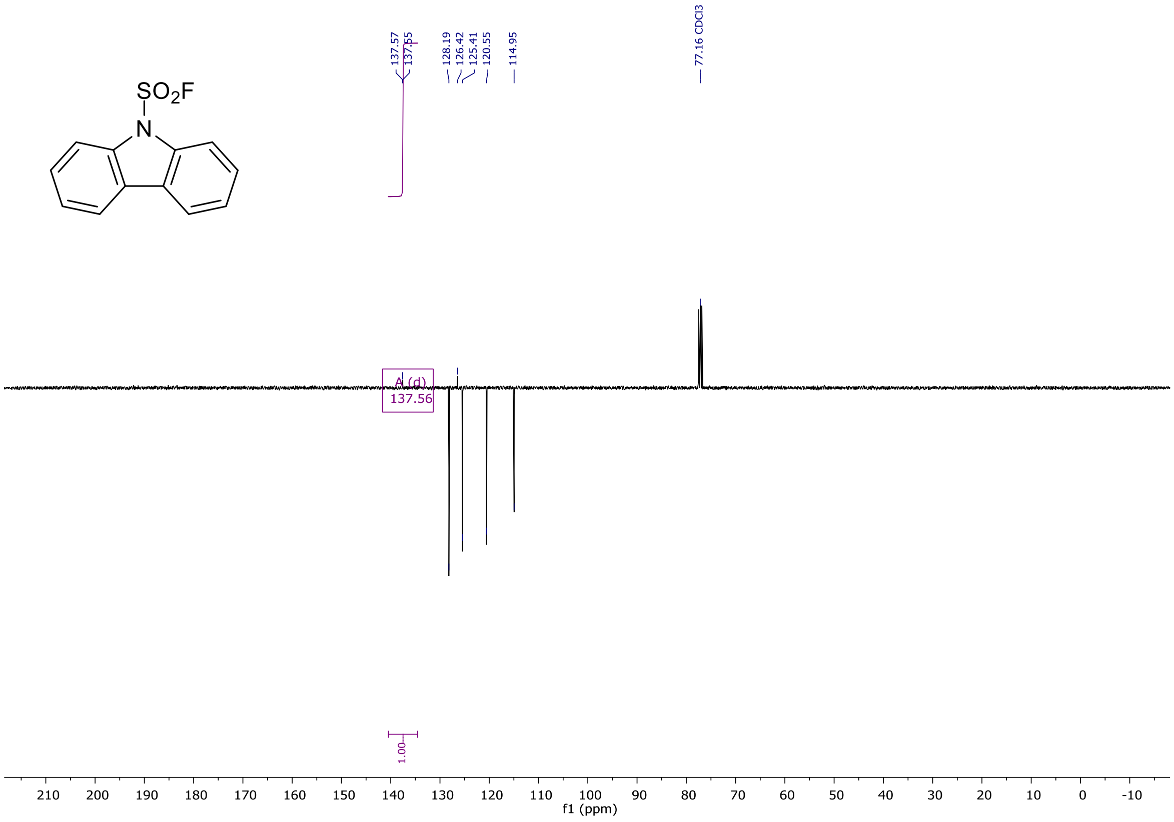

^{19}F NMR

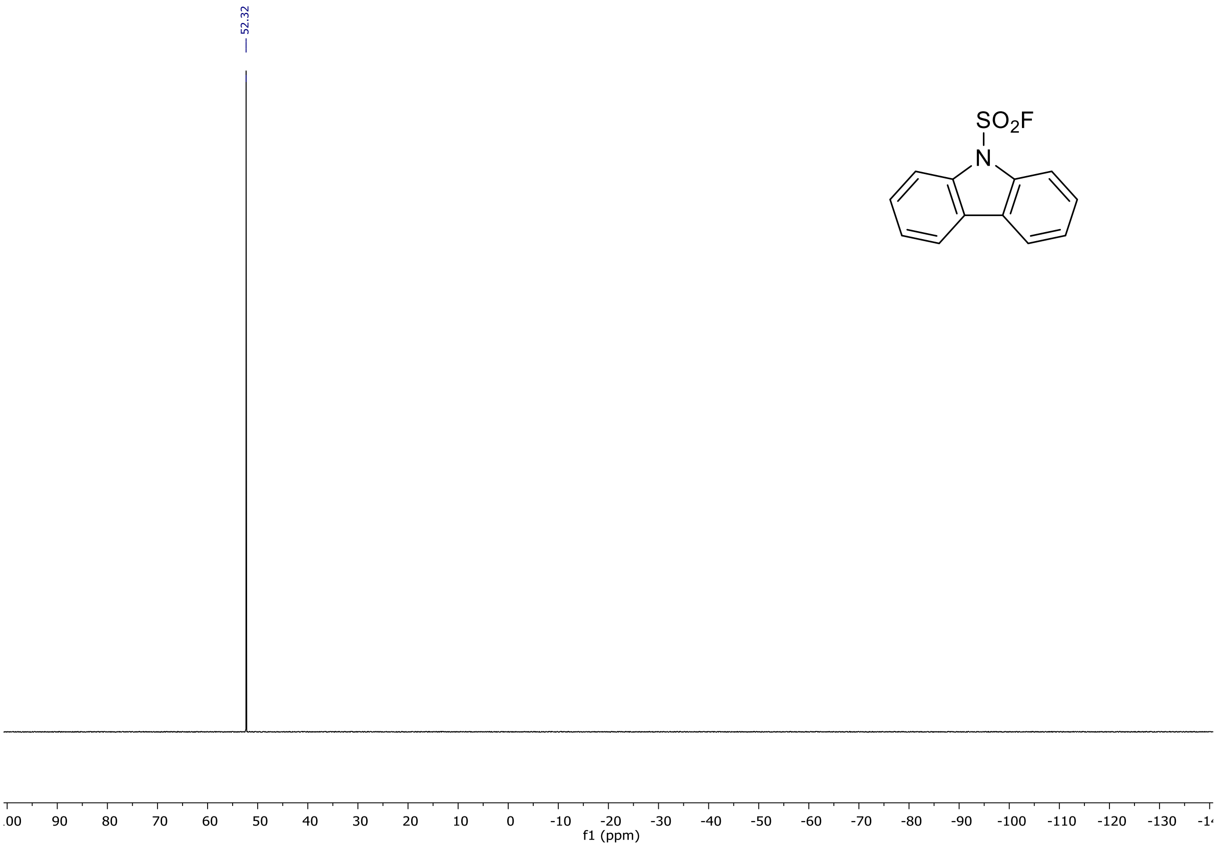

52.32
SO2F
N
.00 90 80 70 60 50 40 30 20 10 0 -10 -20 -30 -40 -50 -60 -70 -80 -90 -100 -110 -120 -130
f1 (ppm)

5-Methoxy-1*H*-indole-1-sulfonyl fluoride (**6**)

^{1}H NMR

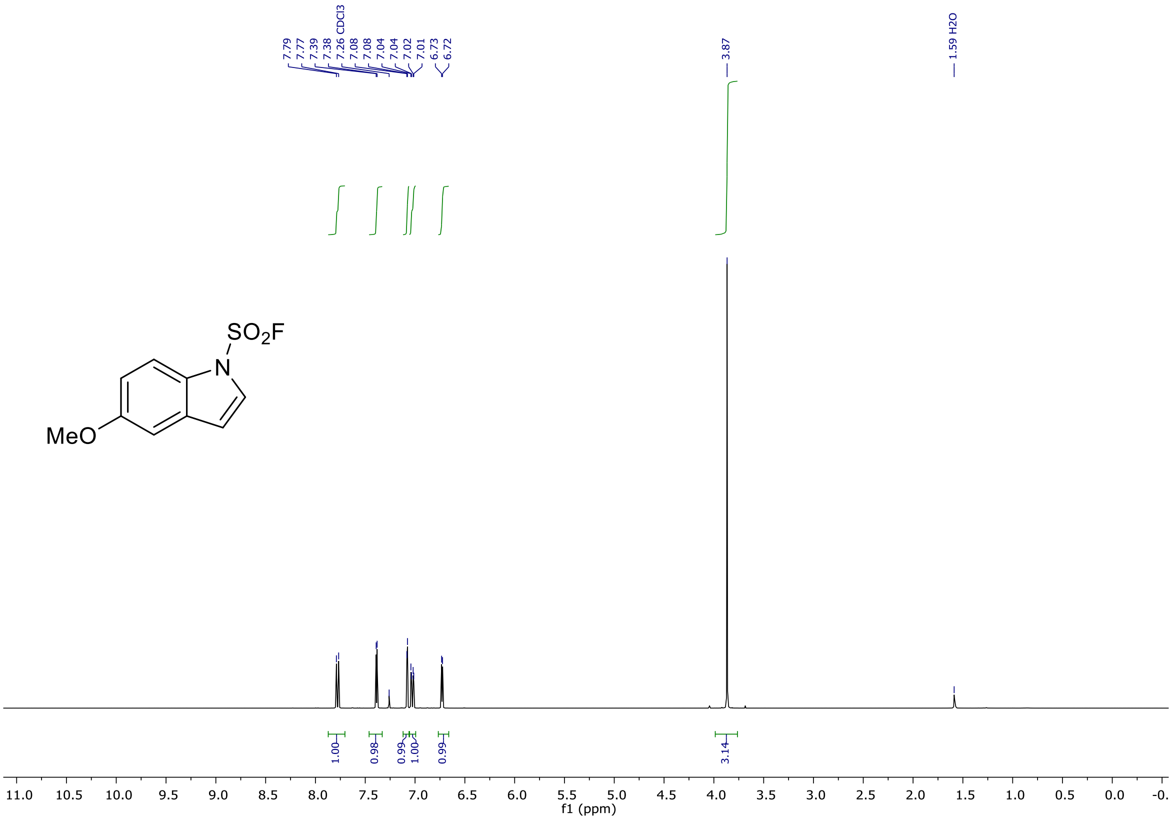

^{13}C{^{1}H} NMR

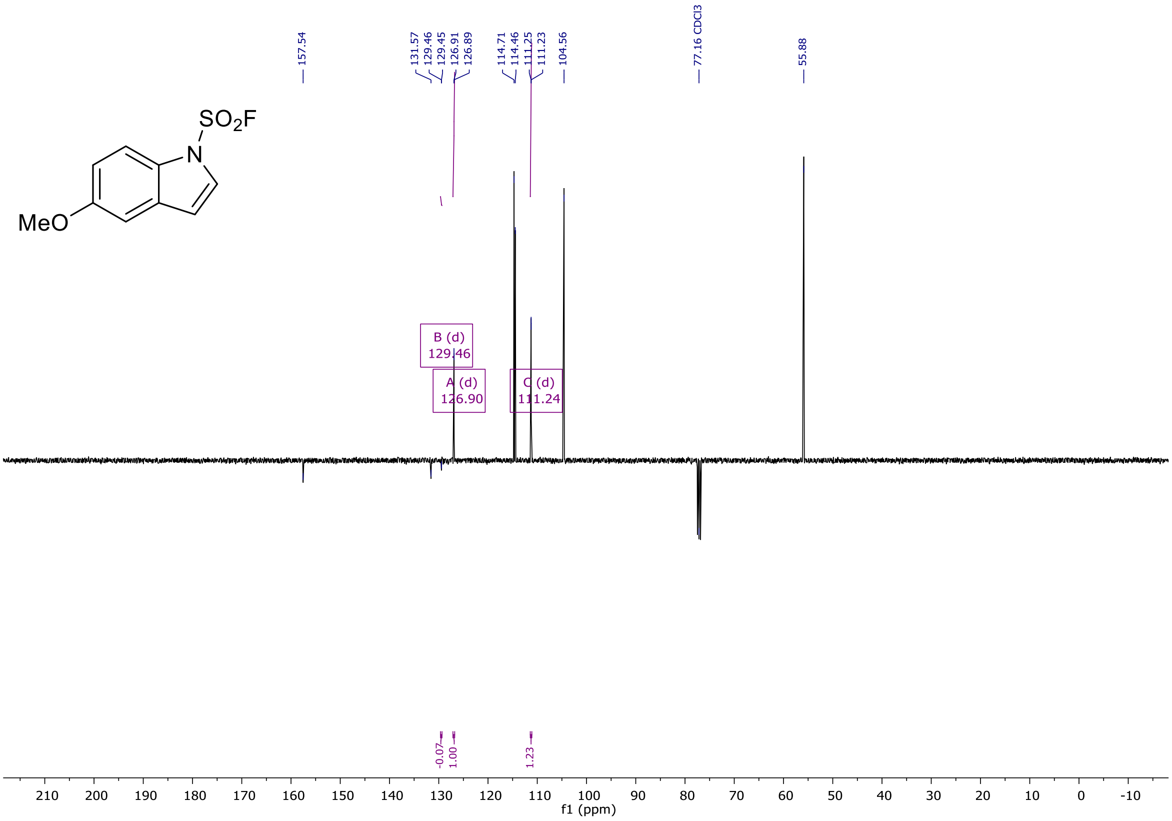

^{19}F NMR

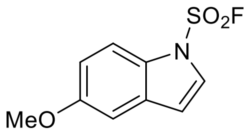

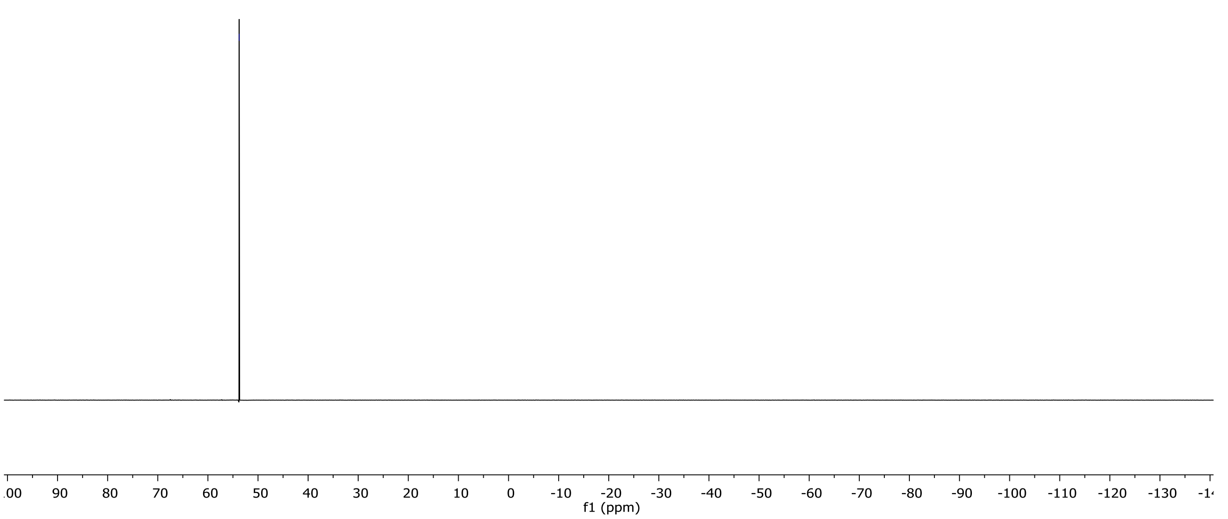

5-Formyl-1*H*-indole-1-sulfonyl fluoride (**7**)

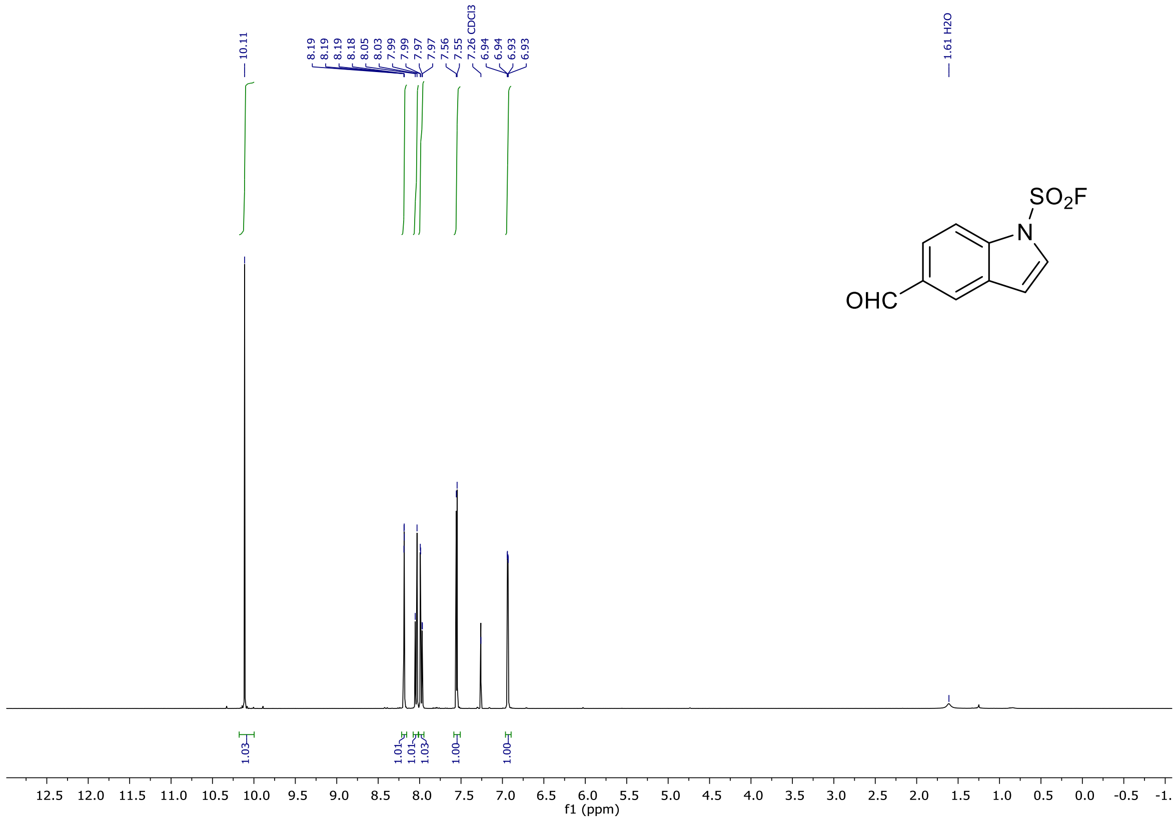

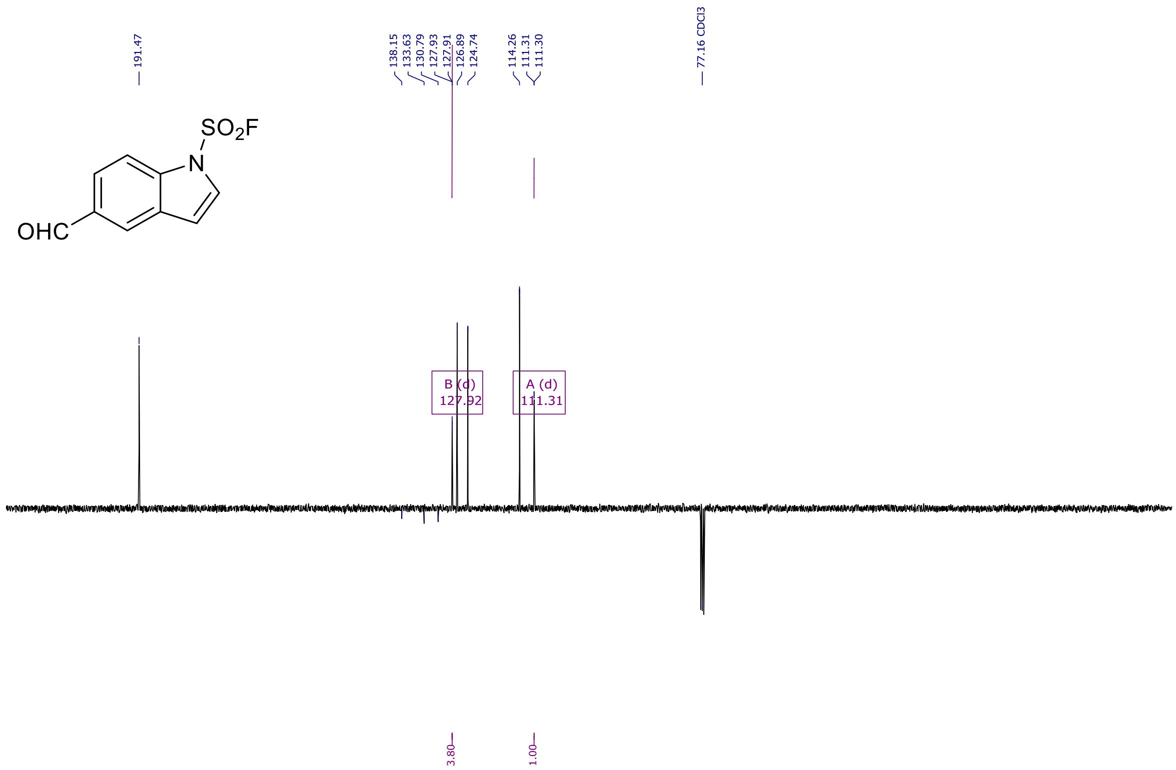

^{19}F NMR

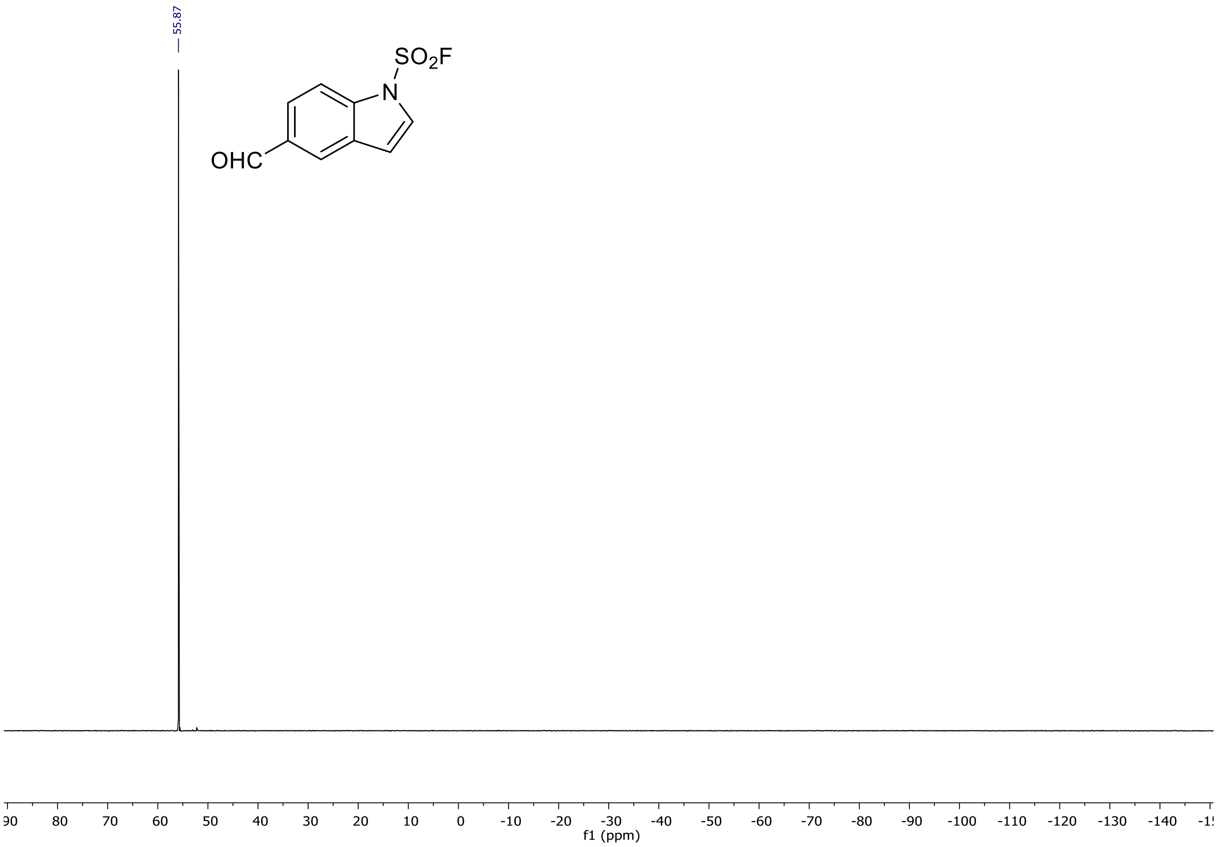

55.87
SO2F
N
OHC
80
70
60
50
40
30
20
10
0
-10
-20
-30
-40
-50
-60
-70
-80
-90
-100
-110
-120
-130
-140
f1 (ppm)

1*H*-Indole-1-sulfonyl fluoride (**8**)

^{1}H NMR

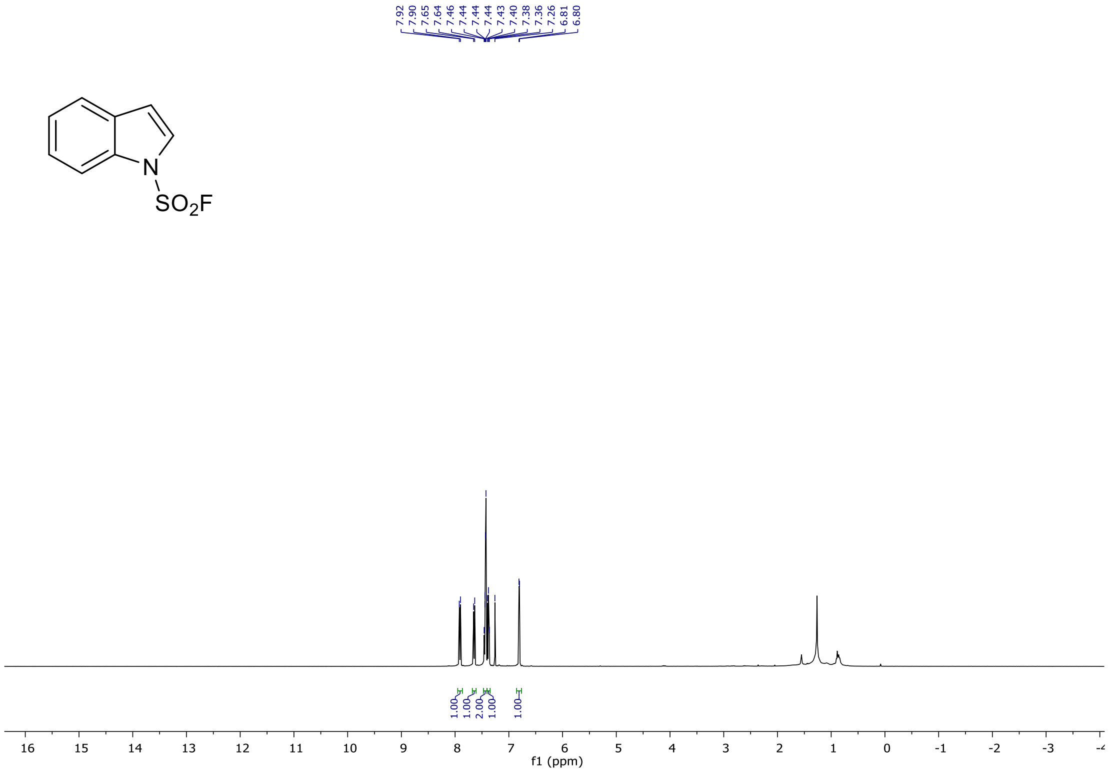

^{13}C{^{1}H} NMR

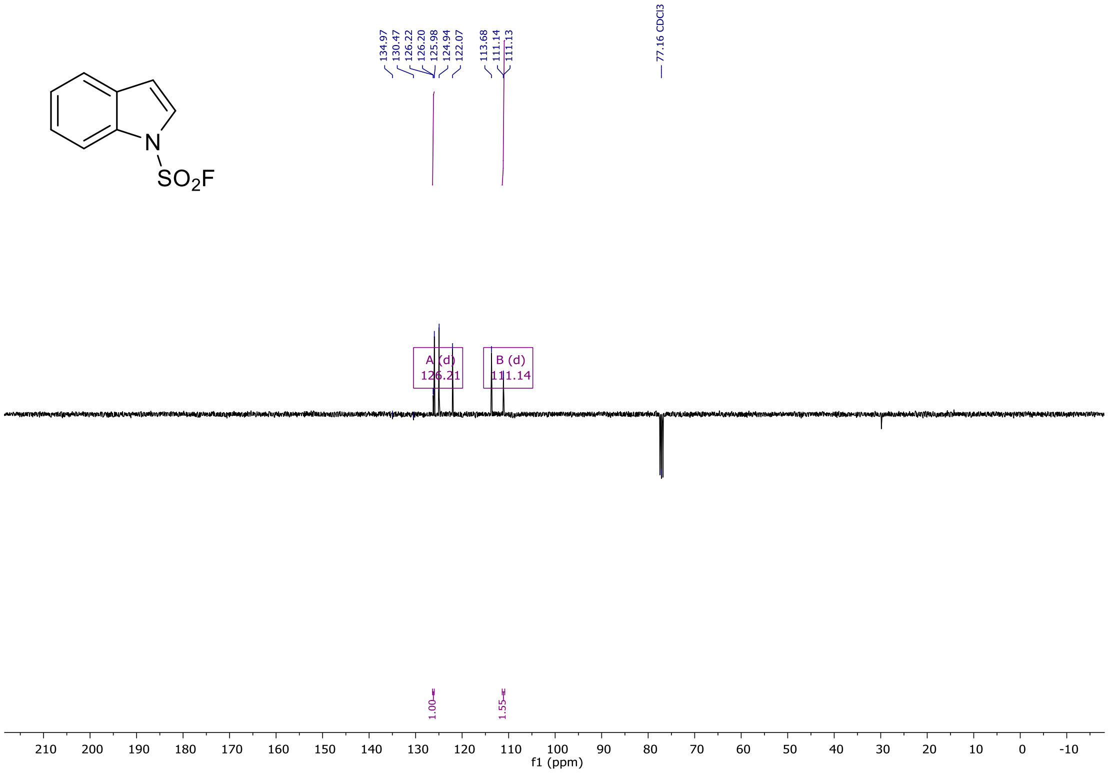

^{19}F NMR

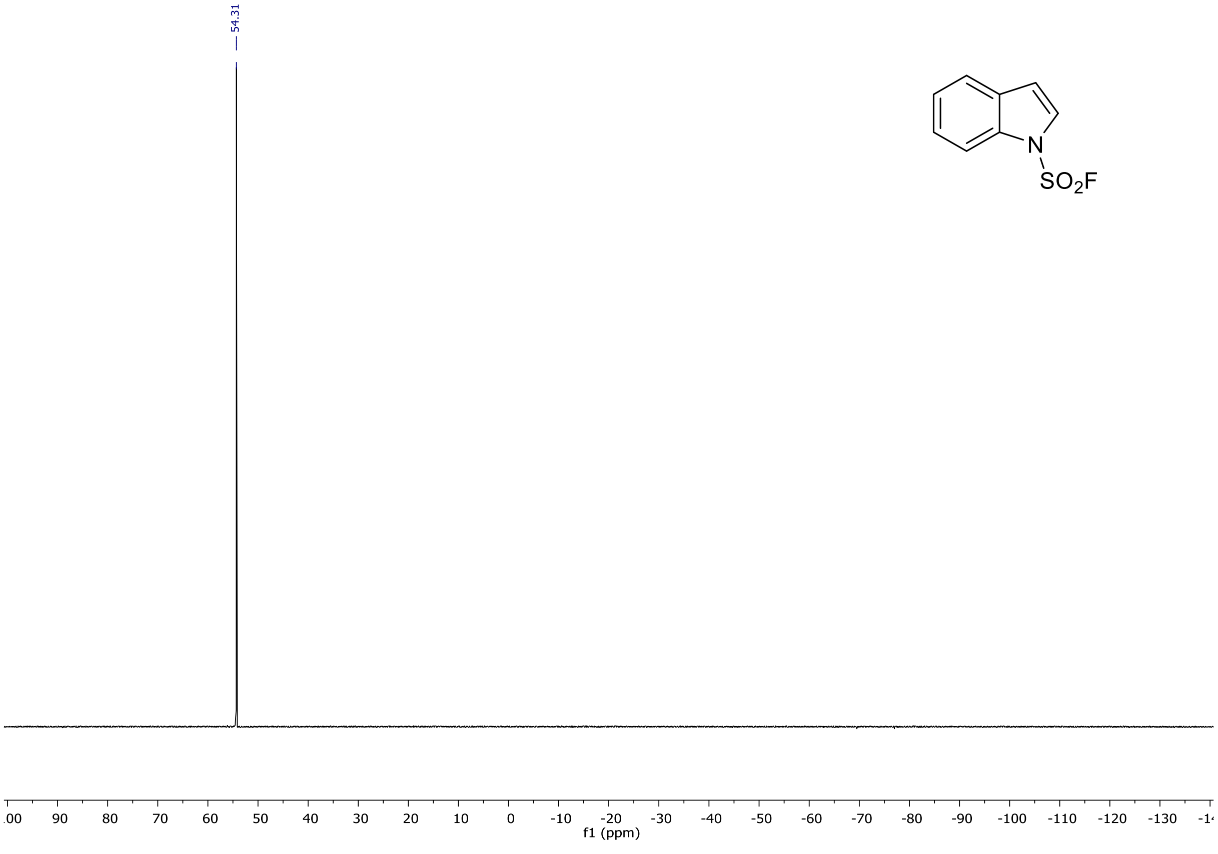

54.31
N
SO2F
.00
90
80
70
60
50
40
30
20
10
0
-10
-20
-30
-40
-50
-60
-70
-80
-90
-100
-110
-120
-130
f1 (ppm)

(4-Methoxyphenyl)(methyl)sulfamoyl fluoride (**10**)

^{1}H NMR

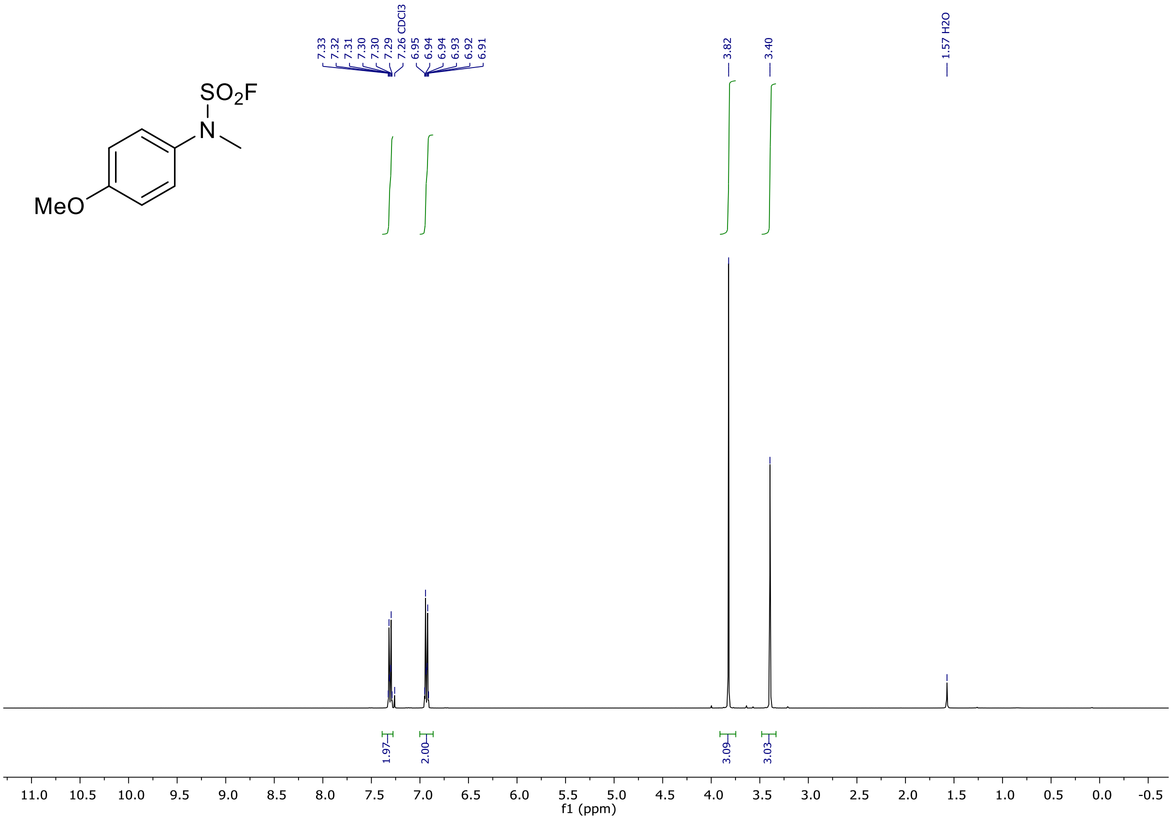

^{13}C{^{1}H} NMR

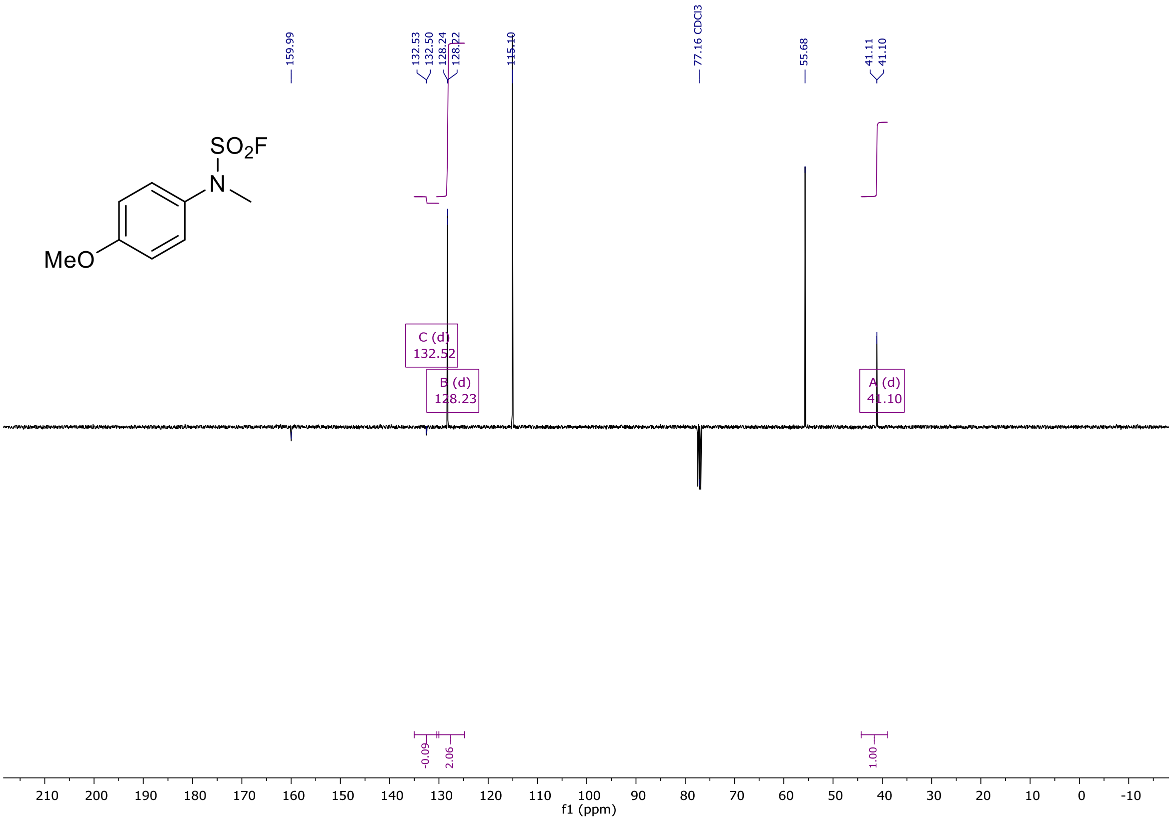

^{19}F NMR

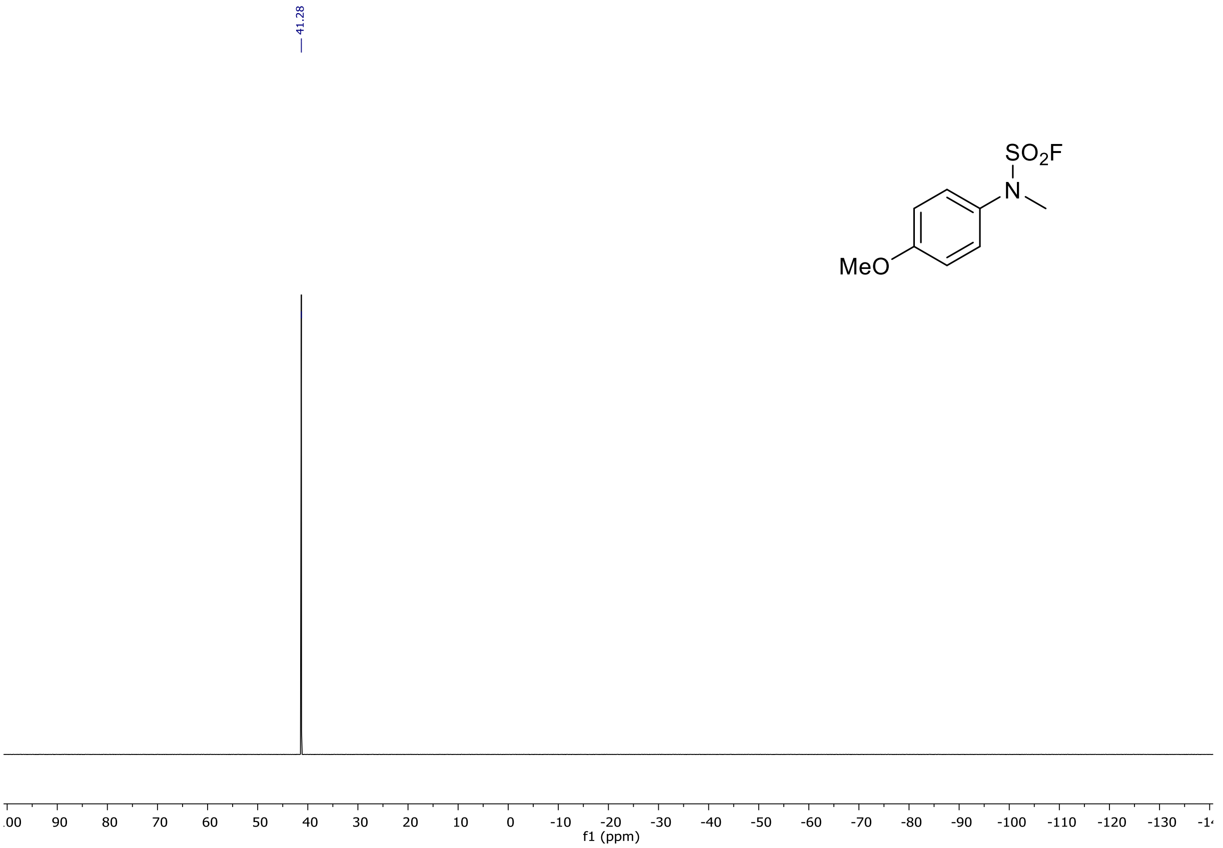

41.28
SO2F
N
MeO
.00
90
80
70
60
50
40
30
20
10
0
-10
-20
-30
-40
-50
-60
-70
-80
-90
-100
-110
-120
-130
f1 (ppm)

[4-(Hydroxymethyl)phenyl](methyl)sulfamoyl fluoride (**11**)

^{1}H NMR

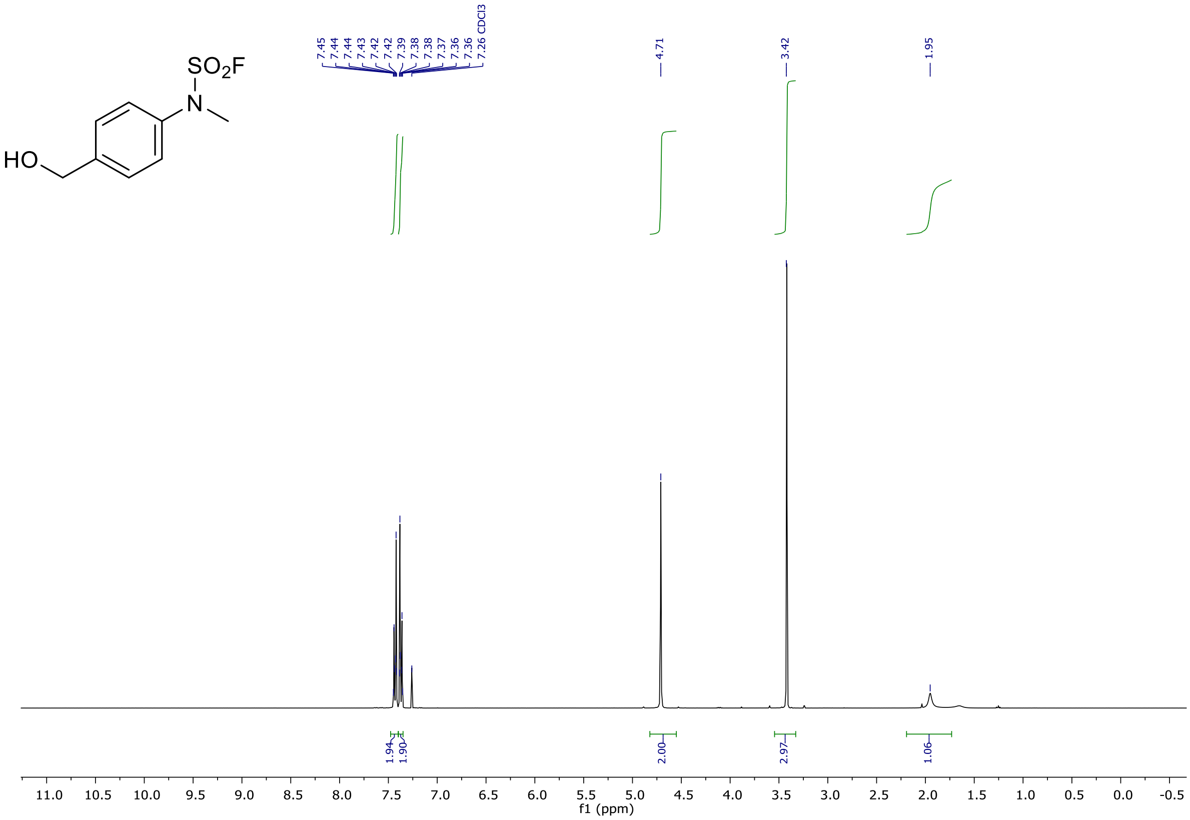

$^{13}C\{^{1}H\}$ NMR

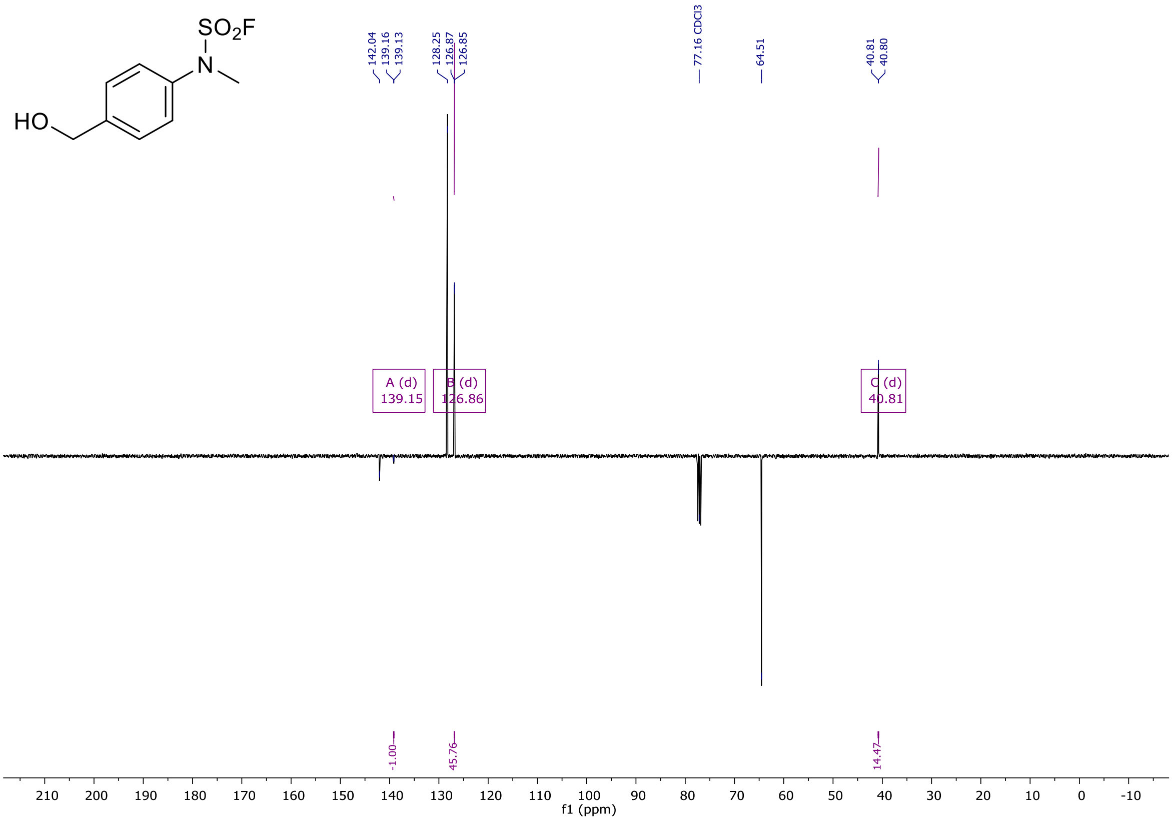

^{19}F NMR

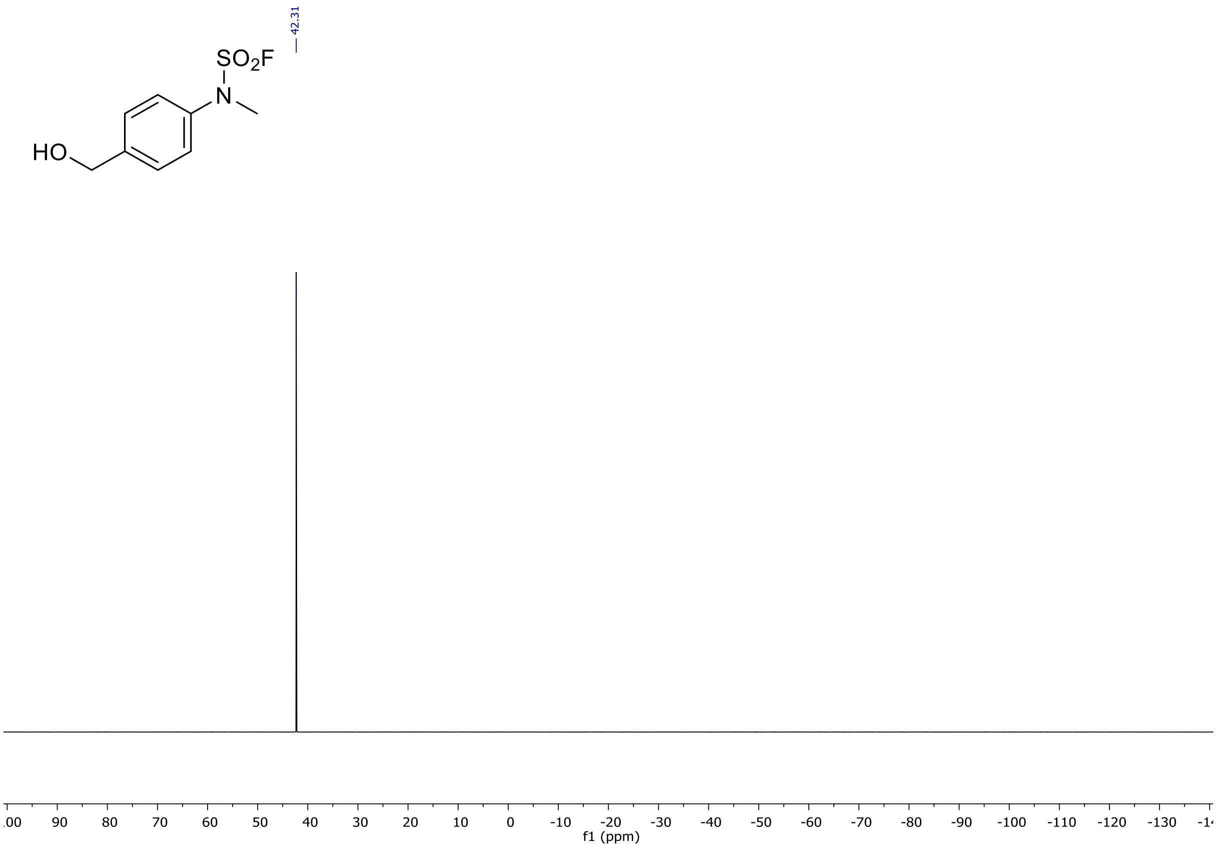

(4-Formylphenyl)(methyl)sulfamoyl fluoride (**12**)

^{1}H NMR

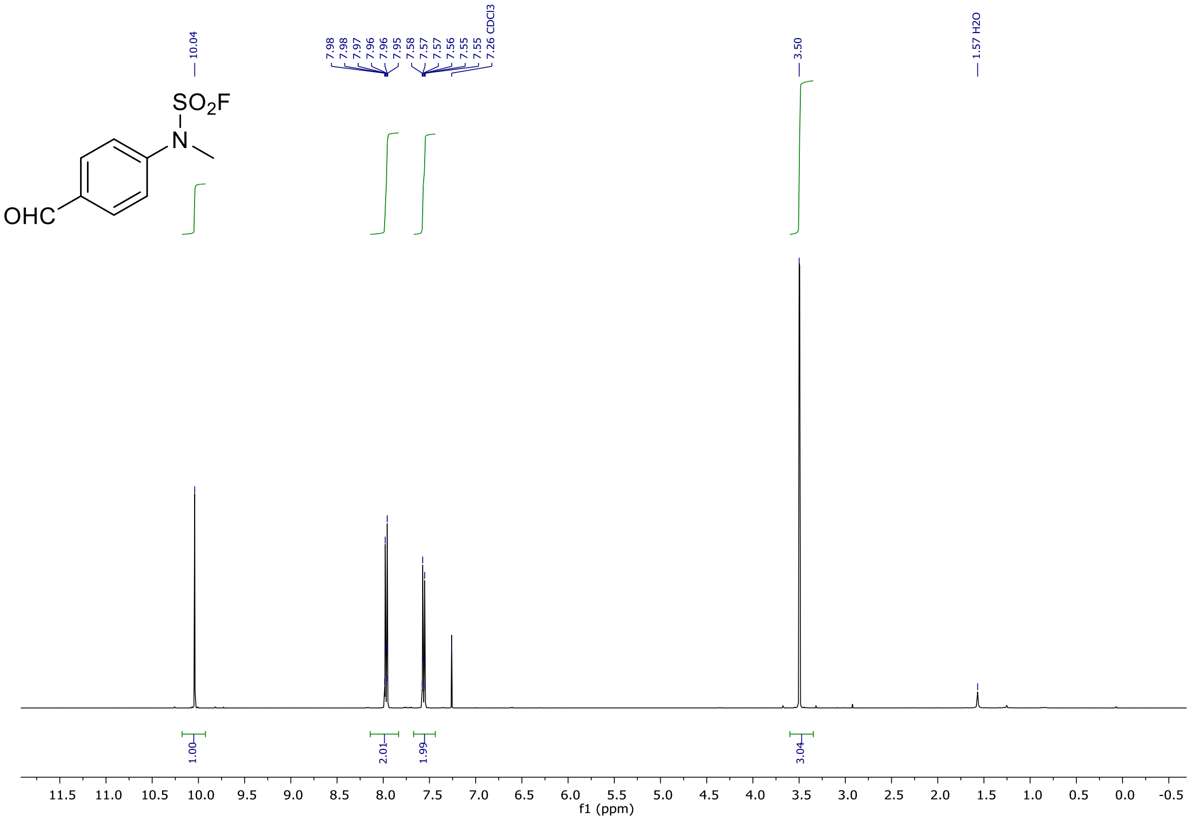

^{13}C{^{1}H} NMR

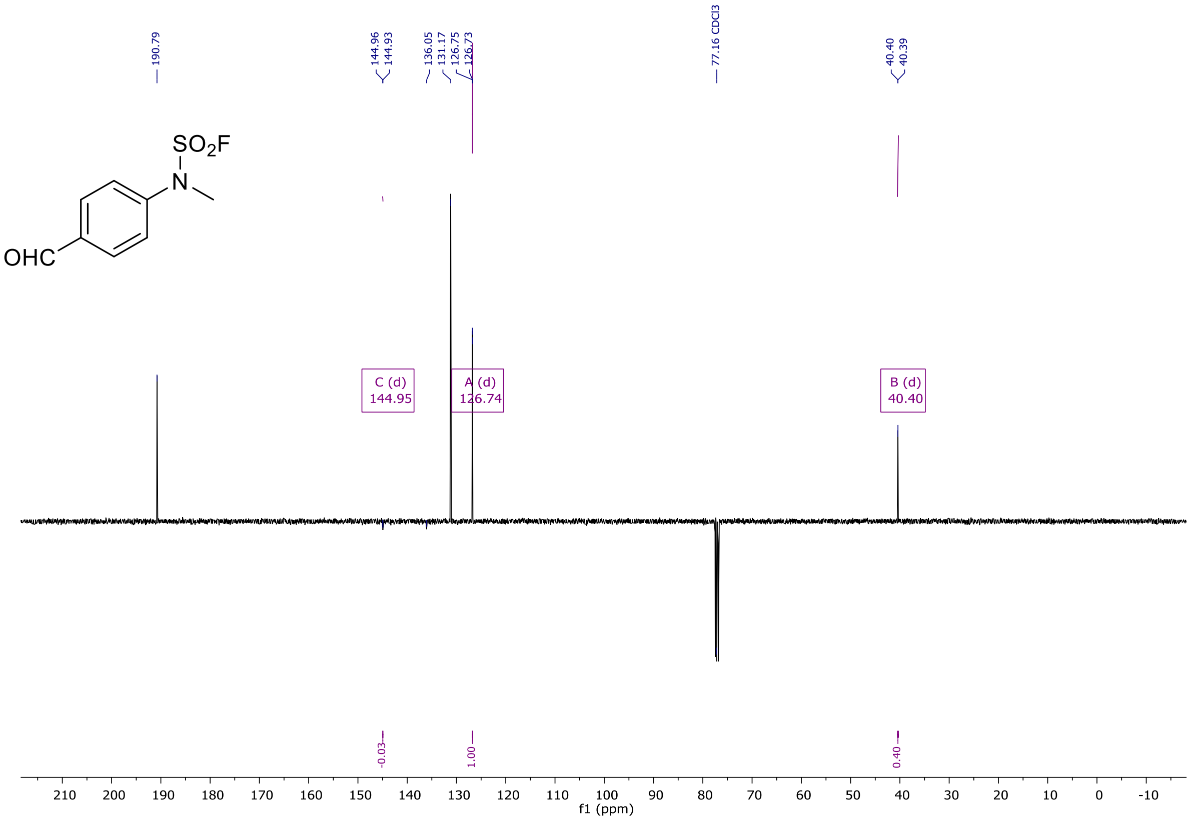

^{19}F NMR

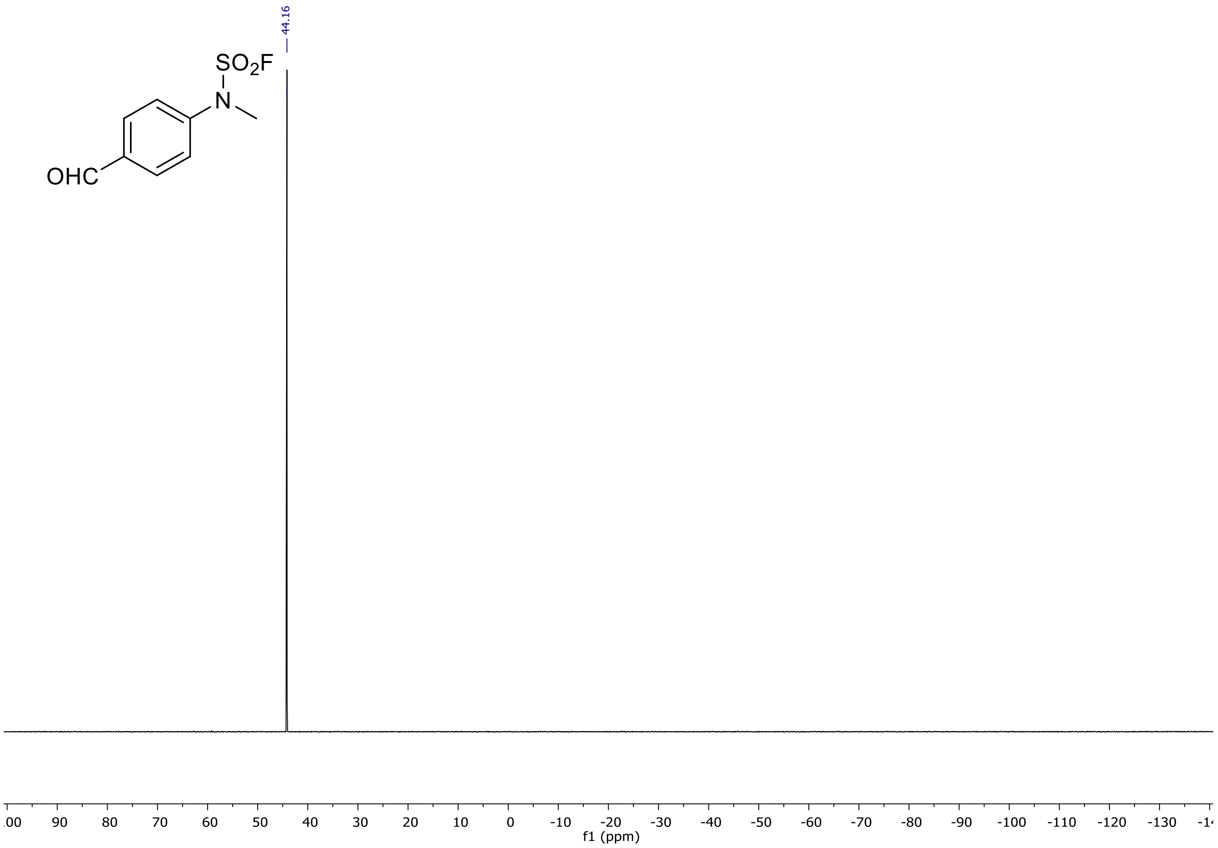
44.16
SO2F
N
OHC
.00 90 80 70 60 50 40 30 20 10 0 -10 -20 -30 -40 -50 -60 -70 -80 -90 -100 -110 -120 -130
f1 (ppm)

3,4-Dihydroquinoline-1(2*H*)-sulfonyl fluoride (**13**)

^{1}H NMR

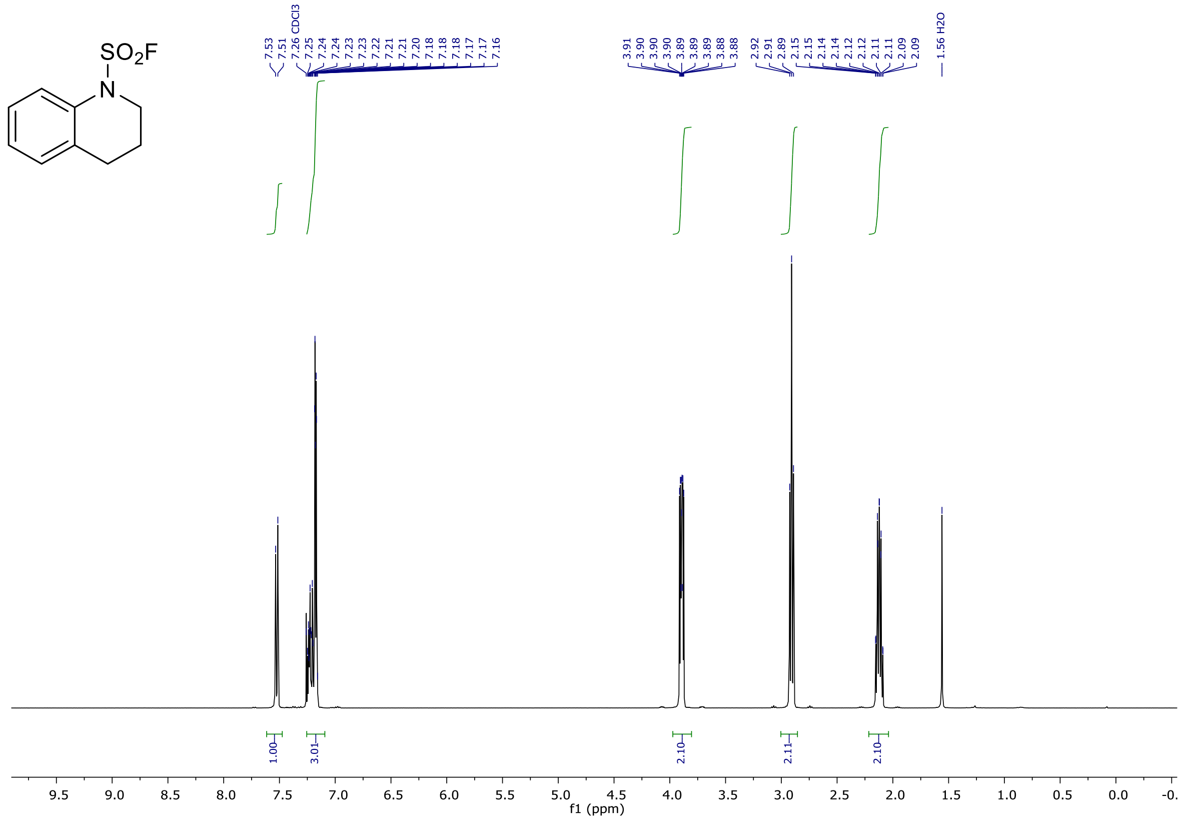

^{13}C{^{1}H} NMR

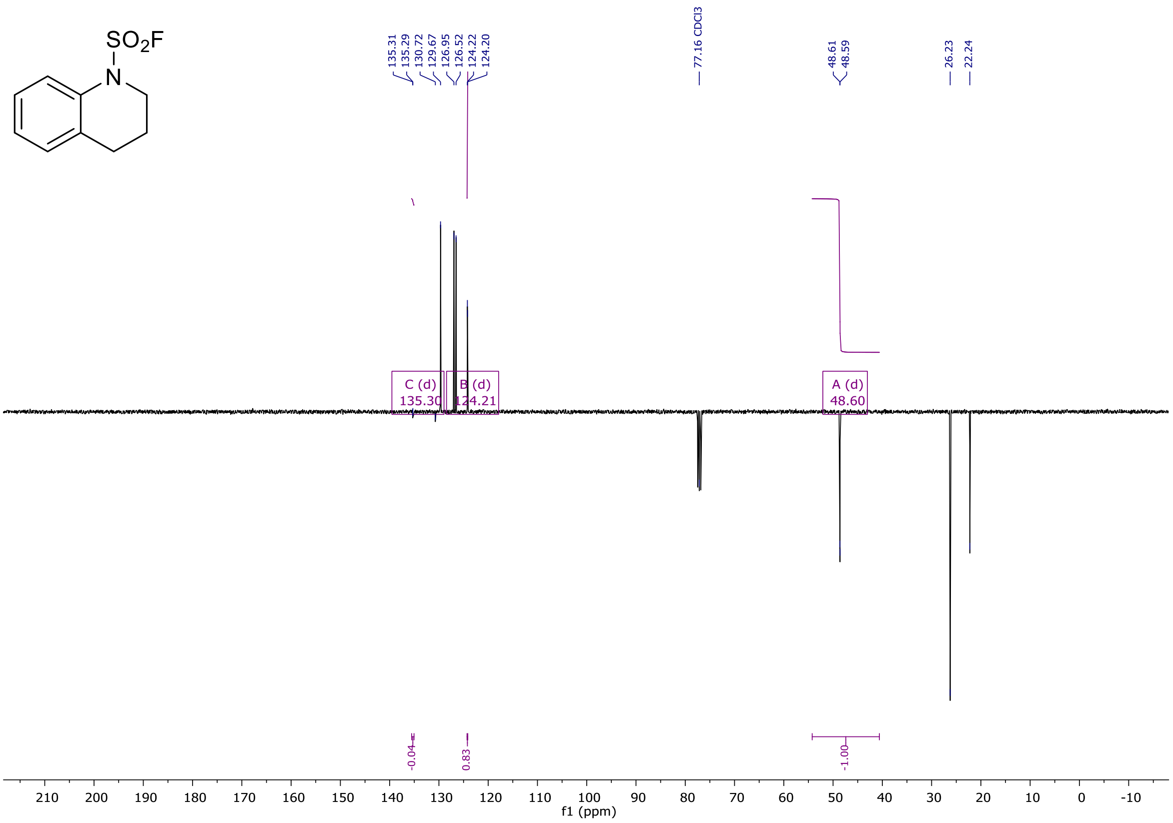

^{19}F NMR

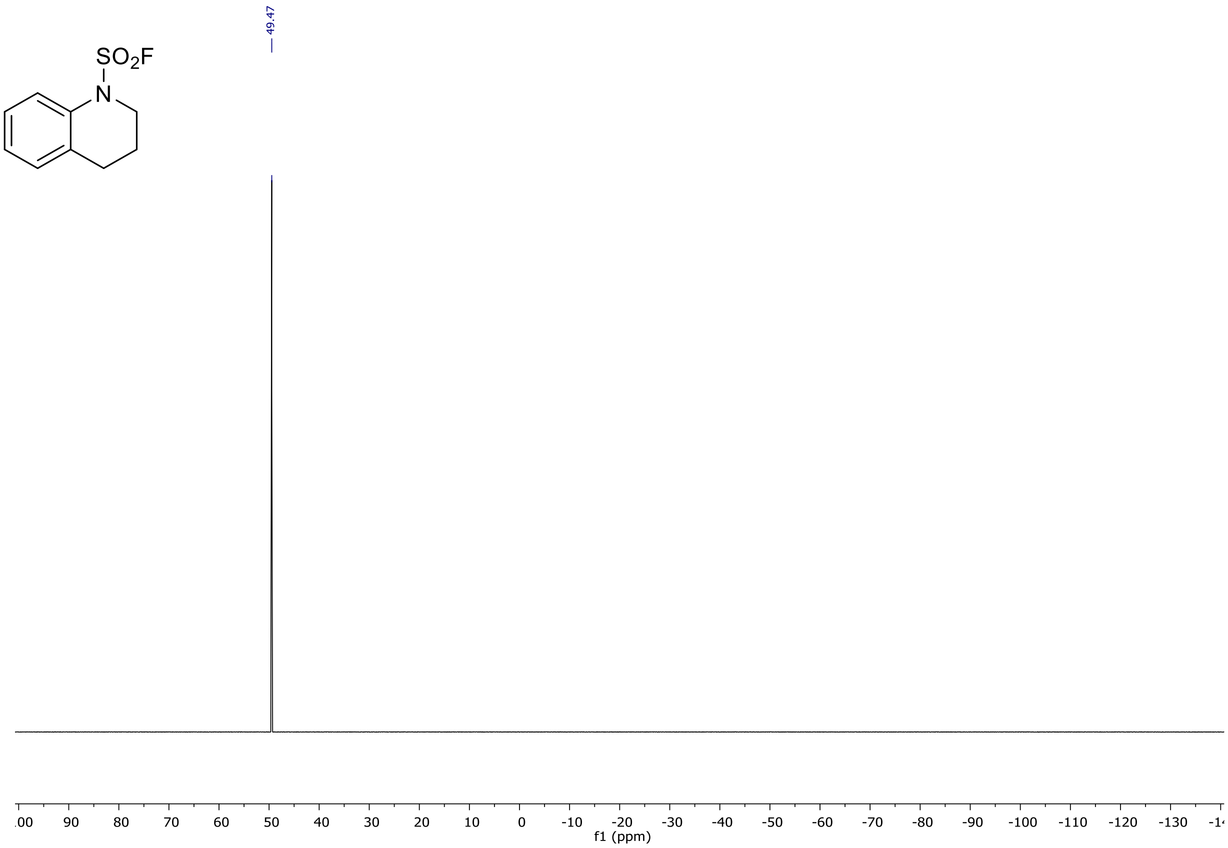

tert-Butyl (*S*)-2-[(*tert*-butoxycarbonyl)amino]-3-[(*S*)-1-(fluorosulfonyl)indolin-3-yl] propanoate [(*S*,*S*)-**14**]

^{1}H NMR

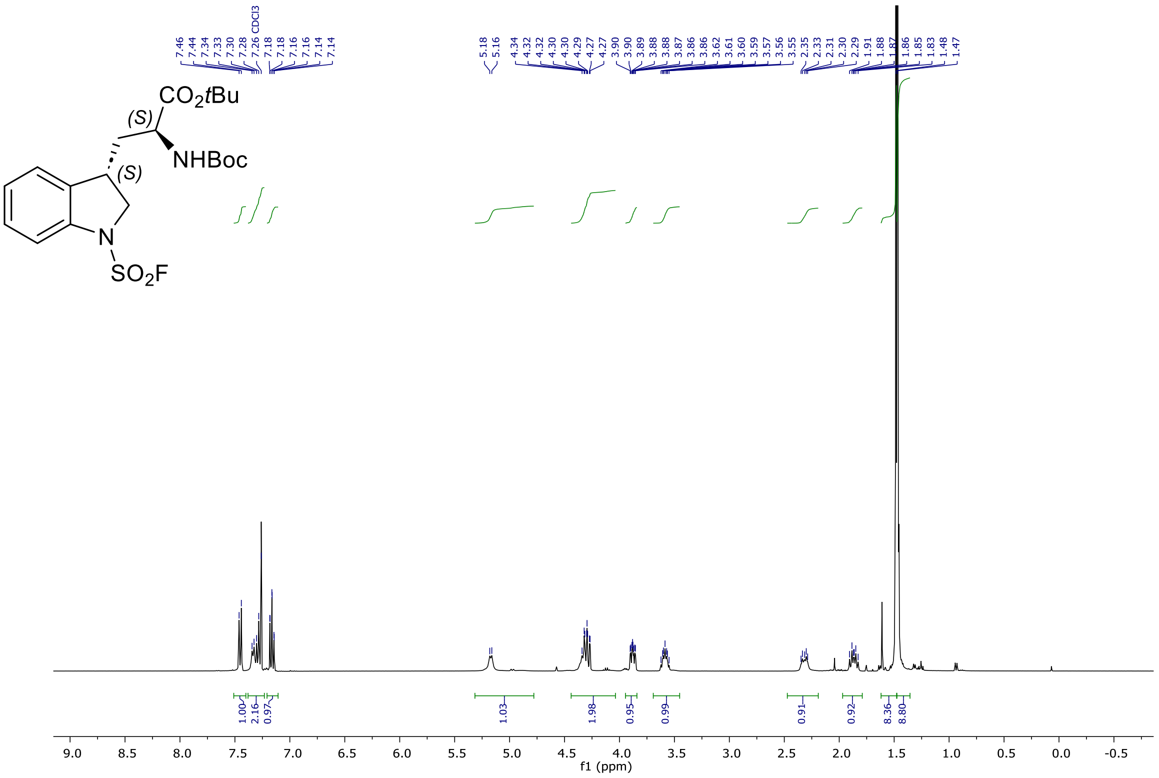

^{13}C{^{1}H} NMR

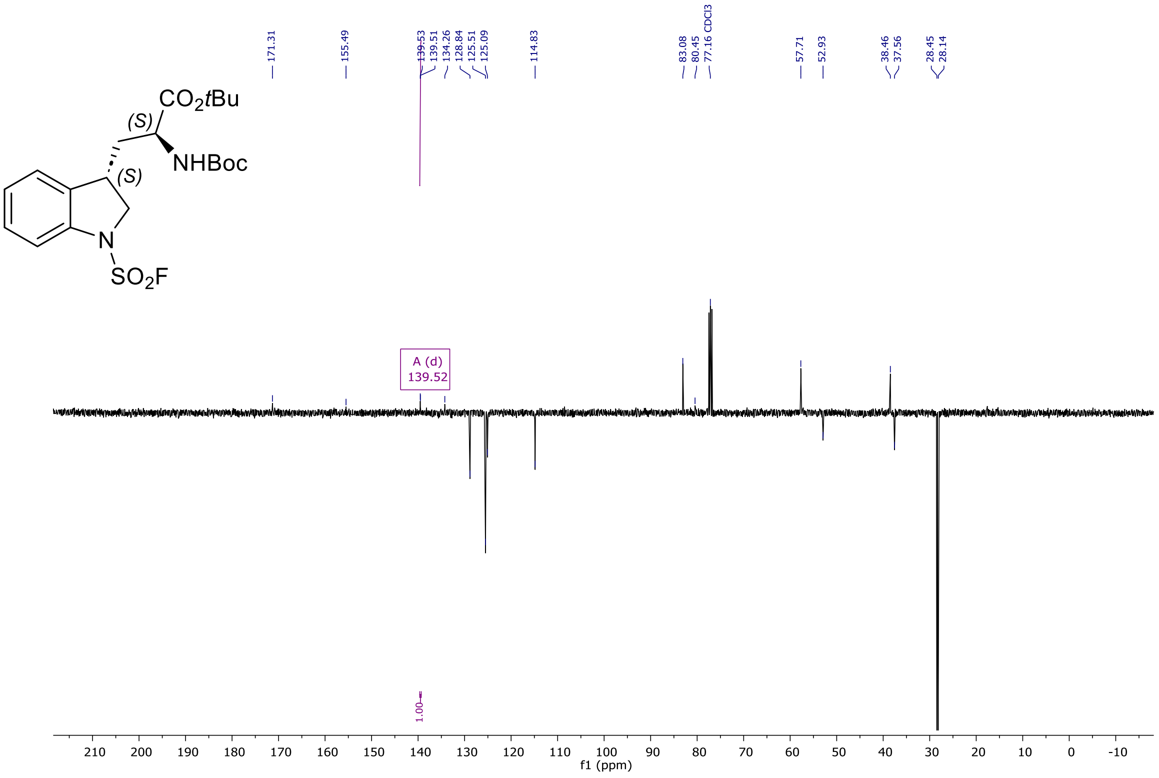

^{19}F NMR

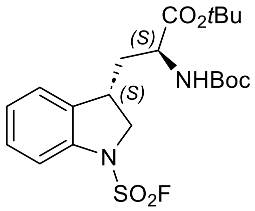

CO_2tBu
(S)
NHBoc
(S)
N
SO_2F

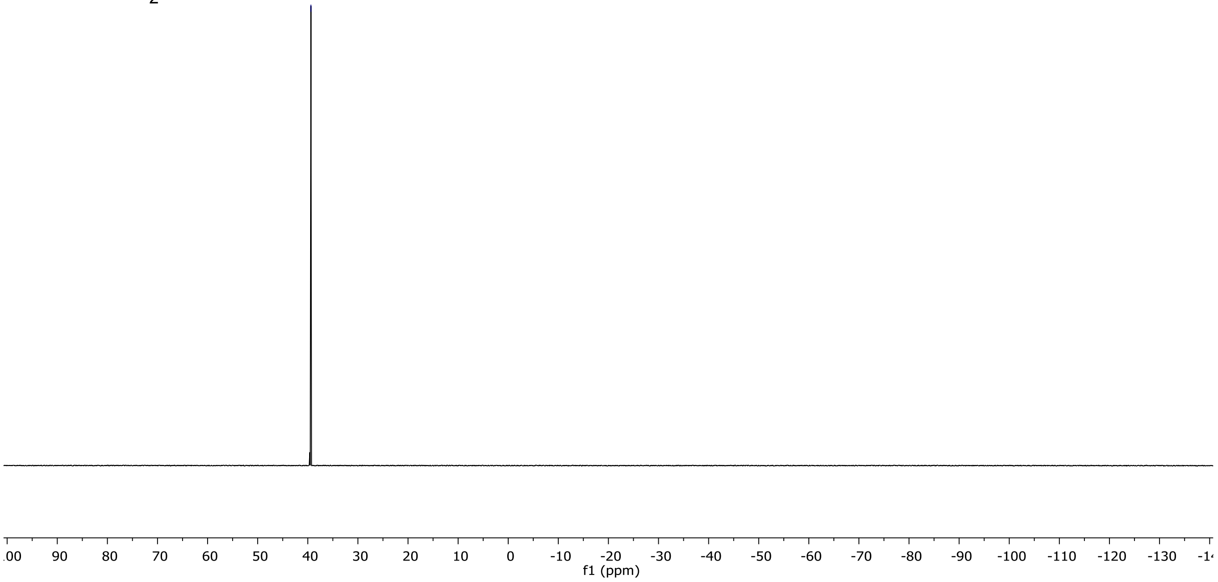

39.38
.00
90
80
70
60
50
40
30
20
10
0
-10
-20
-30
-40
-50
-60
-70
-80
-90
-100
-110
-120
-130
f1 (ppm)

tert-Butyl (*S*)-2-[(*tert*-butoxycarbonyl)amino]-3-[(*R*)-1-(fluorosulfonyl)indolin-3-yl] propanoate [(*S*,*R*)-**14**]

^{1}H NMR

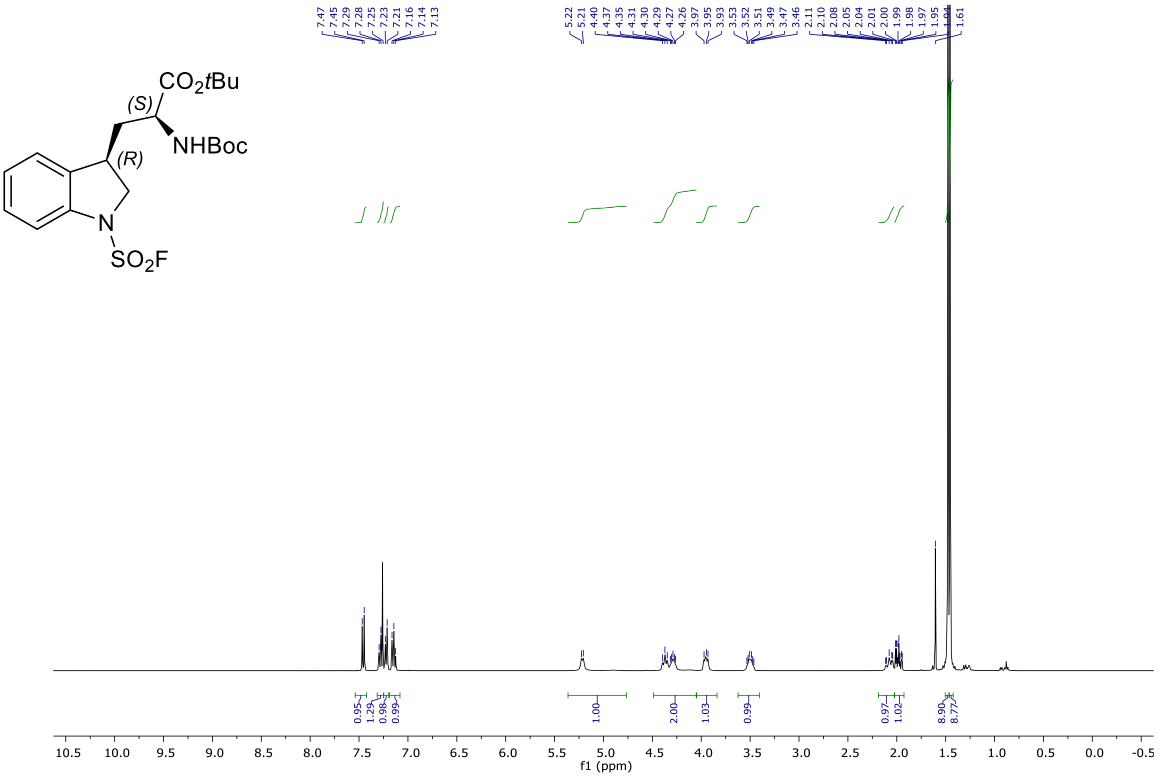

^{13}C{^{1}H} NMR

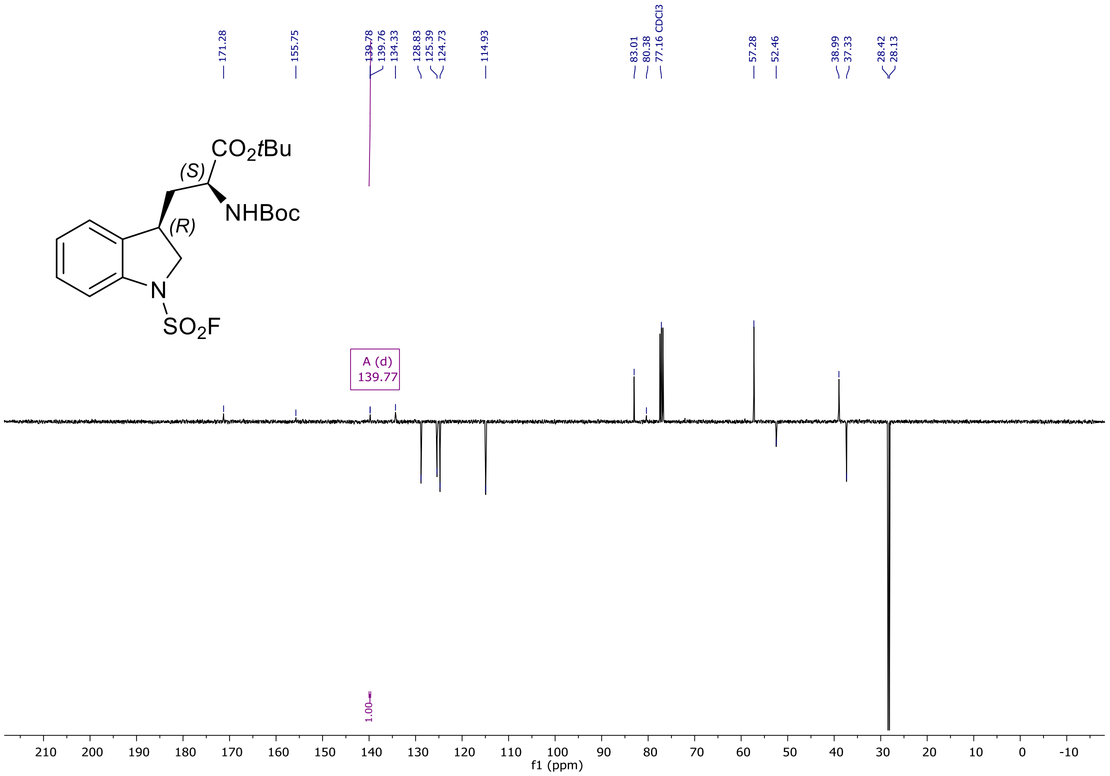

^{19}F NMR

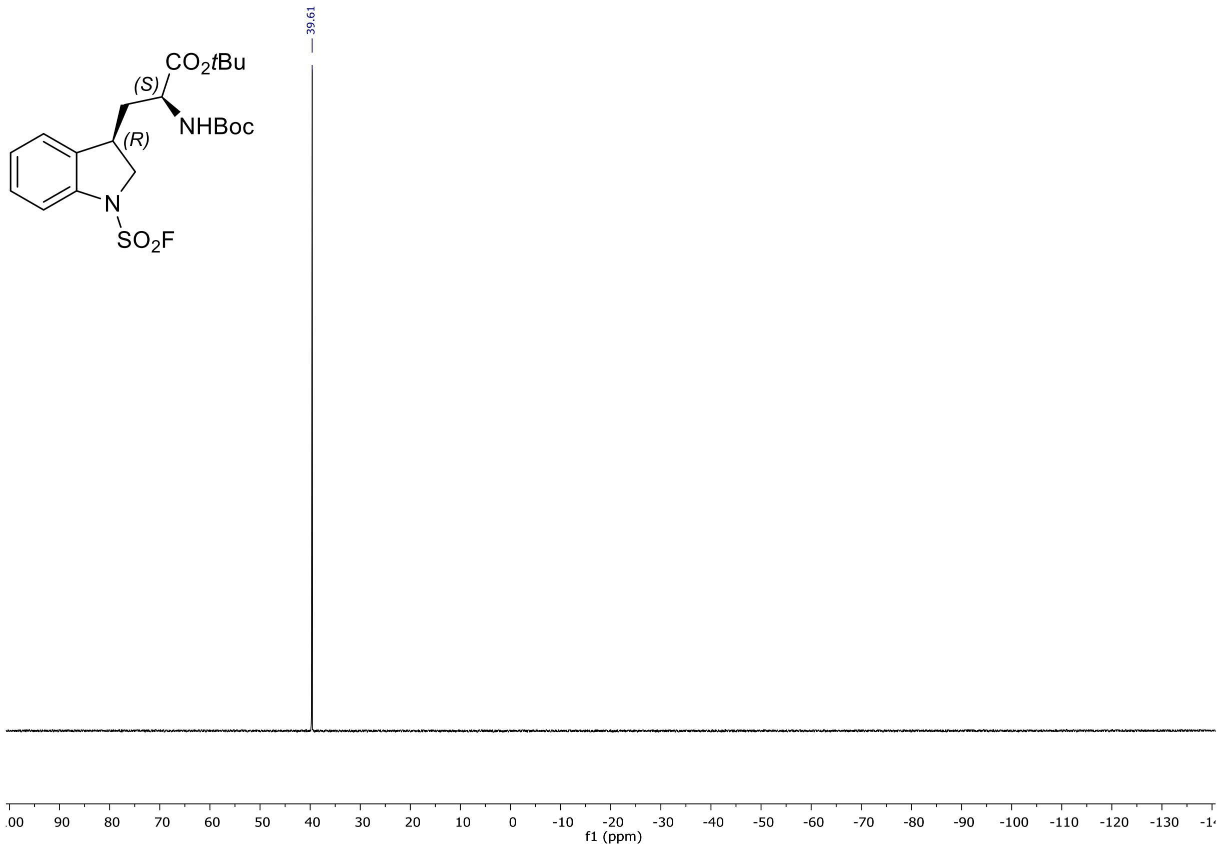

Methyl N_{α}-(*tert*-butoxycarbonyl)-N_{im}-(fluorosulfonyl)-L-histidinate (**15**)

^{1}H NMR

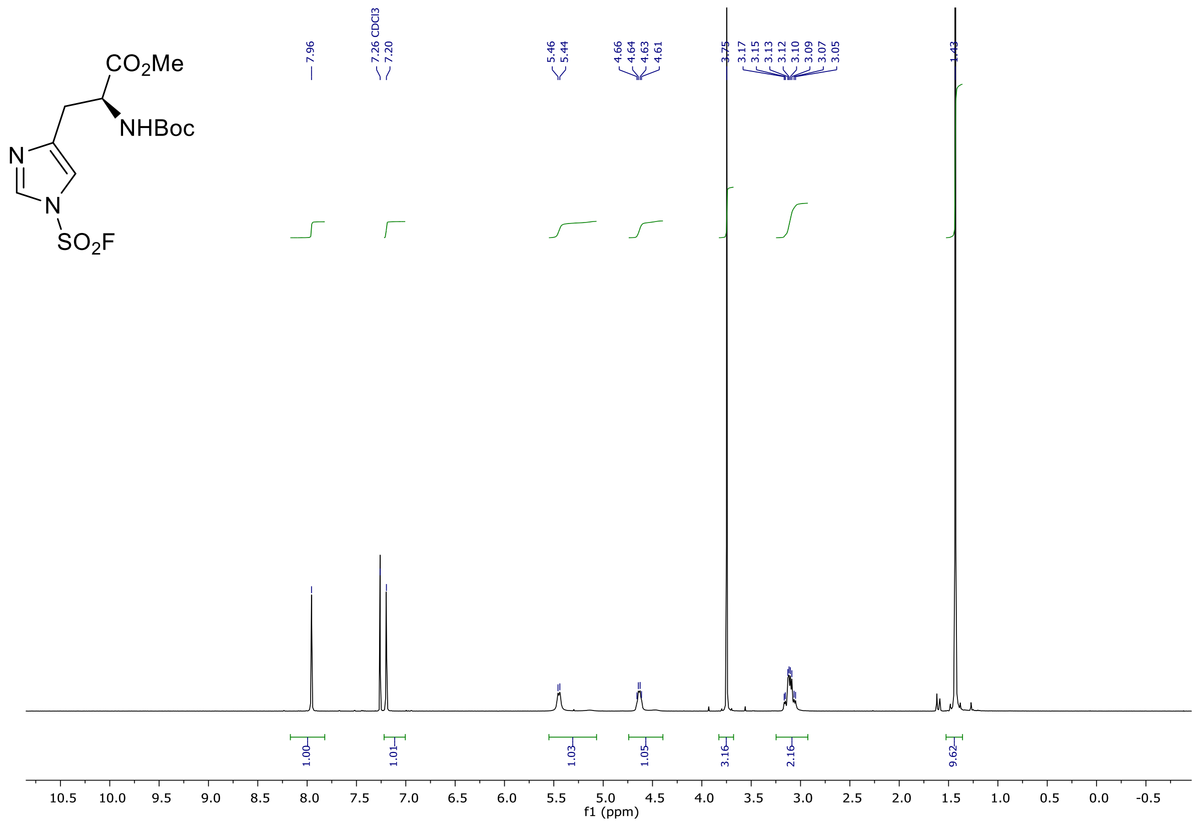

^{13}C{^{1}H} NMR

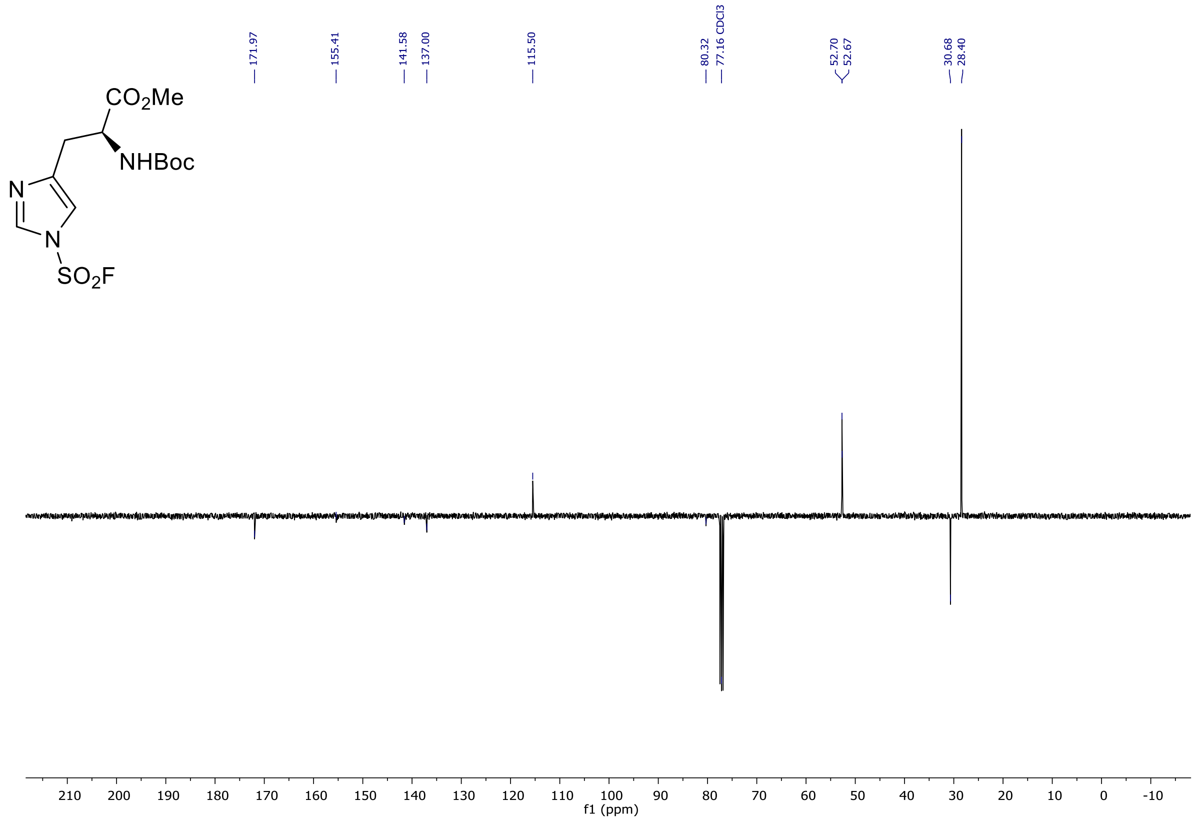

^{19}F NMR

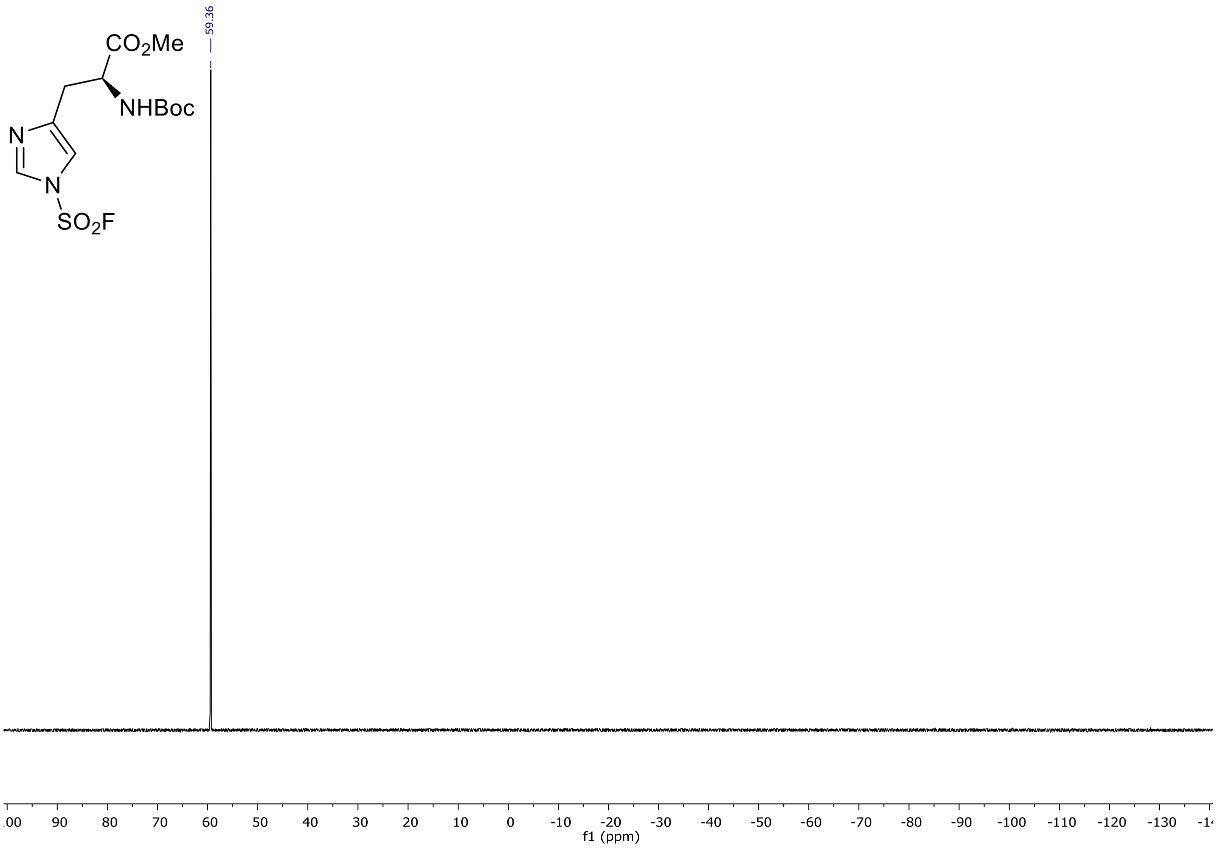

CO2Me
NHBoc
N
N
SO2F
59.36
90
80
70
60
50
40
30
20
10
0
-10
-20
-30
-40
-50
-60
-70
-80
-90
-100
-110
-120
-130
f1 (ppm)

tert-Butyl (*tert*-butoxycarbonyl)-L-tryptophan (**18**)

<u>^{1}H NMR</u>

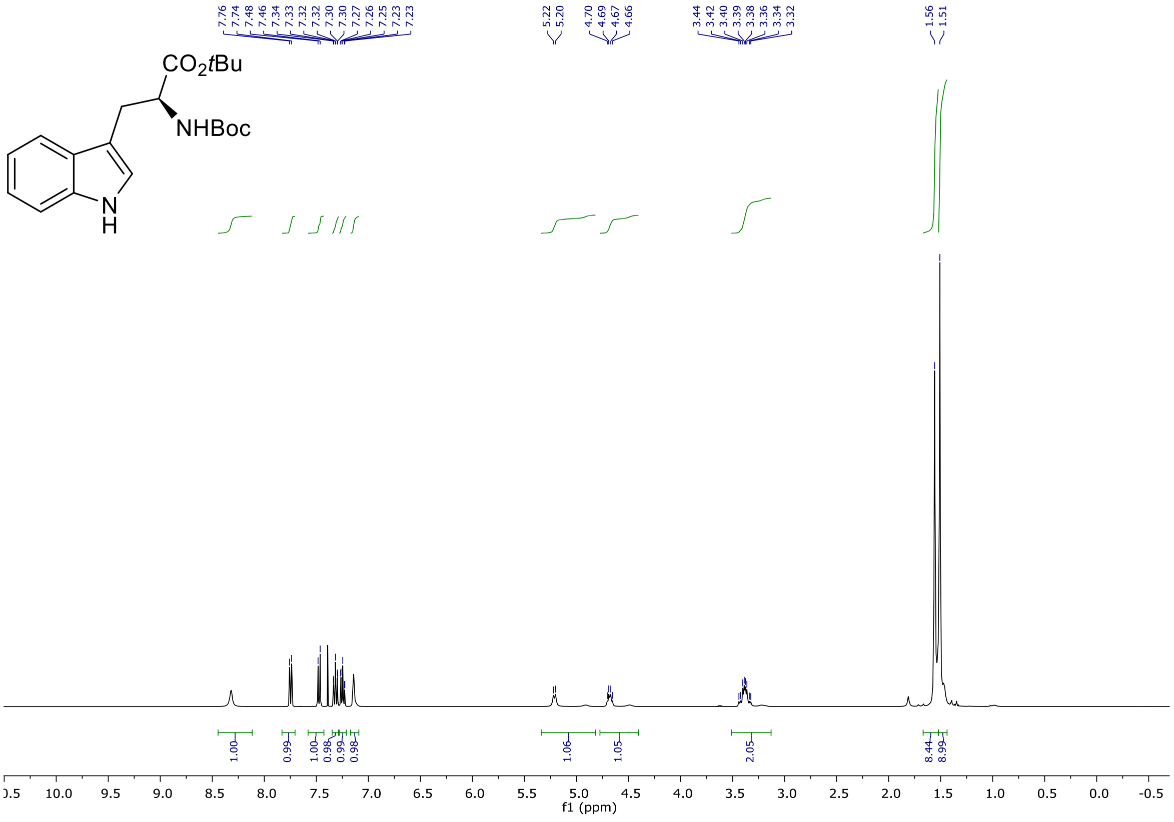

<u>^{13}C{^{1}H} NMR</u>

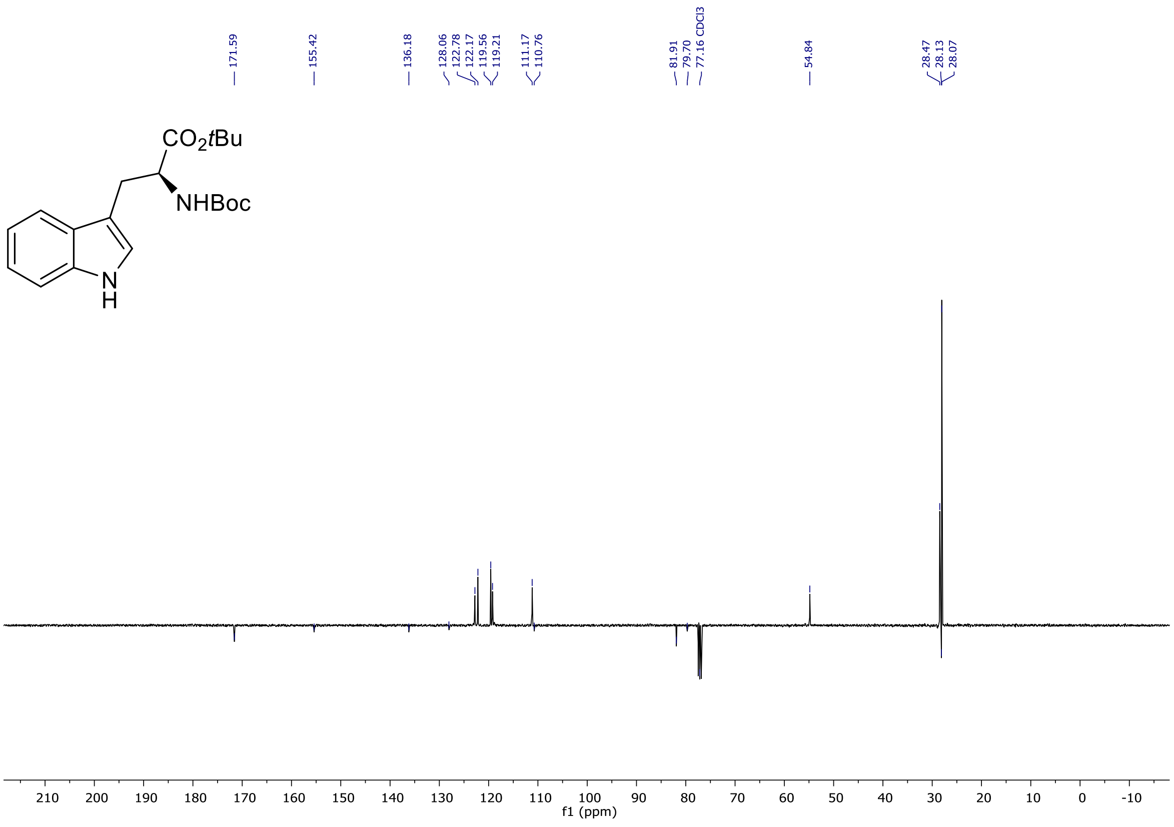

tert-Butyl (*S*)-2-[(*tert*-butoxycarbonyl)amino]-3-[(*S*)-indolin-3-yl]propanoate [(*S*,*S*)-**19**]

<u>^{1}H NMR</u>

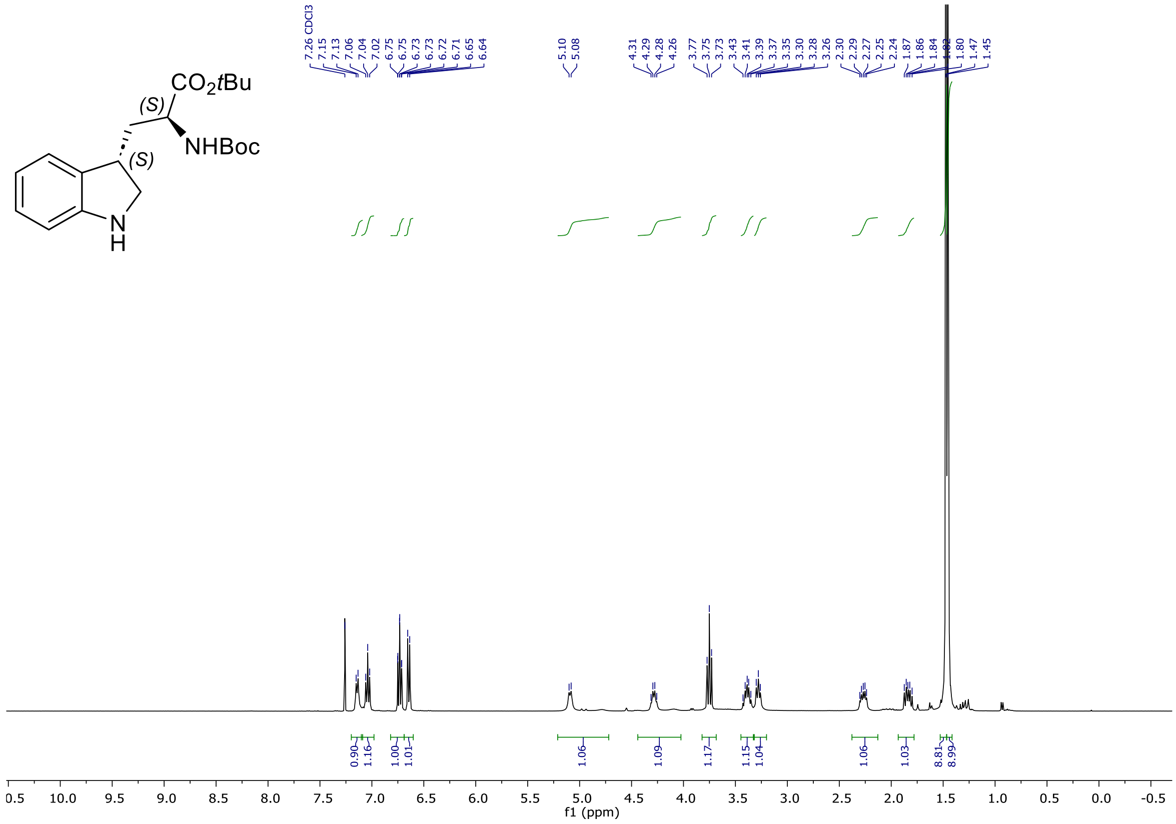

<u>^{13}C{^{1}H} NMR</u>

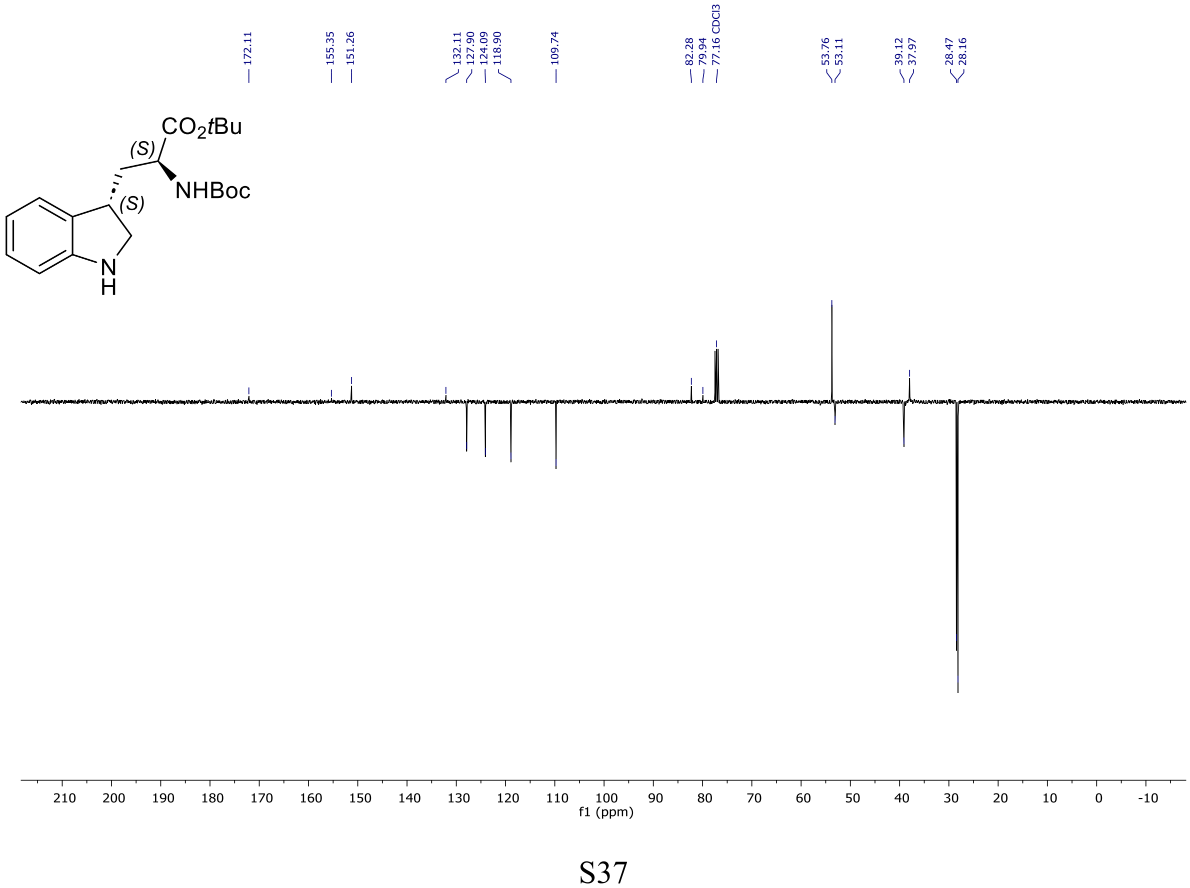

tert-Butyl (*S*)-2-[(*tert*-butoxycarbonyl)amino]-3-[(*R*)-indolin-3-yl]propanoate [(*S*,*R*)-**19**]

^{1}H NMR

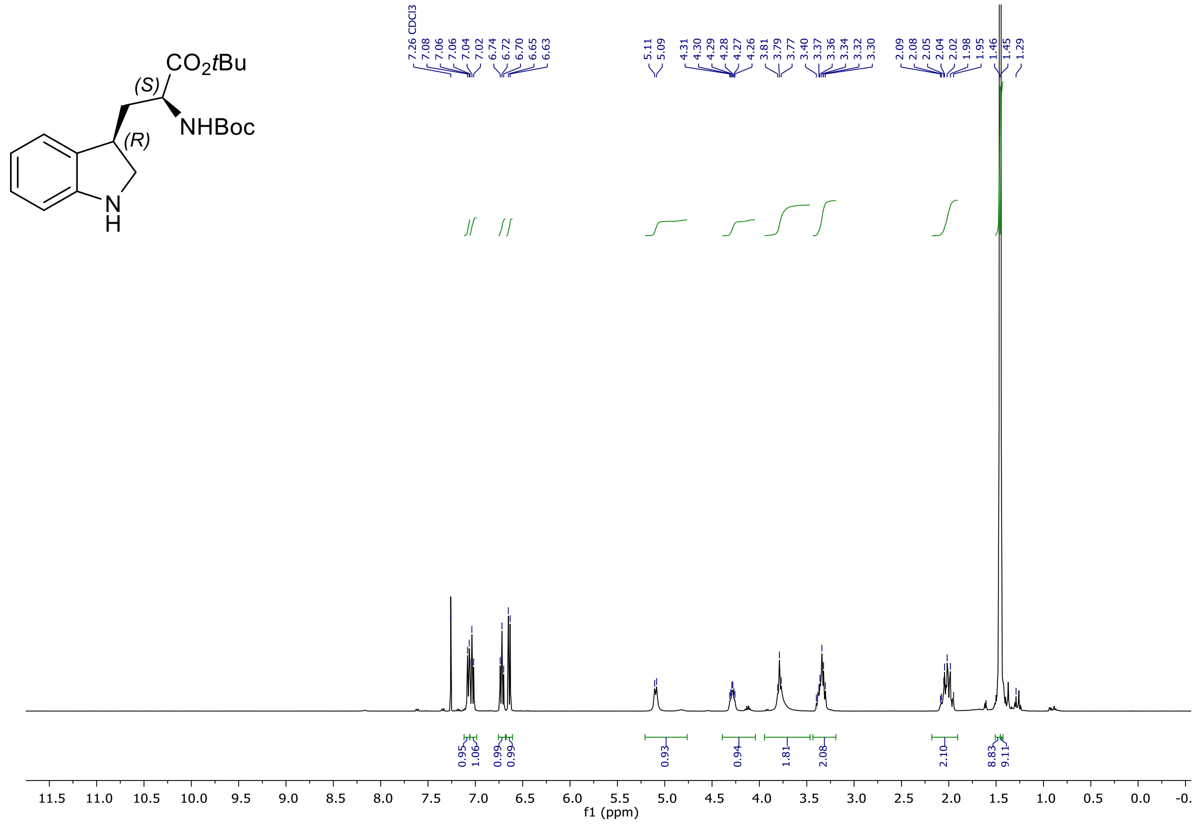

^{13}C{^{1}H} NMR

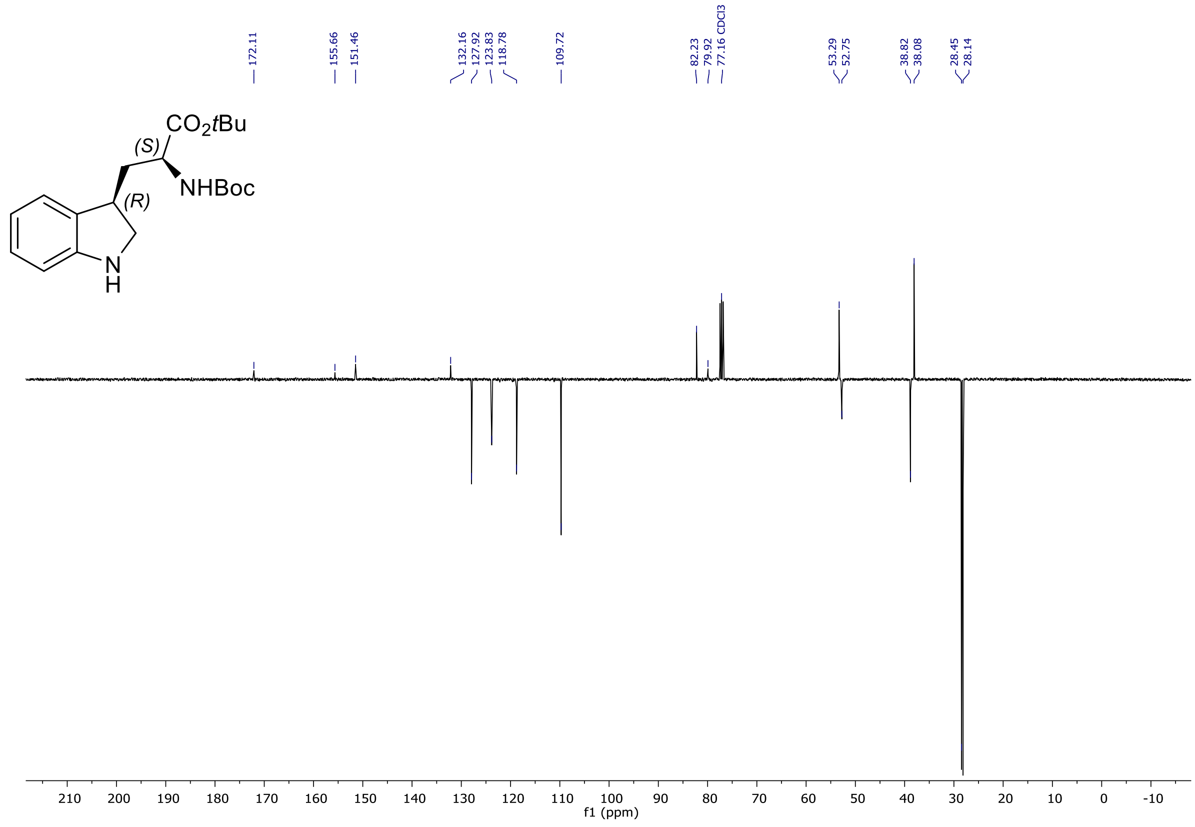

1-(Fluorosulfonyl)-L-tryptophan hydrochloride (**20**·HCl)

^{1}H NMR

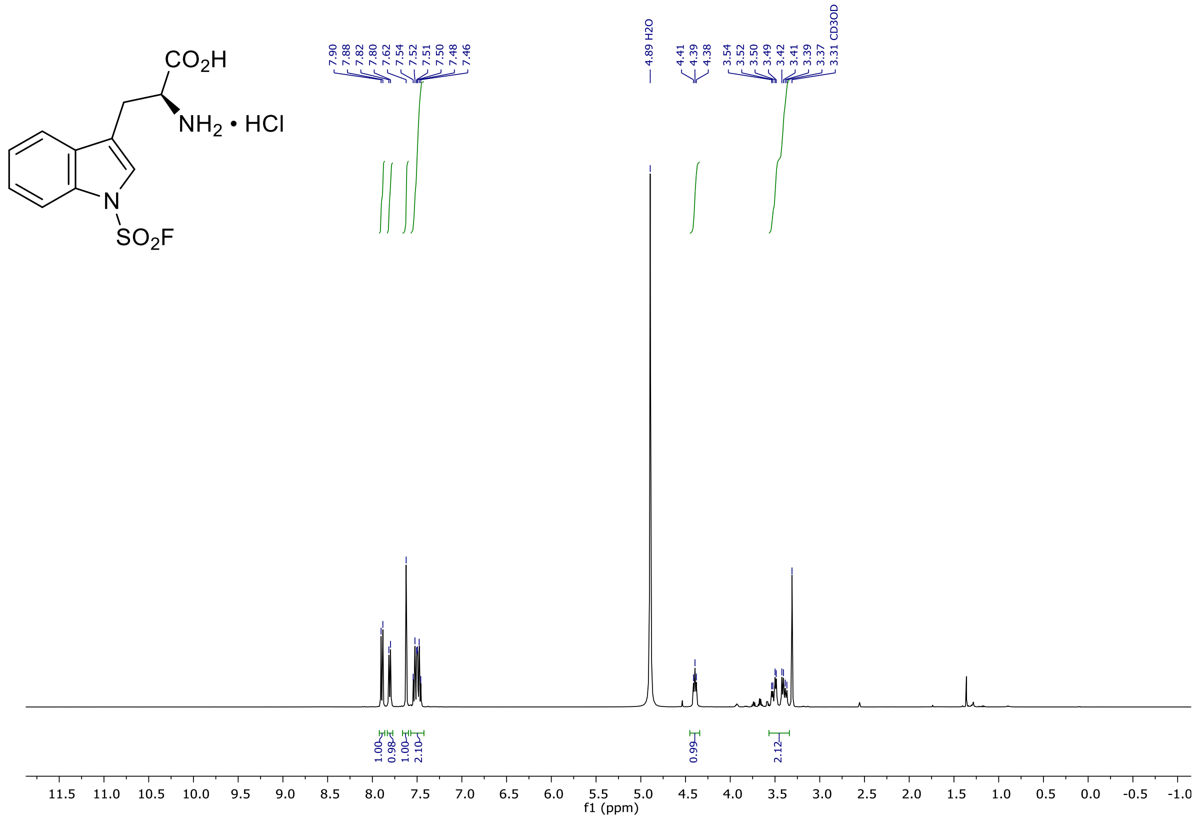

^{13}C{^{1}H} NMR

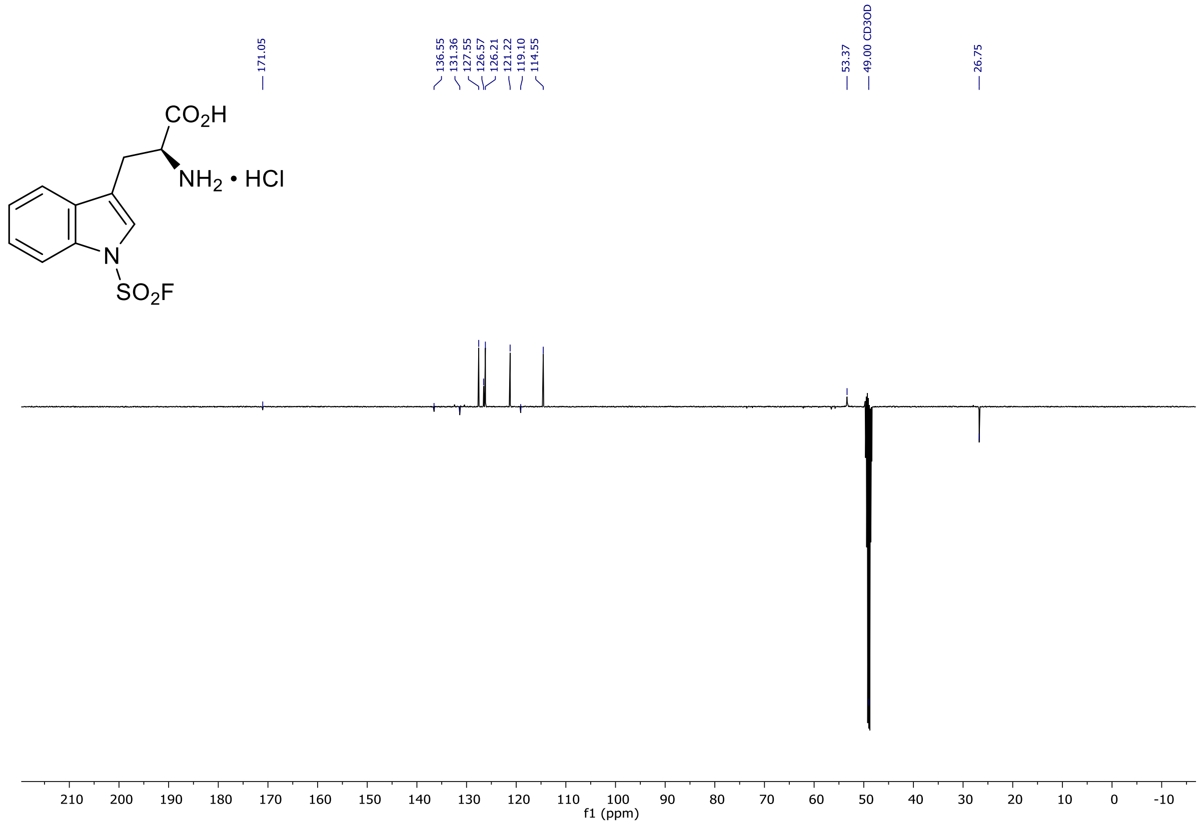

<u>^{19}F NMR</u>

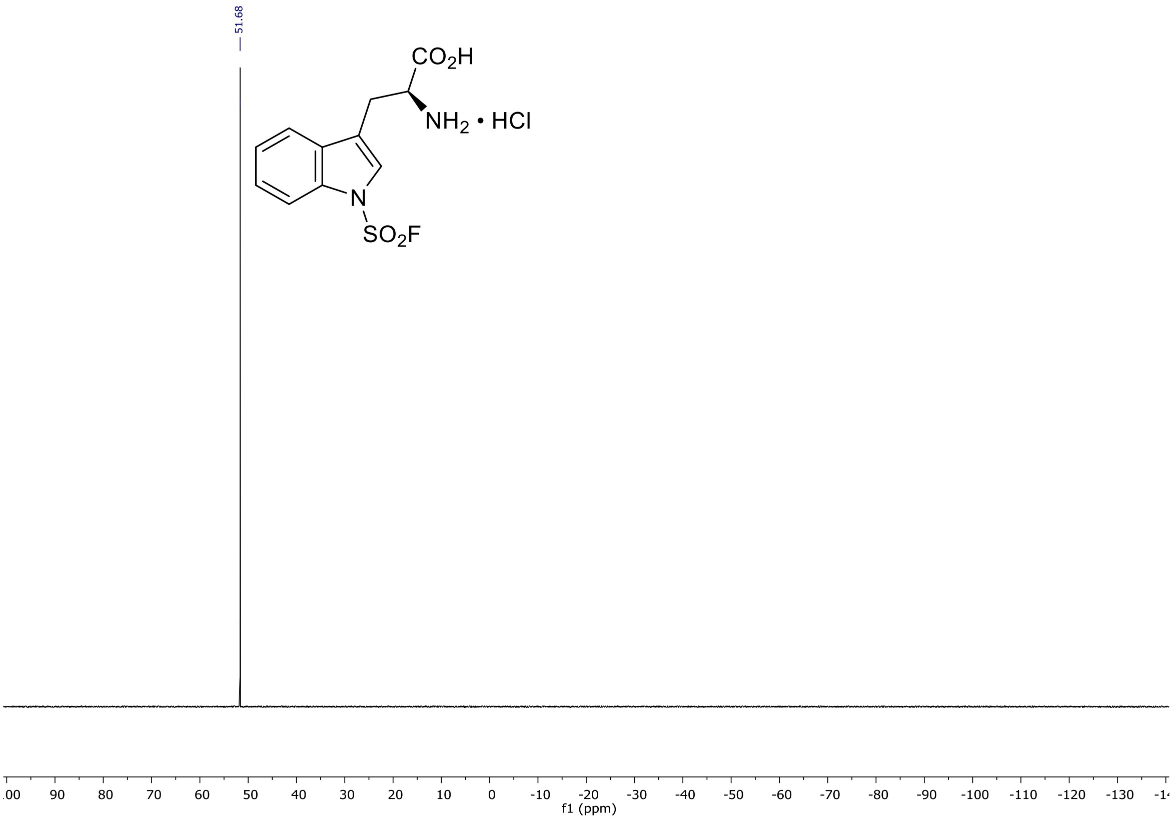

(*S*)-2-Amino-3-[(*R*)-1-(fluorosulfonyl)indolin-3-yl]propanoic acid (**21**·HCl)

^{1}H NMR

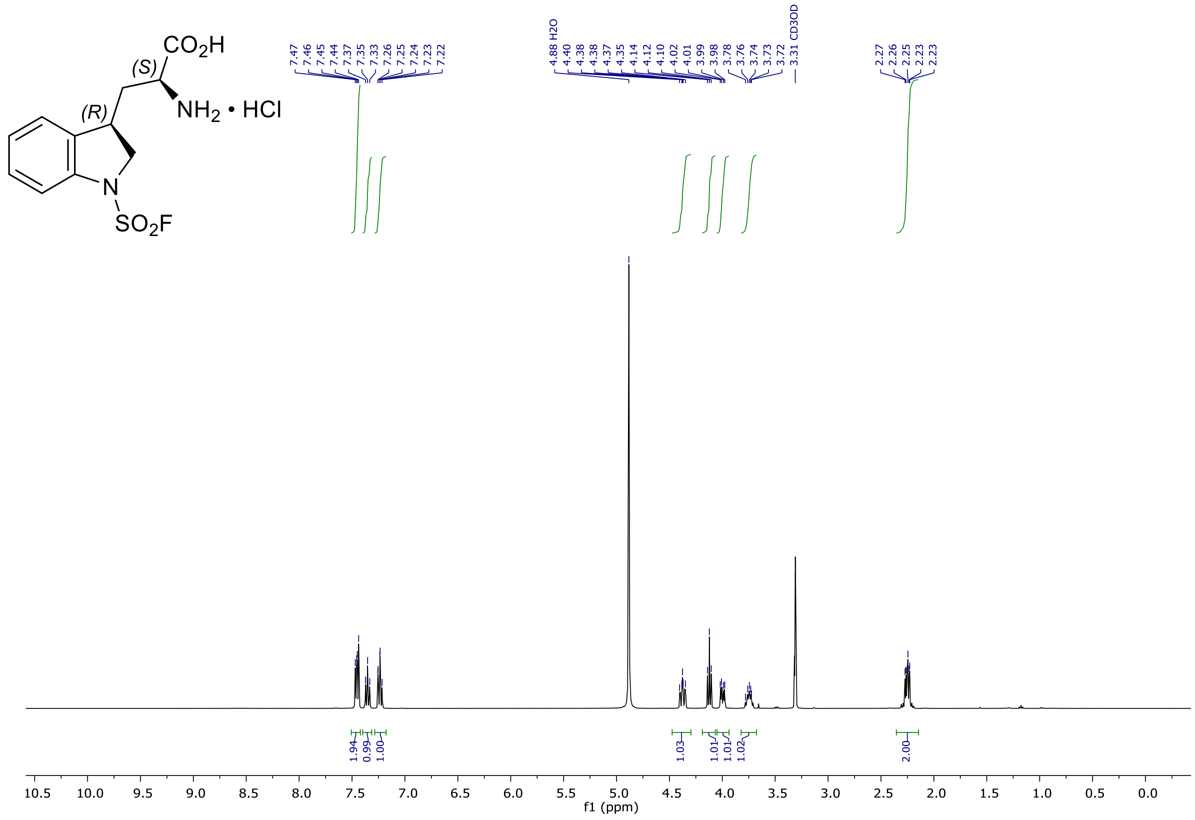

^{13}C{^{1}H} NMR

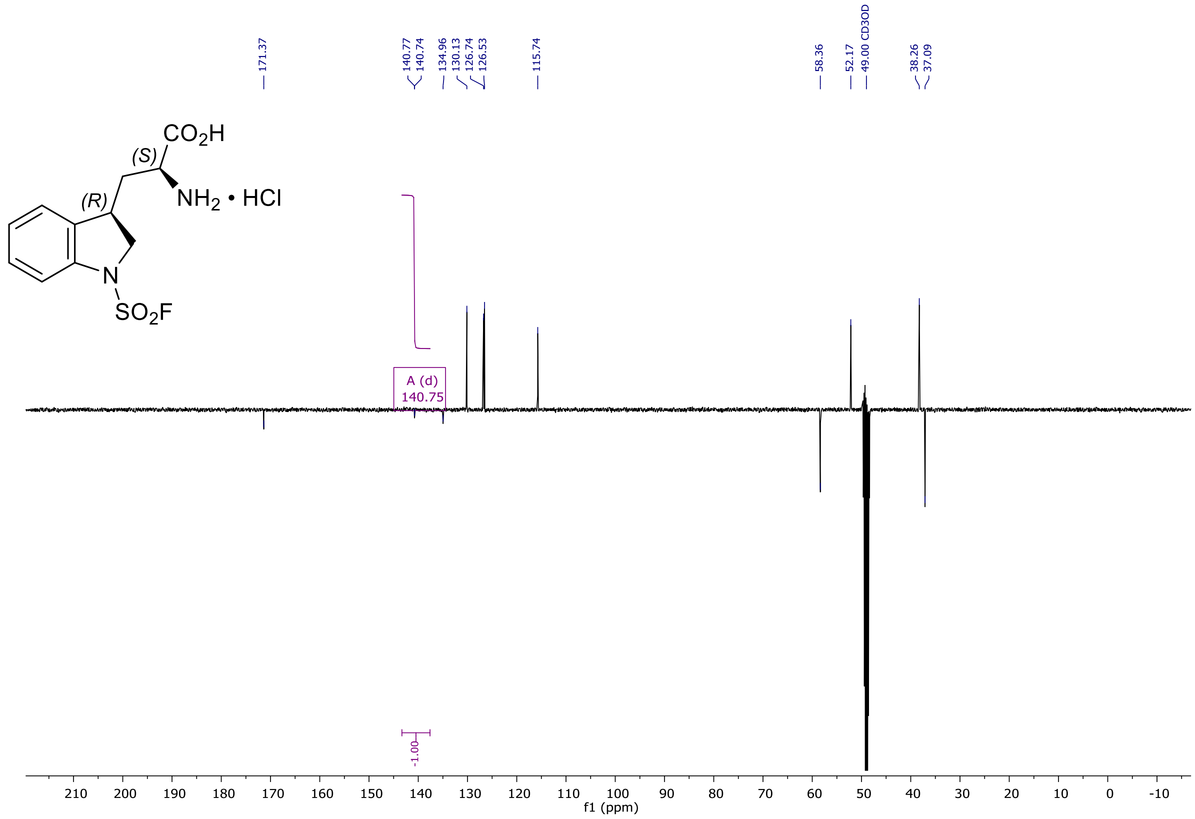

^{19}F NMR

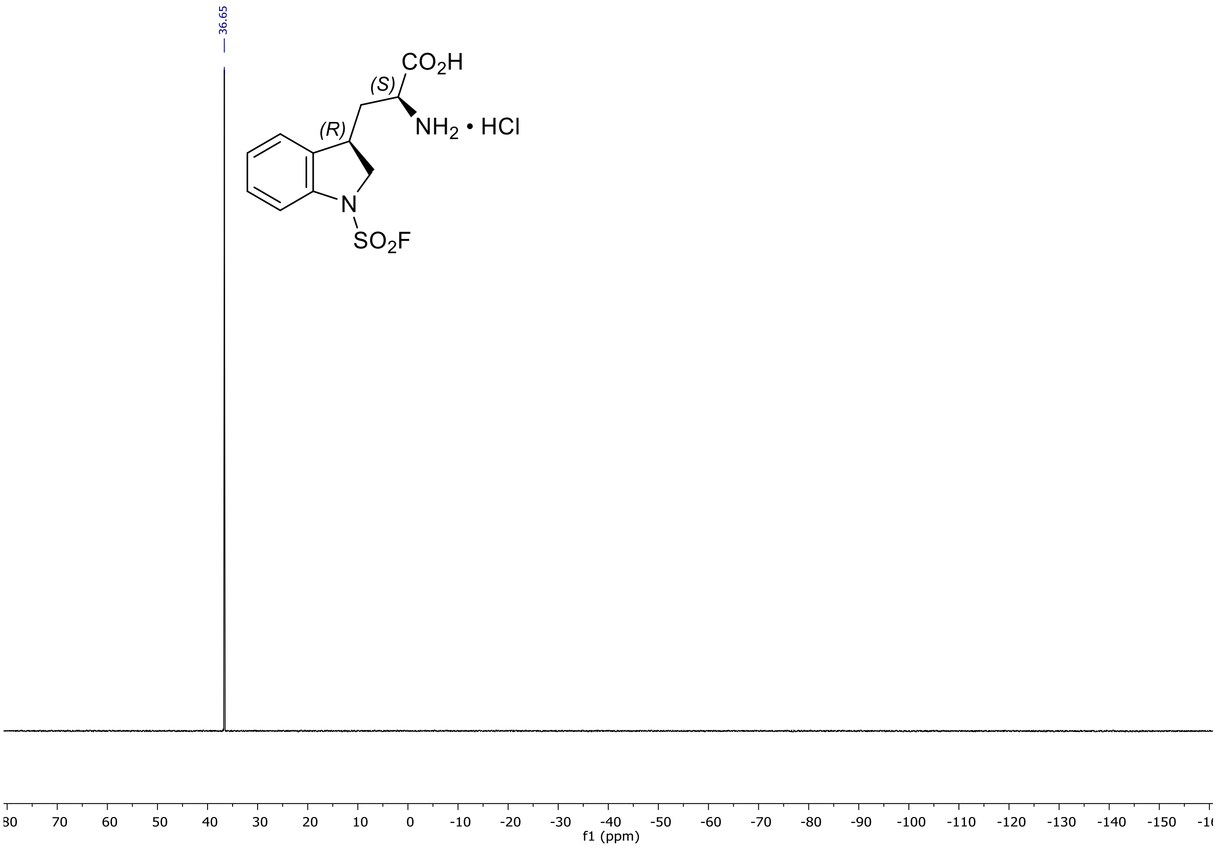

(*S*)-2-Amino-3-[(*S*)-1-(fluorosulfonyl)indolin-3-yl]propanoic acid hydrochloride [(*S,S*)-**21**·HCl]

^{1}H NMR

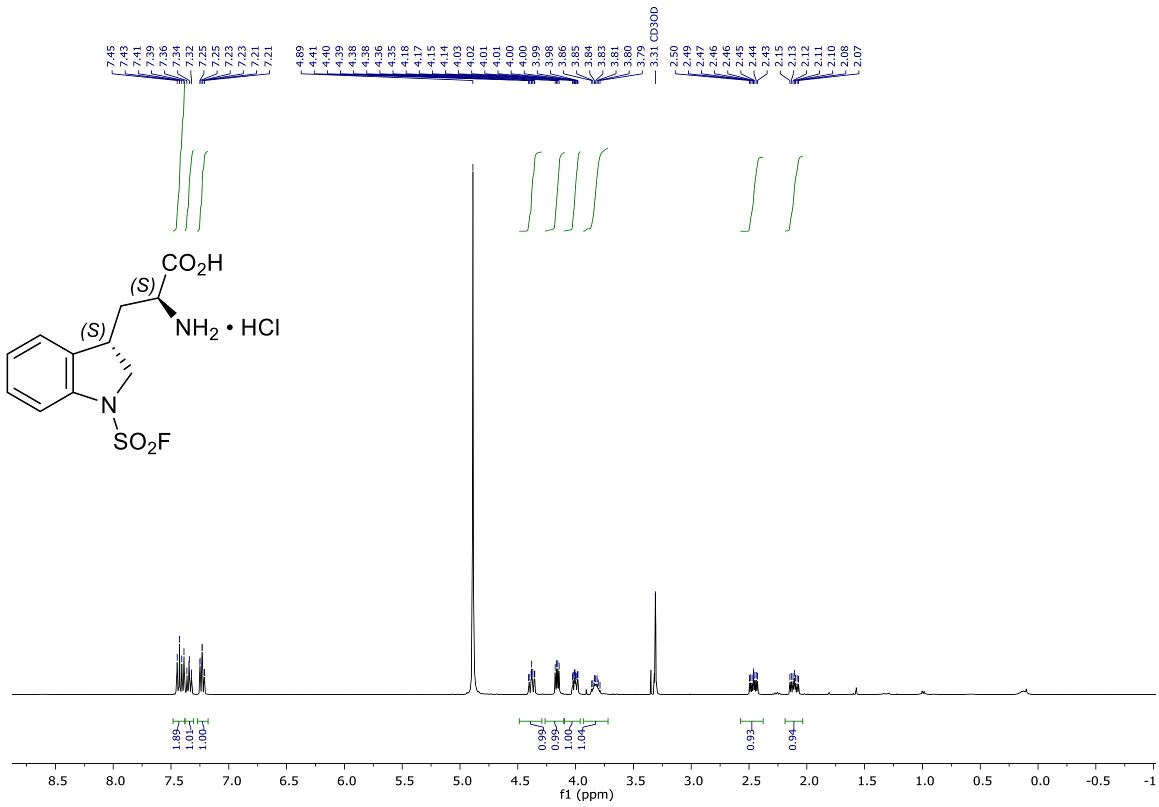

^{13}C{^{1}H} NMR

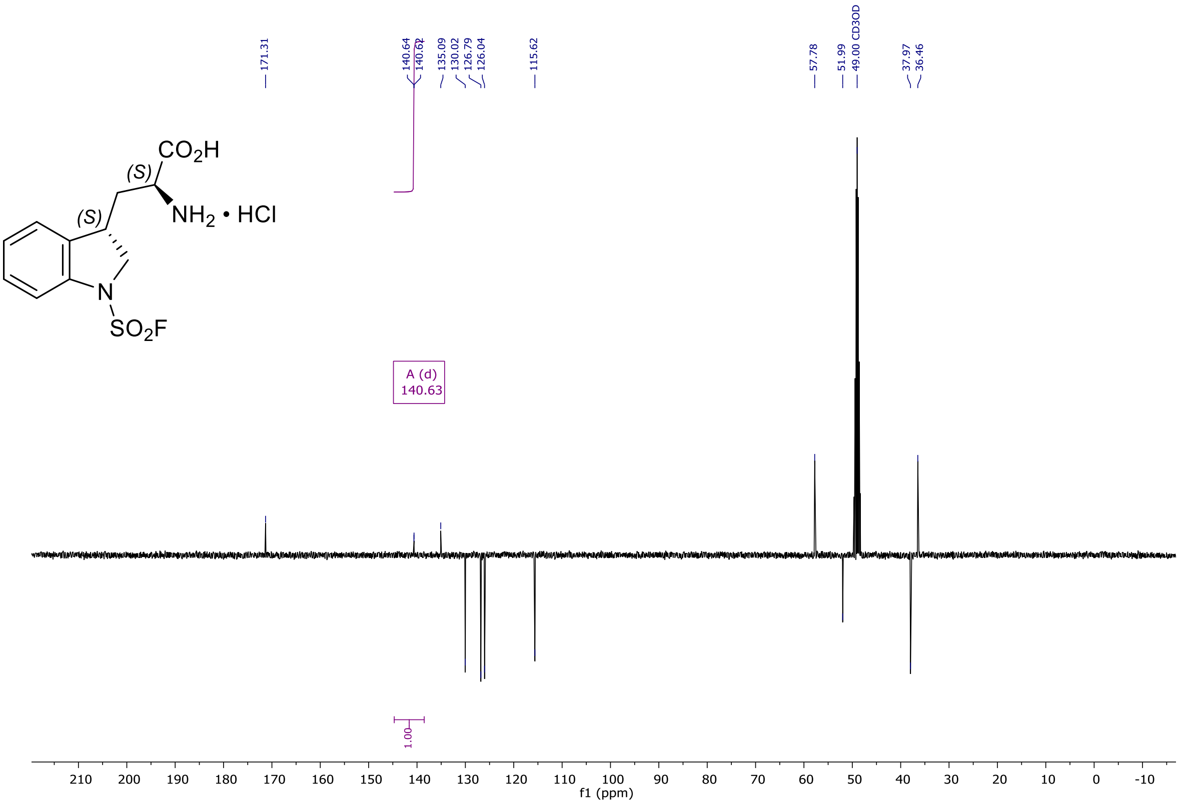

^{19}F NMR

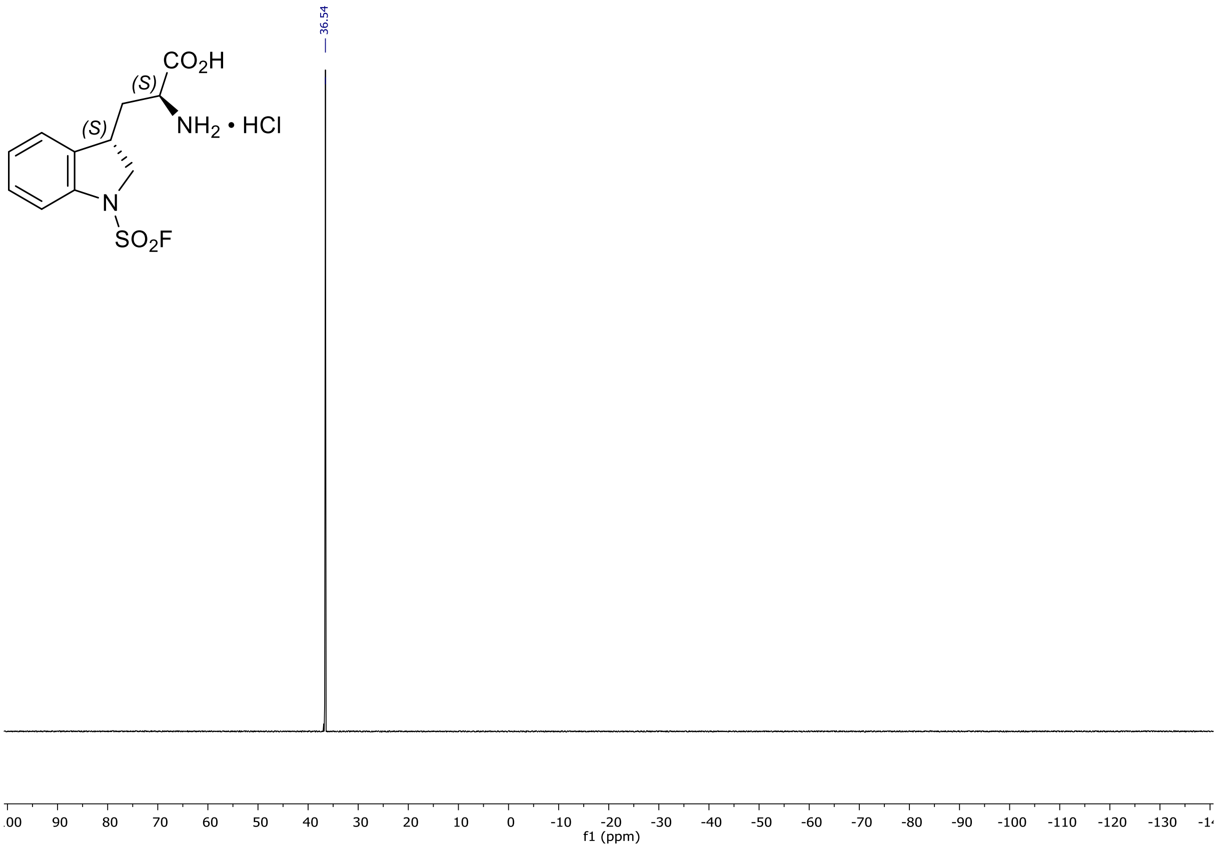

Methyl (*S*)-2-[(*tert*-butoxycarbonyl)amino]-3-(1*H*-indol-5-yl)propanoate (**22**)

<u>^{1}H NMR</u>

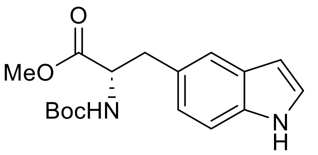

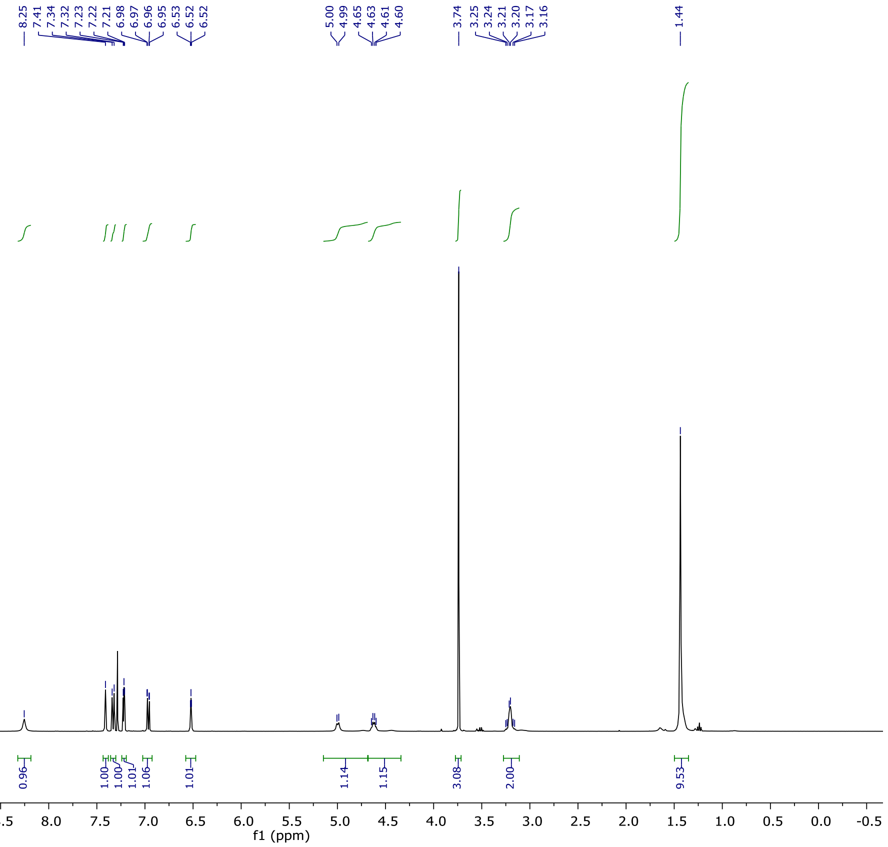

(*S*)-2-[(*tert*-Butoxycarbonyl)amino]-3-(1*H*-indol-5-yl)propanoic acid (**23**)

^{1}H NMR

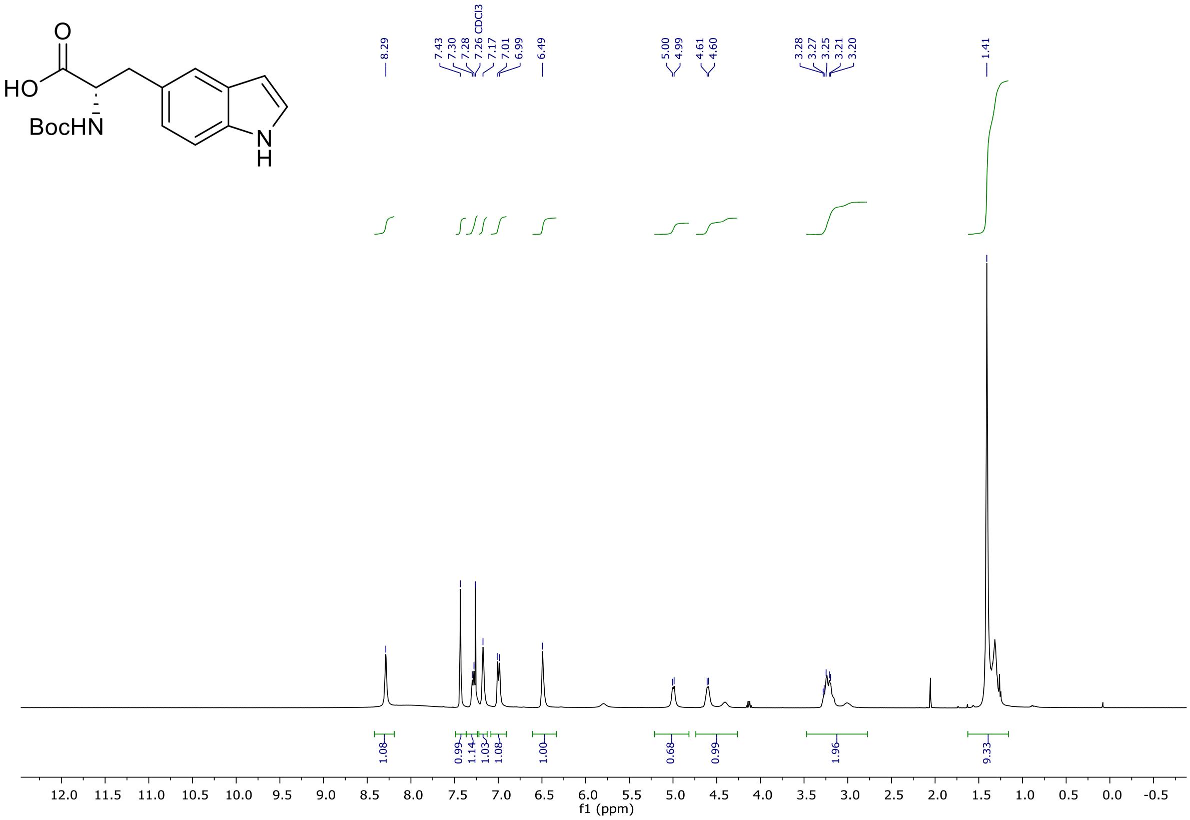

^{13}C{^{1}H} NMR

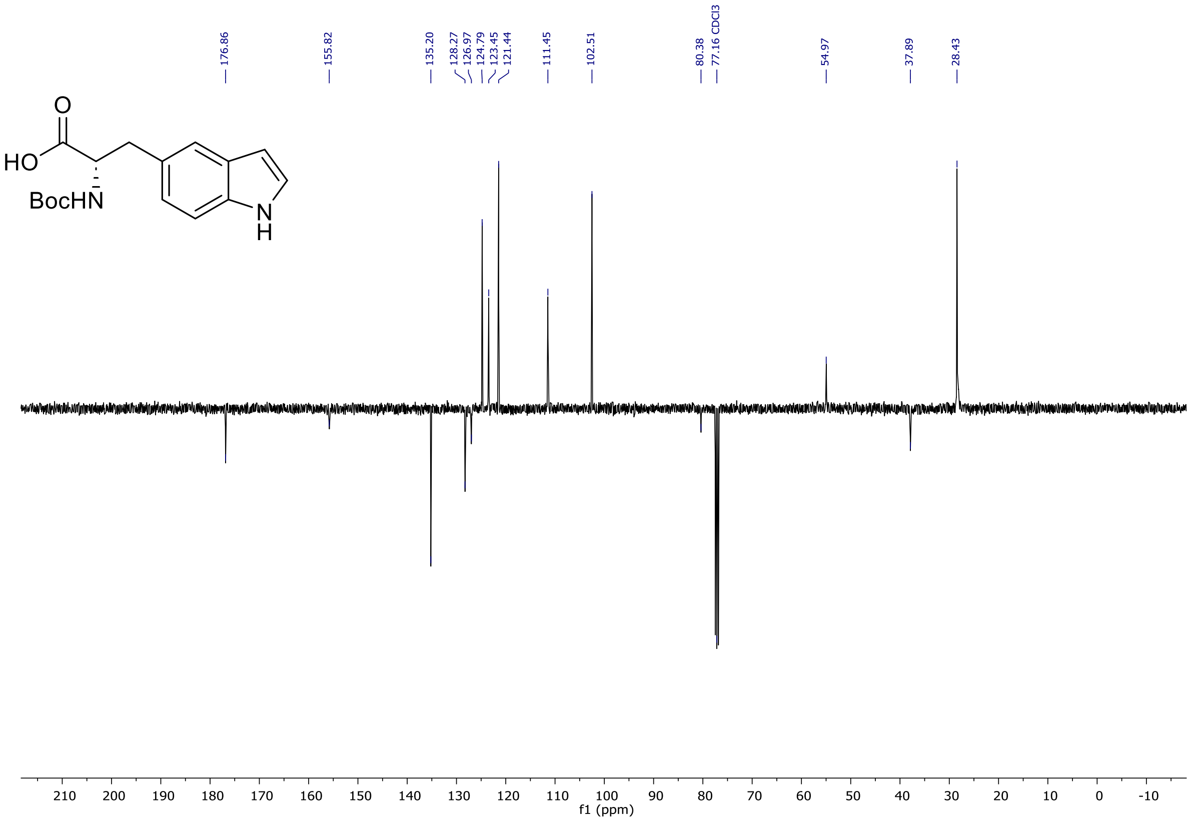

Dicyclopropylmethyl (*S*)-2-[(*tert*-butoxycarbonyl)amino]-3-(1*H*-indol-5-yl)propanoate (**24**)

^{1}H NMR

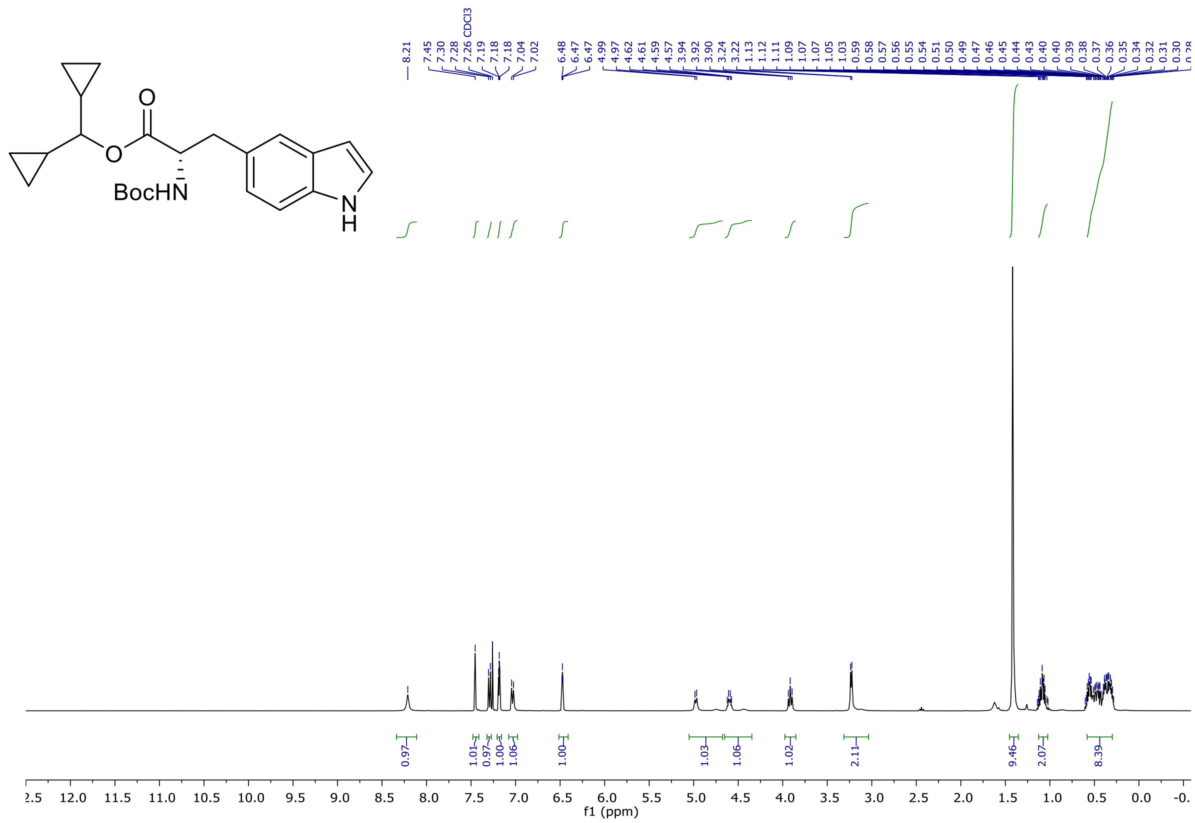

^{13}C{^{1}H} NMR

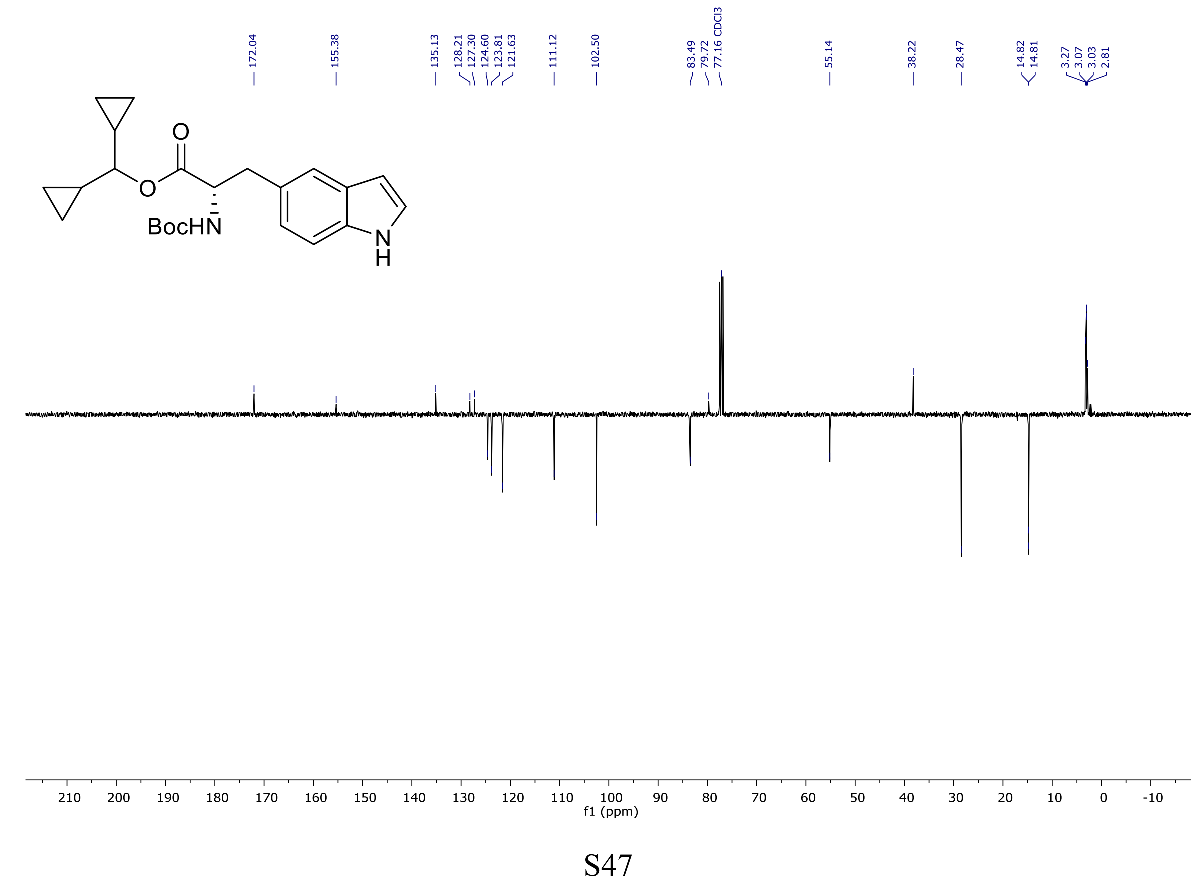

Dicyclopropylmethyl (*S*)-2-[(*tert*-butoxycarbonyl)amino]-3-[1-(fluorosulfonyl)-1*H*-indol-5-yl]propanoate (**25**)

^{1}H NMR

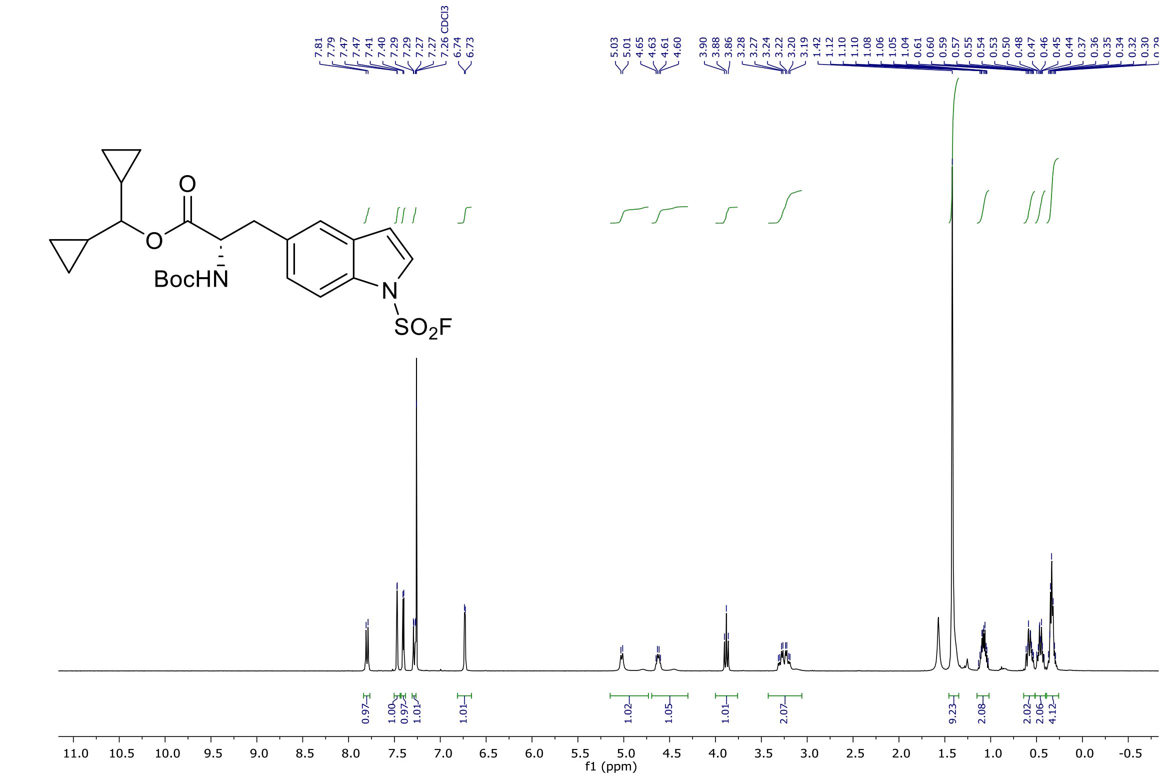

^{13}C{^{1}H} NMR

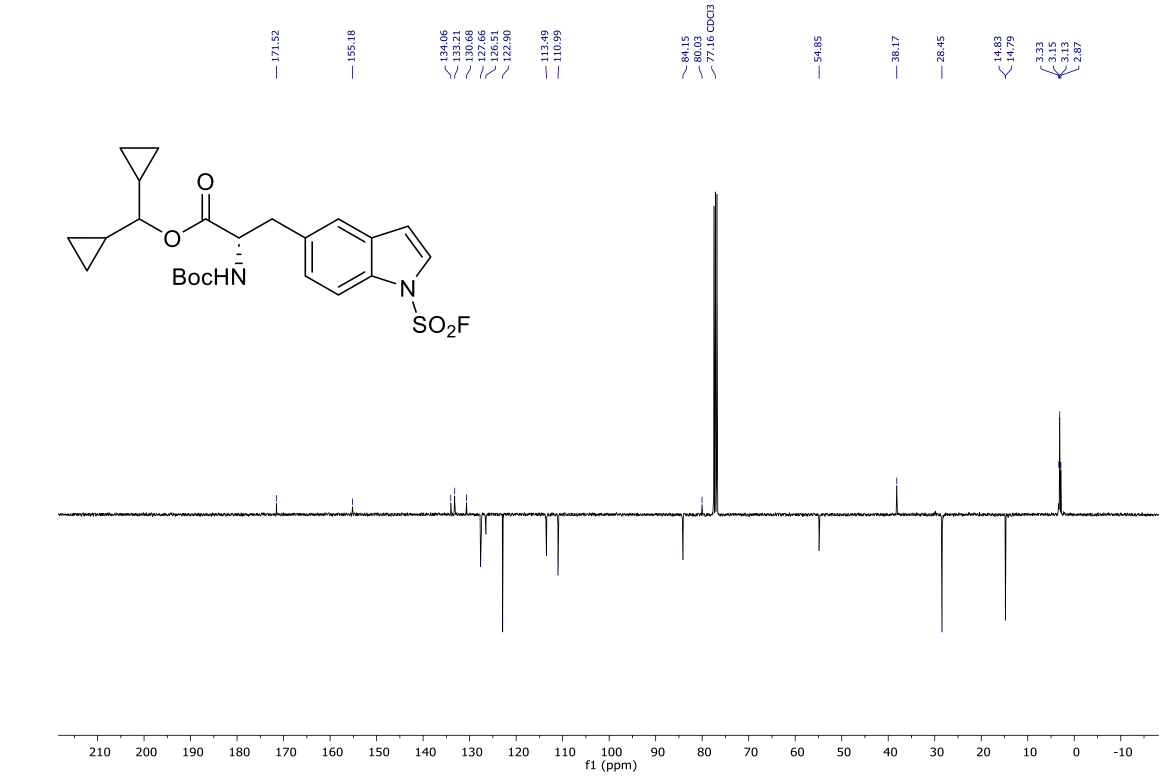

<u>^{19}F NMR</u>

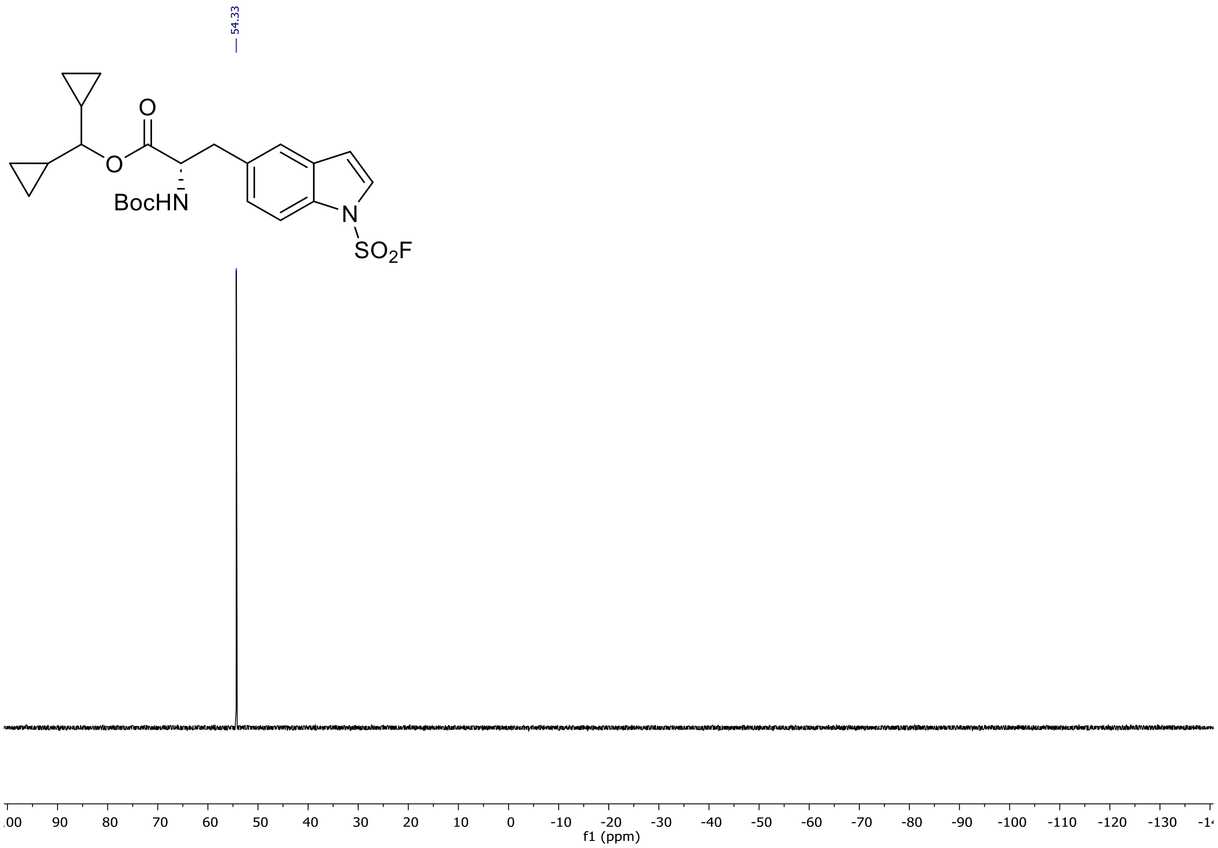

54.33
O
O
BocHN
N
SO2F
.00
90
80
70
60
50
40
30
20
10
0
-10
-20
-30
-40
-50
-60
-70
-80
-90
-100
-110
-120
-130
f1 (ppm)

(*S*)-2-Amino-3-[1-(fluorosulfonyl)-1*H*-indol-5-yl]propanoic acid hydrochloride (**26**·HCl)

<u>^{1}H NMR</u>

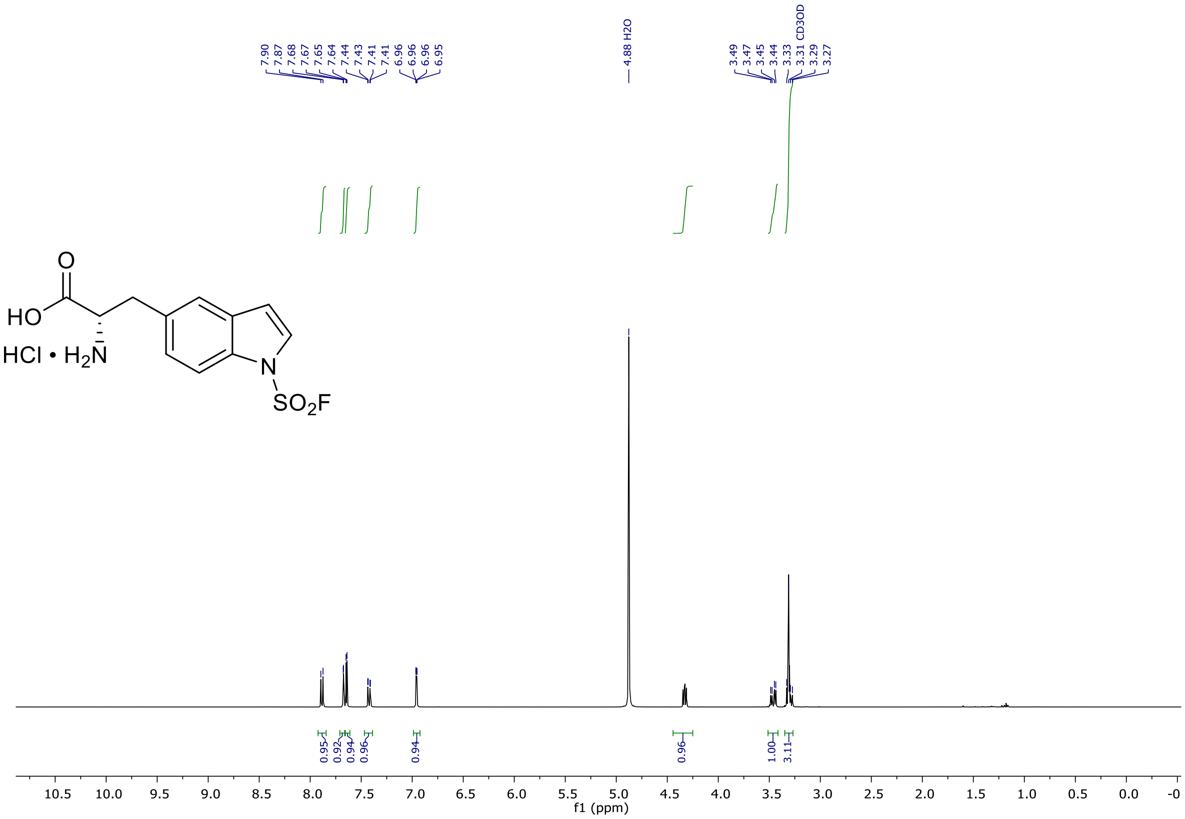

<u>^{13}C{^{1}H} NMR</u>

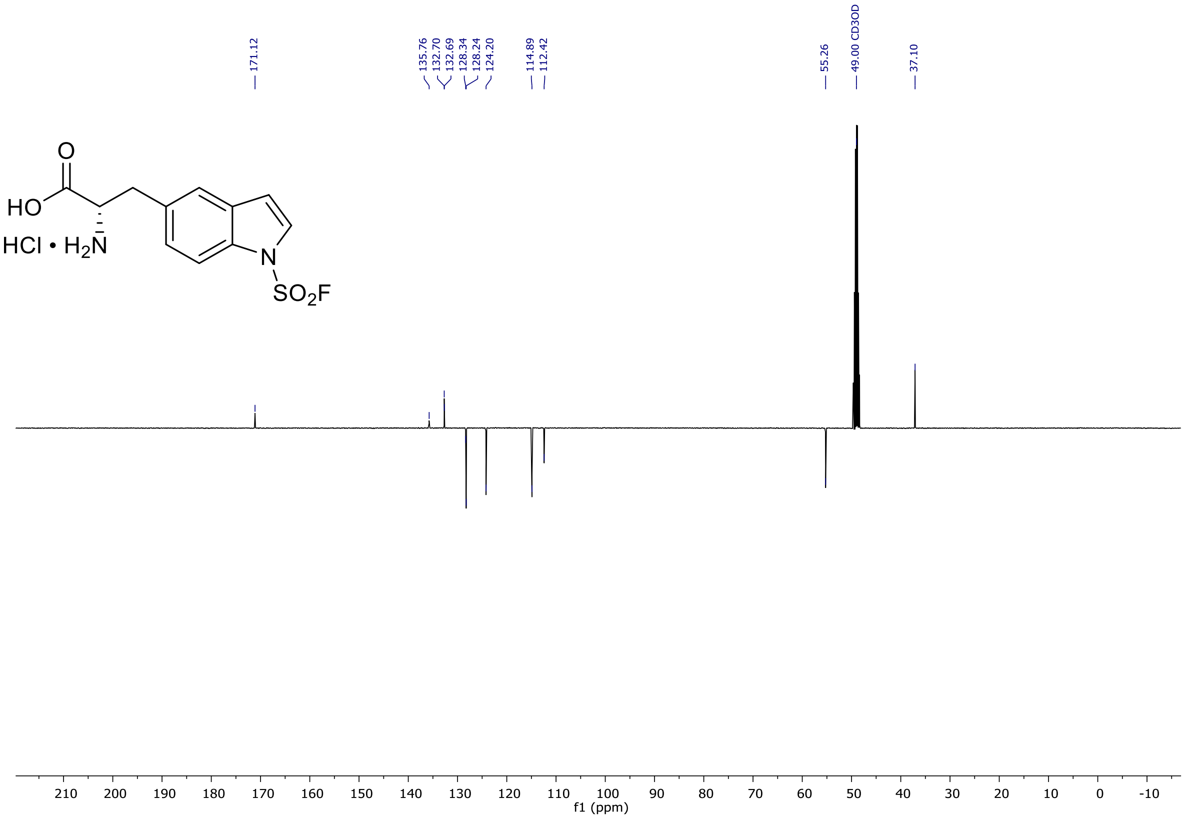

^{19}F NMR

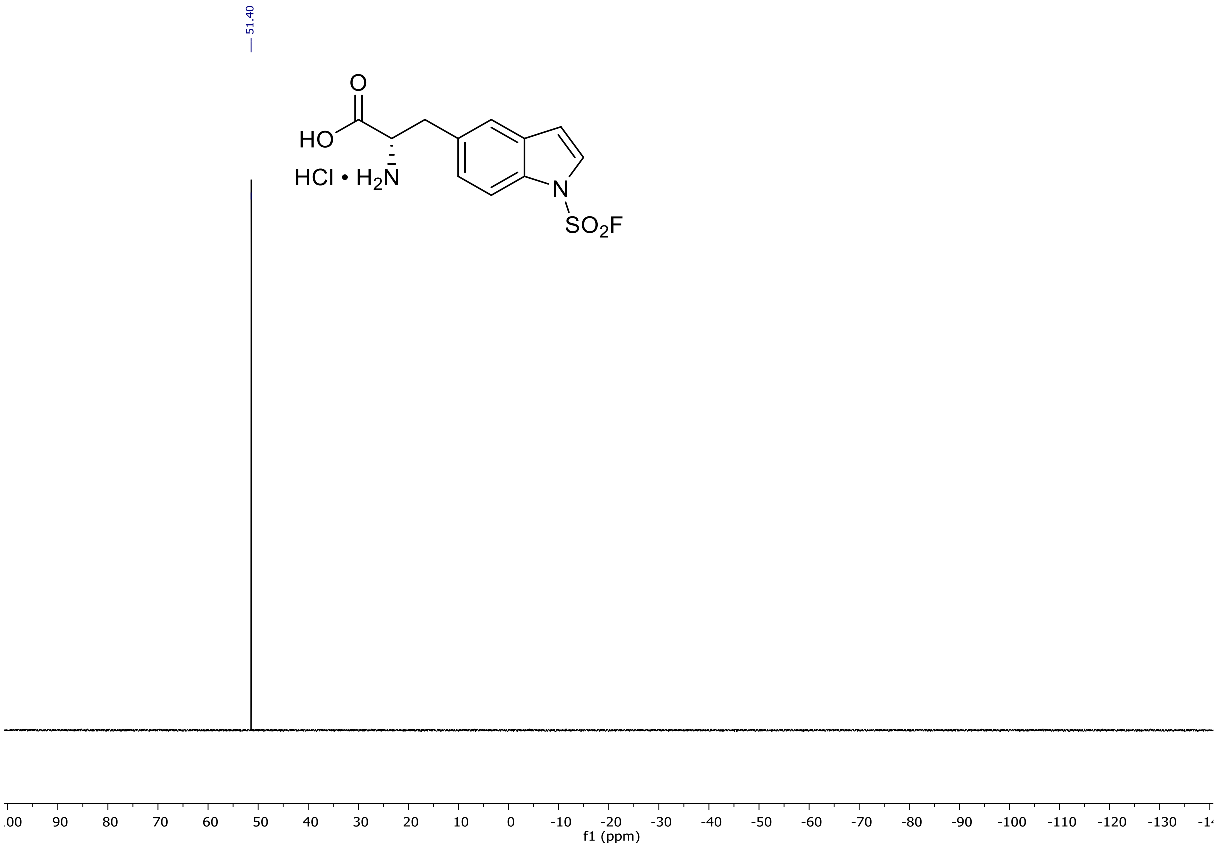

51.40
HO
O
HCl • H2N
N
SO2F
.00
90
80
70
60
50
40
30
20
10
0
-10
-20
-30
-40
-50
-60
-70
-80
-90
-100
-110
-120
-130
f1 (ppm)

4-(Methylamino)benzaldehyde (**27**)

^{1}H NMR

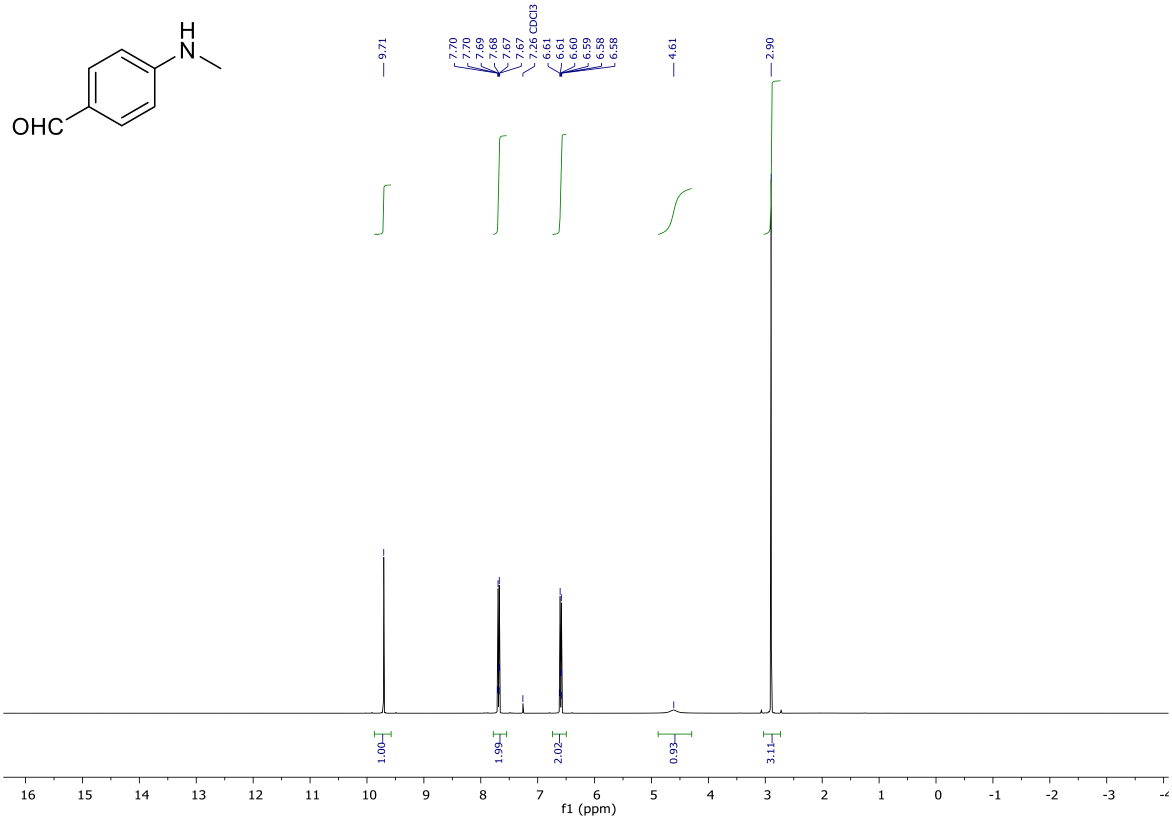

^{13}C{^{1}H} NMR

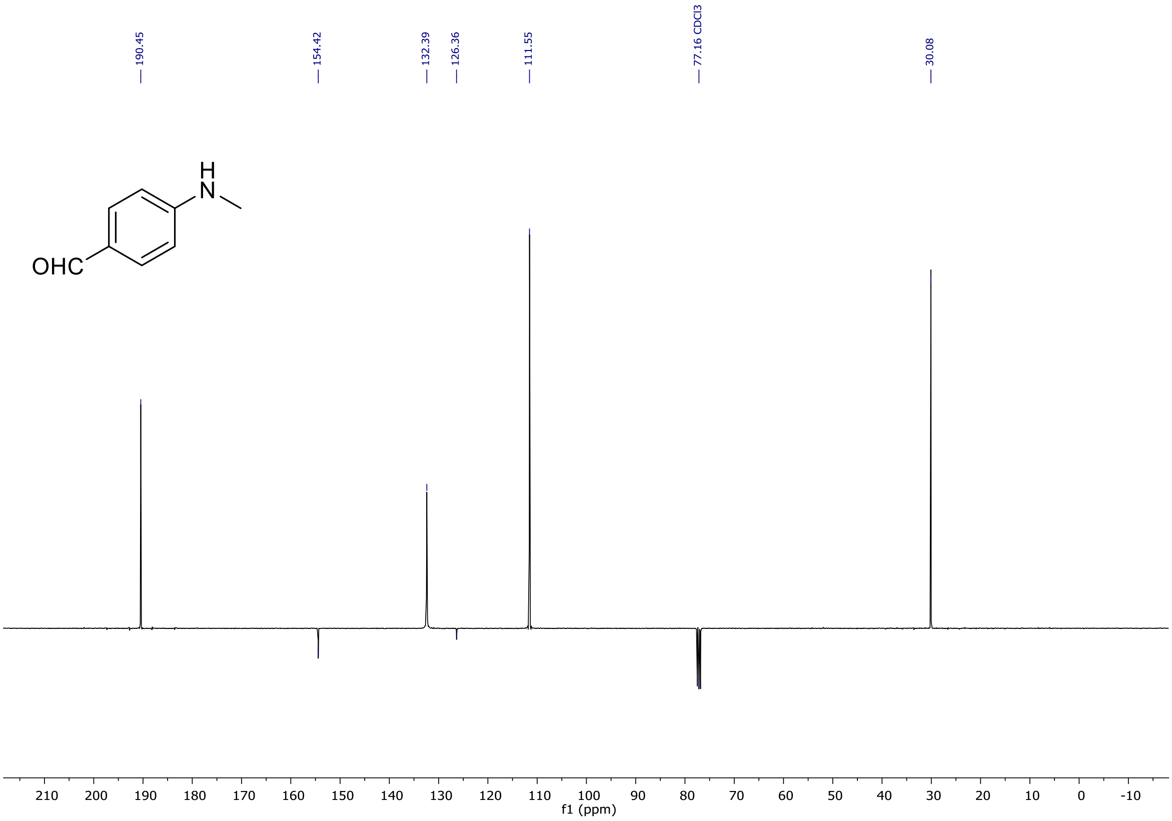

[4-(Methylamino)phenyl]methanol (**28**)

^{1}H NMR

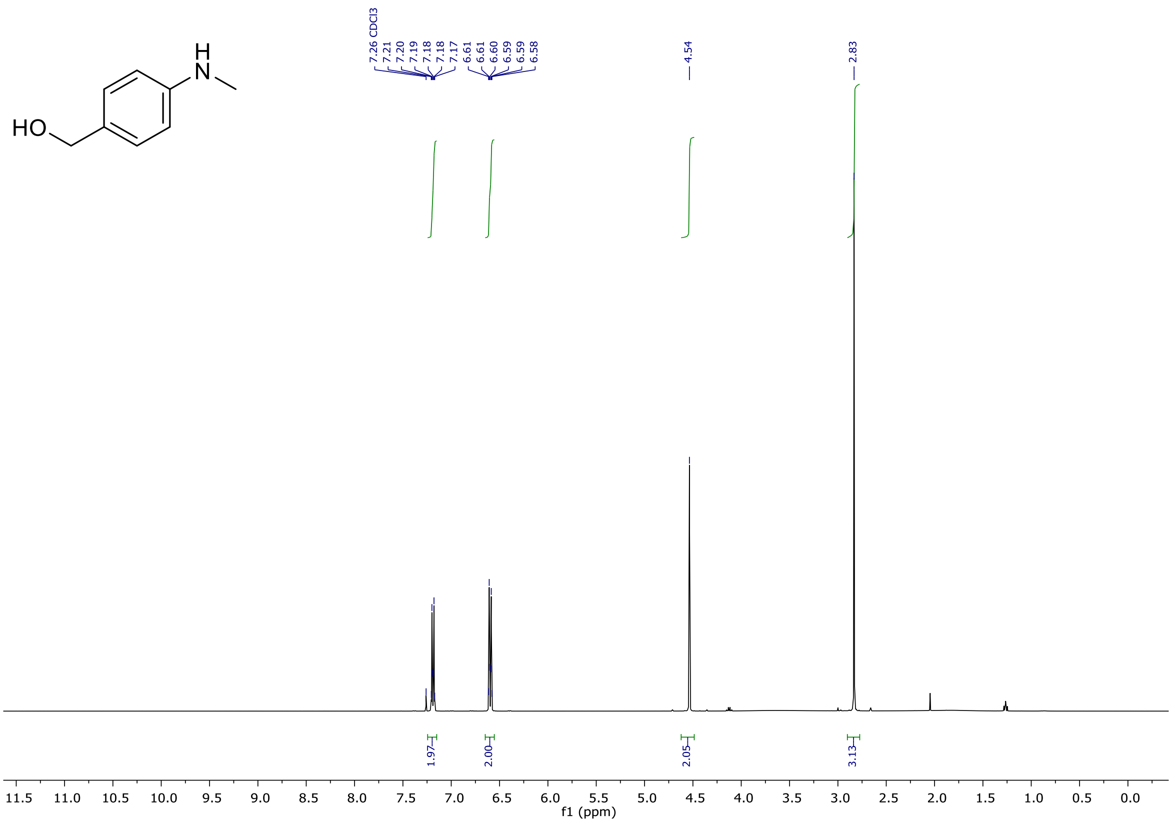

$^{13}C\{^{1}H\}$ NMR

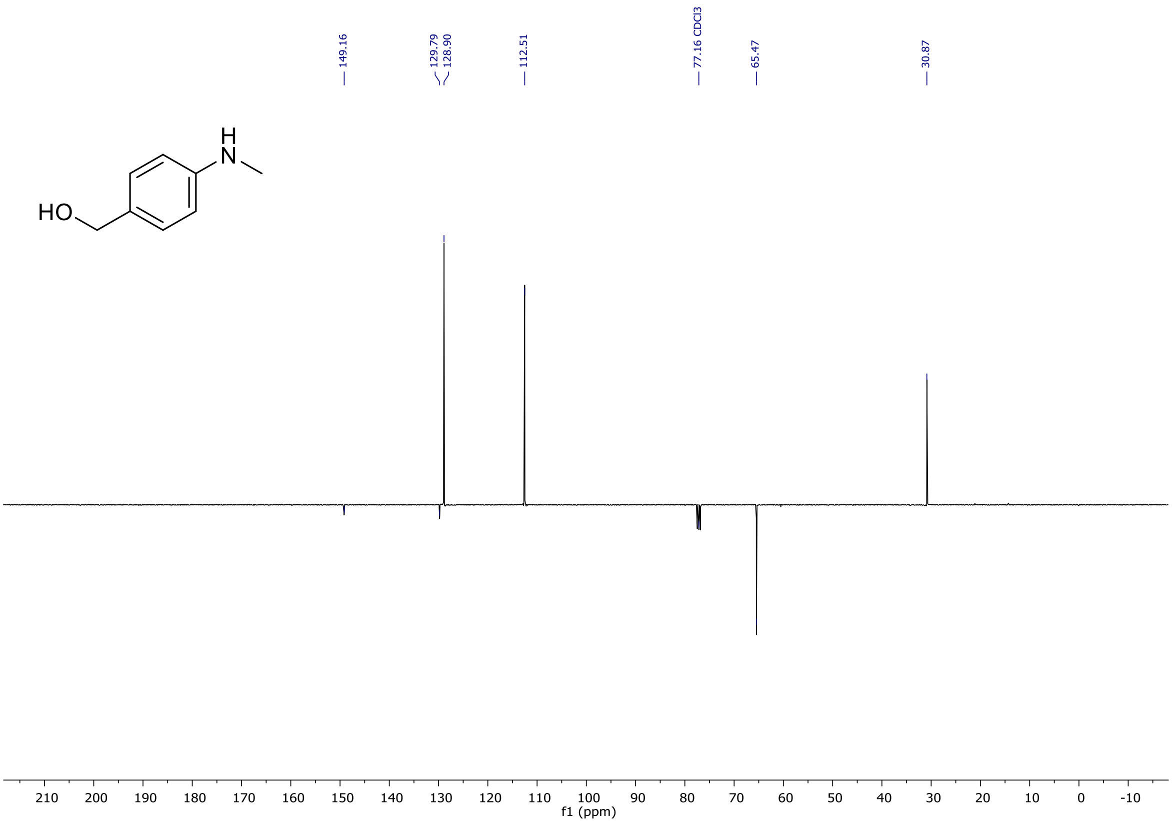

[4-(Bromomethyl)phenyl](methyl)sulfamoyl fluoride (**29**)

<u>^{1}H NMR</u>

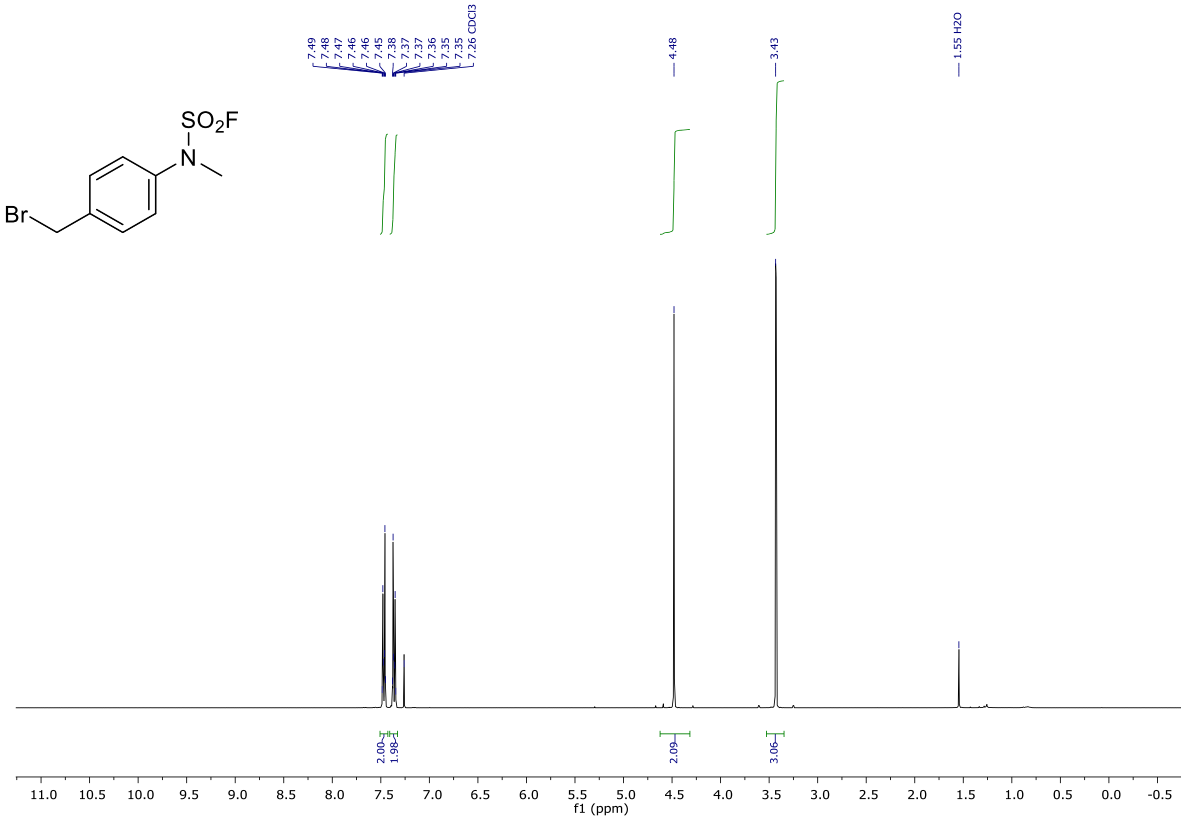

<u>^{13}C{^{1}H} NMR</u>

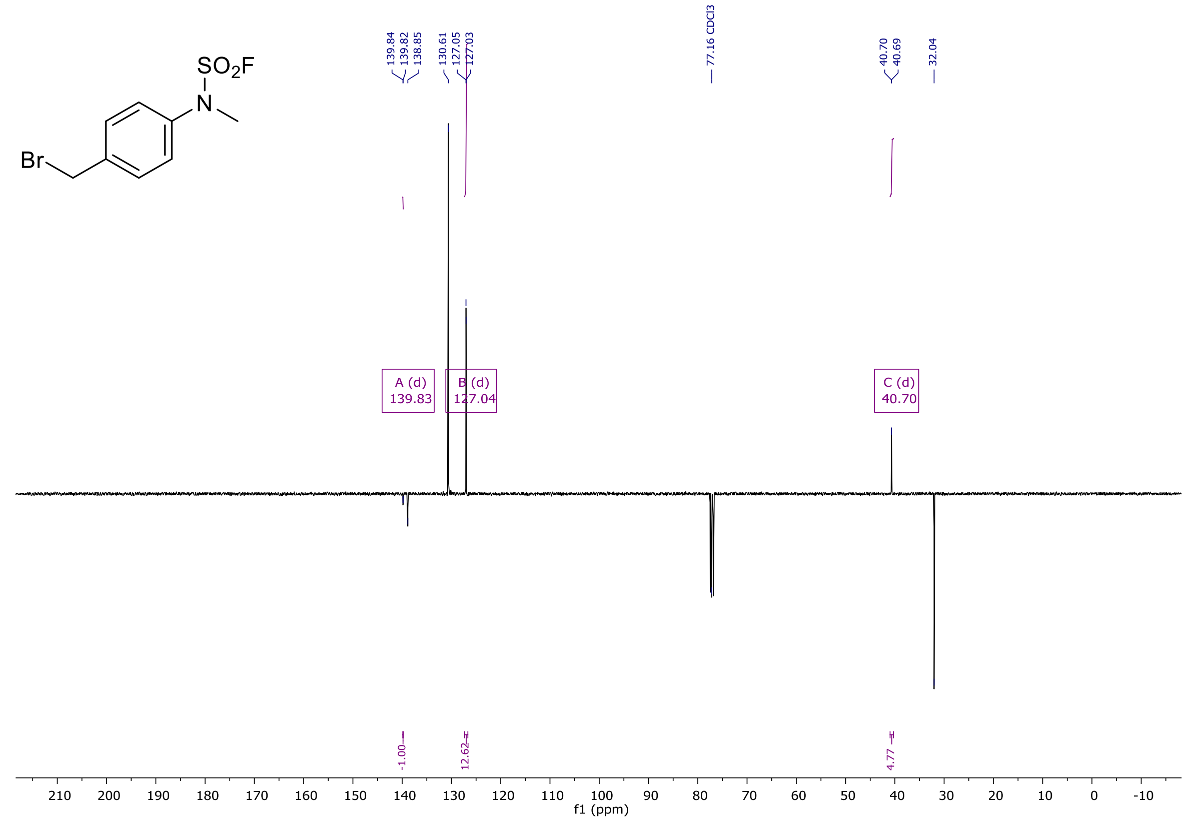

^{19}F NMR

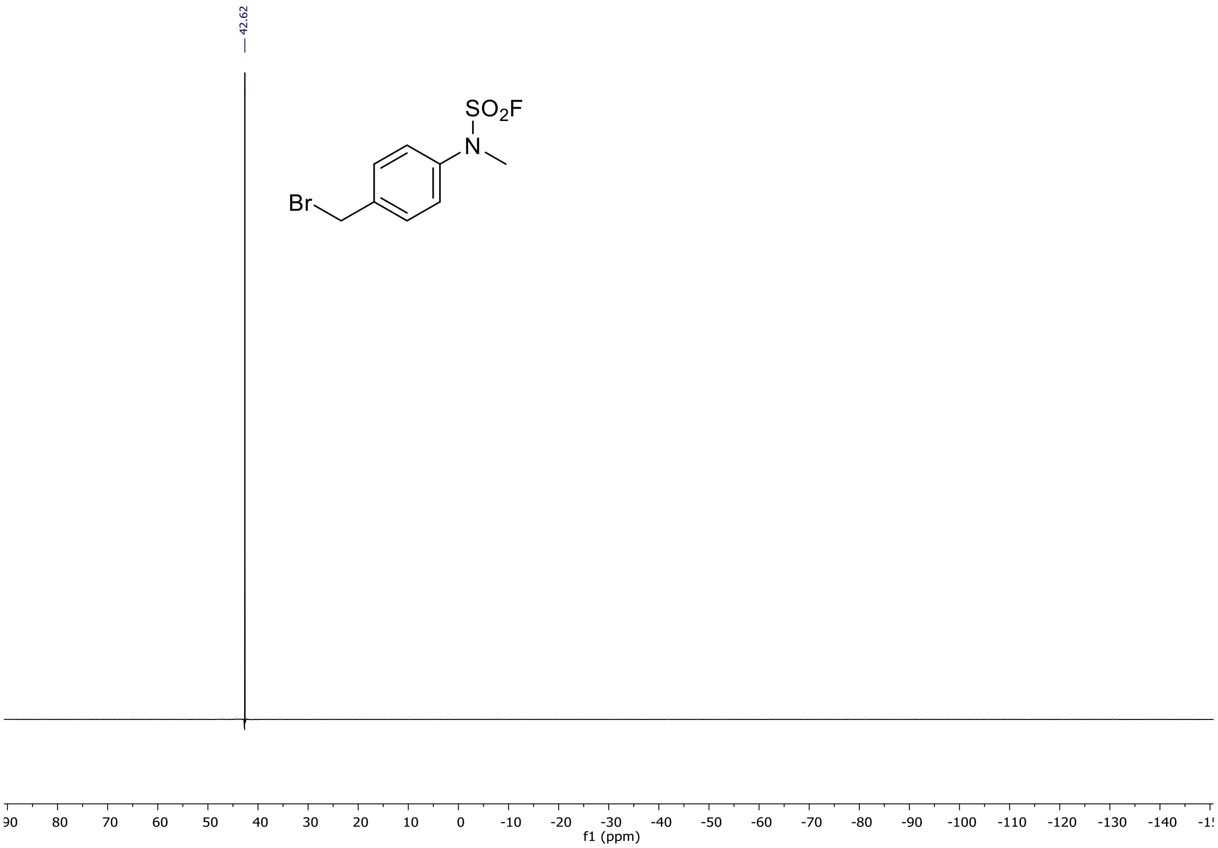

(*S*,*S*)-Ni-BPB-Phe(NMeSO_2F) (**30**)

^{1}H NMR

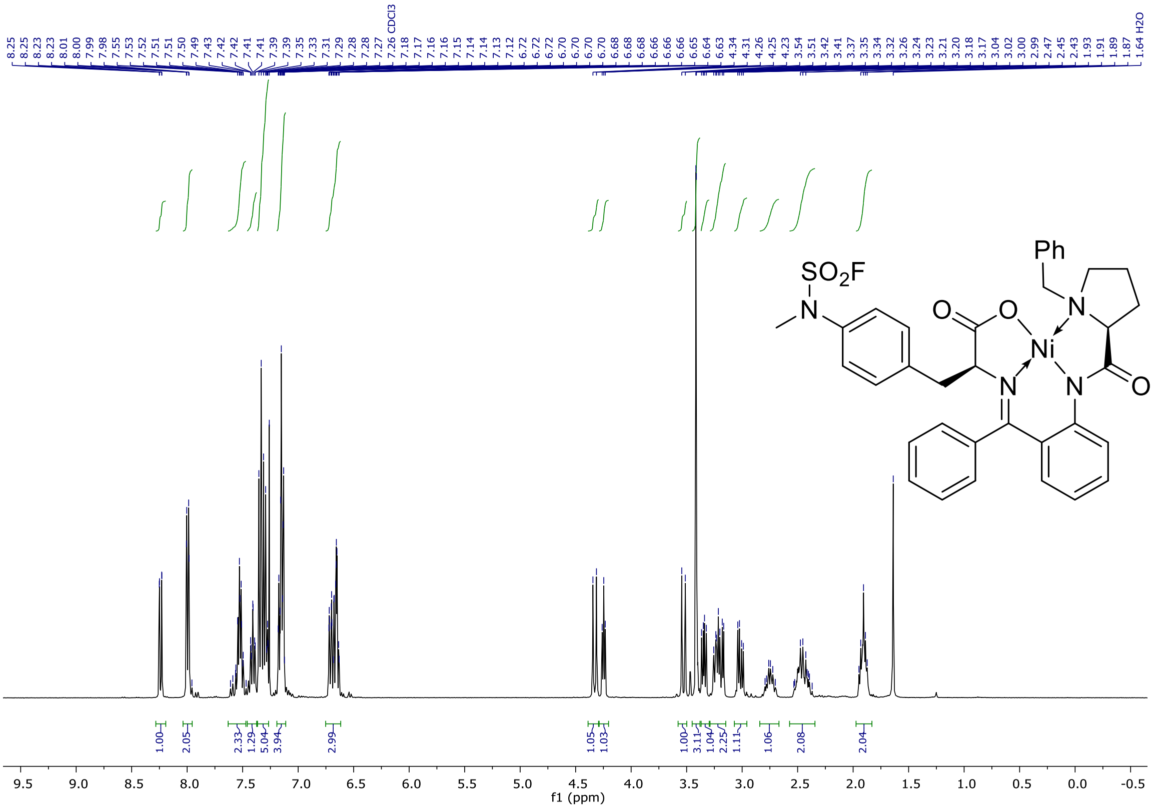

^{13}C{^{1}H} NMR

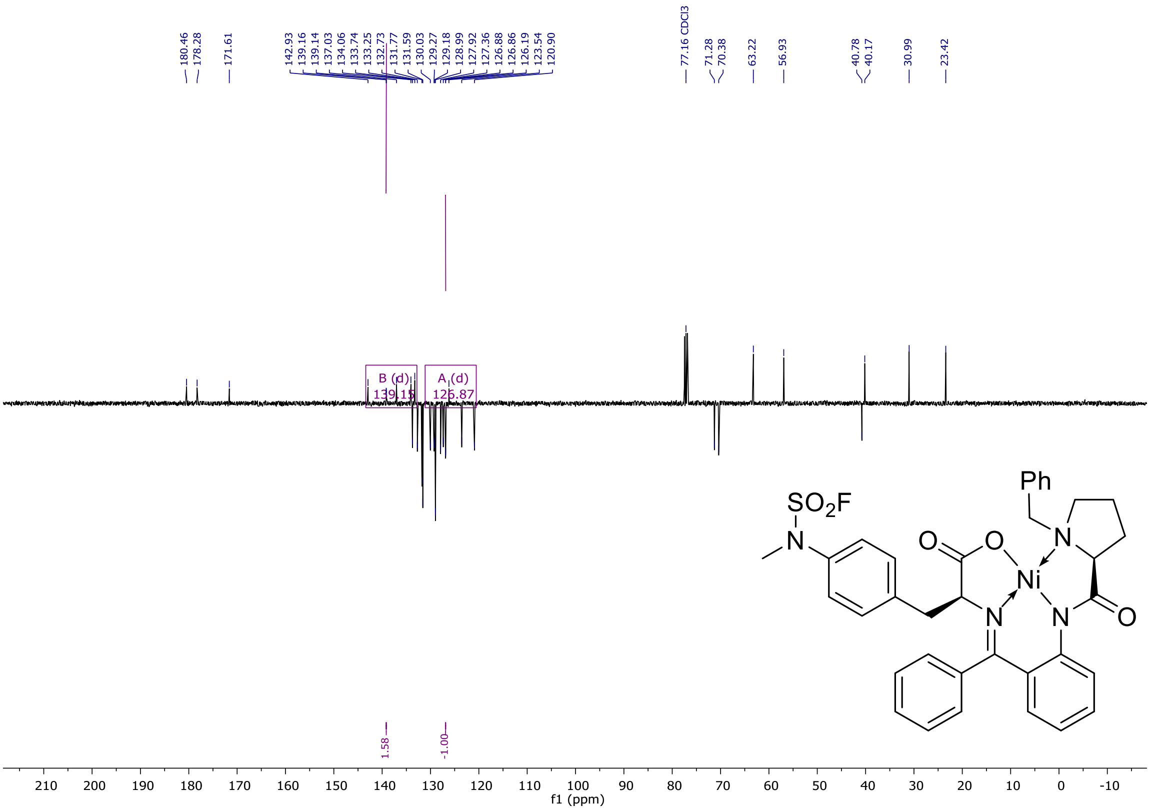

^{19}F NMR

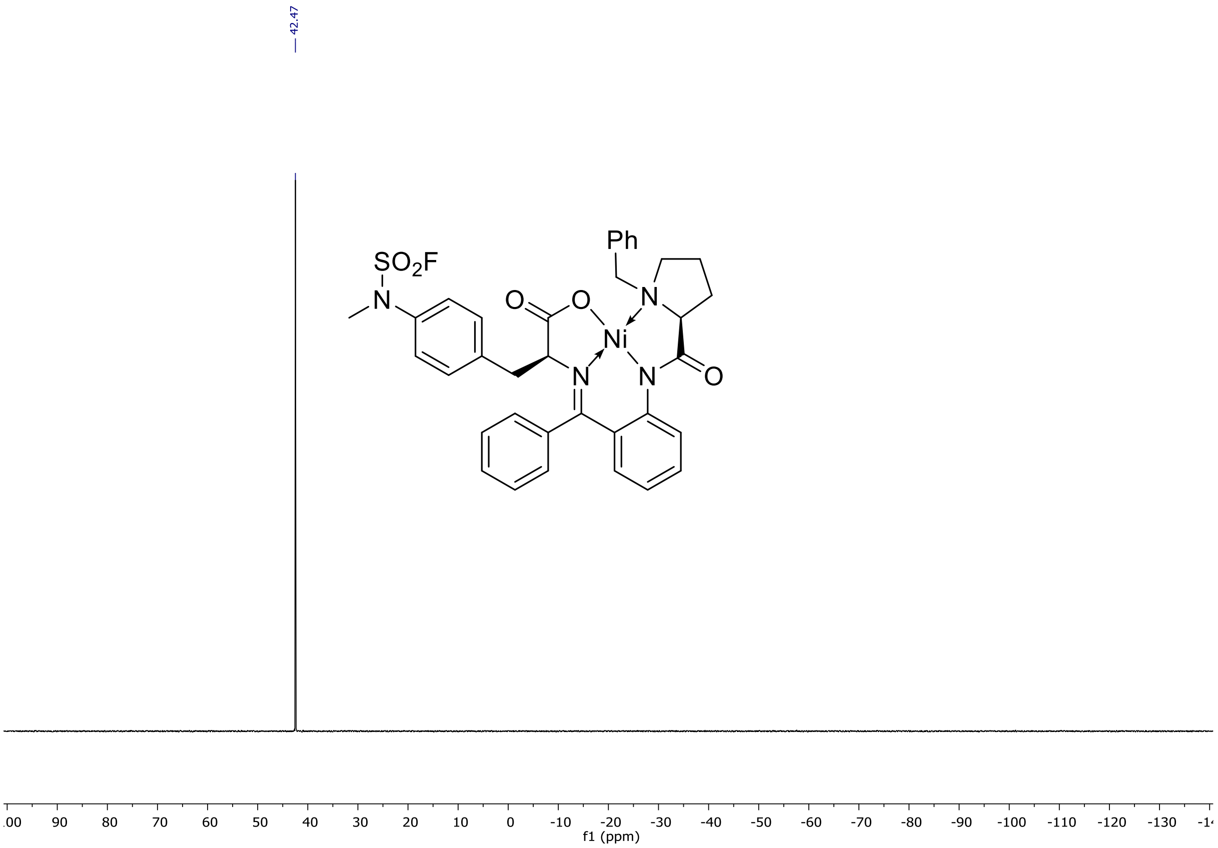

(*S*)-2-[(*tert*-Butoxycarbonyl)amino]-3-{4-[(fluorosulfonyl)(methyl)amino]phenyl} propanoic acid (**31**)

^{1}H NMR

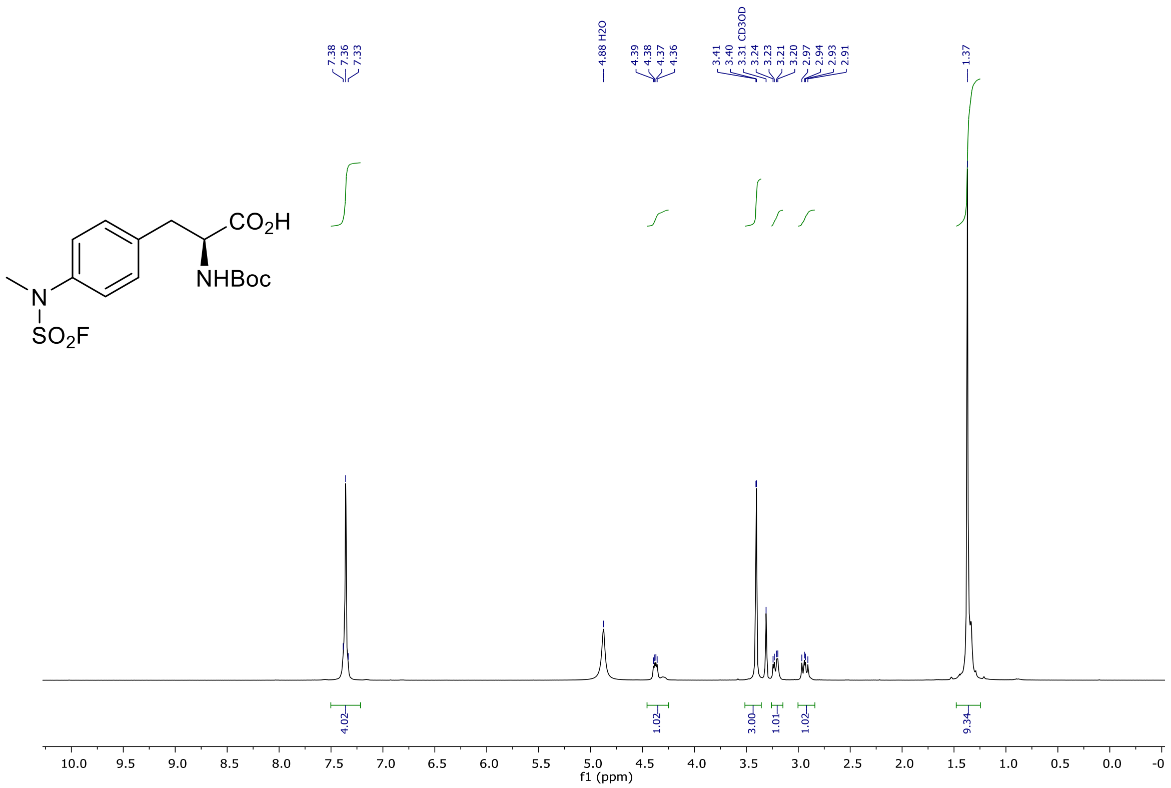

$^{13}C\{^{1}H\}$ NMR

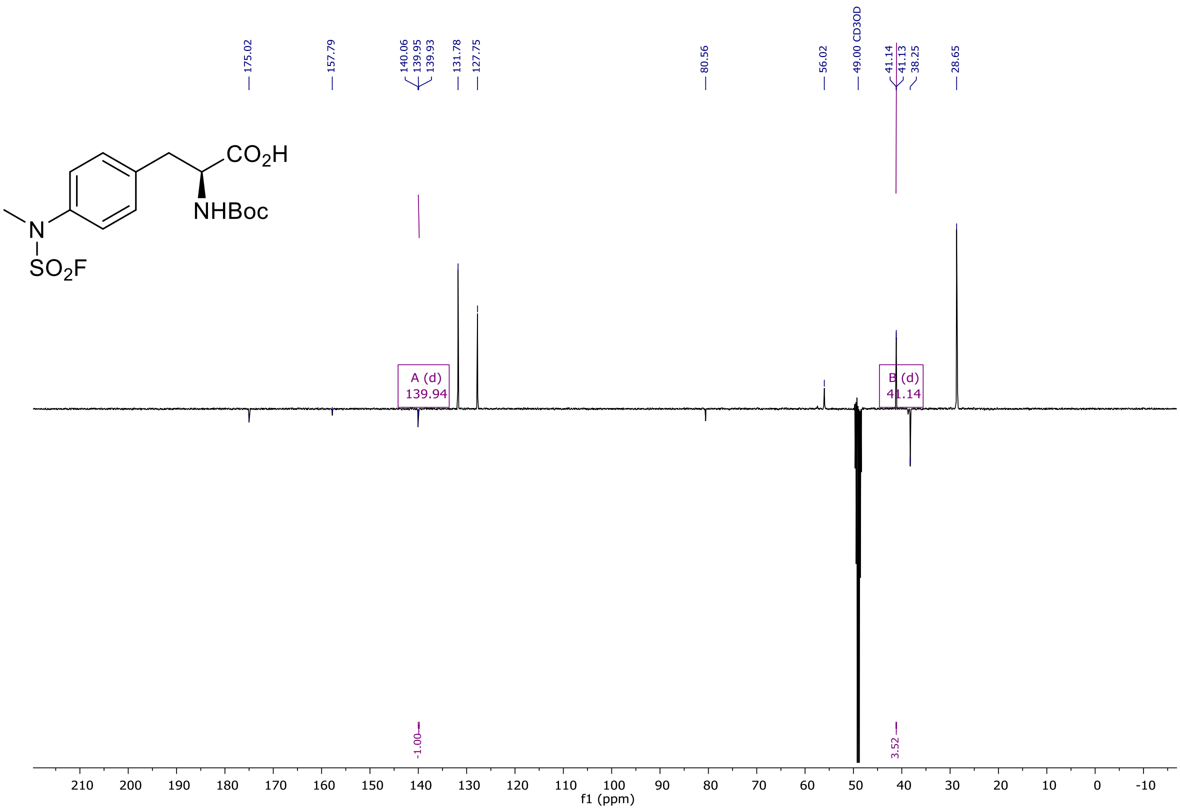

^{19}F NMR

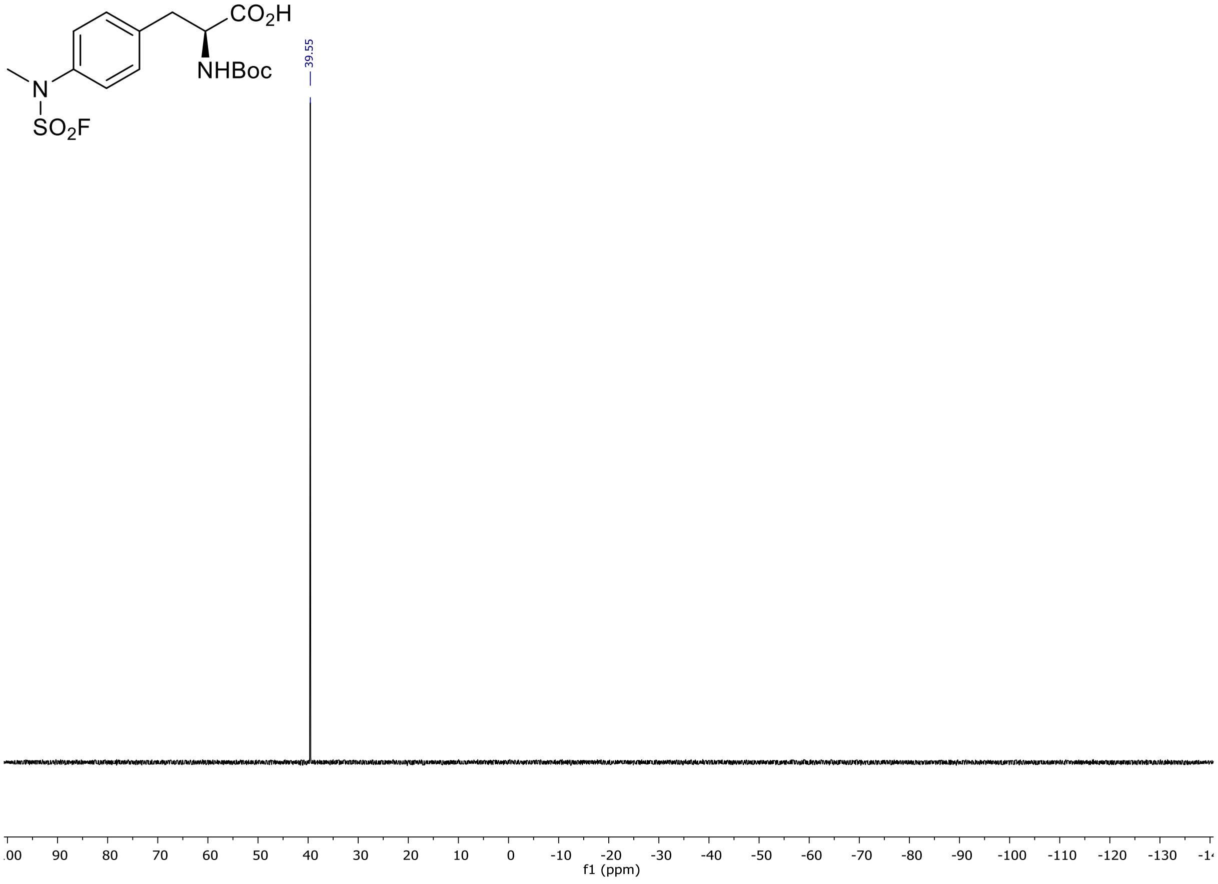

CO2H
NHBoc
N
SO2F
39.55
.00
90
80
70
60
50
40
30
20
10
0
-10
-20
-30
-40
-50
-60
-70
-80
-90
-100
-110
-120
-130
f1 (ppm)

tert-Butyl (*S*)-2-[(*tert*-butoxycarbonyl)amino]-3-{4-[(fluorosulfonyl)(methyl)amino]phenyl} propanoate (**32**)

^{1}H NMR

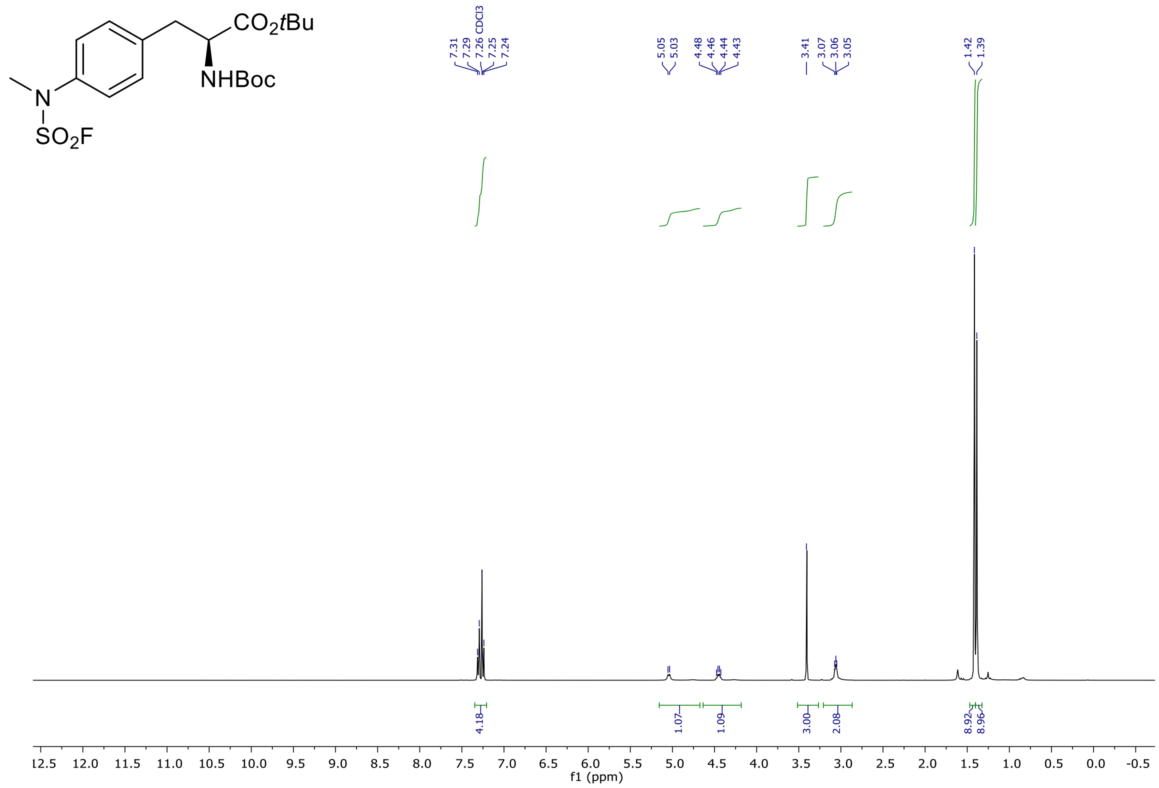

^{13}C{^{1}H} NMR

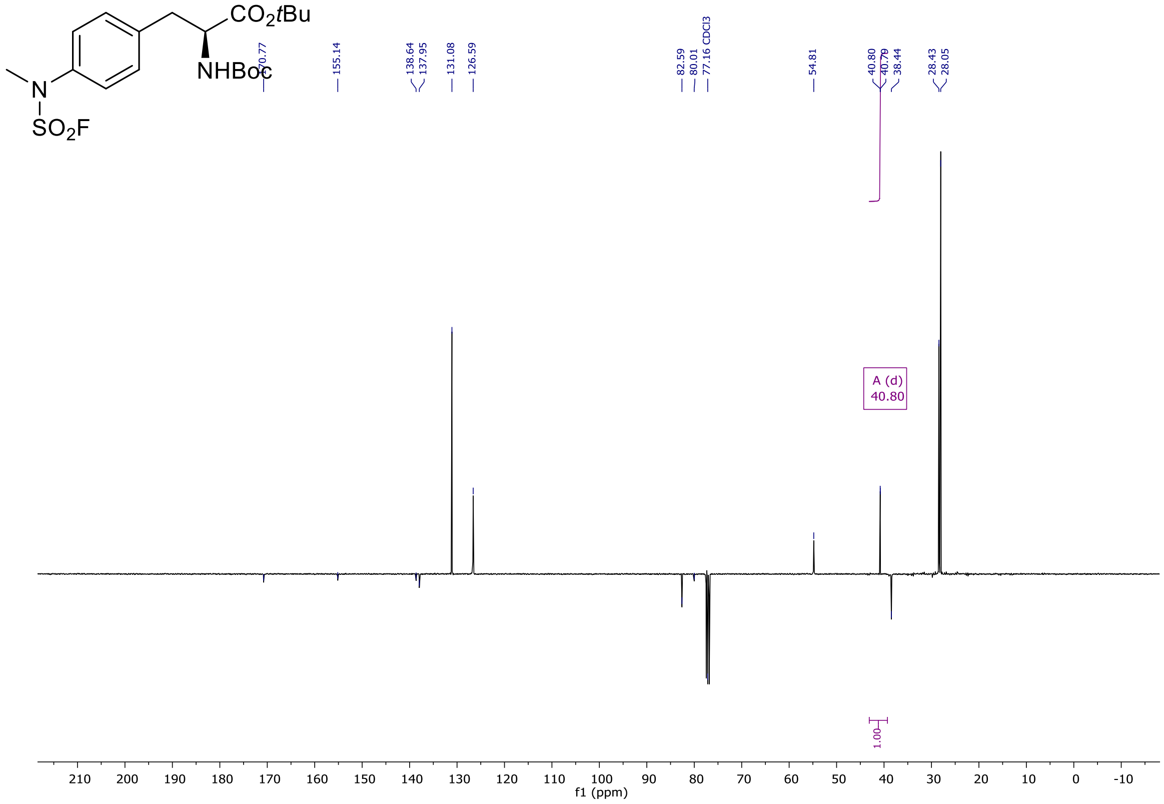

19F NMR

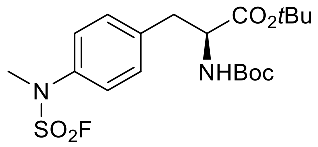

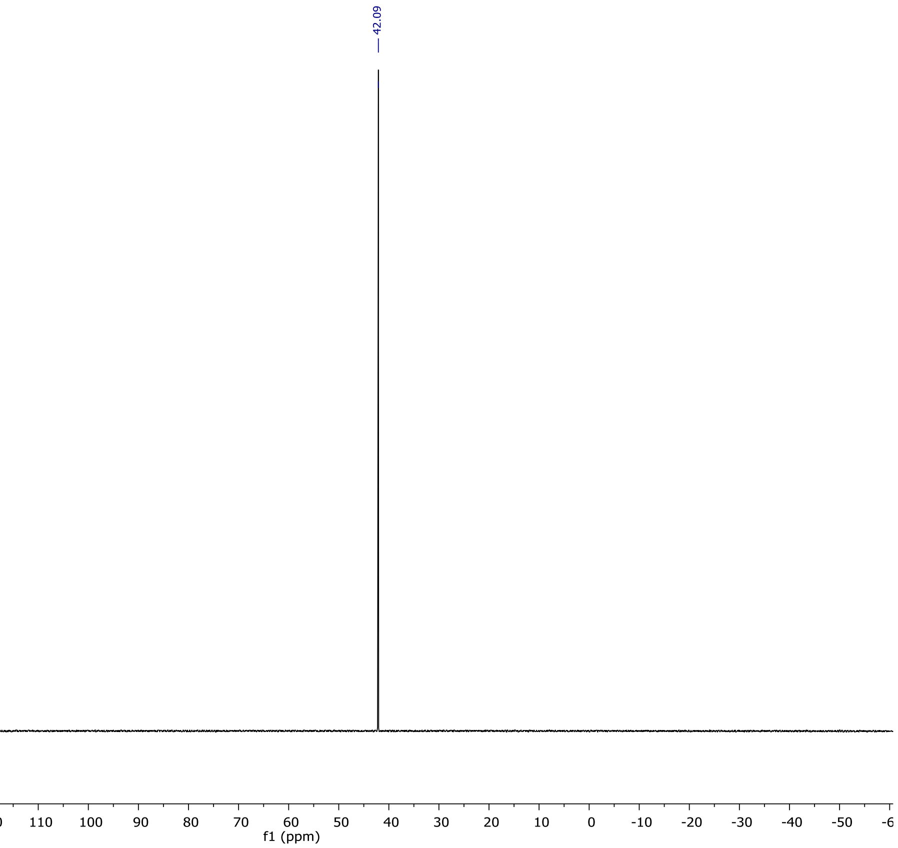
42.09
180
170
160
150
140
130
120
110
100
90
80
70
60
50
40
30
20
10
0
-10
-20
-30
-40
-50
-60
f1 (ppm)

(*S*)-2-Amino-3-{4-[(fluorosulfonyl)(methyl)amino]phenyl}propanoic acid hydrochloride (**33**·HCl)

^{1}H NMR

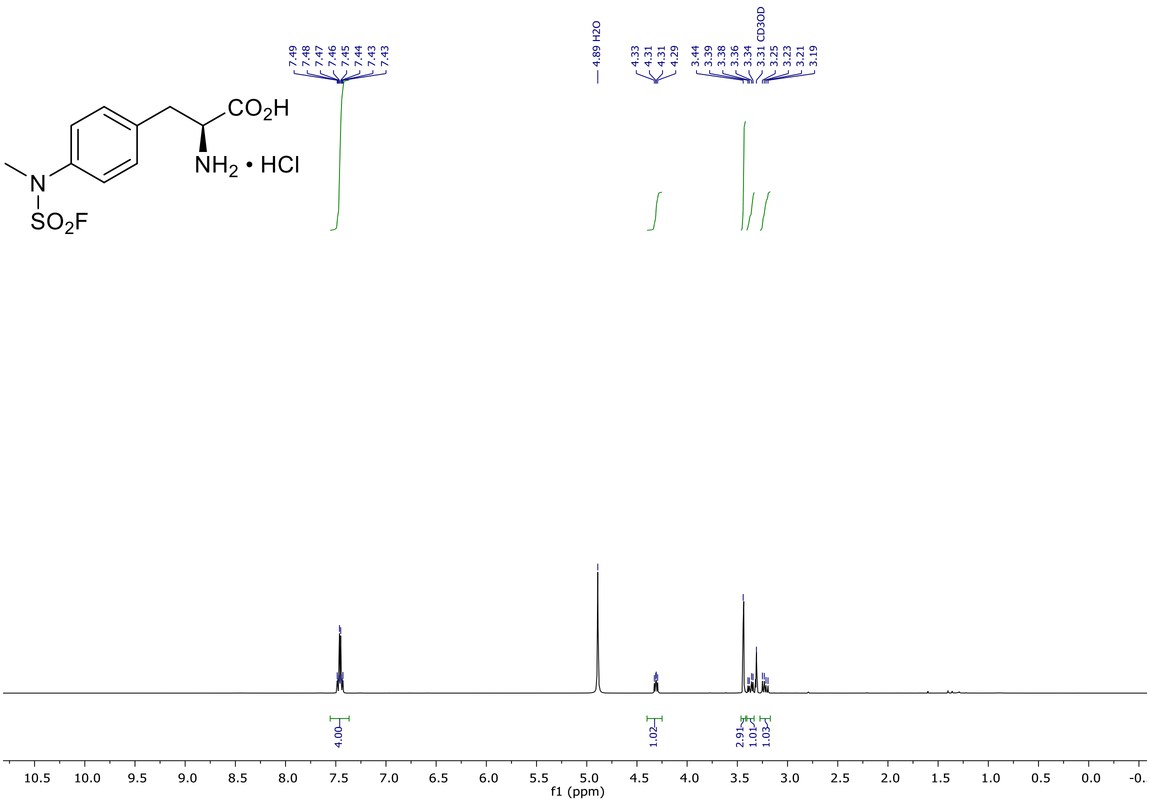

$^{13}C\{^{1}H\}$ NMR

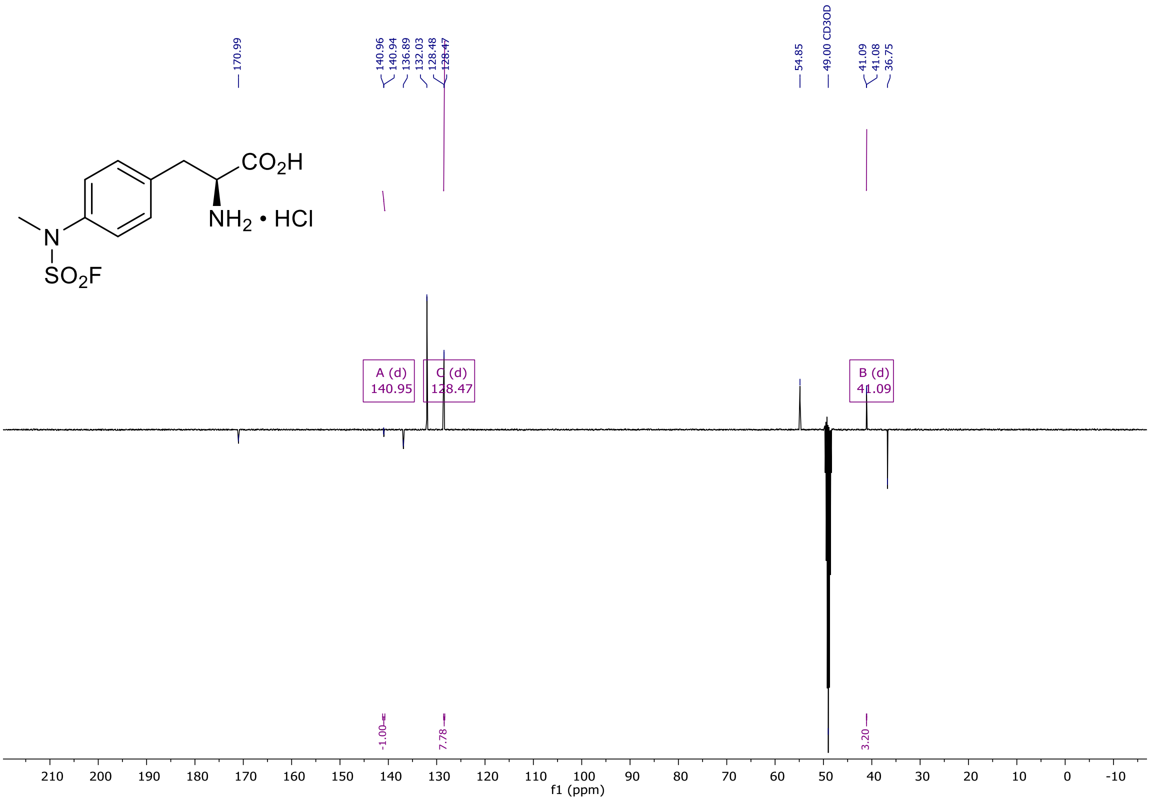

<u>^{19}F NMR</u>

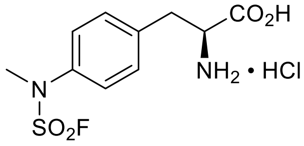

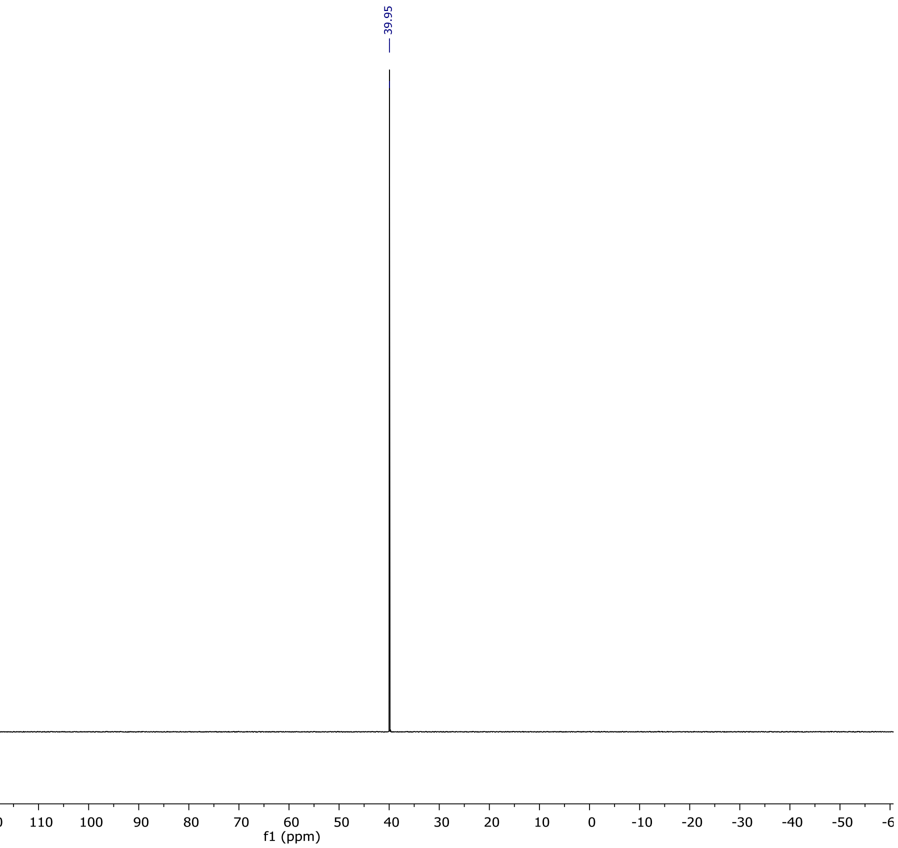
39.95
.80
170
160
150
140
130
120
110
100
90
80
70
60
50
40
30
20
10
0
-10
-20
-30
-40
-50
-6
f1 (ppm)

Chromatograms

N_α-(*tert*-Butoxycarbonyl)-1-([^{18}F]fluorosulfonyl)-L-tryptophan methyl ester ([^{18}F]**2**)

Column: Chromolith® SpeedROD RP-18e 50 × 4.6 mm (Merck KGaA, Darmstadt, Germany)

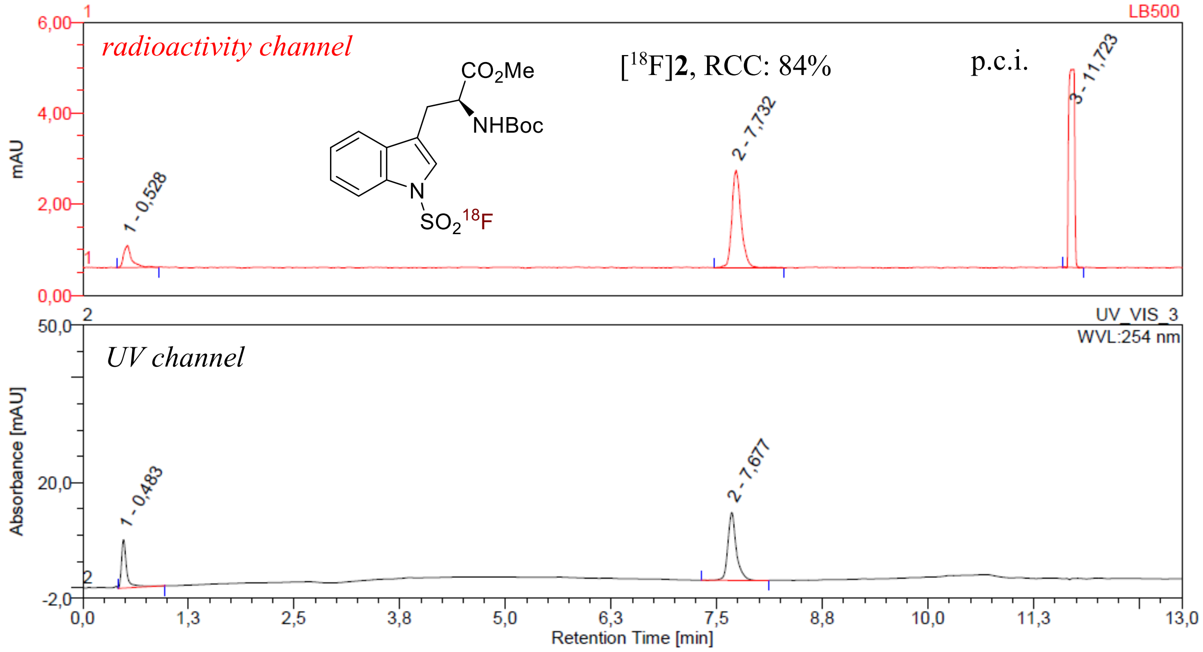

Eluent: 0–2 min: 10% MeCN, 2–10 min: 10 → 90% MeCN, 10–12.5 min: 90% MeCN, 12.5–13 min: 90 → 10% MeCN; flow-rate: 2 mL/min. Abbreviation: p.c.i. – post-column injection.

Indoline-1-sulfonyl [^{18}F]fluoride ([^{18}F]**3**)

Column: Chromolith® SpeedROD RP-18e 50 × 4.6 mm (Merck KGaA, Darmstadt, Germany)

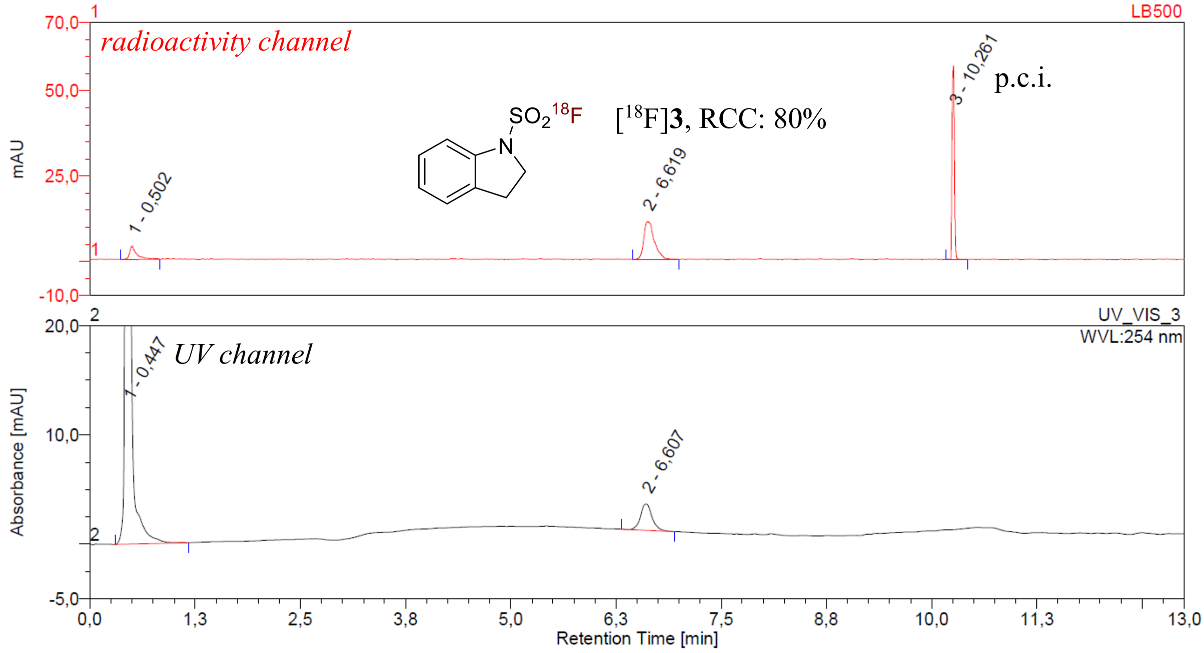

Eluent: 0–2 min: 10% MeCN, 2–10 min: 10 → 90% MeCN, 10–12.5 min: 90% MeCN, 12.5–13 min: 90 → 10% MeCN; flow-rate: 2 mL/min. Abbreviation: p.c.i. – post-column injection.

4-Phenylpiperidine-1-sulfonyl [^{18}F]fluoride ([^{18}F]**4**)

Column: Chromolith® SpeedROD RP-18e 50 × 4.6 mm (Merck KGaA, Darmstadt, Germany)

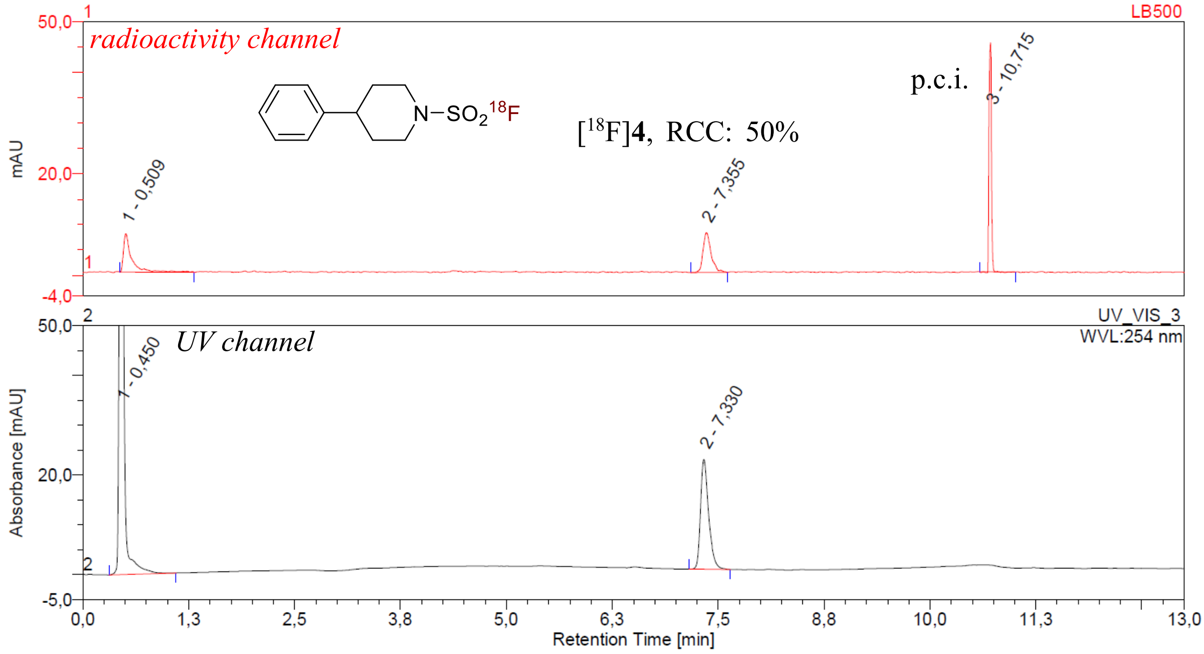

Eluent: 0–2 min: 10% MeCN, 2–10 min: 10 → 90% MeCN, 10–12.5 min: 90% MeCN, 12.5–13 min: 90 → 10% MeCN; flow-rate: 2 mL/min. Abbreviation: p.c.i. – post-column injection.

9*H*-Carbazole-9-sulfonyl [^{18}F]fluoride ([^{18}F]**5**)

Column: Chromolith® SpeedROD RP-18e 50 × 4.6 mm (Merck KGaA, Darmstadt, Germany)

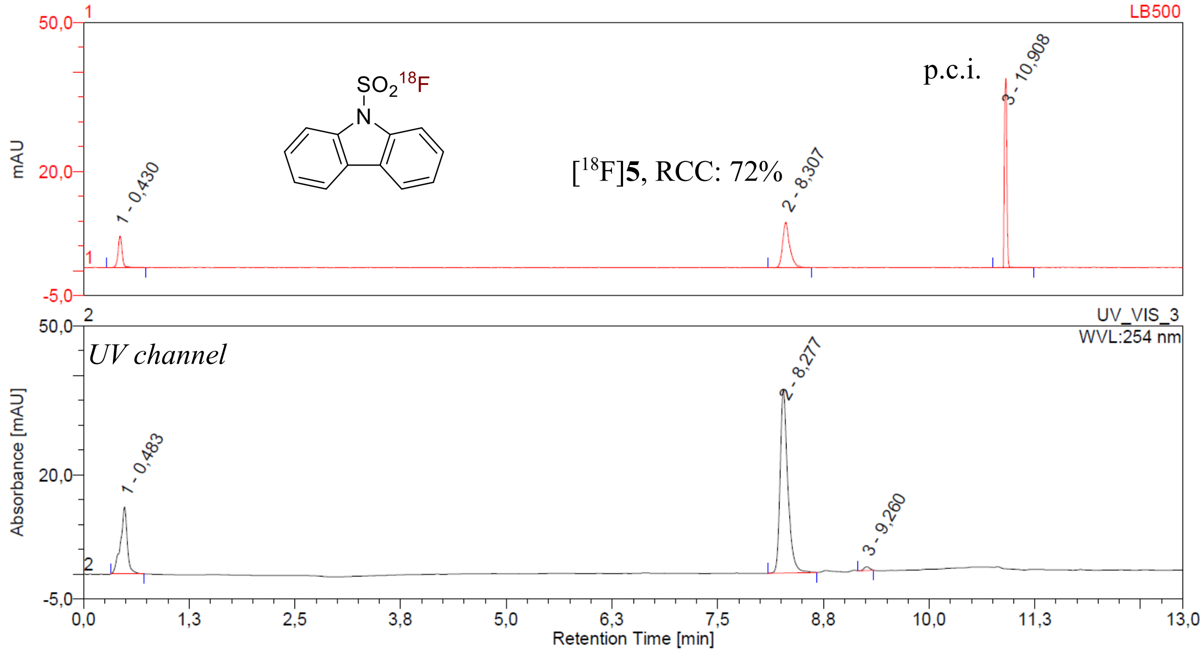

Eluent: 0–2 min: 10% MeCN, 2–10 min: 10 → 90% MeCN, 10–12.5 min: 90% MeCN, 12.5–13 min: 90 → 10% MeCN; flow-rate: 2 mL/min. Abbreviation: p.c.i. – post-column injection.

5-Methoxy-1*H*-indole-1-sulfonyl [^{18}F]fluoride ([^{18}F]**6**)

Column: Chromolith® SpeedROD RP-18e 50 × 4.6 mm (Merck KGaA, Darmstadt, Germany)

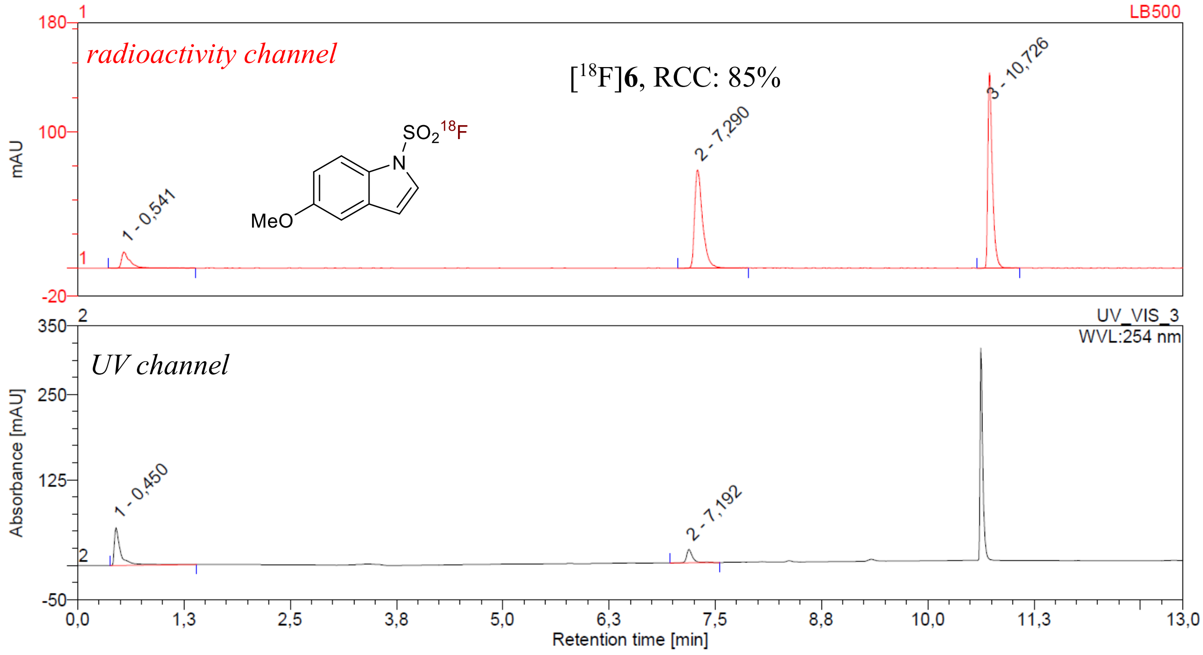

Eluent: 0–2 min: 10% MeCN, 2–10 min: 10 → 90% MeCN, 10–12.5 min: 90% MeCN, 12.5–13 min: 90 → 10% MeCN; flow-rate: 2 mL/min. Abbreviation: p.c.i. – post-column injection.

5-Formyl-1*H*-indole-1-sulfonyl [^{18}F]fluoride ([^{18}F]**7**)

Column: Chromolith® SpeedROD RP-18e 50 × 4.6 mm (Merck KGaA, Darmstadt, Germany)

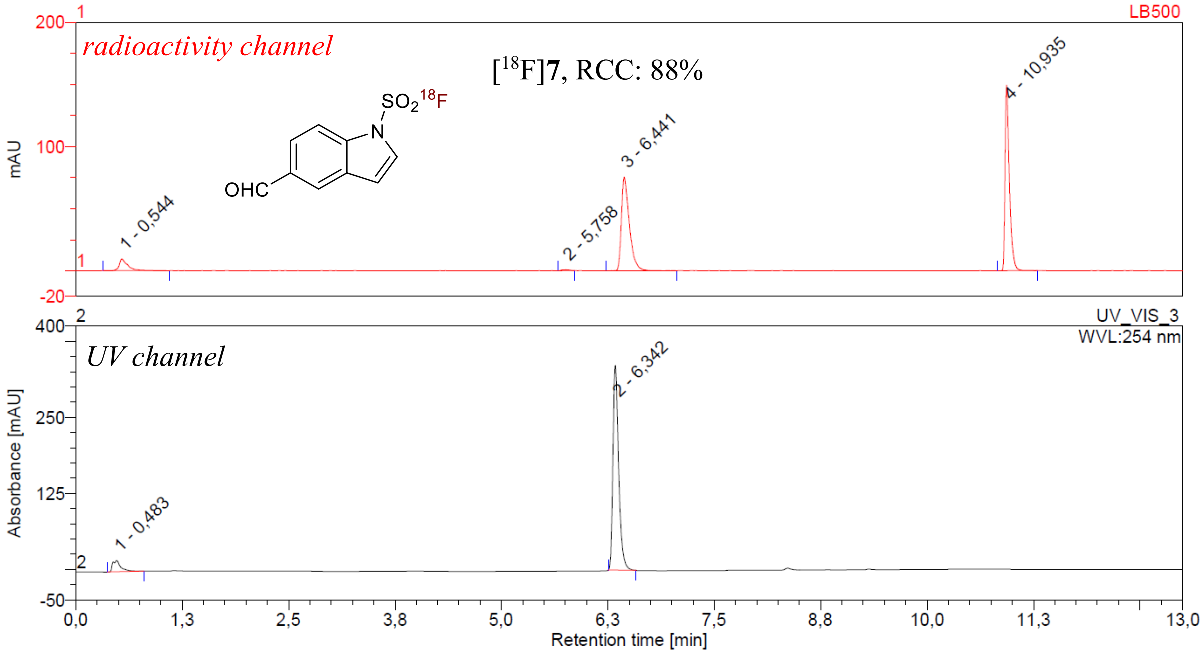

Eluent: 0–2 min: 10% MeCN, 2–10 min: 10 → 90% MeCN, 10–12.5 min: 90% MeCN, 12.5–13 min: 90 → 10% MeCN; flow-rate: 2 mL/min. Abbreviation: p.c.i. – post-column injection.

1*H*-Indole-1-sulfonyl [18F]fluoride ([18F]**8**)

Column: Chromolith® SpeedROD RP-18e 50 × 4.6 mm (Merck KGaA, Darmstadt, Germany)

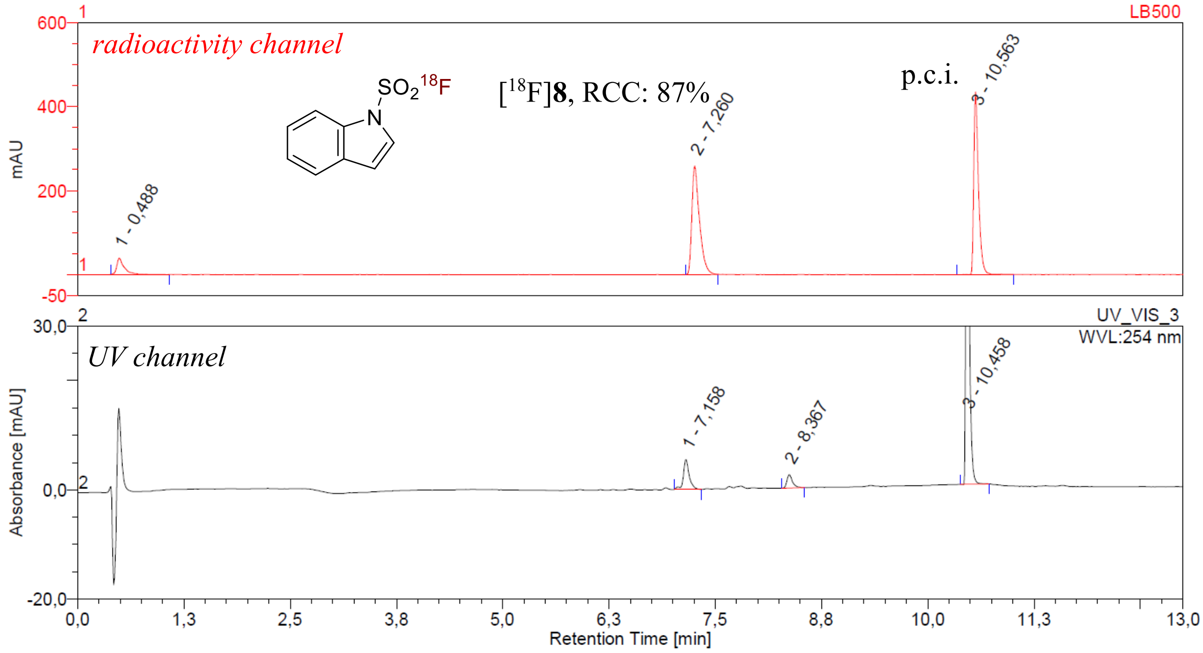

Eluent: 0–2 min: 10% MeCN, 2–10 min: 10 → 90% MeCN, 10–12.5 min: 90% MeCN, 12.5–13 min: 90 → 10% MeCN; flow-rate: 2 mL/min. Abbreviation: p.c.i. – post-column injection.

Methyl(phenyl)sulfamoyl [18F]fluoride ([18F]**9**)

Column: Chromolith® SpeedROD RP-18e 50 × 4.6 mm (Merck KGaA, Darmstadt, Germany)

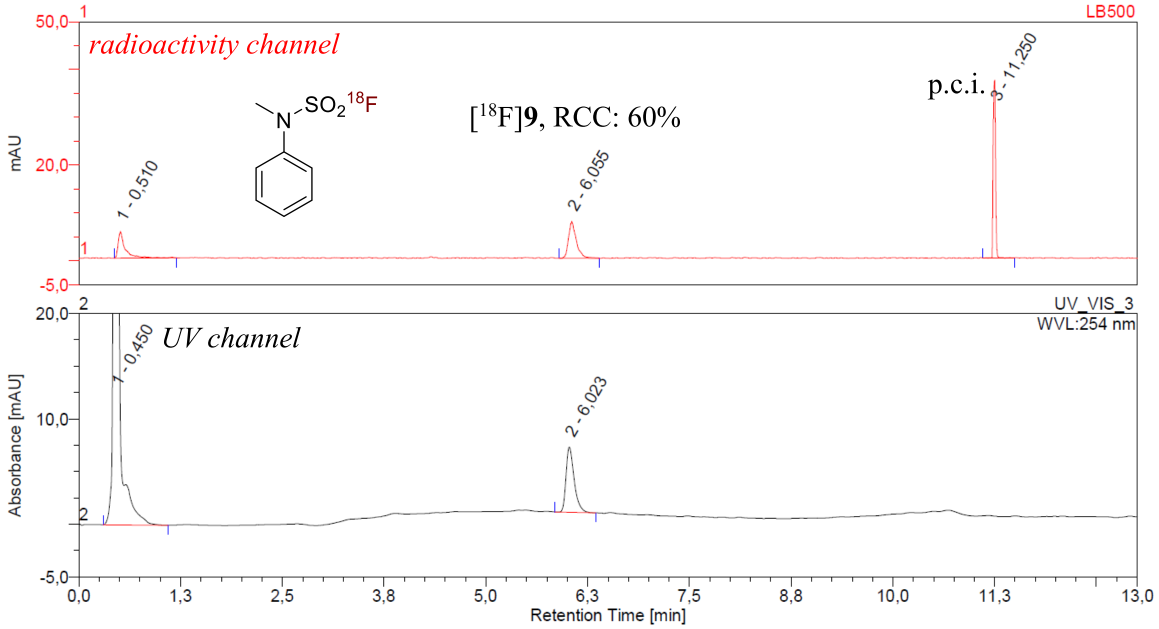

Eluent: 0–2 min: 10% MeCN, 2–10 min: 10 → 90% MeCN, 10–12.5 min: 90% MeCN, 12.5–13 min: 10 → 90% MeCN; flow-rate: 2 mL/min. Abbreviation: p.c.i. – post-column injection.

(4-Methoxyphenyl)(methyl)sulfamoyl [^{18}F]fluoride ([^{18}F]**10**)

Column: Chromolith® SpeedROD RP-18e 50 × 4.6 mm (Merck KGaA, Darmstadt, Germany)

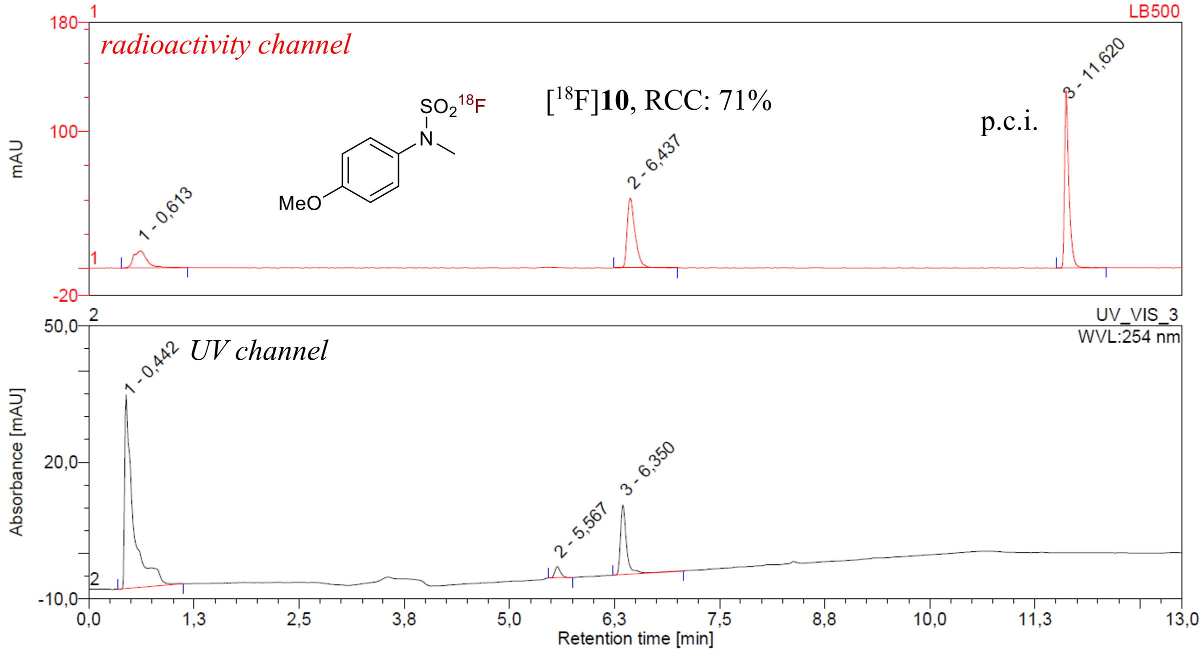

Eluent: 0–2 min: 10% MeCN, 2–10 min: 10 → 90% MeCN, 10–12.5 min: 90% MeCN, 12.5–13 min: 90 → 10% MeCN; flow-rate: 2 mL/min. Abbreviation: p.c.i. – post-column injection.

[4-(Hydroxymethyl)phenyl](methyl)sulfamoyl [^{18}F]fluoride ([^{18}F]**11**)

Column: Chromolith® SpeedROD RP-18e 50 × 4.6 mm (Merck KGaA, Darmstadt, Germany)

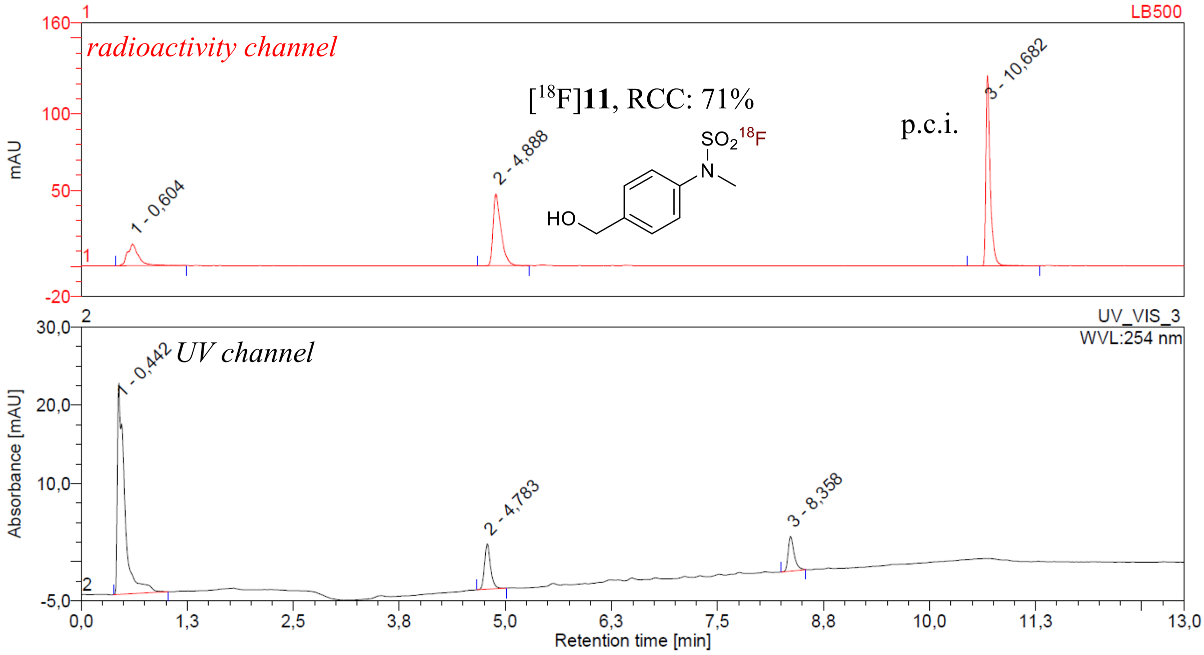

Eluent: 0–2 min: 10% MeCN, 2–10 min: 10 → 90% MeCN, 10–12.5 min: 90% MeCN, 12.5–13 min: 10 → 90% MeCN; flow-rate: 2 mL/min. Abbreviation: p.c.i. – post-column injection.

(4-Formylphenyl)(methyl)sulfamoyl [^{18}F]fluoride ([^{18}F]**12**)

Column: Chromolith® SpeedROD RP-18e 50 × 4.6 mm (Merck KGaA, Darmstadt, Germany)

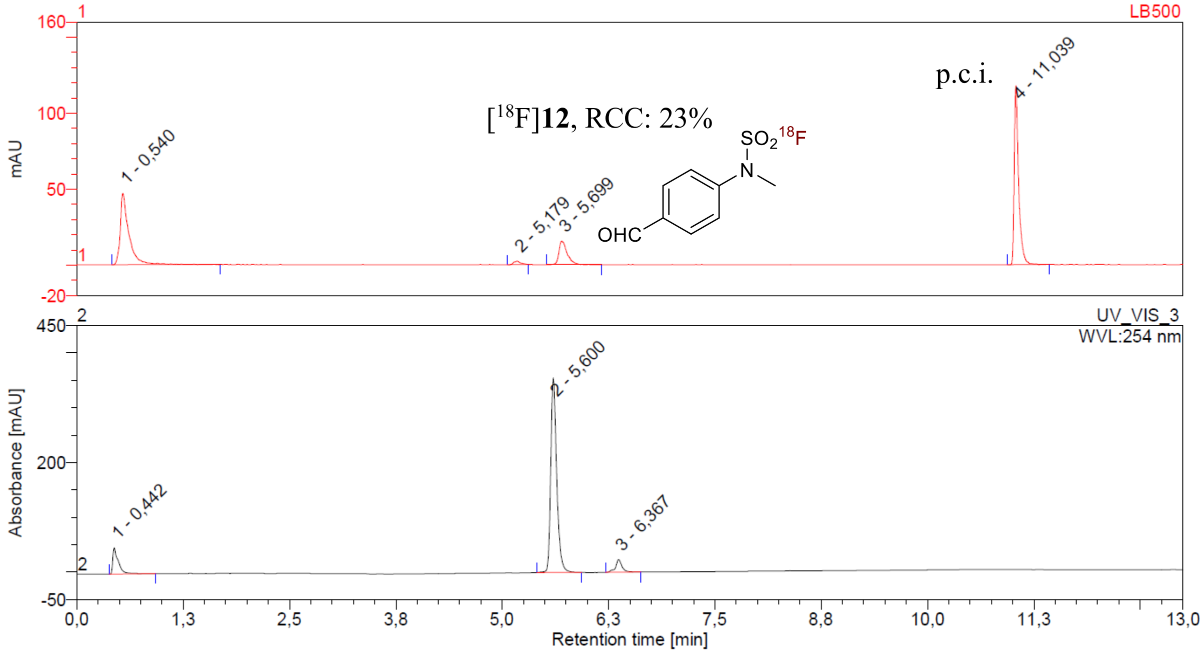

Eluent: 0–2 min: 10% MeCN, 2–10 min: 10 → 90% MeCN, 10–12.5 min: 90% MeCN, 12.5–13 min: 90 → 10% MeCN; flow-rate: 2 mL/min. Abbreviation: p.c.i. – post-column injection.

3,4-Dihydroquinoline-1(2*H*)-sulfonyl [^{18}F]fluoride ([^{18}F]**13**)

Column: Chromolith® SpeedROD RP-18e 50 × 4.6 mm (Merck KGaA, Darmstadt, Germany)

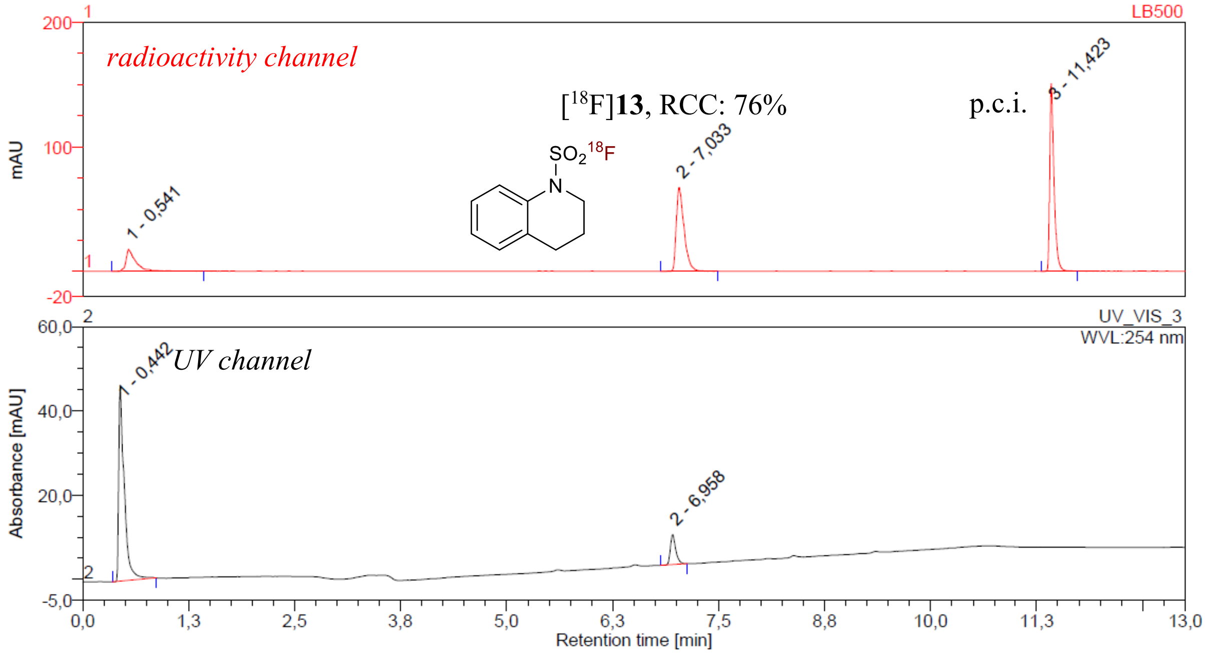

Eluent: 0–2 min: 10% MeCN, 2–10 min: 10 → 90% MeCN, 10–12.5 min: 90% MeCN, 12.5–13 min: 10 → 90% MeCN; flow-rate: 2 mL/min. Abbreviation: p.c.i. – post-column injection.

tert-Butyl (2*S*)-2-[(*tert*-butoxycarbonyl)amino]-3-{(*S*)-1-([^{18}F]fluorosulfonyl)indolin-3-yl}propanoate {(*S*,*S*)-[^{18}F]**14**}

Column: Chromolith® SpeedROD RP-18e 50 × 4.6 mm (Merck KGaA, Darmstadt, Germany)

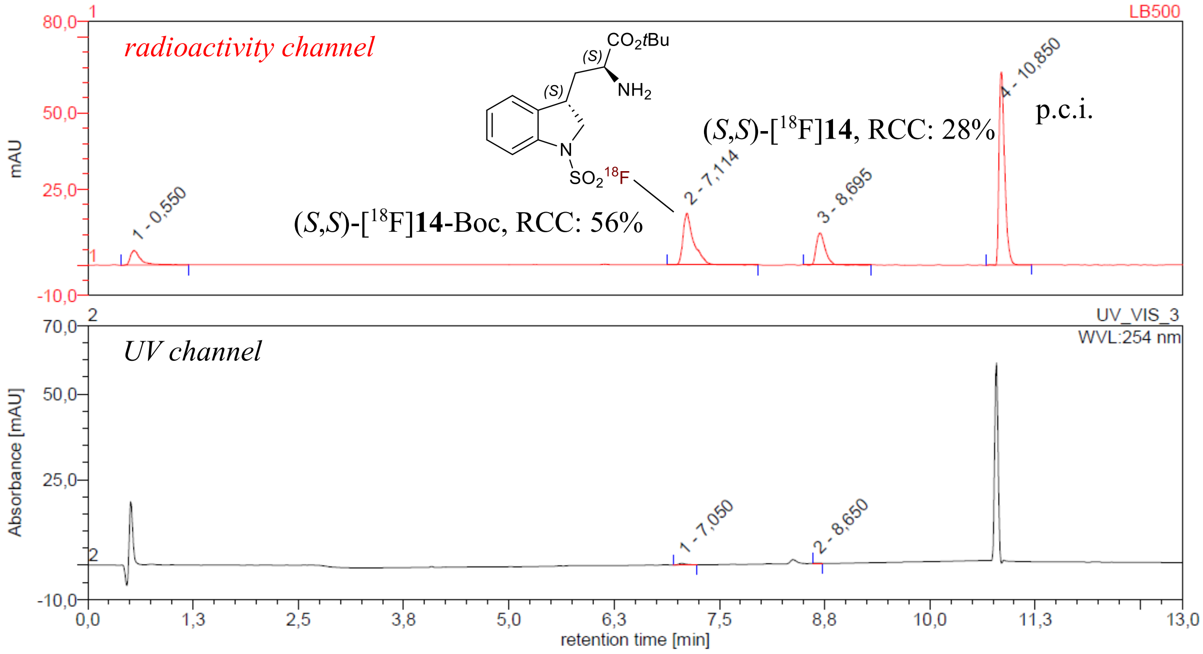

Eluent: 0–2 min: 10% MeCN, 2–10 min: 10 → 90% MeCN, 10–12.5 min: 90% MeCN, 12.5–13 min: 90 → 10% MeCN; flow-rate: 2 mL/min. Abbreviation: p.c.i. – post-column injection.

tert-Butyl (2*S*)-2-[(*tert*-butoxycarbonyl)amino]-3-{(*R*)-1-([^{18}F]fluorosulfonyl)indolin-3-yl}propanoate {(*S*,*R*)-[^{18}F]**14**}

Column: Chromolith® SpeedROD RP-18e 50 × 4.6 mm (Merck KGaA, Darmstadt, Germany)

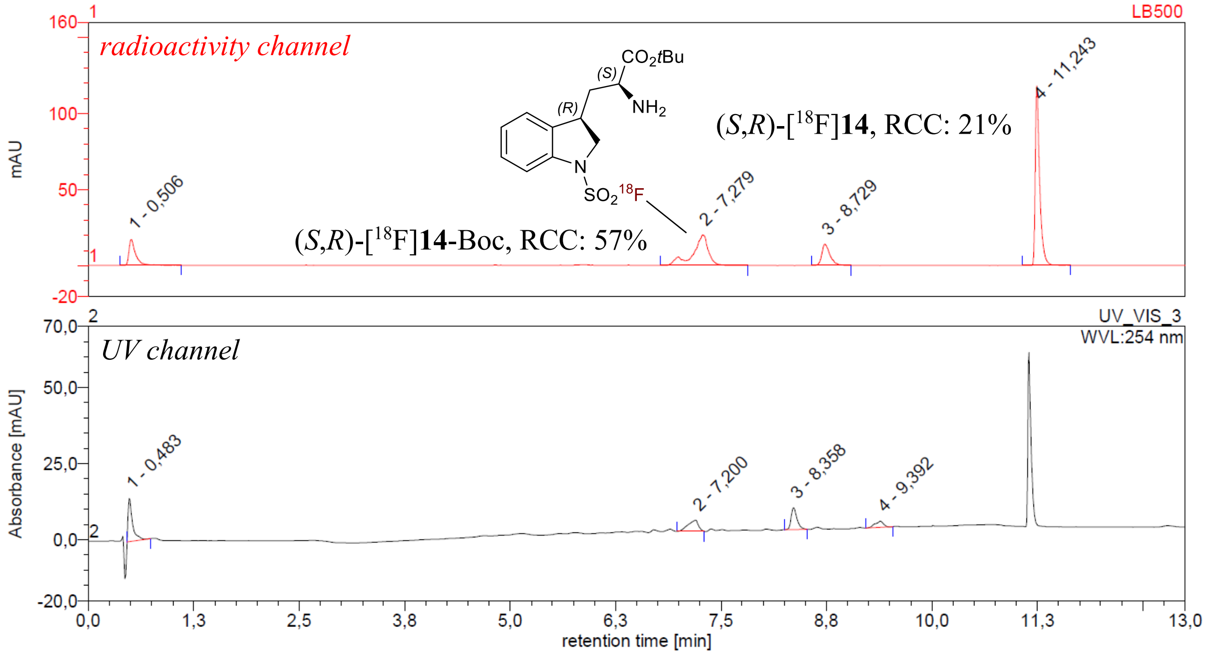

Eluent: 0–2 min: 10% MeCN, 2–10 min: 10 → 90% MeCN, 10–12.5 min: 90% MeCN, 12.5–13 min: 90 → 10% MeCN; flow-rate: 2 mL/min. Abbreviation: p.c.i. – post-column injection.

Methyl N_{α}-(*tert*-butoxycarbonyl)-N_{im}-([^{18}F]fluorosulfonyl)-L-histidinate ([^{18}F]**15**)

Column: Chromolith® SpeedROD RP-18e 50 × 4.6 mm (Merck KGaA, Darmstadt, Germany)

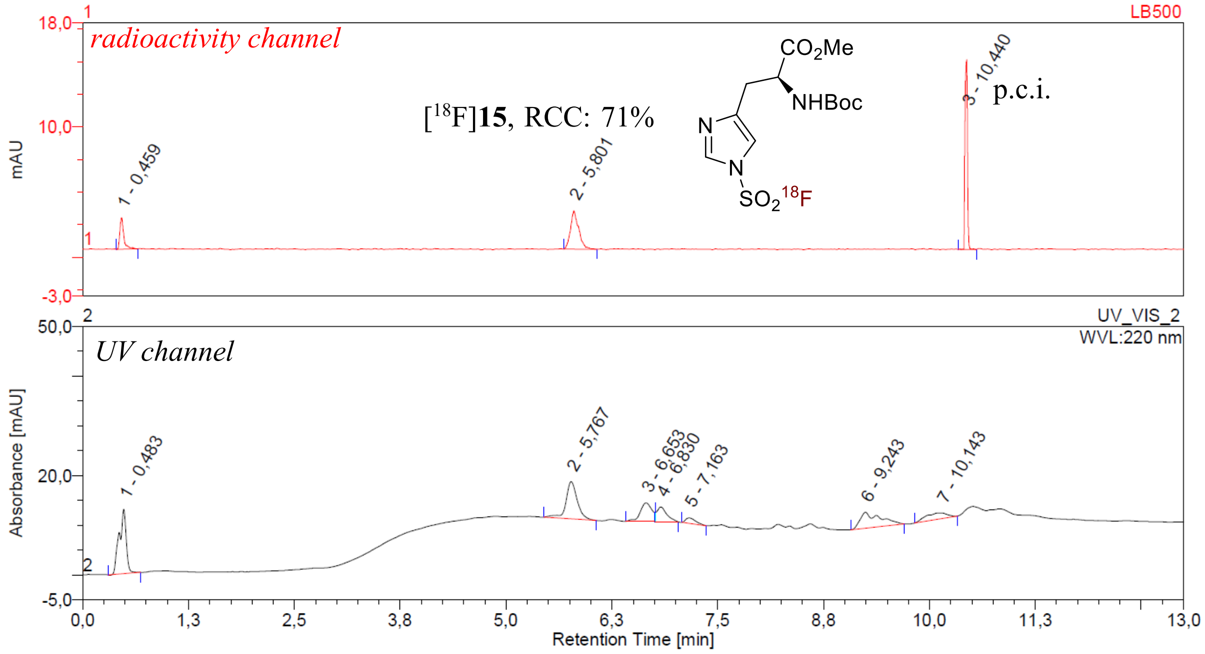

Eluent: 0–2 min: 10% MeCN, 2–10 min: 10 → 90% MeCN, 10–12.5 min: 90% MeCN, 12.5–13 min: 90 → 10% MeCN; flow-rate: 2 mL/min. Abbreviation: p.c.i. – post-column injection.

1,2,3,4-Tetrahydroisoquinoline-2-sulfonyl [^{18}F]fluoride ([^{18}F]**16**)

Column: Chromolith® SpeedROD RP-18e 50 × 4.6 mm (Merck KGaA, Darmstadt, Germany)

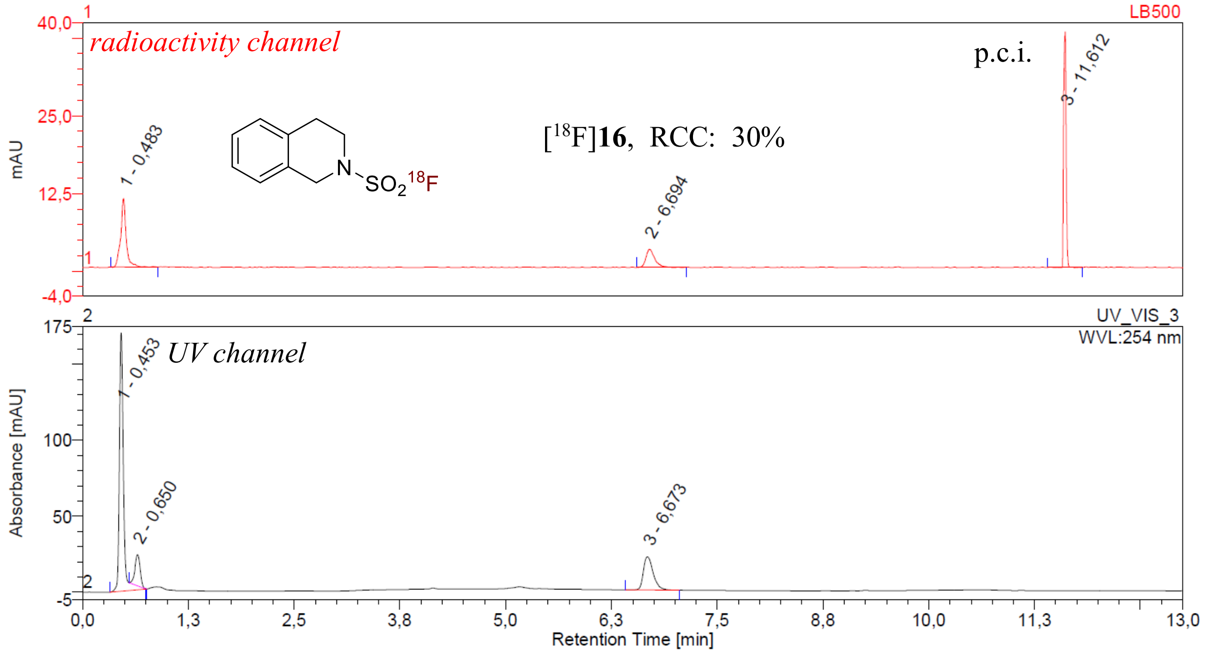

Eluent: 0–2 min: 10% MeCN, 2–10 min: 10 → 90% MeCN, 10–12.5 min: 90% MeCN, 12.5–13 min: 90 → 10% MeCN; flow-rate: 2 mL/min. Abbreviation: p.c.i. – post-column injection.

Phenyliminodisulfonyl [18F]difluoride ([18F]**17**)

Column: Chromolith® SpeedROD RP-18e 50 × 4.6 mm (Merck KGaA, Darmstadt, Germany)

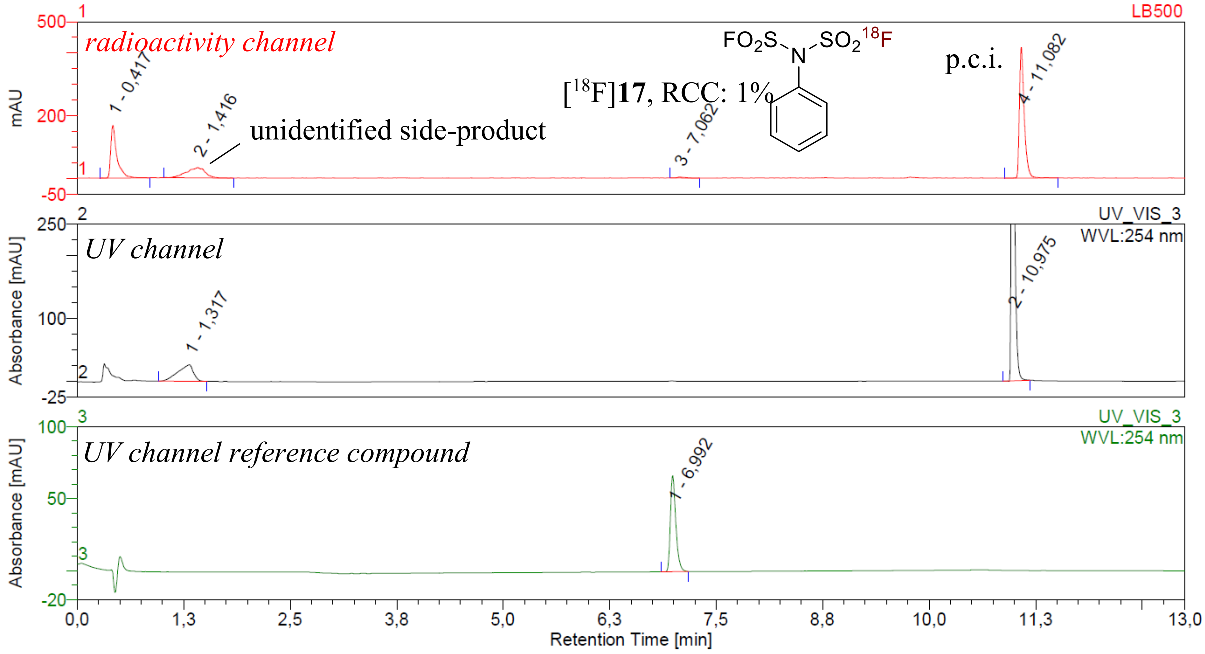

Eluent: 0–2 min: 10% MeCN, 2–10 min: 10 → 90% MeCN, 10–12.5 min: 90% MeCN, 12.5–13 min: 90 → 10% MeCN; flow-rate: 2 mL/min. Abbreviation: p.c.i. – post-column injection.

Chromatograms of purified compounds

N_α-(*tert*-Butoxycarbonyl)-1-([^{18}F]fluorosulfonyl)-L-tryptophan methyl ester ([^{18}F]**2**)

Column: MultoKrom® 100–5 C18 (250×2 mm) (CS-Chromatographie Service GmbH, Langerwehe, Germany).

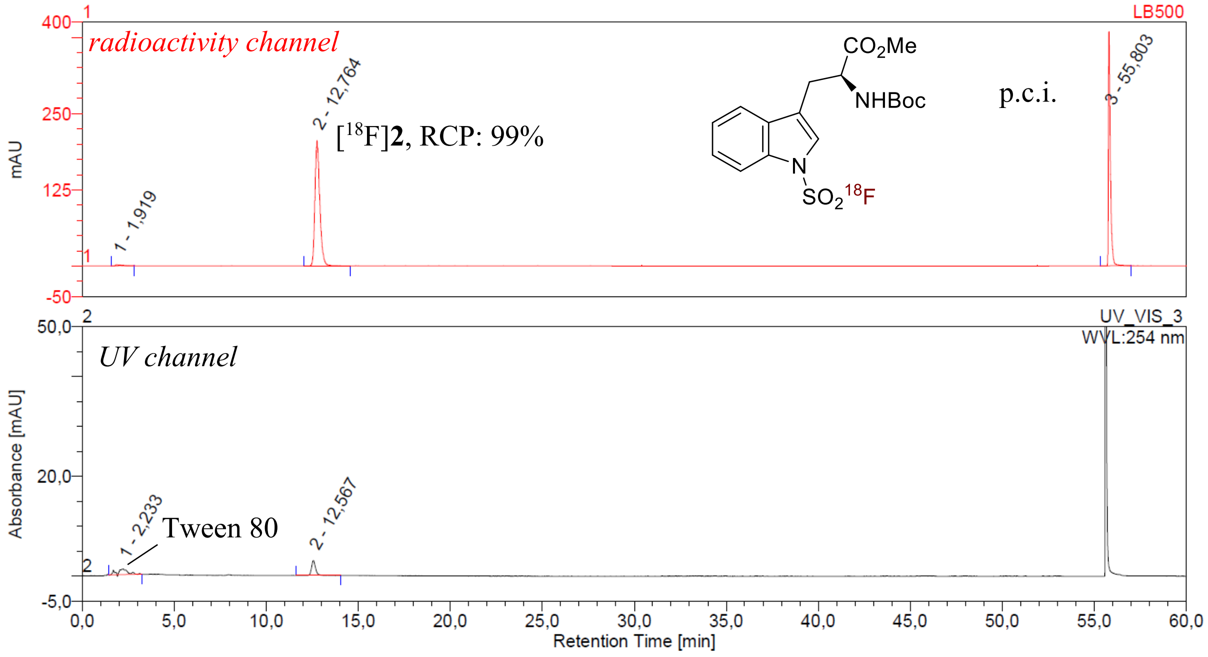

Eluent: 0–60 min: 70% MeCN; flow-rate: 1 mL/min. Abbreviation: p.c.i. – post-column injection.

1-([^{18}F]Fluorosulfonyl)-L-tryptophan ([^{18}F]**20**)

Column: MultoKrom® 100–5 C18 AQ (250×2 mm) (CS-Chromatographie Service GmbH, Langerwehe, Germany).

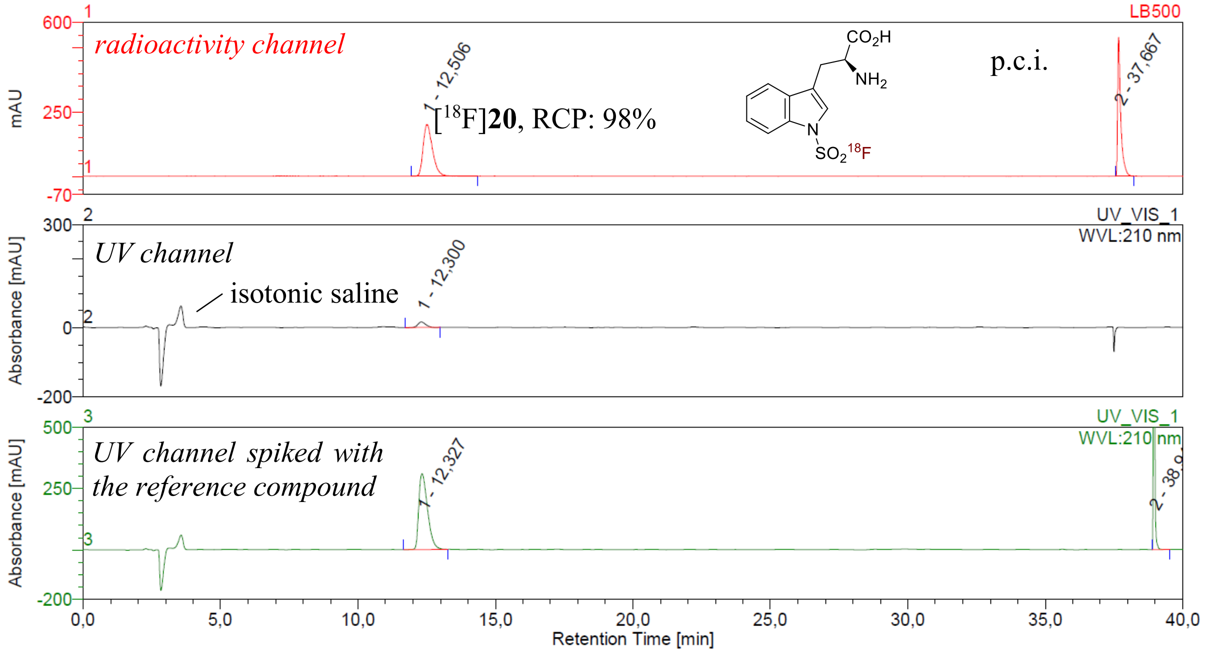

Eluent: 0–40 min: 30% MeCN (0.1% TFA); flow-rate: 1 mL/min. Abbreviation: p.c.i. – post-column injection.

(*S*)-2-Amino-3-{(*S*)-1-([^{18}F]fluorosulfonyl)indolin-3-yl}propanoic acid {(*S*,*S*)-[^{18}F]**21**}

Column: MultoKrom® 100–5 C18 AQ (250×2 mm) (CS-Chromatographie Service GmbH, Langerwehe, Germany).

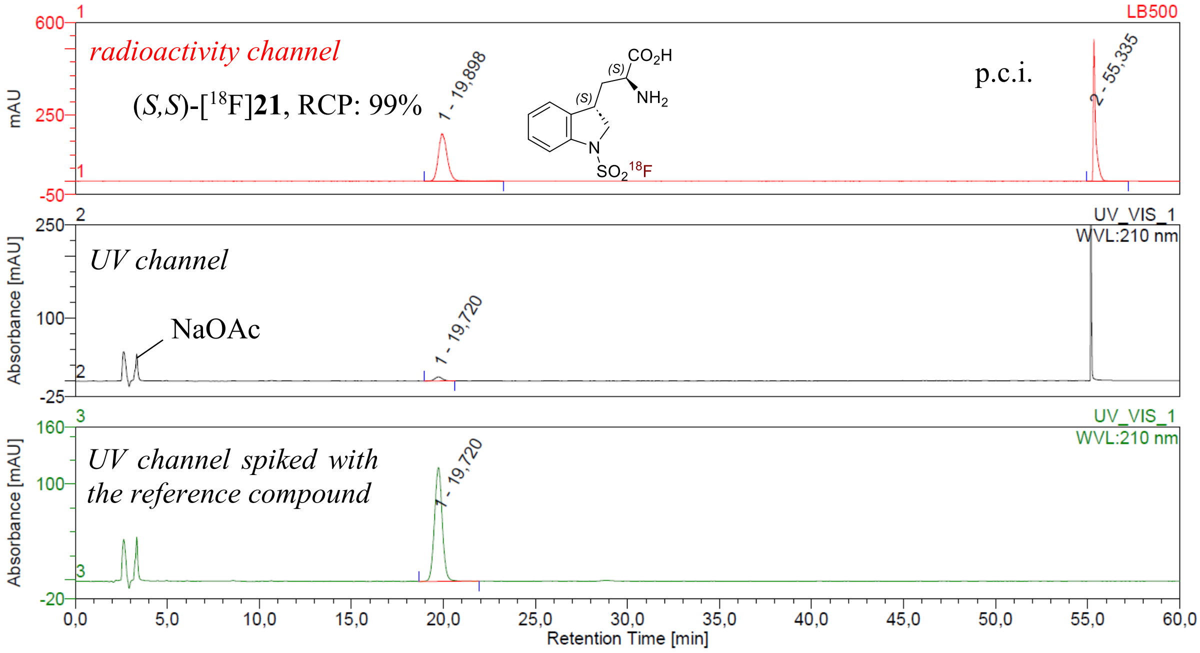

Eluent: 0 – 60 min: 30% EtOH in 0.01 M NaOAc-buffer (pH 5.3); flow-rate: 1 mL/min. Abbreviation: p.c.i. – post-column injection.

(*S*)-2-Amino-3-{(*R*)-1-([^{18}F]fluorosulfonyl)indolin-3-yl}propanoic acid {(*S*,*R*)-[^{18}F]**21**}

Column: MultoKrom® 100–5 C18 AQ (250×2 mm) (CS-Chromatographie Service GmbH, Langerwehe, Germany).

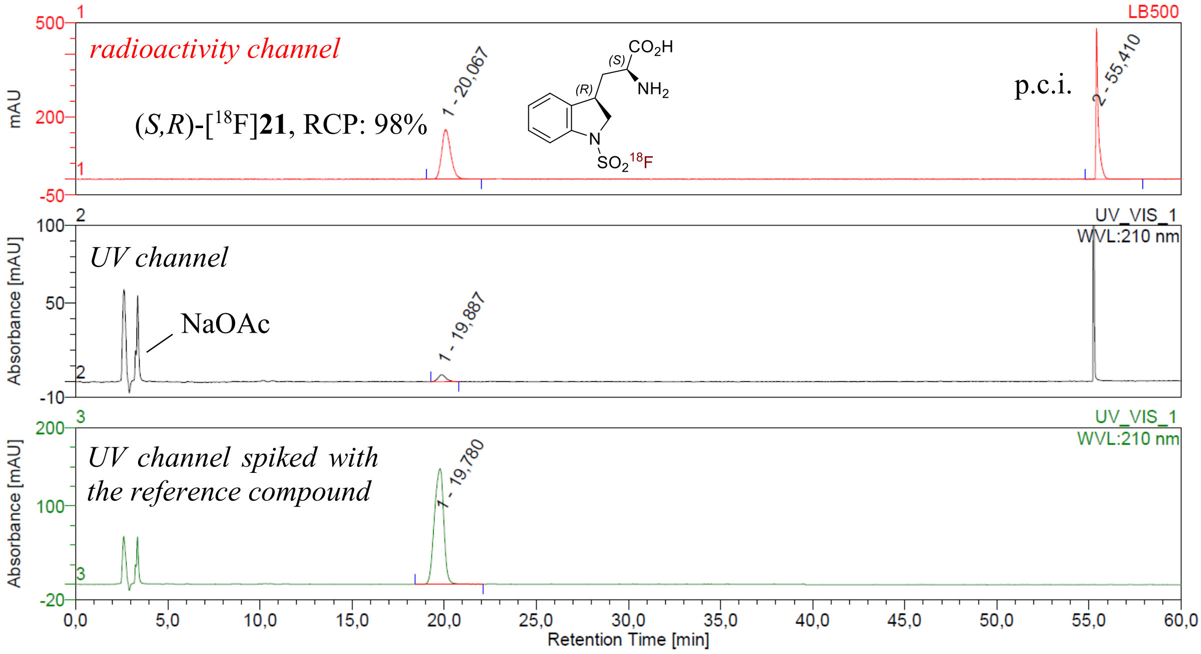

Eluent: 0 – 60 min: 30% EtOH in 0.01 M NaOAc-buffer (pH 5.3); flow-rate: 1 mL/min. Abbreviation: p.c.i. – post-column injection.

(*S*)-2-Amino-3-[1-([^{18}F]fluorosulfonyl)-1*H*-indol-5-yl]propanoic acid hydrochloride ([^{18}F]**26**)

Column: MultoKrom® 100–5 C18 AQ (250×2 mm) (CS-Chromatographie Service GmbH, Langerwehe, Germany).

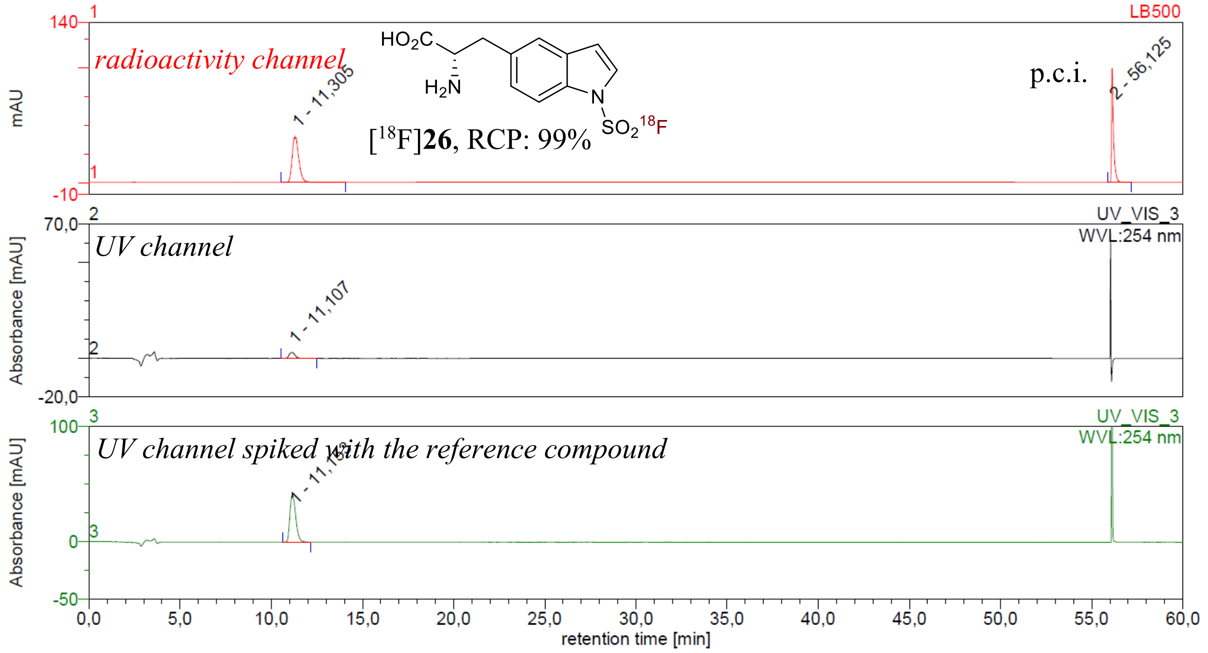

Eluent: 0–60 min: 30% MeCN (0.1% TFA); flow-rate: 1 mL/min. Abbreviation: p.c.i. – post-column injection.

(*S*)-2-Amino-3-{4-[([^{18}F]fluorosulfonyl)(methyl)amino]phenyl}propanoic acid ([^{18}F]**33**)

Column: MultoKrom® 100–5 C18 AQ (250×2 mm) (CS-Chromatographie Service GmbH, Langerwehe, Germany).

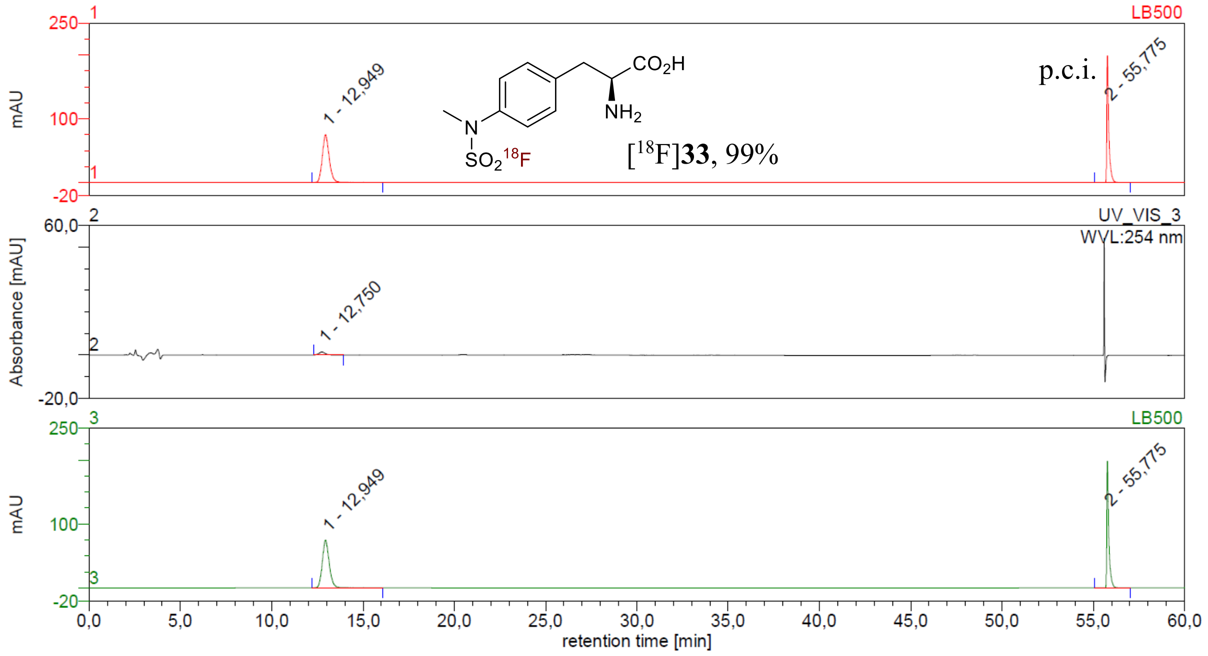

Eluent: 0–60 min: 25% MeCN (0.1% TFA); flow-rate: 1 mL/min. Abbreviation: p.c.i. – post-column injection.

Determination of molar activity of [^{18}F]2

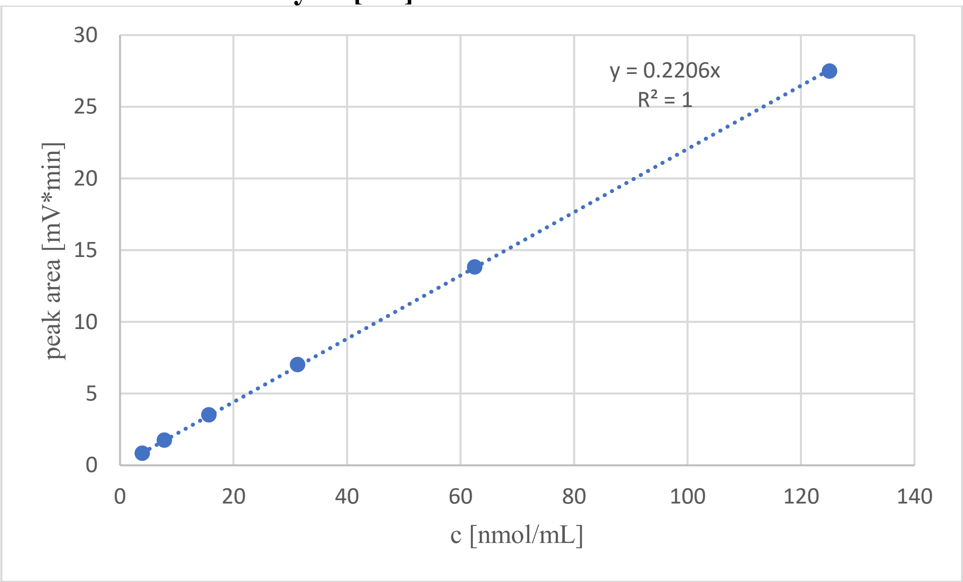

Figure S3: Calibration curve for determination of molar activity.

Table S1: Data for determination of molar activity

formulation volume [mL]	1
peak area [mV*min]	5.932
activity concentration (MBq/mL)	188.14
amount of substance used [nmol]	30
amount of substance determined [nmol/mL]	27
final tracer activity [MBq]	200
activity yield [%]	28
molar activity calculated** [GBq/µmol]	**6.67**
molar activity determined*** [GBq/µmol]	**6.99**
deviation of calc. & deter. A_m	4.7%

*calculated from used precursor amount and final tracer activity.
**determined with calibration curve and final activity concentration.

Radio-TLCs pH stability tests (after 120 min)

Table S2: Aqueous solutions used for hydrolytic stability test at different pH

pH	1	2	5	7.4	8.5	10	12
Aqueous solution	0.55% TFA	0.1% TFA	0.1 M NaOAc-buffer	1×PBS-buffer*	0.1 M $NaHCO_3$	VWR-buffer**	0.01 M NaOH

*Gibco™ PBS, pH 7,4, ×1;

** VWR® AVS TITRINORM Buffer pH 10.0

Indoline-1-sulfonyl [^{18}F]fluoride ([^{18}F]**3**)

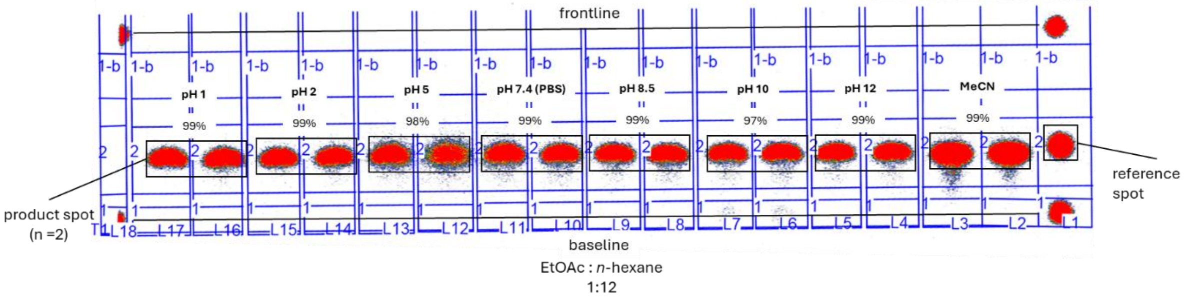

4-Phenylpiperidine-1-sulfonyl [^{18}F]fluoride ([^{18}F]**4**)

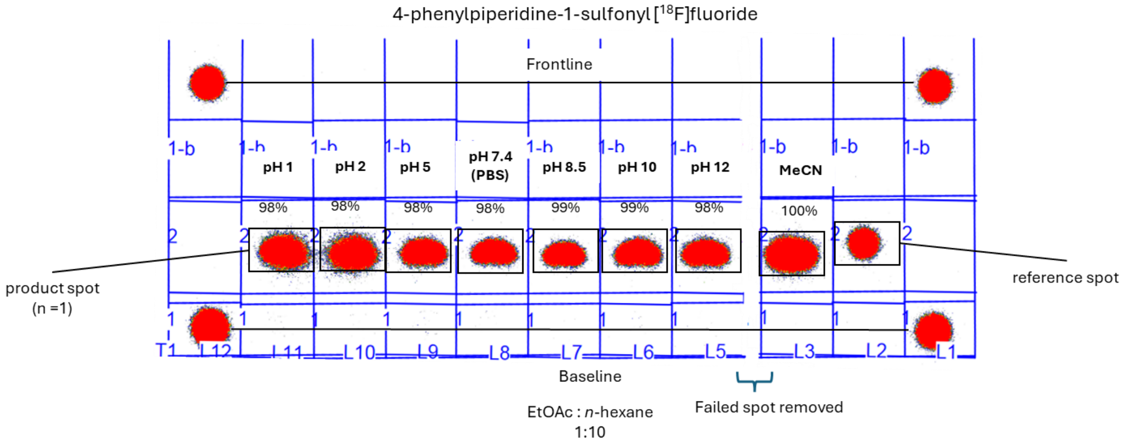

9*H*-Carbazole-9-sulfonyl [^{18}F]fluoride ([^{18}F]**5**)

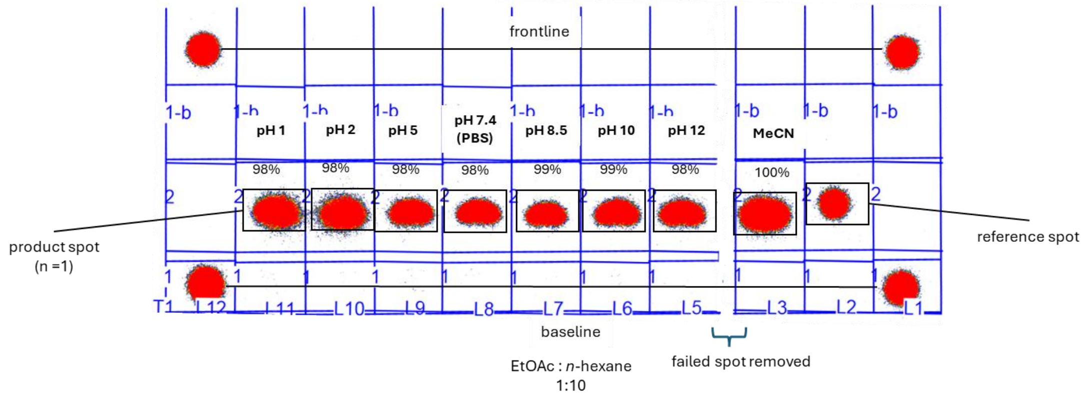

Methyl N_{α}-(*tert*-butoxycarbonyl)-N_{im}-([^{18}F]fluorosulfonyl)-L-histidinate ([^{18}F]**15**)

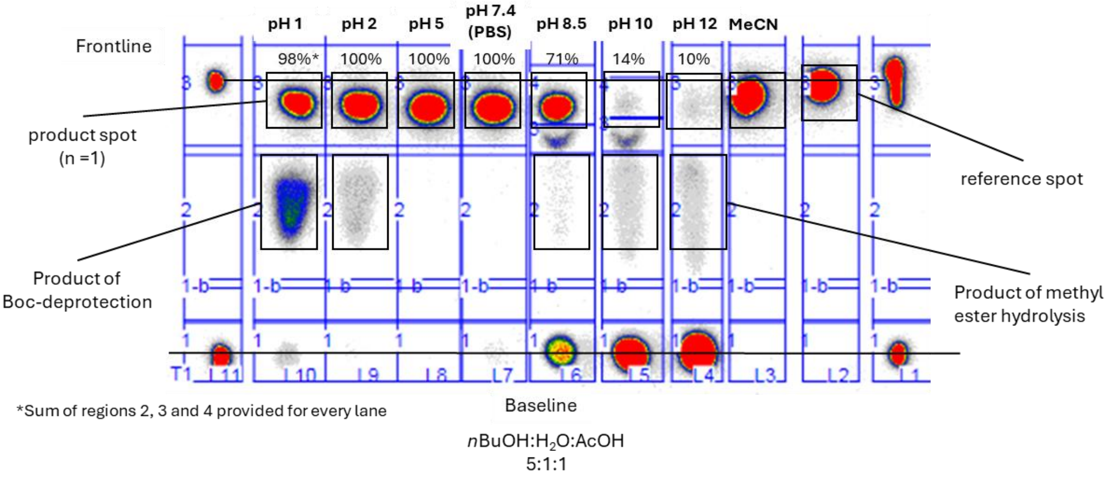

1-([^{18}F]Fluorosulfonyl)-L-tryptophan ([^{18}F]**20**)

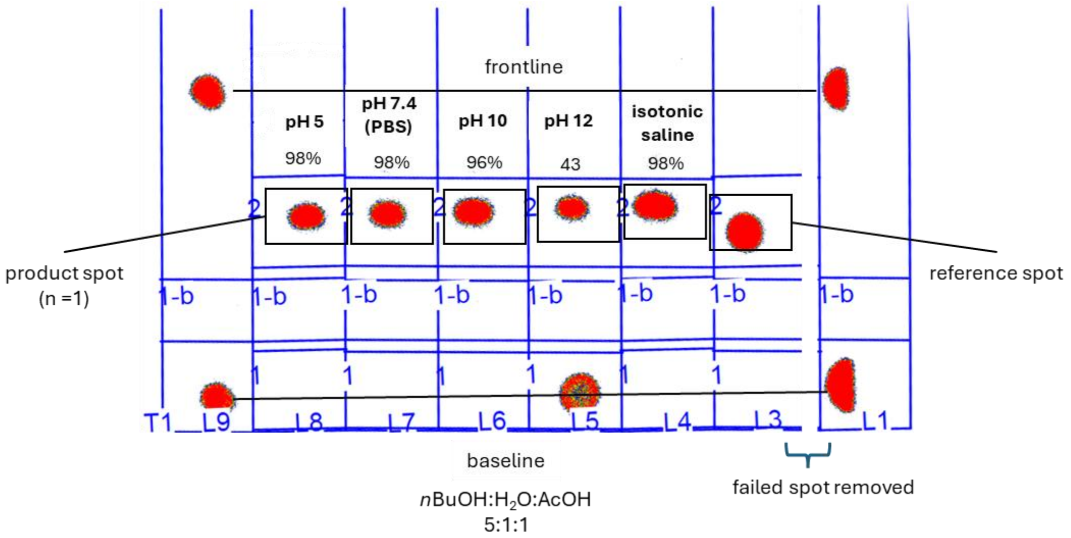

(*S*)-2-Amino-3-{(*S*)-1-([^{18}F]fluorosulfonyl)indolin-3-yl}propanoic acid {(*S*,*S*)-[^{18}F]**21**}

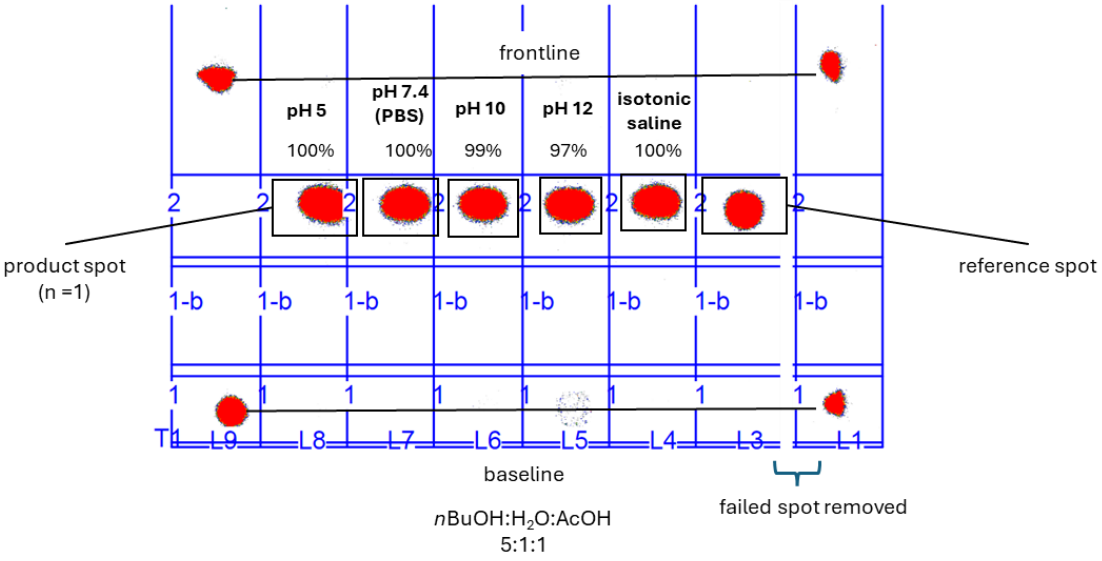

Radio-TLCs: Blood serum stability tests (after 120 min)

1-([^{18}F]Fluorosulfonyl)-L-tryptophan ([^{18}F]**20**)

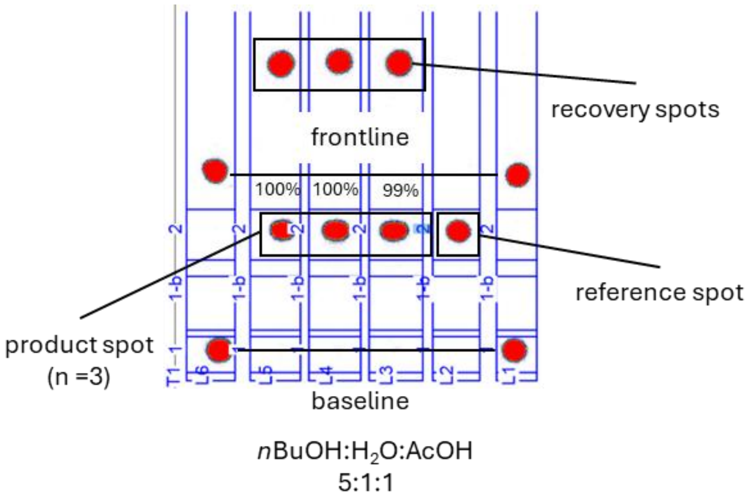

(*S*)-2-Amino-3-{(*S*)-1-([^{18}F]fluorosulfonyl)indolin-3-yl}propanoic acid {(*S*,*S*)-[^{18}F]**21**}

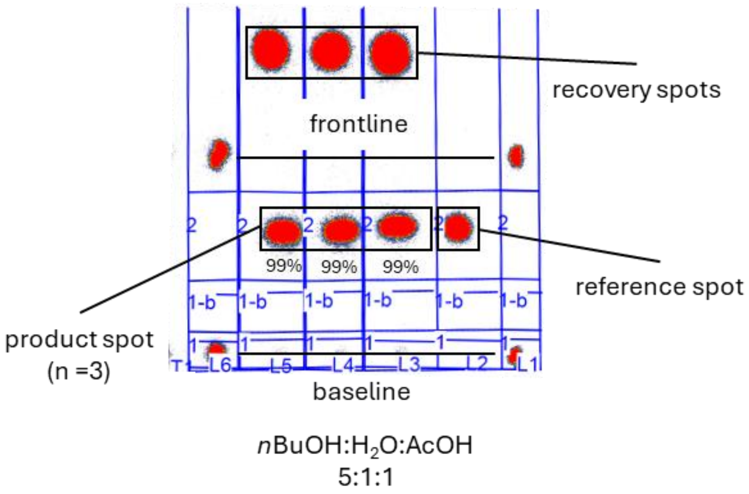

Hydrolytic stability of [^{18}F]15

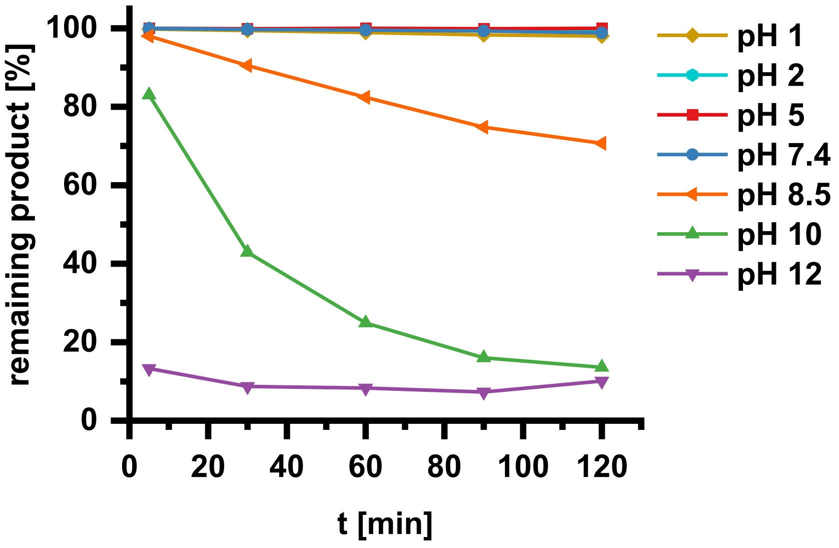

Figure S4: Hydrolytic stability of [^{18}F]**15** at different pH values (1–12) over 2 h. [^{18}F]**15** was prepared and isolated as described for Boc-Trp(SO2[^{18}F]F)-OMe (see section 4.2.4 in the manuscript) using 100 nmol precursor and evaluated as described in section 4.3.1 of the manuscript.

Ester cleavage during labeling of methyl ester 2

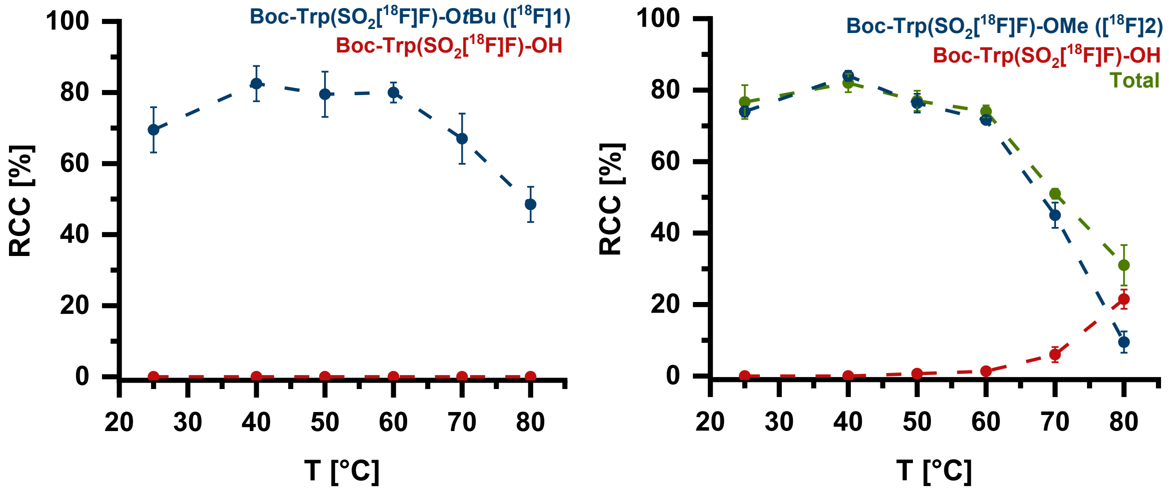

Figure S5: Radiochemical conversions (RCCs) for Boc-Trp(SO2[^{18}F]F)-O*t*Bu ([^{18}F]**1**, left), Boc-Trp(SO2[^{18}F]F)-OMe ([^{18}F]**2**, right), and the corresponding free carboxylic acid Boc-Trp(SO2[^{18}F]F)-OH obtained during labeling of **1** and **2** for 5 min at different temperatures. Conditions: 30 nmol precursor, 1 mL MeCN, 10 µmol elution salt in 1 mL MeOH.

Blood time-activity-curve (TAC) for experiments shown in Fig.4

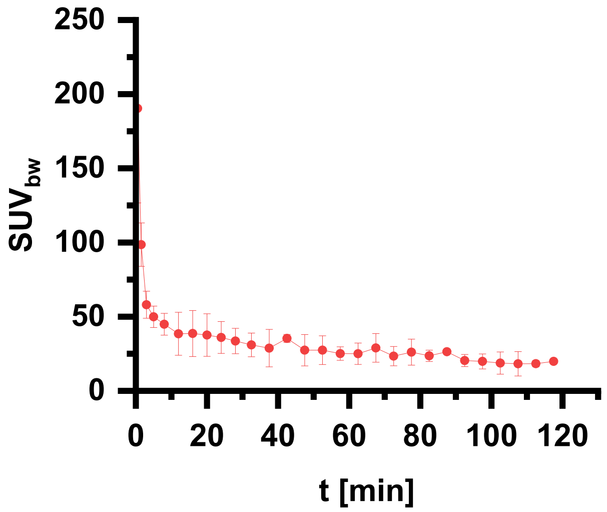

Figure S6: Blood time-activity curve (TAC) for Boc-Trp(SO2[^{18}F]F)-OMe ([^{18}F]**2**) in healthy mice. Data are shown as mean ± standard deviation (n = 3). For details and TACs for other tissues, see Fig. 4 in the main article.

References

(1) van Puffelen, B.; Lu, Y.; Minnee, H.; Schuller, M.; van der Marel, G. A.; Ahel, I.; Codée, J. D. C.; Filippov, D. V. Synthesis of Adenosine Diphosphate Ribose Histidine. *Organic Letters* **2026**, *28* (3), 917-922. DOI: https://doi.org/10.1021/acs.orglett.5c04626.

(2) Verschueren, R. H.; Gilles, P.; Van Mileghem, S.; De Borggraeve, W. M. Solvent-free *N*-Boc deprotection by ex situ generation of hydrogen chloride gas. *Organic & Biomolecular Chemistry* **2021**, *19* (26), 5782-5787,. DOI: https://doi.org/10.1039/D1OB00728A.
